# Supplementary figures and images for: Motor neurons in the escape response circuit of white shrimp (Litopenaeus setiferus) (part 2 of 4)
Source: PeerJ. 2015 Jul 21;3:e1112. doi: 10.7717/peerj.1112 (PMC4517965; doi:10.7717/peerj.1112)

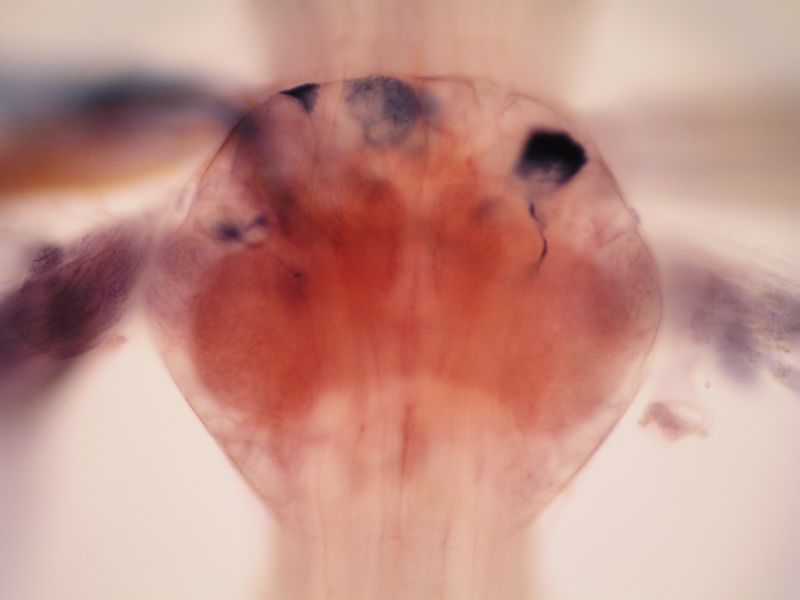

Supplement: Supplemental Information 2 — Micrographs of N2 backfills. Images have been reduced in size. [file peerj-03-1112-s002.zip › A2N2 2009 06 21 d.jpg]

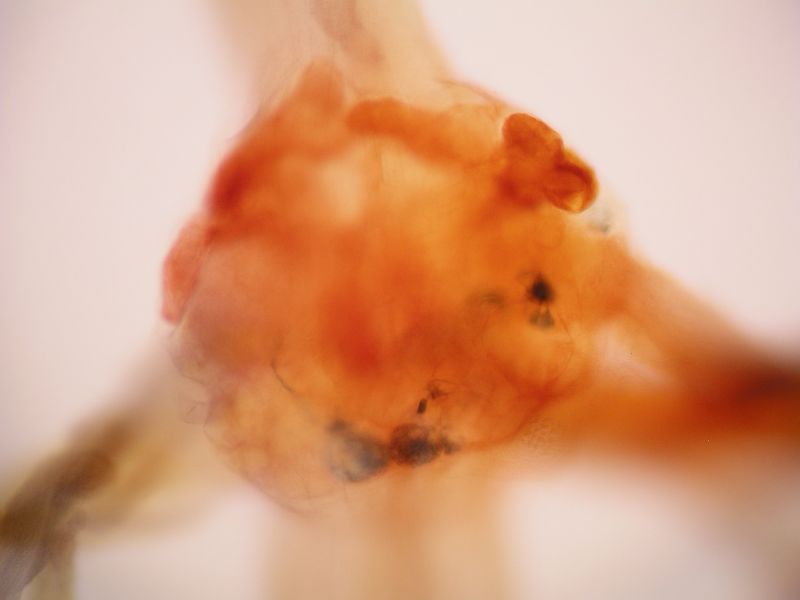

Supplement: Supplemental Information 2 — Micrographs of N2 backfills. Images have been reduced in size. [file peerj-03-1112-s002.zip › A2N2 2009 07 03a.jpg]

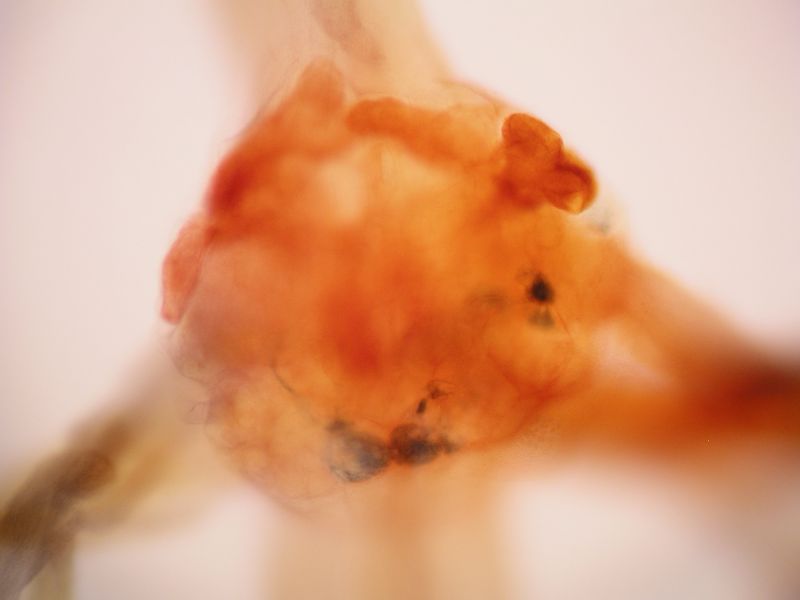

Supplement: Supplemental Information 2 — Micrographs of N2 backfills. Images have been reduced in size. [file peerj-03-1112-s002.zip › A2N2 2009 07 03b.jpg]

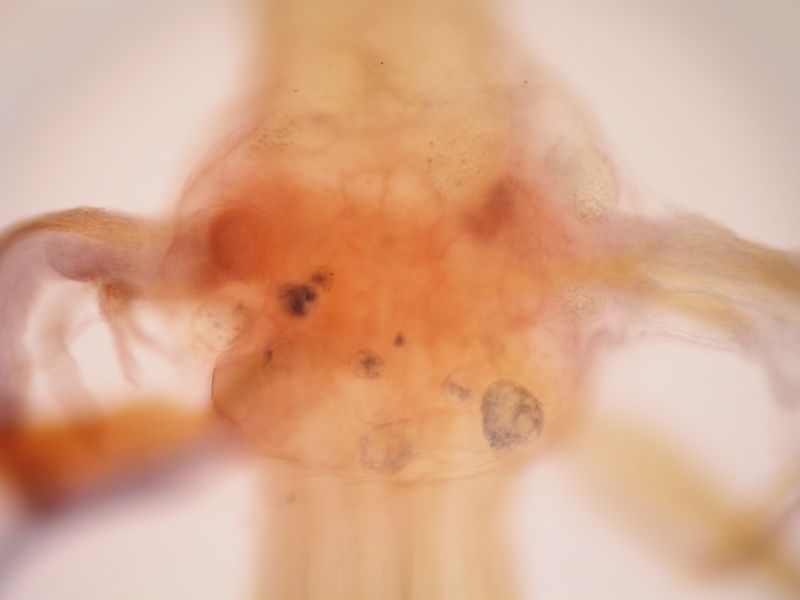

Supplement: Supplemental Information 2 — Micrographs of N2 backfills. Images have been reduced in size. [file peerj-03-1112-s002.zip › A2N2 2009 07 04 a.jpg]

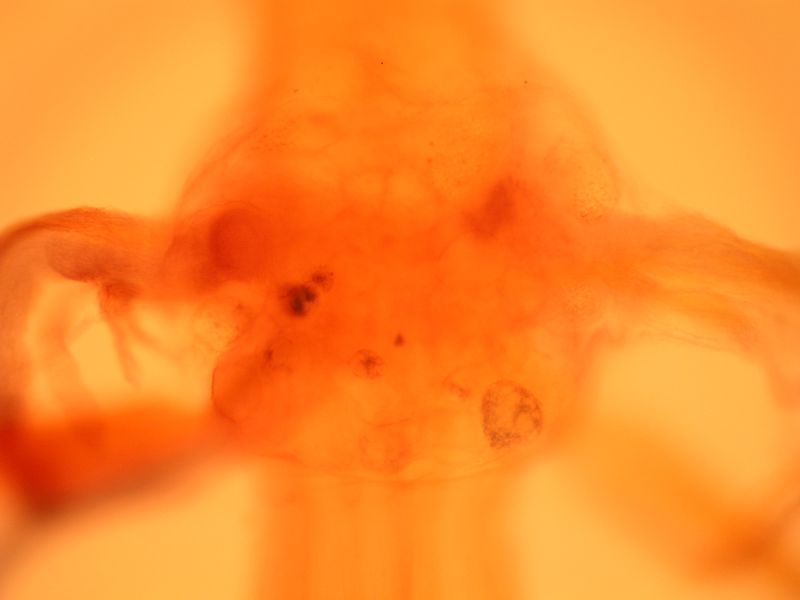

Supplement: Supplemental Information 2 — Micrographs of N2 backfills. Images have been reduced in size. [file peerj-03-1112-s002.zip › A2N2 2009 07 04 b.jpg]

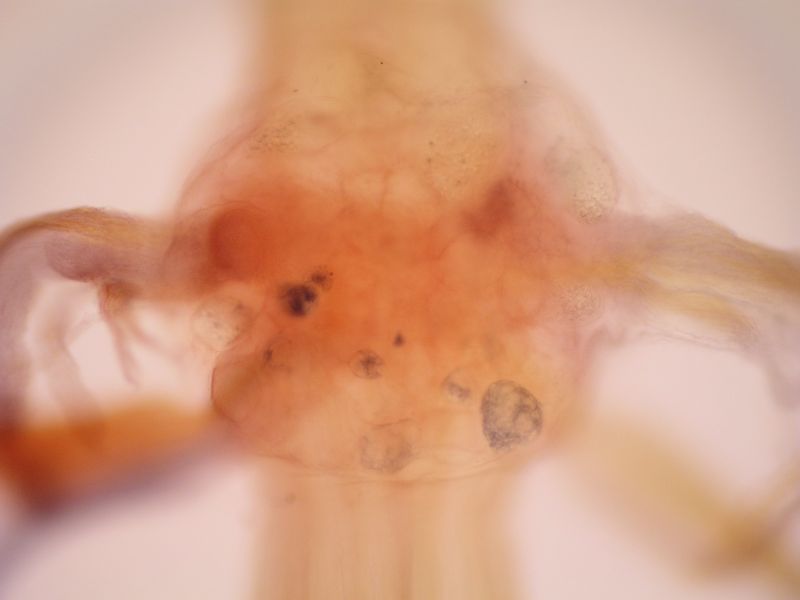

Supplement: Supplemental Information 2 — Micrographs of N2 backfills. Images have been reduced in size. [file peerj-03-1112-s002.zip › A2N2 2009 07 04 c.jpg]

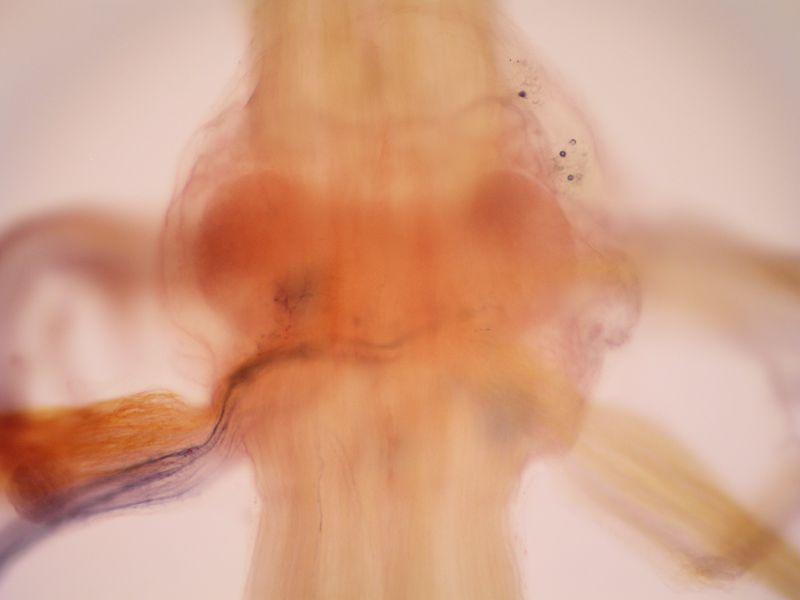

Supplement: Supplemental Information 2 — Micrographs of N2 backfills. Images have been reduced in size. [file peerj-03-1112-s002.zip › A2N2 2009 07 04 d.jpg]

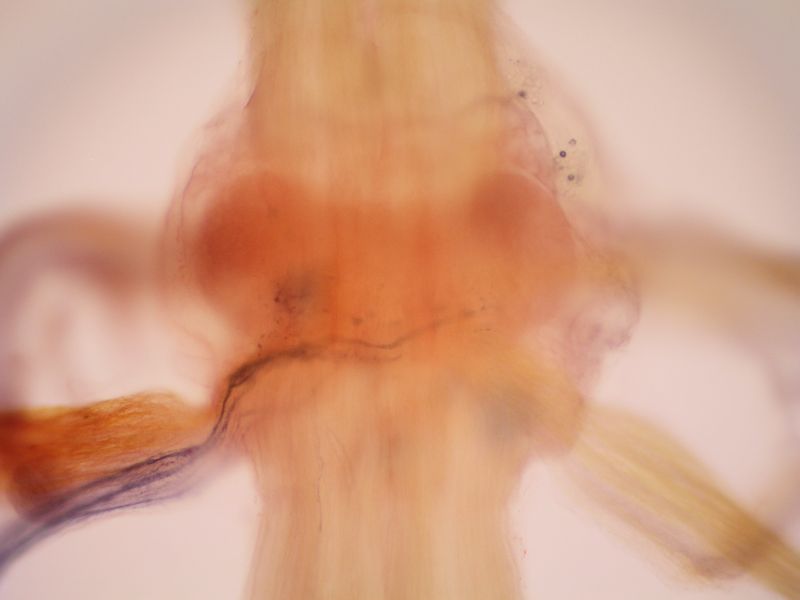

Supplement: Supplemental Information 2 — Micrographs of N2 backfills. Images have been reduced in size. [file peerj-03-1112-s002.zip › A2N2 2009 07 04 e.jpg]

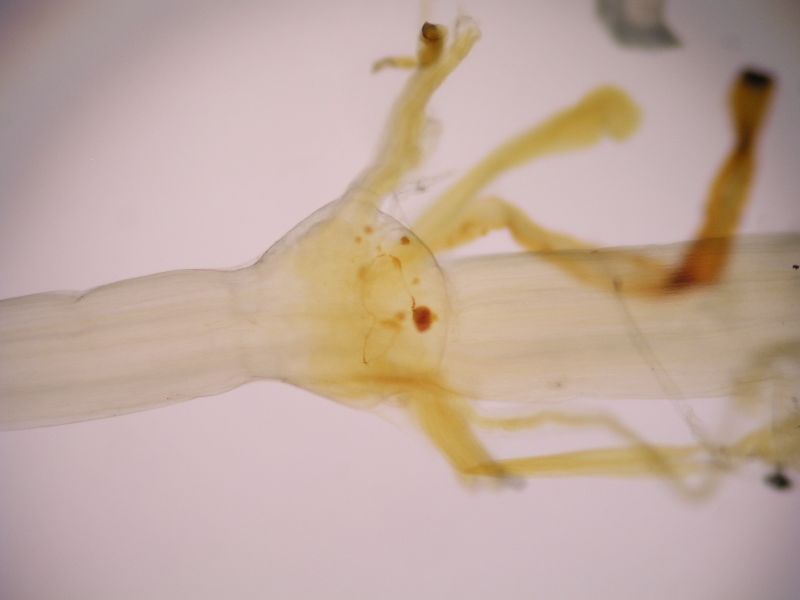

Supplement: Supplemental Information 2 — Micrographs of N2 backfills. Images have been reduced in size. [file peerj-03-1112-s002.zip › A2N2p 2009 06 18 a.jpg]

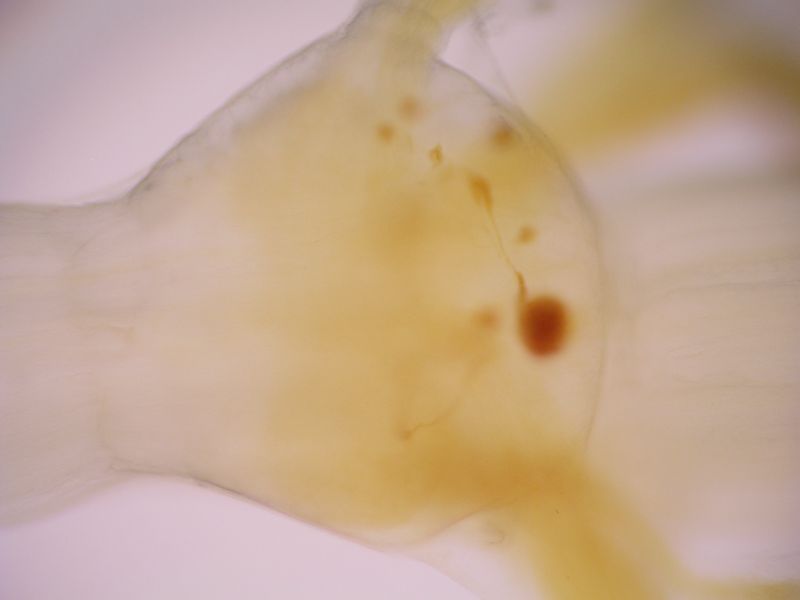

Supplement: Supplemental Information 2 — Micrographs of N2 backfills. Images have been reduced in size. [file peerj-03-1112-s002.zip › A2N2p 2009 06 18 b.jpg]

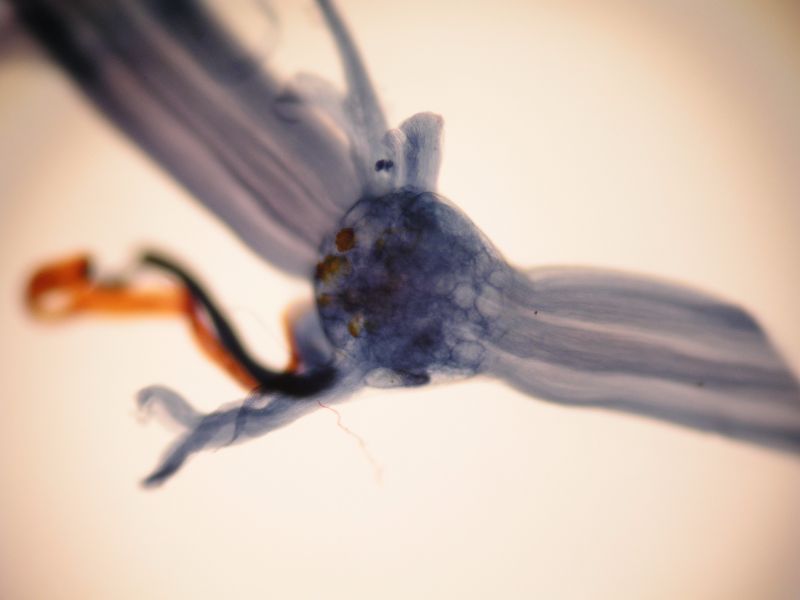

Supplement: Supplemental Information 2 — Micrographs of N2 backfills. Images have been reduced in size. [file peerj-03-1112-s002.zip › A2N2p 2009 06 19 a.jpg]

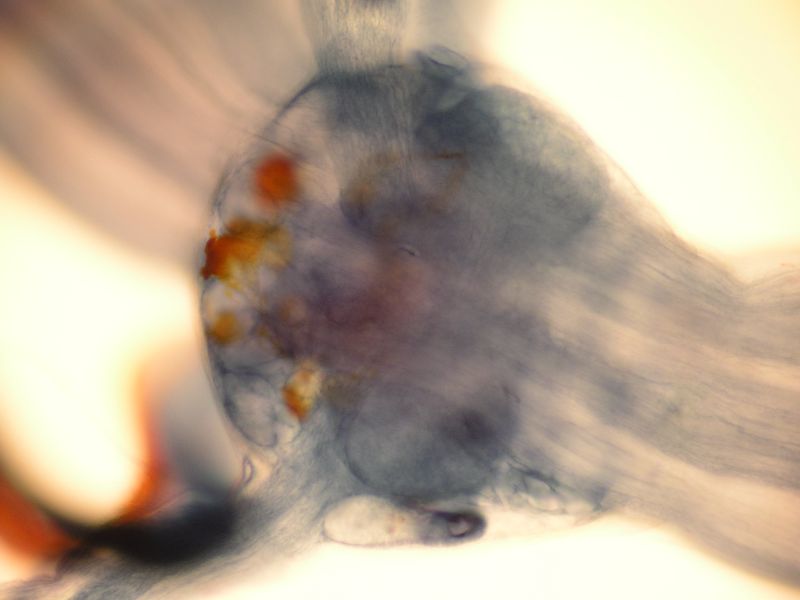

Supplement: Supplemental Information 2 — Micrographs of N2 backfills. Images have been reduced in size. [file peerj-03-1112-s002.zip › A2N2p 2009 06 19 b.jpg]

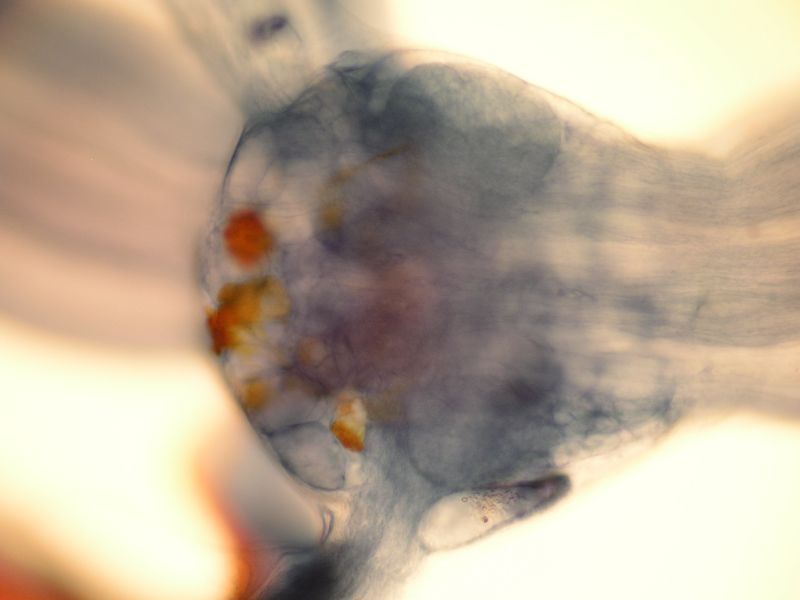

Supplement: Supplemental Information 2 — Micrographs of N2 backfills. Images have been reduced in size. [file peerj-03-1112-s002.zip › A2N2p 2009 06 19 c.jpg]

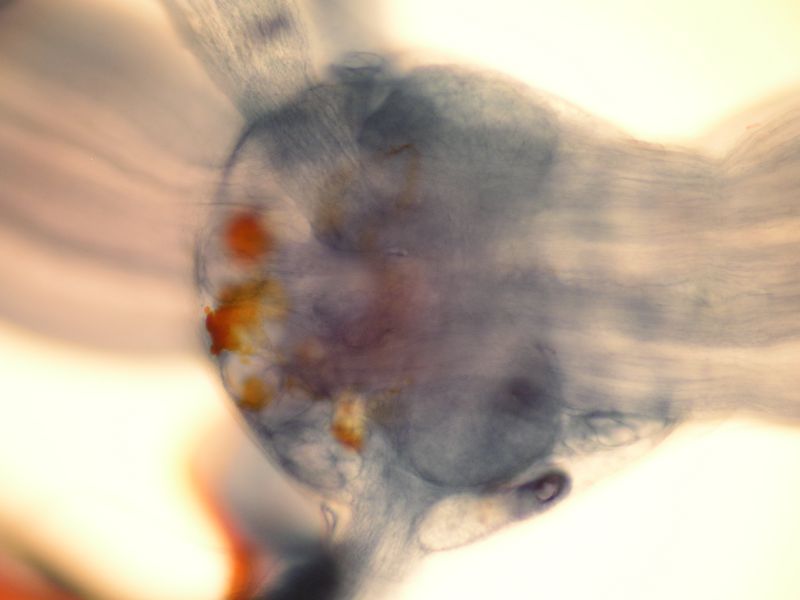

Supplement: Supplemental Information 2 — Micrographs of N2 backfills. Images have been reduced in size. [file peerj-03-1112-s002.zip › A2N2p 2009 06 19 d.jpg]

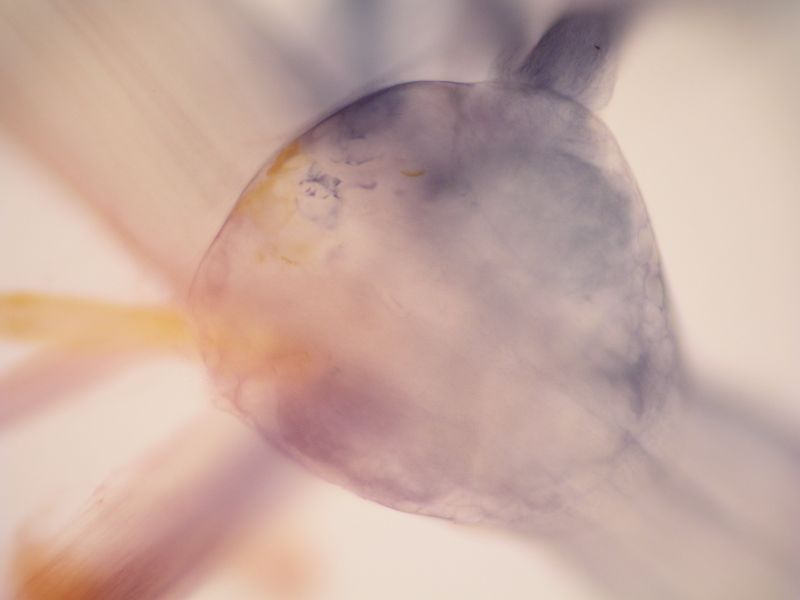

Supplement: Supplemental Information 2 — Micrographs of N2 backfills. Images have been reduced in size. [file peerj-03-1112-s002.zip › A3 P1010032.jpg]

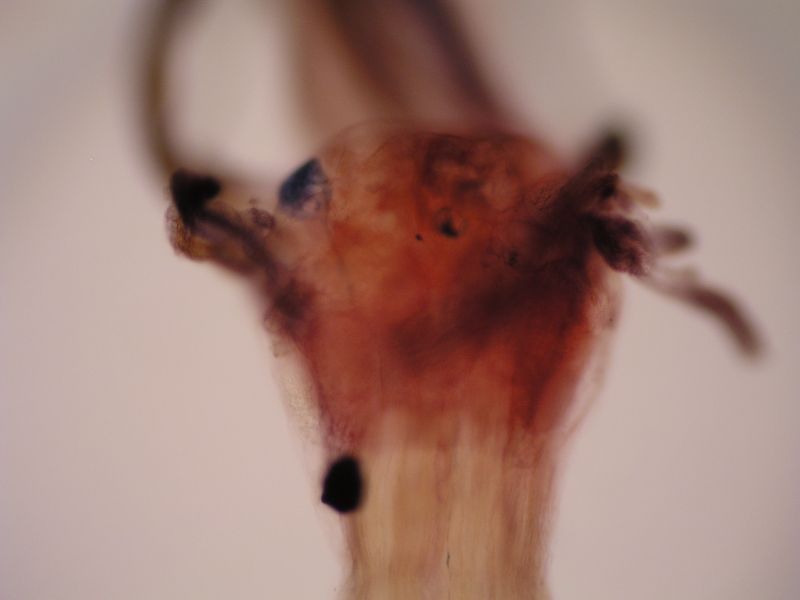

Supplement: Supplemental Information 2 — Micrographs of N2 backfills. Images have been reduced in size. [file peerj-03-1112-s002.zip › A3N2 2009 07 04 a.jpg]

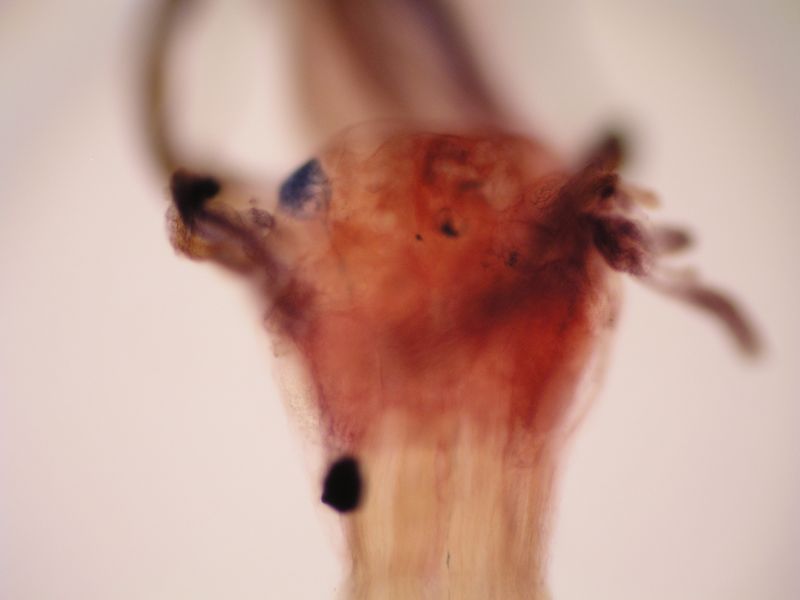

Supplement: Supplemental Information 2 — Micrographs of N2 backfills. Images have been reduced in size. [file peerj-03-1112-s002.zip › A3N2 2009 07 04 b.jpg]

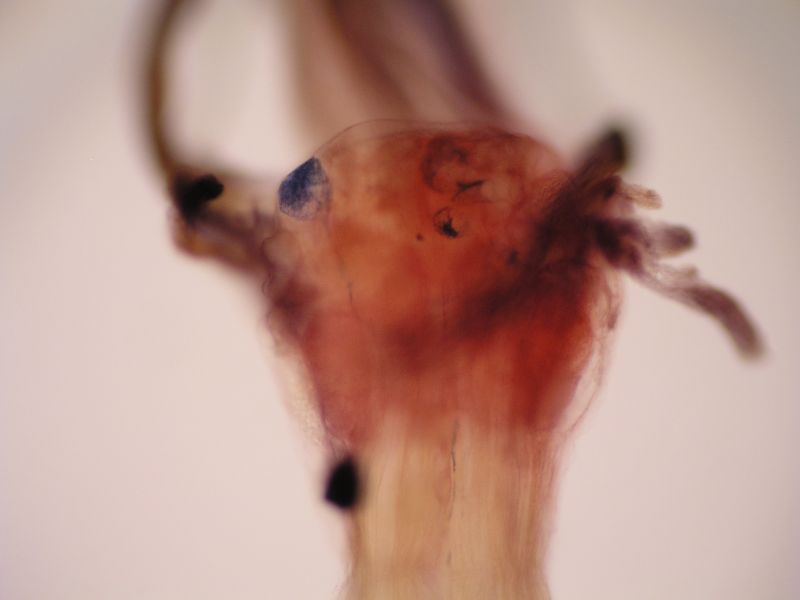

Supplement: Supplemental Information 2 — Micrographs of N2 backfills. Images have been reduced in size. [file peerj-03-1112-s002.zip › A3N2 2009 07 04 c.jpg]

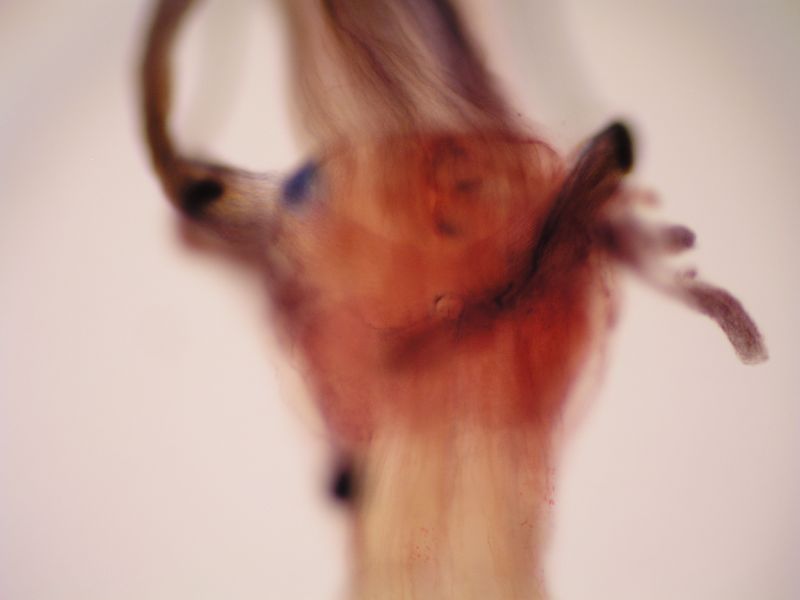

Supplement: Supplemental Information 2 — Micrographs of N2 backfills. Images have been reduced in size. [file peerj-03-1112-s002.zip › A3N2 2009 07 04 d.jpg]

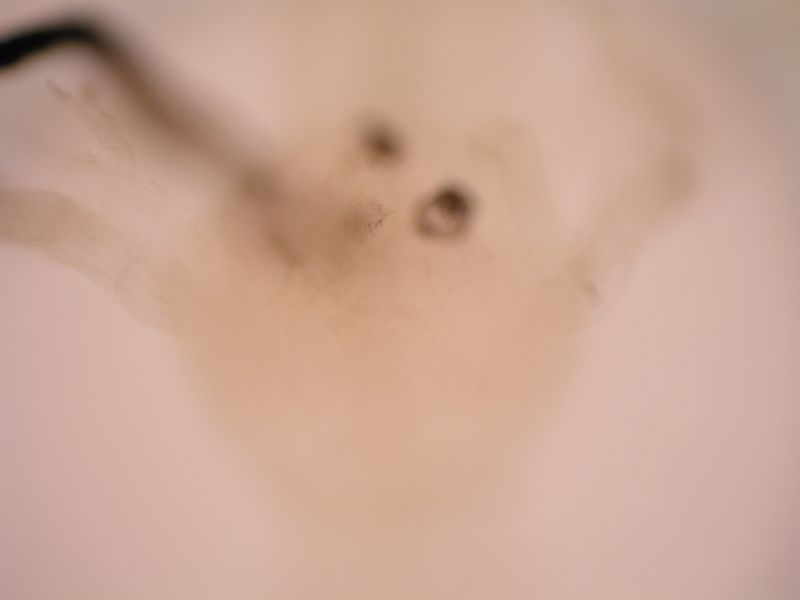

Supplement: Supplemental Information 2 — Micrographs of N2 backfills. Images have been reduced in size. [file peerj-03-1112-s002.zip › A3N2p 2009 07 13 (1).jpg]

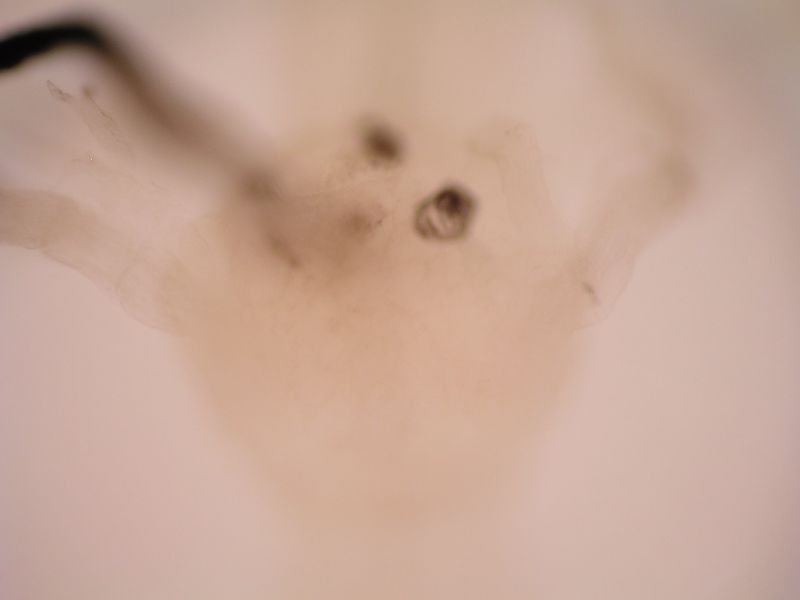

Supplement: Supplemental Information 2 — Micrographs of N2 backfills. Images have been reduced in size. [file peerj-03-1112-s002.zip › A3N2p 2009 07 13 (2).jpg]

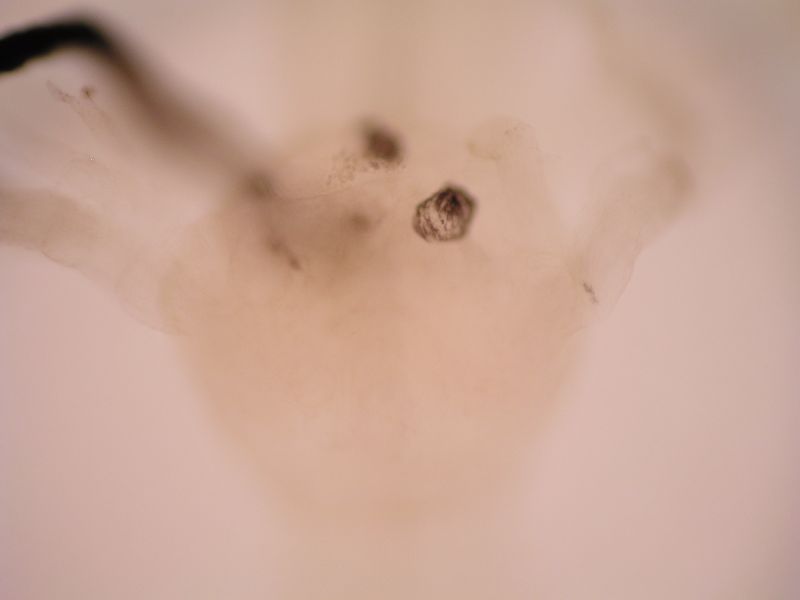

Supplement: Supplemental Information 2 — Micrographs of N2 backfills. Images have been reduced in size. [file peerj-03-1112-s002.zip › A3N2p 2009 07 13 (3).jpg]

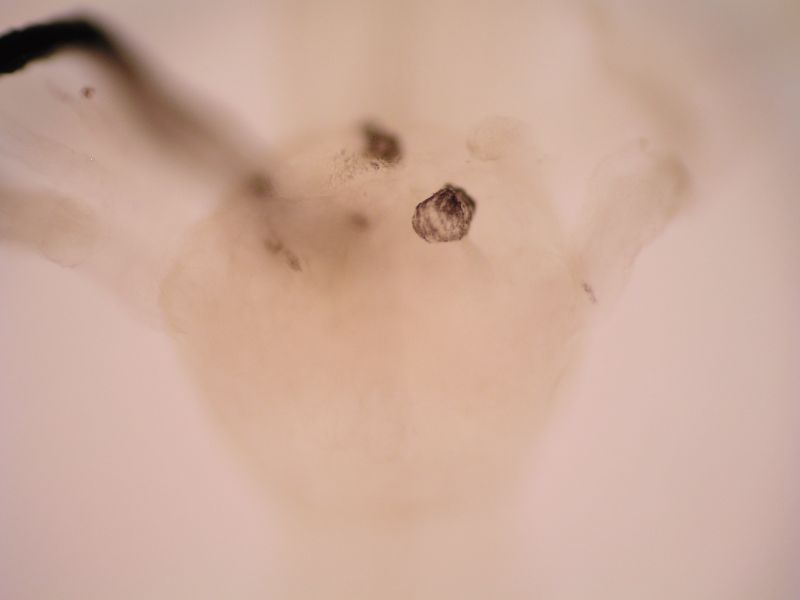

Supplement: Supplemental Information 2 — Micrographs of N2 backfills. Images have been reduced in size. [file peerj-03-1112-s002.zip › A3N2p 2009 07 13 (4).jpg]

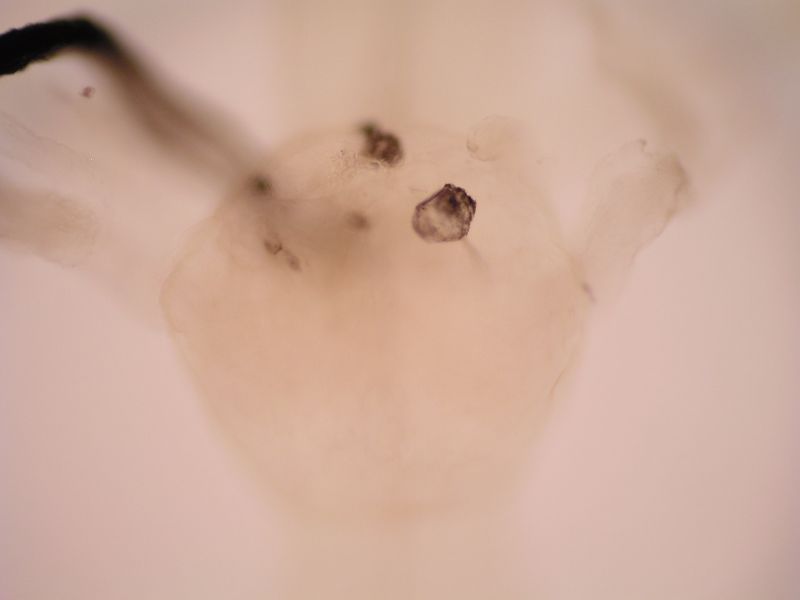

Supplement: Supplemental Information 2 — Micrographs of N2 backfills. Images have been reduced in size. [file peerj-03-1112-s002.zip › A3N2p 2009 07 13 (5).jpg]

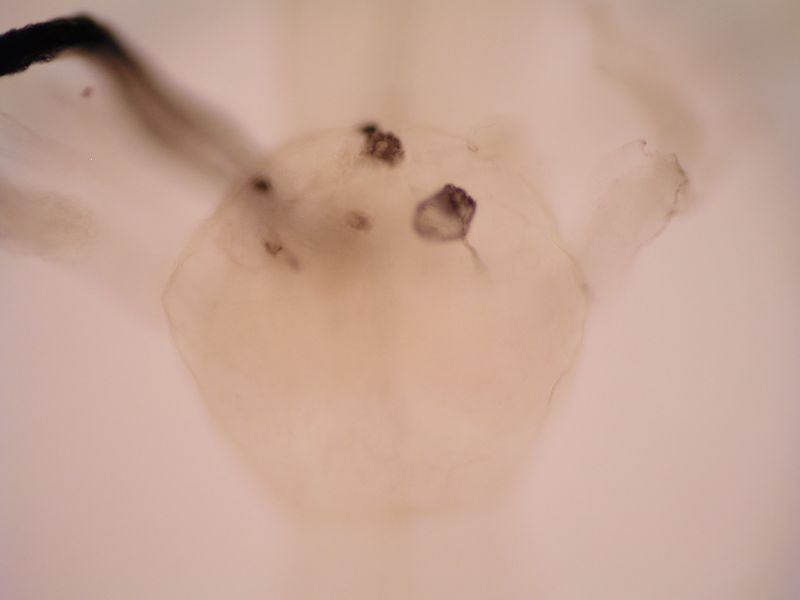

Supplement: Supplemental Information 2 — Micrographs of N2 backfills. Images have been reduced in size. [file peerj-03-1112-s002.zip › A3N2p 2009 07 13 (6).jpg]

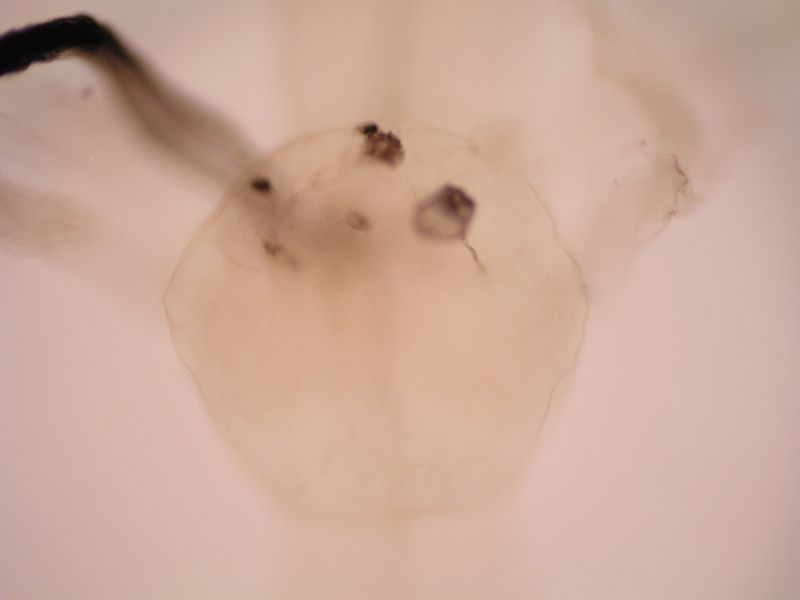

Supplement: Supplemental Information 2 — Micrographs of N2 backfills. Images have been reduced in size. [file peerj-03-1112-s002.zip › A3N2p 2009 07 13 (7).jpg]

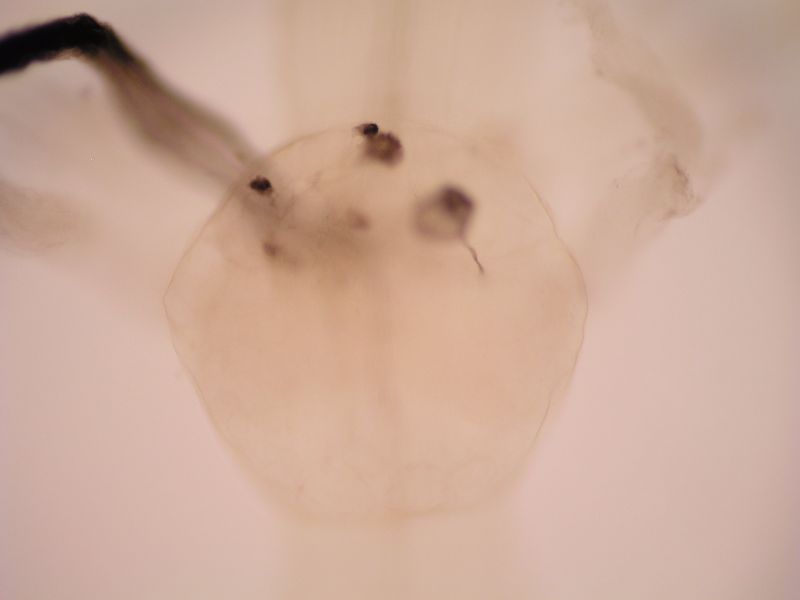

Supplement: Supplemental Information 2 — Micrographs of N2 backfills. Images have been reduced in size. [file peerj-03-1112-s002.zip › A3N2p 2009 07 13 (8).jpg]

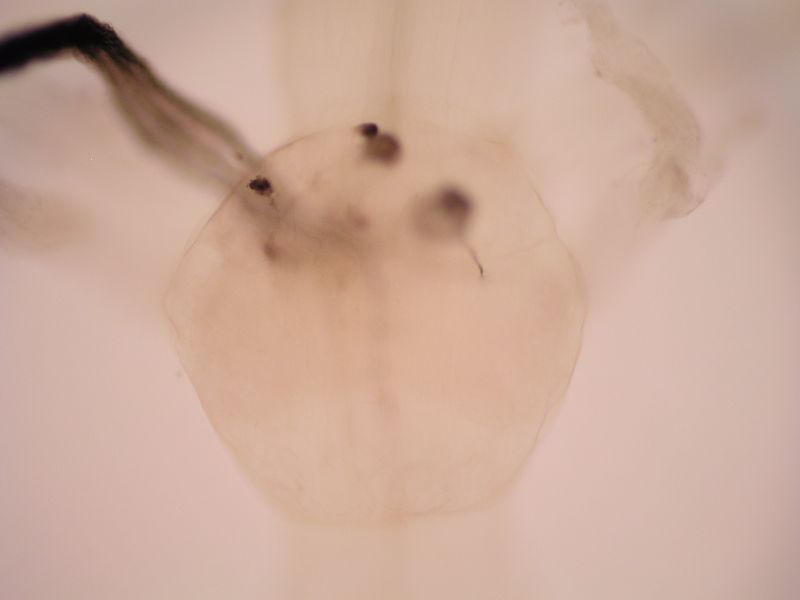

Supplement: Supplemental Information 2 — Micrographs of N2 backfills. Images have been reduced in size. [file peerj-03-1112-s002.zip › A3N2p 2009 07 13 (9).jpg]

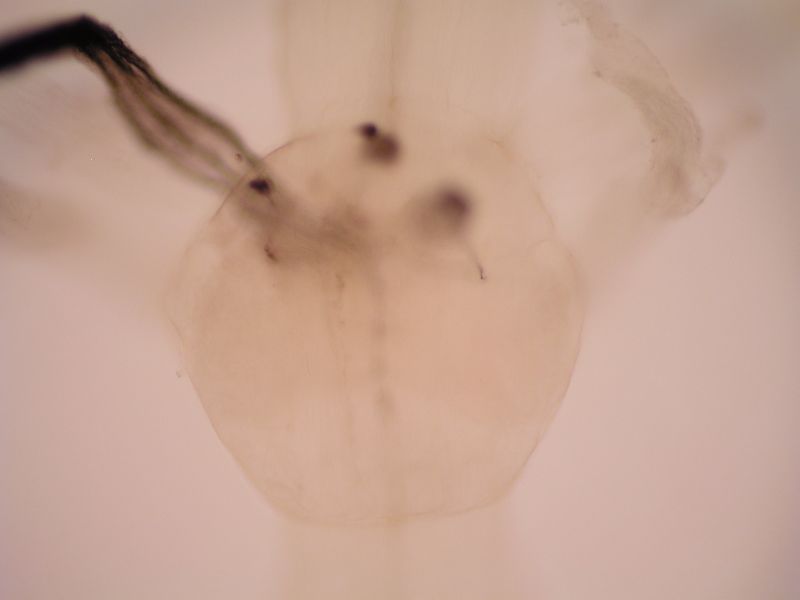

Supplement: Supplemental Information 2 — Micrographs of N2 backfills. Images have been reduced in size. [file peerj-03-1112-s002.zip › A3N2p 2009 07 13 (10).jpg]

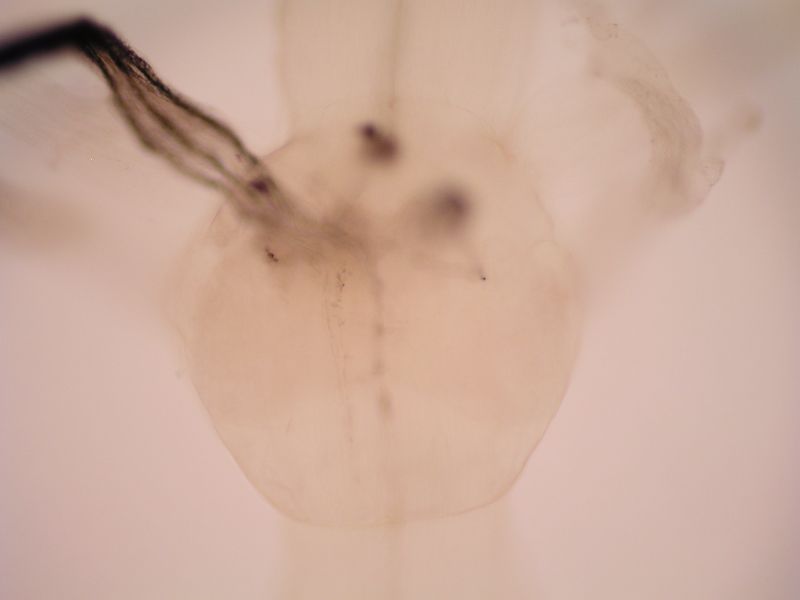

Supplement: Supplemental Information 2 — Micrographs of N2 backfills. Images have been reduced in size. [file peerj-03-1112-s002.zip › A3N2p 2009 07 13 (11).jpg]

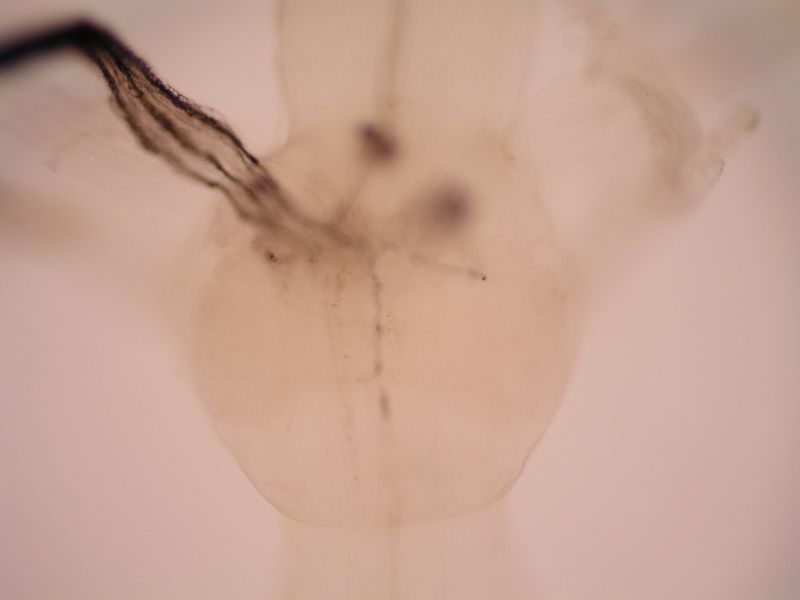

Supplement: Supplemental Information 2 — Micrographs of N2 backfills. Images have been reduced in size. [file peerj-03-1112-s002.zip › A3N2p 2009 07 13 (12).jpg]

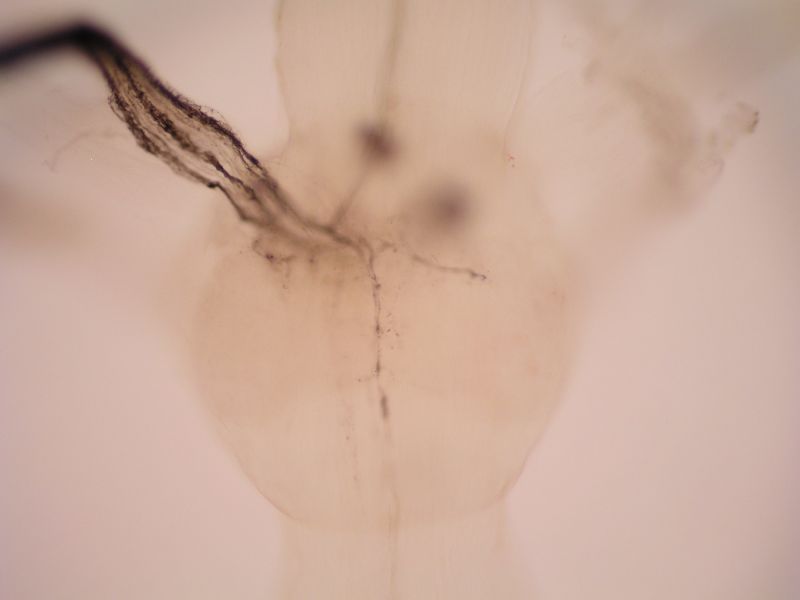

Supplement: Supplemental Information 2 — Micrographs of N2 backfills. Images have been reduced in size. [file peerj-03-1112-s002.zip › A3N2p 2009 07 13 (13).jpg]

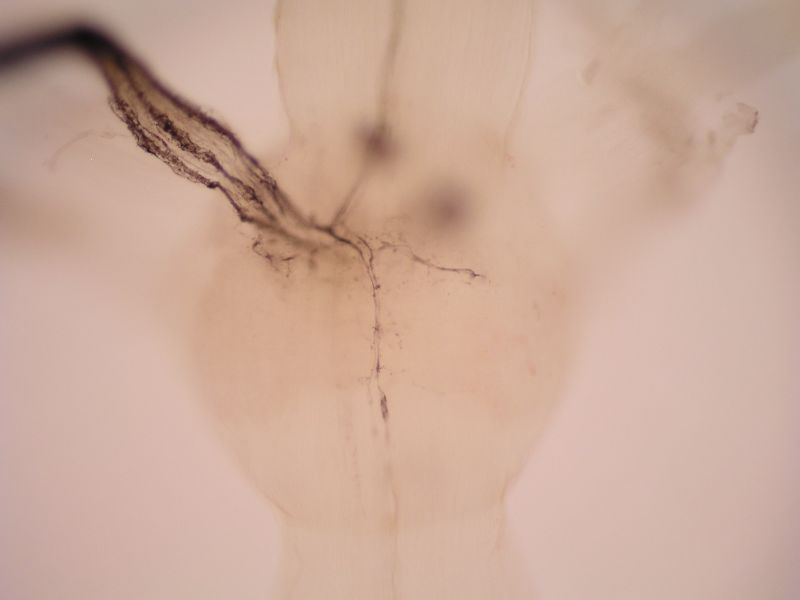

Supplement: Supplemental Information 2 — Micrographs of N2 backfills. Images have been reduced in size. [file peerj-03-1112-s002.zip › A3N2p 2009 07 13 (14).jpg]

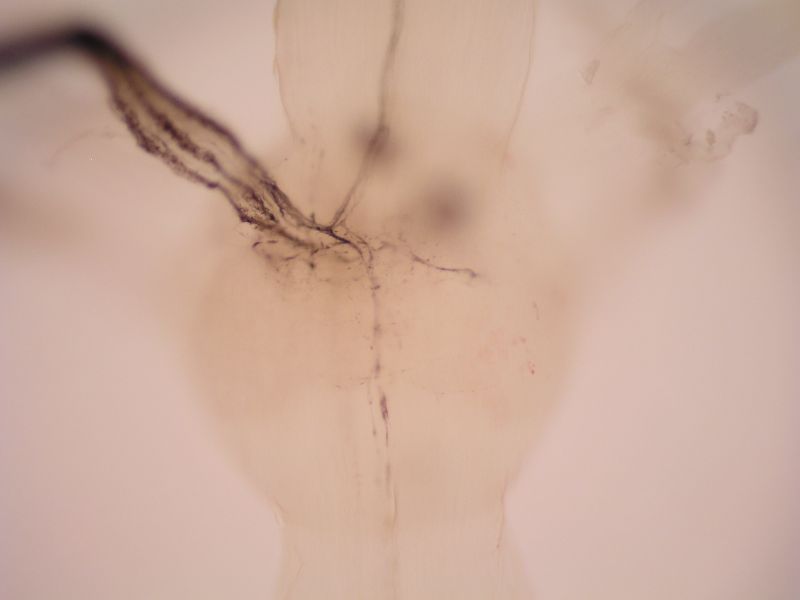

Supplement: Supplemental Information 2 — Micrographs of N2 backfills. Images have been reduced in size. [file peerj-03-1112-s002.zip › A3N2p 2009 07 13 (15).jpg]

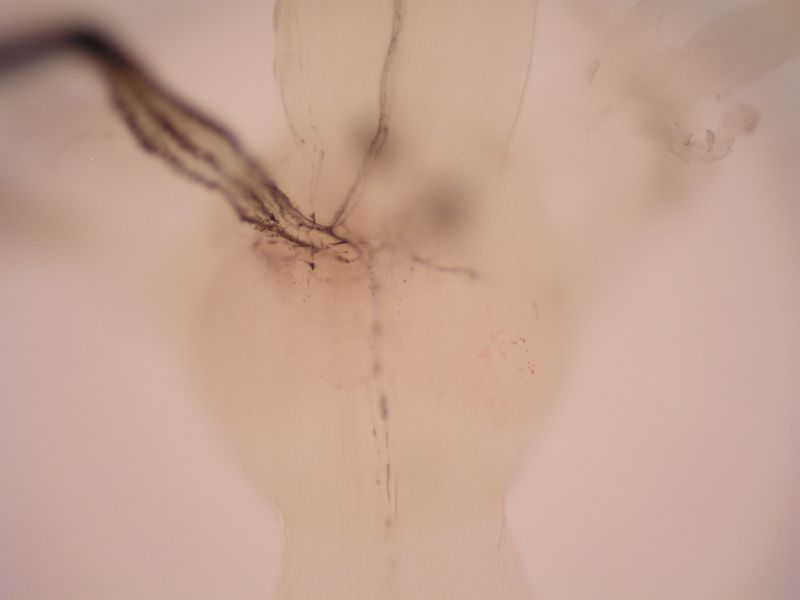

Supplement: Supplemental Information 2 — Micrographs of N2 backfills. Images have been reduced in size. [file peerj-03-1112-s002.zip › A3N2p 2009 07 13 (16).jpg]

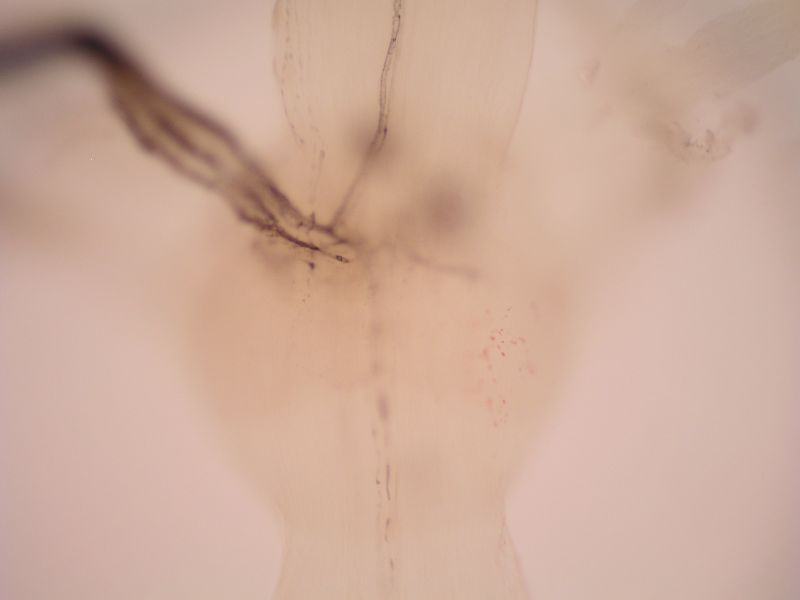

Supplement: Supplemental Information 2 — Micrographs of N2 backfills. Images have been reduced in size. [file peerj-03-1112-s002.zip › A3N2p 2009 07 13 (17).jpg]

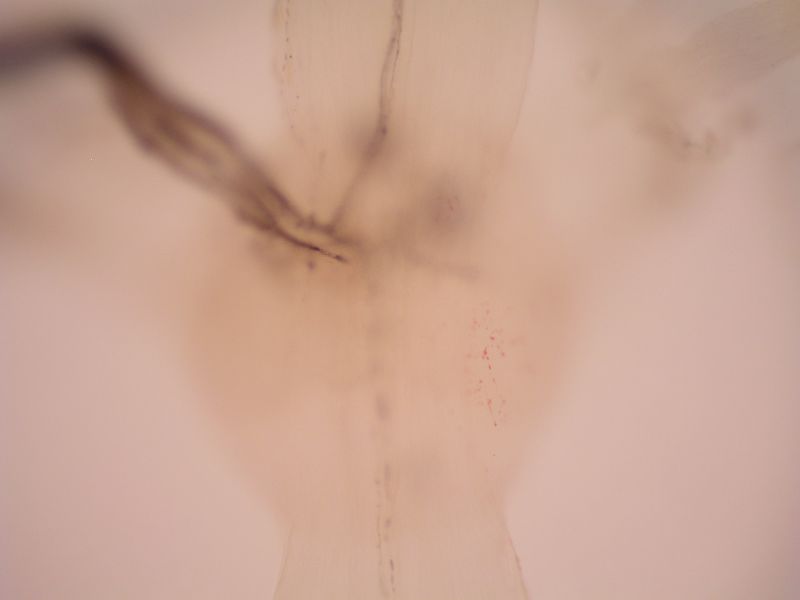

Supplement: Supplemental Information 2 — Micrographs of N2 backfills. Images have been reduced in size. [file peerj-03-1112-s002.zip › A3N2p 2009 07 13 (18).jpg]

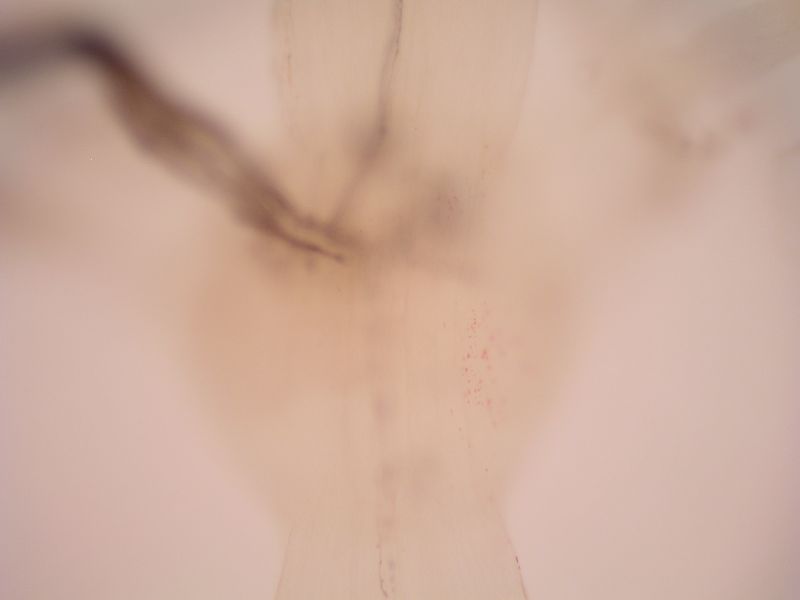

Supplement: Supplemental Information 2 — Micrographs of N2 backfills. Images have been reduced in size. [file peerj-03-1112-s002.zip › A3N2p 2009 07 13 (19).jpg]

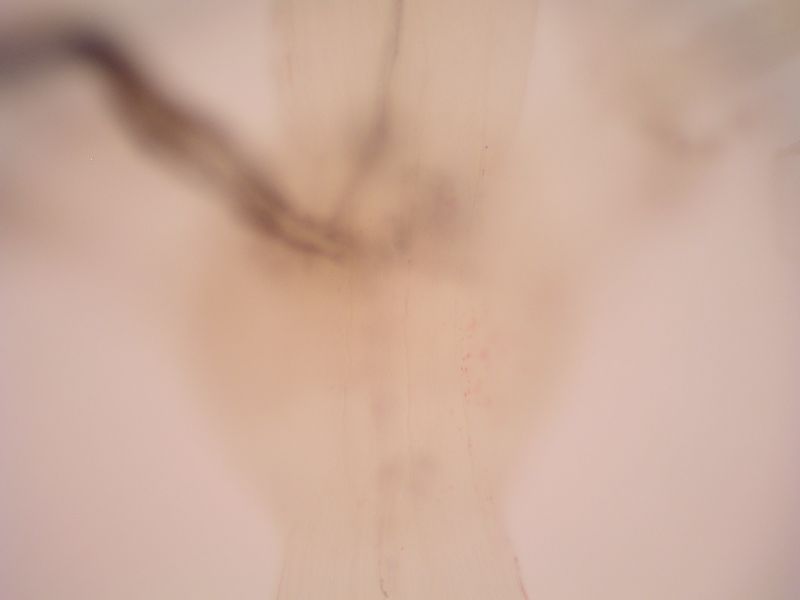

Supplement: Supplemental Information 2 — Micrographs of N2 backfills. Images have been reduced in size. [file peerj-03-1112-s002.zip › A3N2p 2009 07 13 (20).jpg]

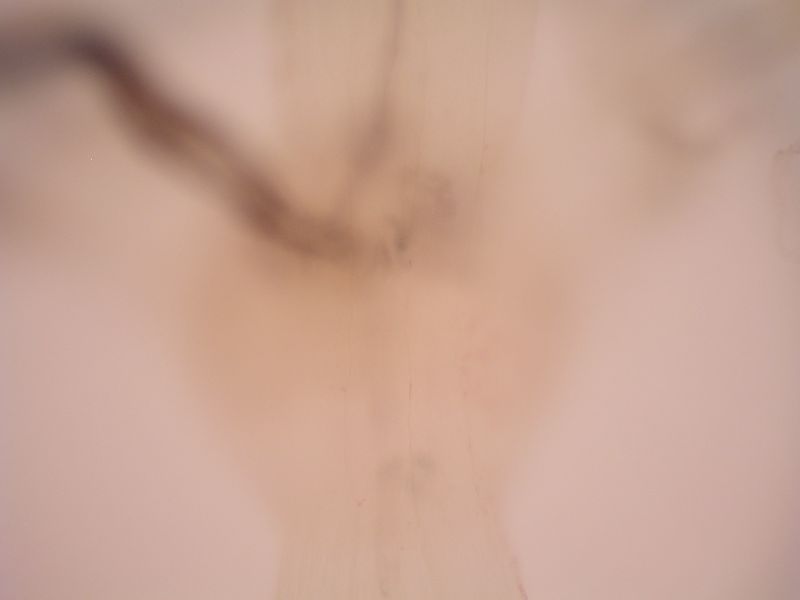

Supplement: Supplemental Information 2 — Micrographs of N2 backfills. Images have been reduced in size. [file peerj-03-1112-s002.zip › A3N2p 2009 07 13.jpg]

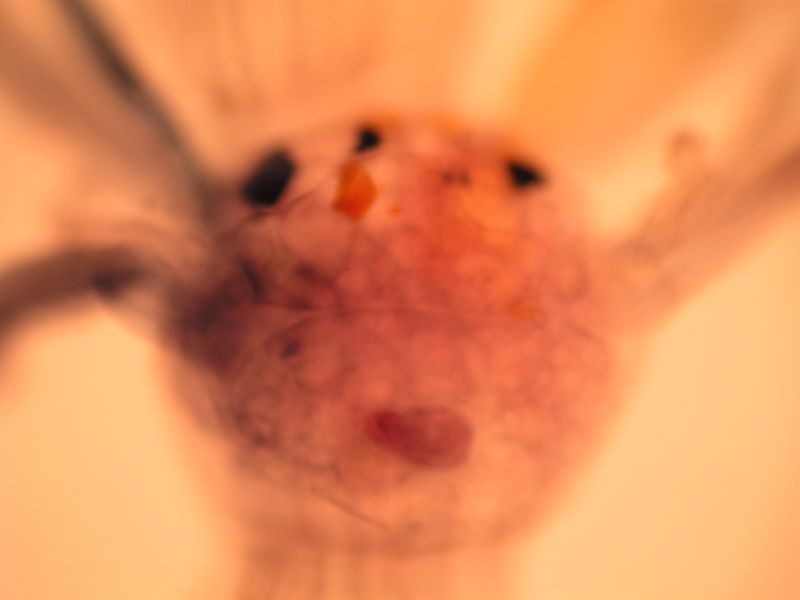

Supplement: Supplemental Information 2 — Micrographs of N2 backfills. Images have been reduced in size. [file peerj-03-1112-s002.zip › A4N2 2007 07 07 stack (1).jpg]

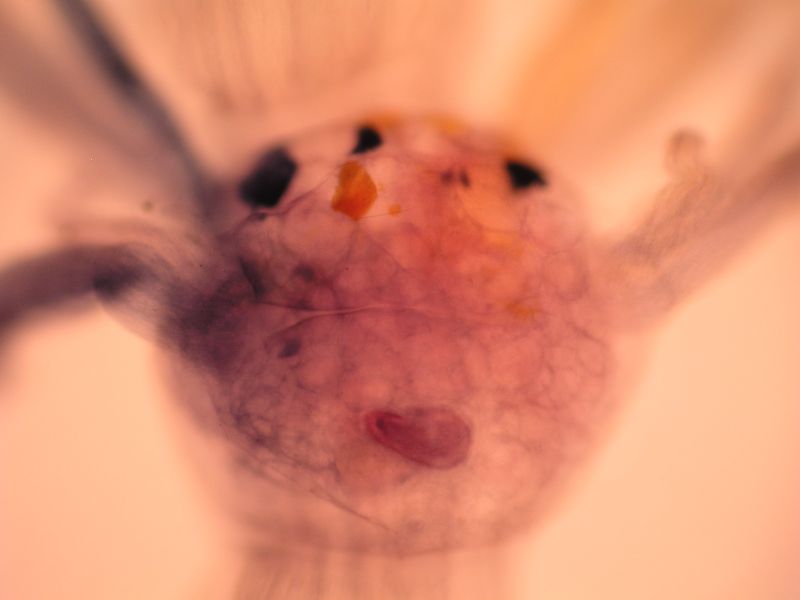

Supplement: Supplemental Information 2 — Micrographs of N2 backfills. Images have been reduced in size. [file peerj-03-1112-s002.zip › A4N2 2007 07 07 stack (2).jpg]

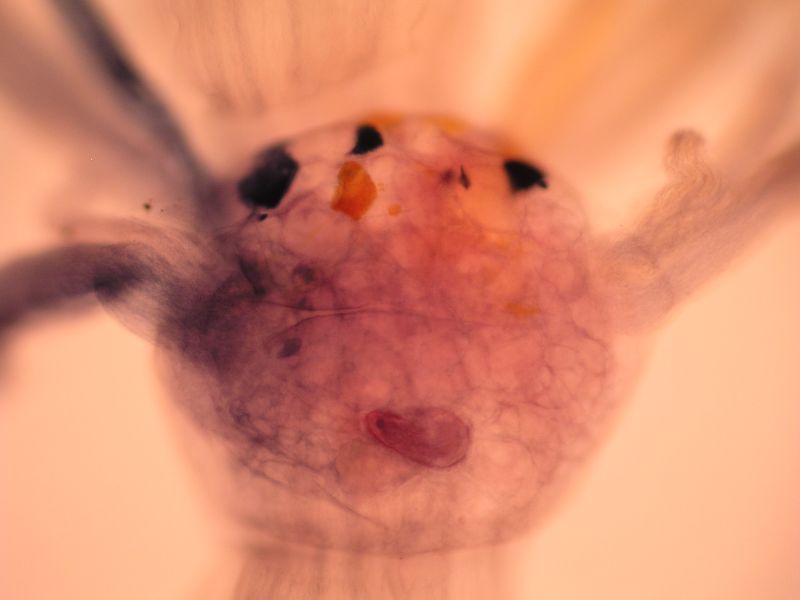

Supplement: Supplemental Information 2 — Micrographs of N2 backfills. Images have been reduced in size. [file peerj-03-1112-s002.zip › A4N2 2007 07 07 stack (3).jpg]

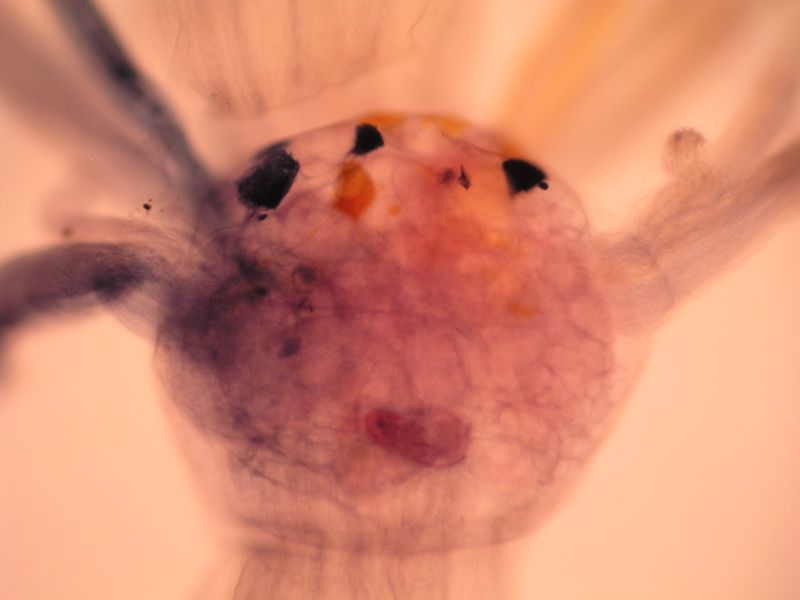

Supplement: Supplemental Information 2 — Micrographs of N2 backfills. Images have been reduced in size. [file peerj-03-1112-s002.zip › A4N2 2007 07 07 stack (4).jpg]

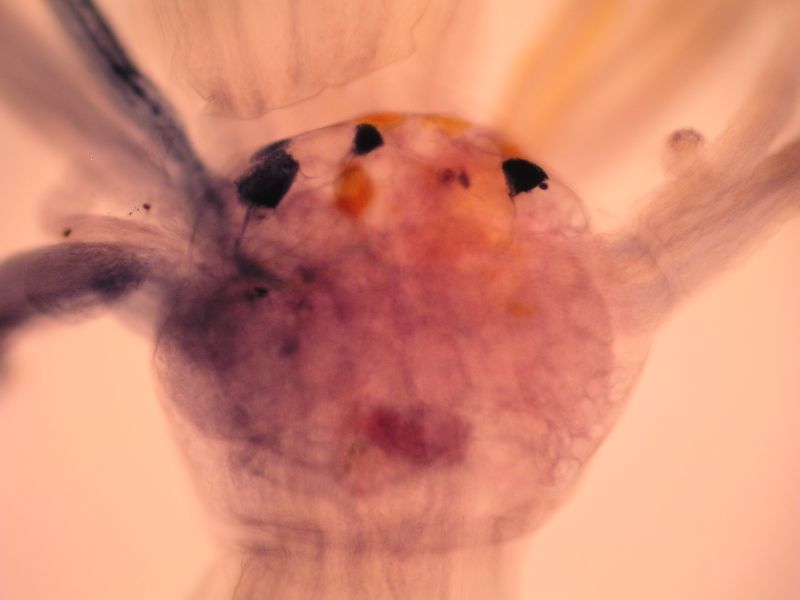

Supplement: Supplemental Information 2 — Micrographs of N2 backfills. Images have been reduced in size. [file peerj-03-1112-s002.zip › A4N2 2007 07 07 stack (5).jpg]

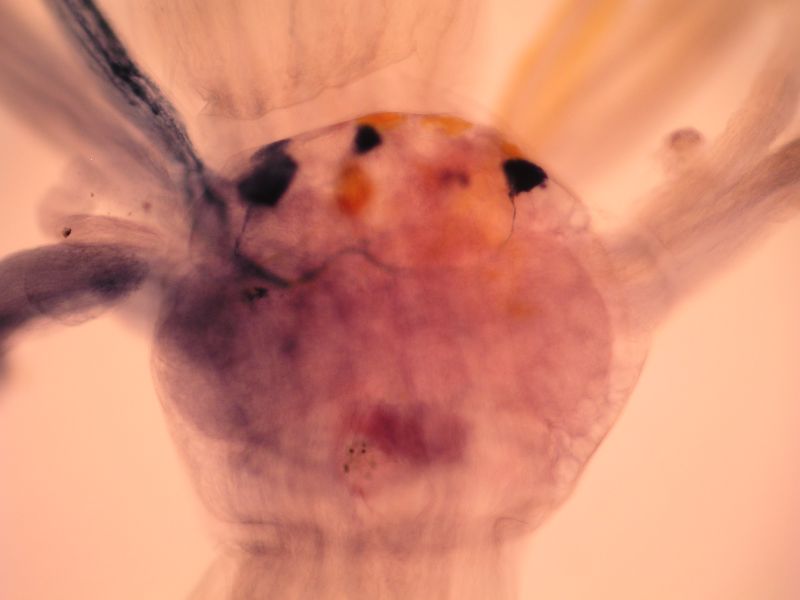

Supplement: Supplemental Information 2 — Micrographs of N2 backfills. Images have been reduced in size. [file peerj-03-1112-s002.zip › A4N2 2007 07 07 stack (6).jpg]

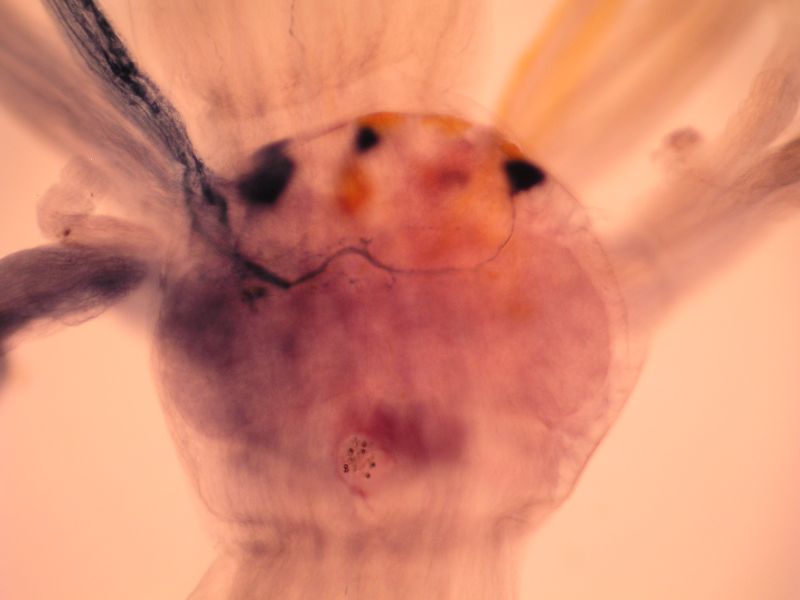

Supplement: Supplemental Information 2 — Micrographs of N2 backfills. Images have been reduced in size. [file peerj-03-1112-s002.zip › A4N2 2007 07 07 stack (7).jpg]

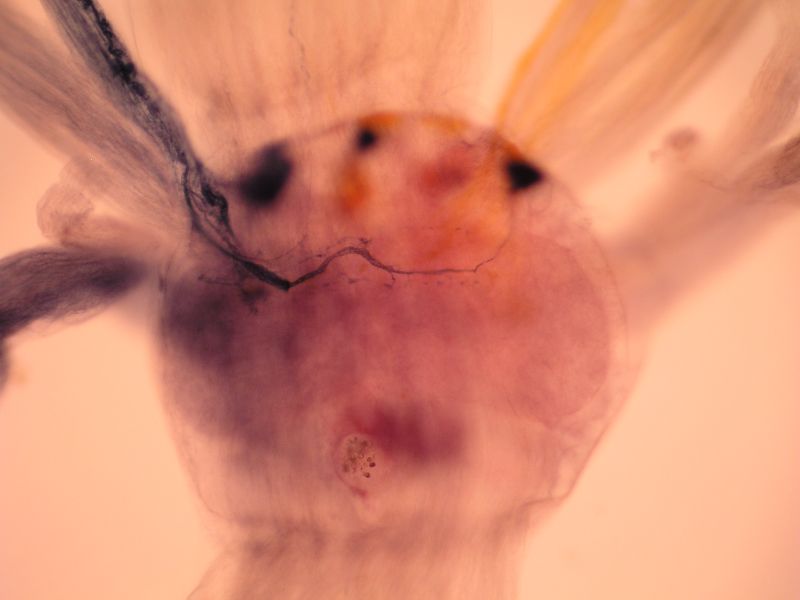

Supplement: Supplemental Information 2 — Micrographs of N2 backfills. Images have been reduced in size. [file peerj-03-1112-s002.zip › A4N2 2007 07 07 stack (8).jpg]

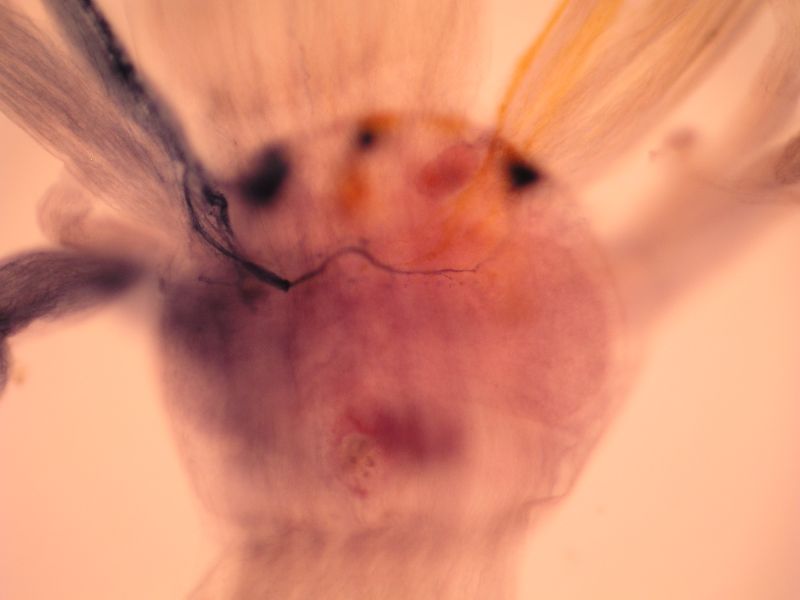

Supplement: Supplemental Information 2 — Micrographs of N2 backfills. Images have been reduced in size. [file peerj-03-1112-s002.zip › A4N2 2007 07 07 stack (9).jpg]

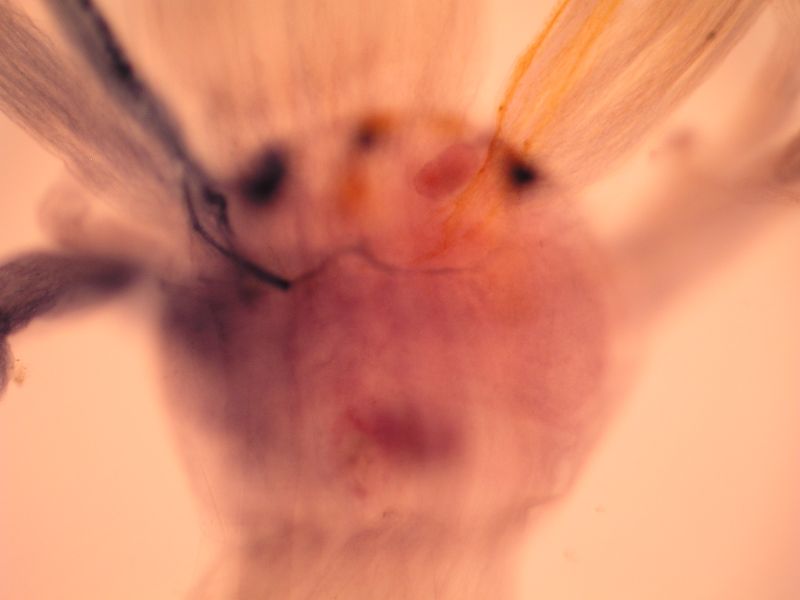

Supplement: Supplemental Information 2 — Micrographs of N2 backfills. Images have been reduced in size. [file peerj-03-1112-s002.zip › A4N2 2007 07 07 stack (10).jpg]

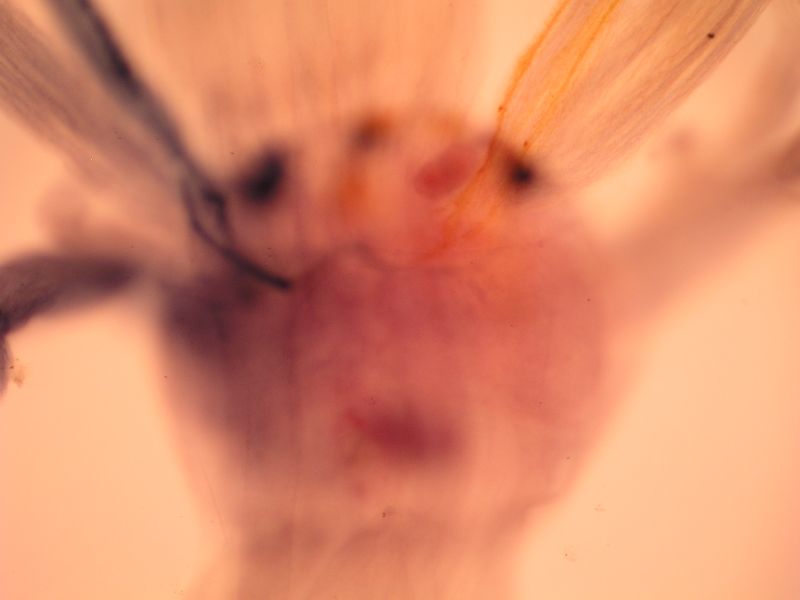

Supplement: Supplemental Information 2 — Micrographs of N2 backfills. Images have been reduced in size. [file peerj-03-1112-s002.zip › A4N2 2007 07 07 stack (11).jpg]

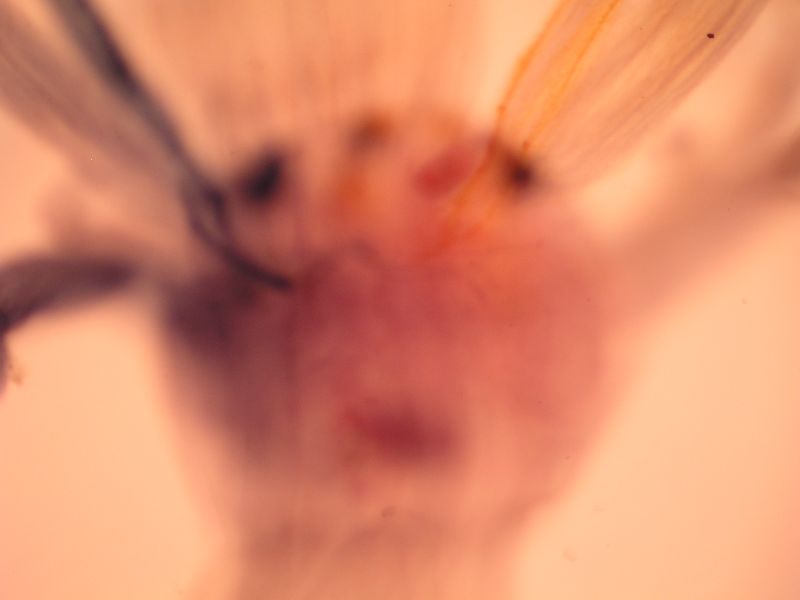

Supplement: Supplemental Information 2 — Micrographs of N2 backfills. Images have been reduced in size. [file peerj-03-1112-s002.zip › A4N2 2007 07 07 stack (12).jpg]

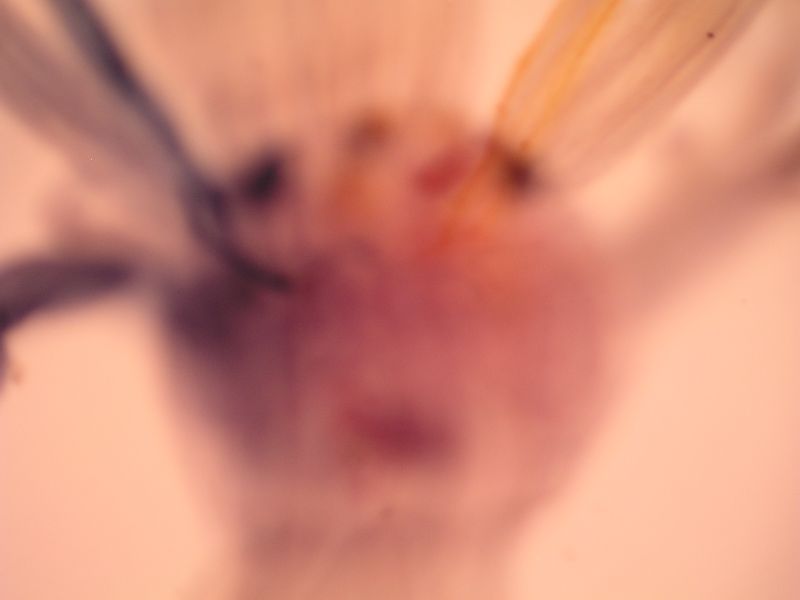

Supplement: Supplemental Information 2 — Micrographs of N2 backfills. Images have been reduced in size. [file peerj-03-1112-s002.zip › A4N2 2007 07 07 stack (13).jpg]

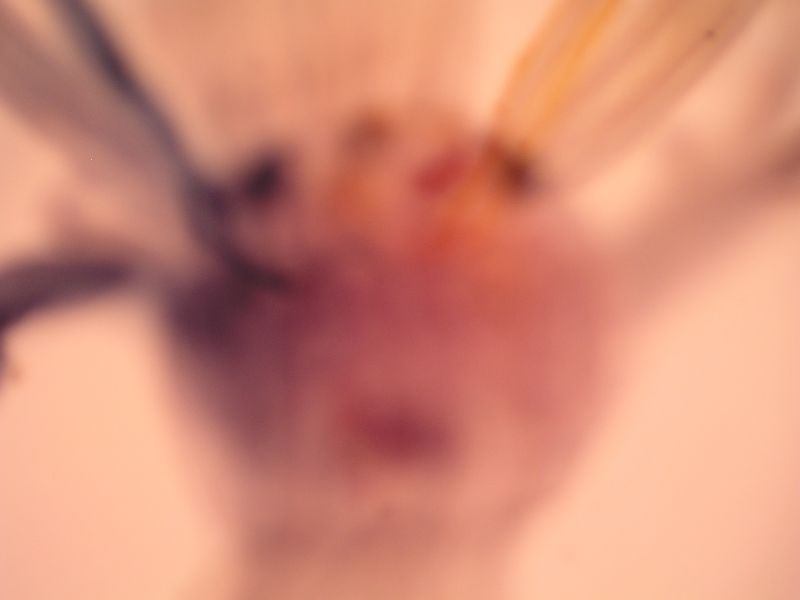

Supplement: Supplemental Information 2 — Micrographs of N2 backfills. Images have been reduced in size. [file peerj-03-1112-s002.zip › A4N2 2007 07 07 stack (14).jpg]

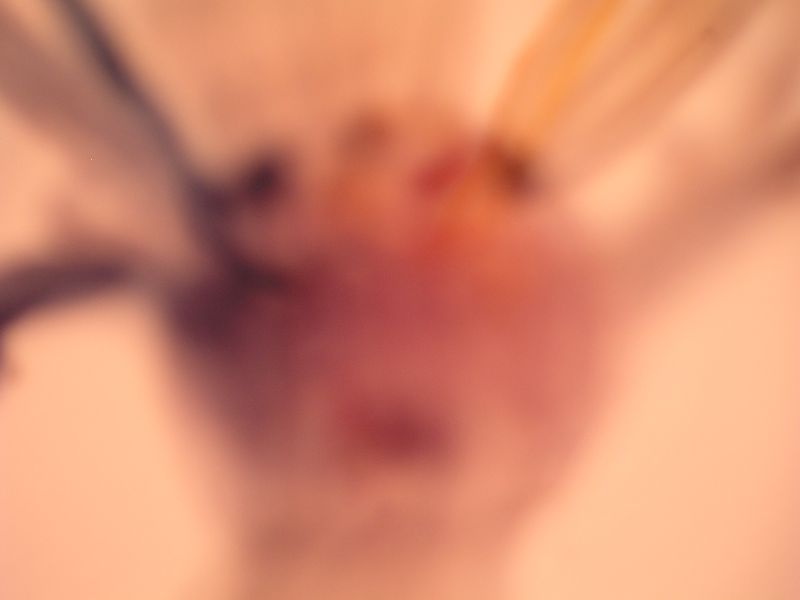

Supplement: Supplemental Information 2 — Micrographs of N2 backfills. Images have been reduced in size. [file peerj-03-1112-s002.zip › A4N2 2007 07 07 stack.jpg]

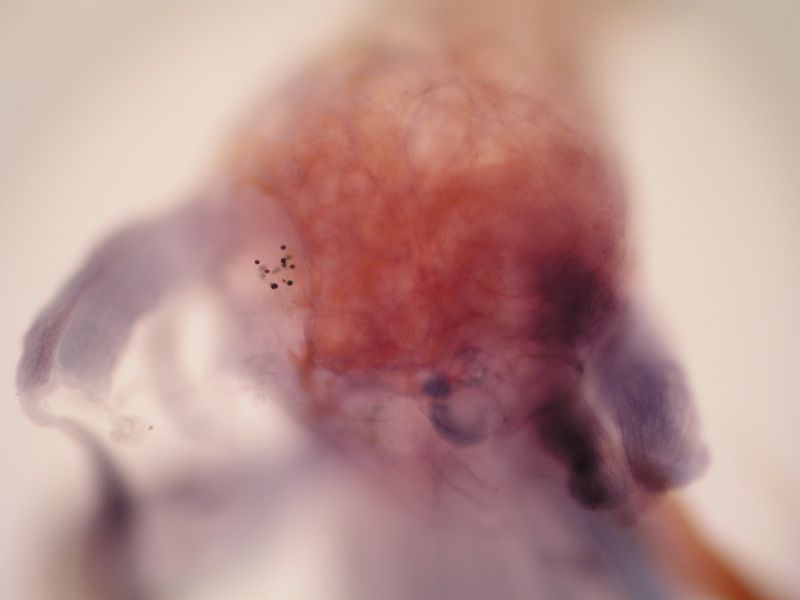

Supplement: Supplemental Information 2 — Micrographs of N2 backfills. Images have been reduced in size. [file peerj-03-1112-s002.zip › A4N2 2009 06 21 a.jpg]

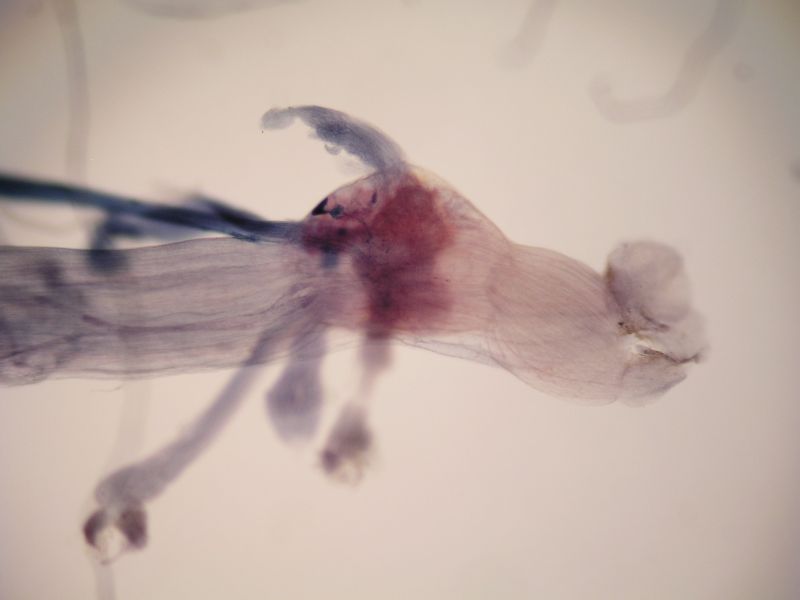

Supplement: Supplemental Information 2 — Micrographs of N2 backfills. Images have been reduced in size. [file peerj-03-1112-s002.zip › A4N2p 2009 06 18 a.jpg]

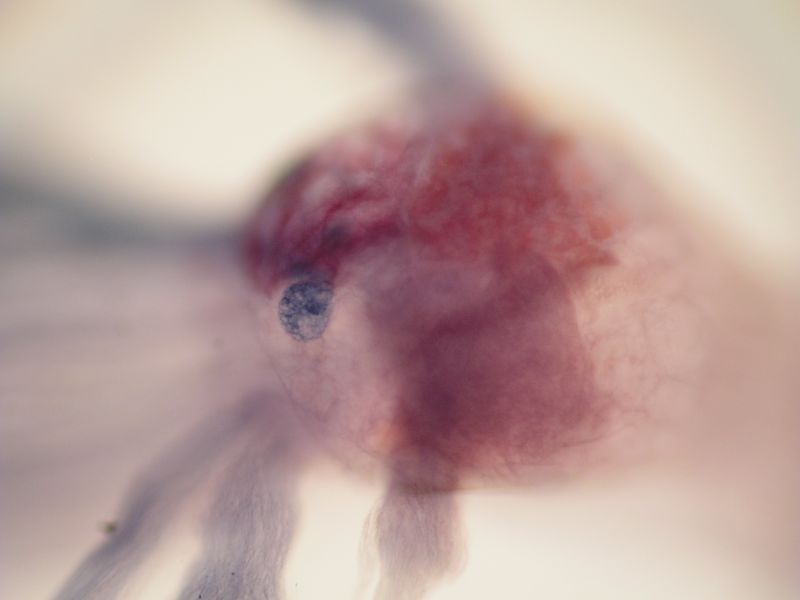

Supplement: Supplemental Information 2 — Micrographs of N2 backfills. Images have been reduced in size. [file peerj-03-1112-s002.zip › A4N2p 2009 06 18 b.jpg]

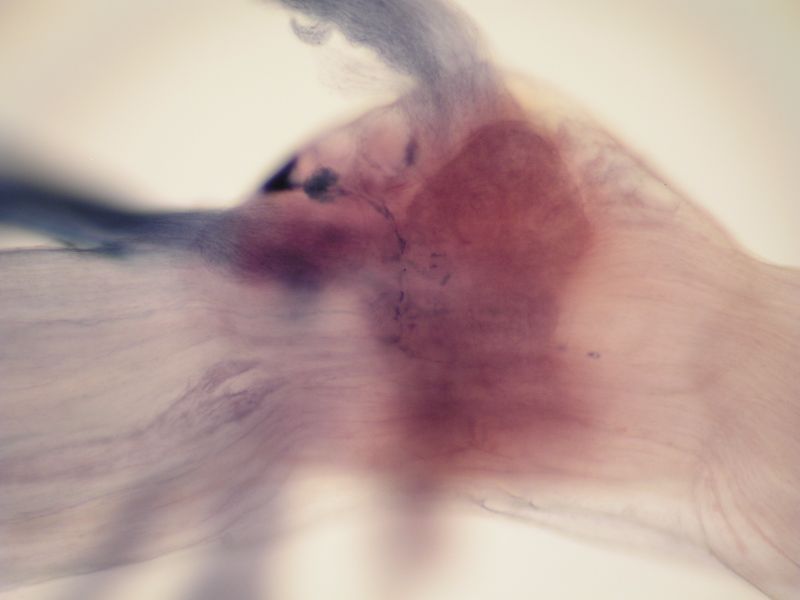

Supplement: Supplemental Information 2 — Micrographs of N2 backfills. Images have been reduced in size. [file peerj-03-1112-s002.zip › A4N2p 2009 06 18 c.jpg]

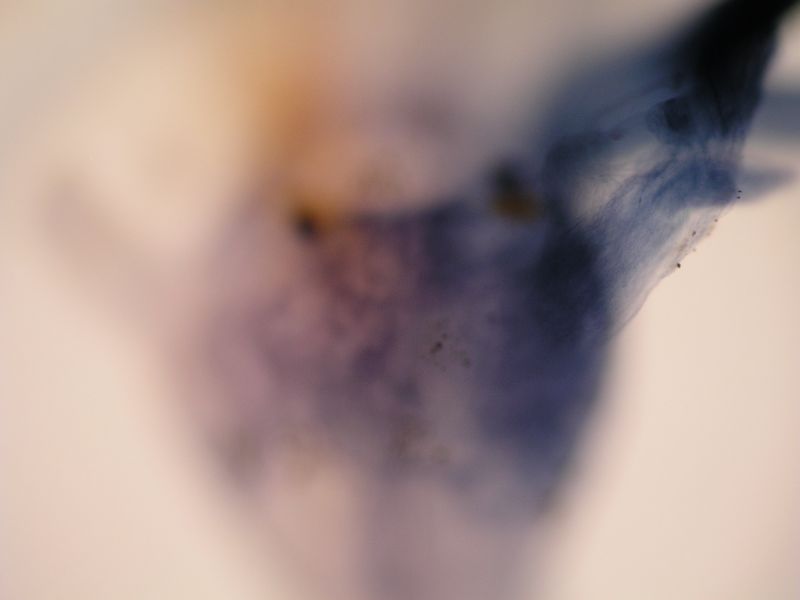

Supplement: Supplemental Information 2 — Micrographs of N2 backfills. Images have been reduced in size. [file peerj-03-1112-s002.zip › A4N2p 2009 0708 (1).jpg]

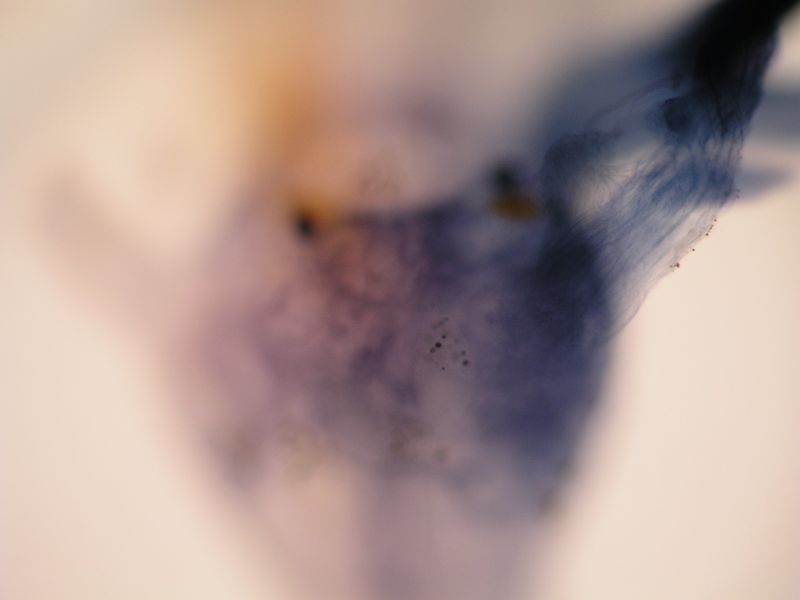

Supplement: Supplemental Information 2 — Micrographs of N2 backfills. Images have been reduced in size. [file peerj-03-1112-s002.zip › A4N2p 2009 0708 (2).jpg]

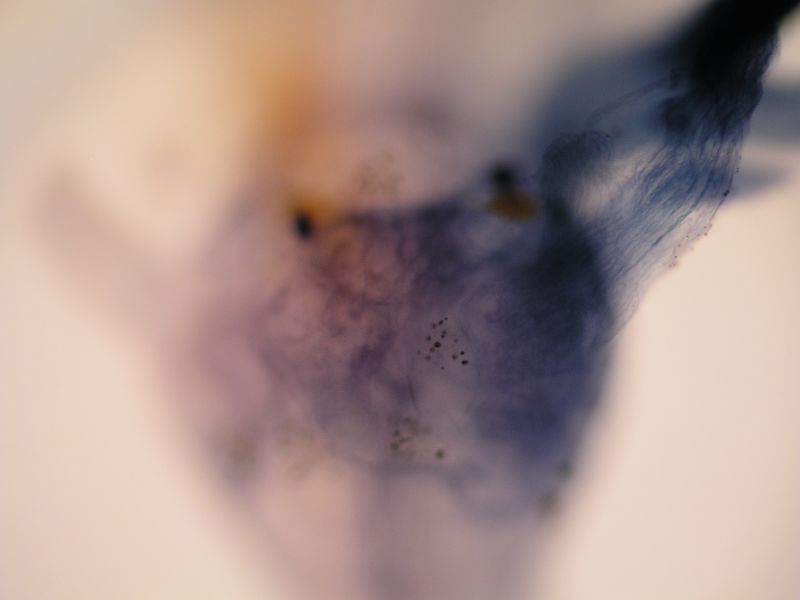

Supplement: Supplemental Information 2 — Micrographs of N2 backfills. Images have been reduced in size. [file peerj-03-1112-s002.zip › A4N2p 2009 0708 (3).jpg]

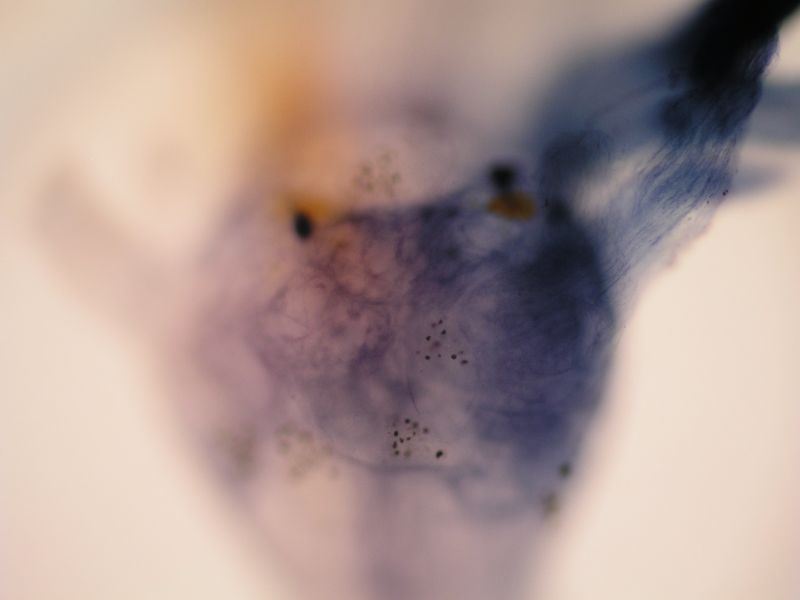

Supplement: Supplemental Information 2 — Micrographs of N2 backfills. Images have been reduced in size. [file peerj-03-1112-s002.zip › A4N2p 2009 0708 (4).jpg]

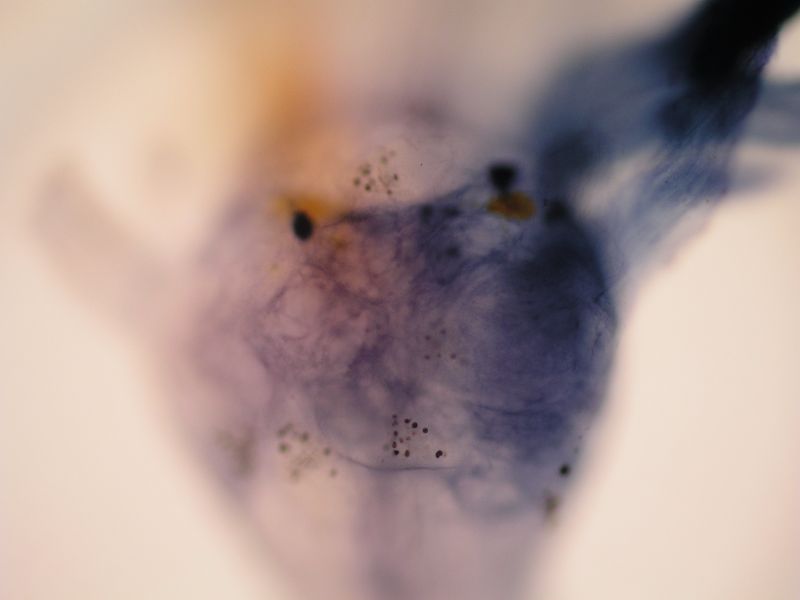

Supplement: Supplemental Information 2 — Micrographs of N2 backfills. Images have been reduced in size. [file peerj-03-1112-s002.zip › A4N2p 2009 0708 (5).jpg]

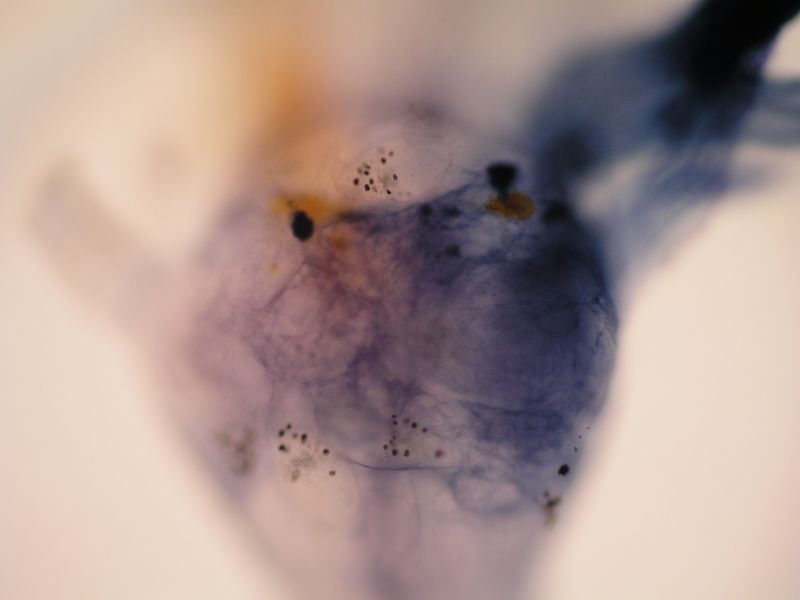

Supplement: Supplemental Information 2 — Micrographs of N2 backfills. Images have been reduced in size. [file peerj-03-1112-s002.zip › A4N2p 2009 0708 (6).jpg]

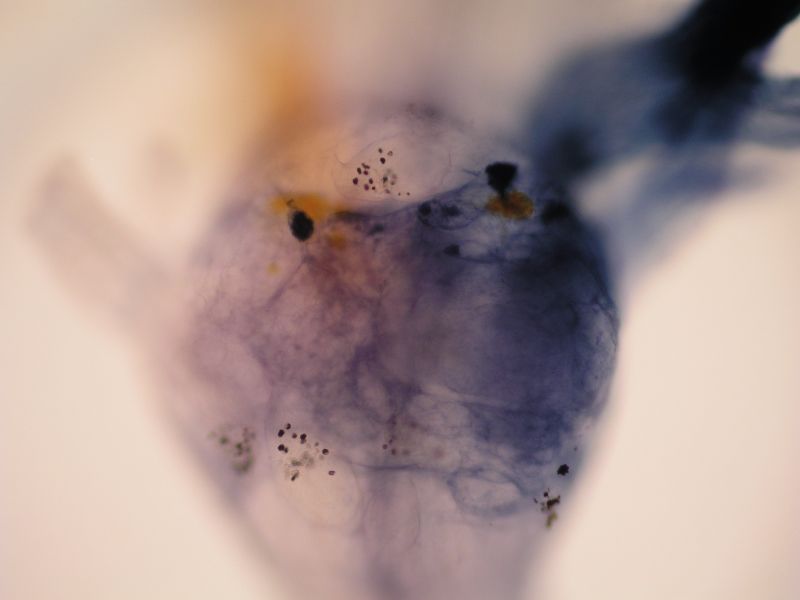

Supplement: Supplemental Information 2 — Micrographs of N2 backfills. Images have been reduced in size. [file peerj-03-1112-s002.zip › A4N2p 2009 0708 (7).jpg]

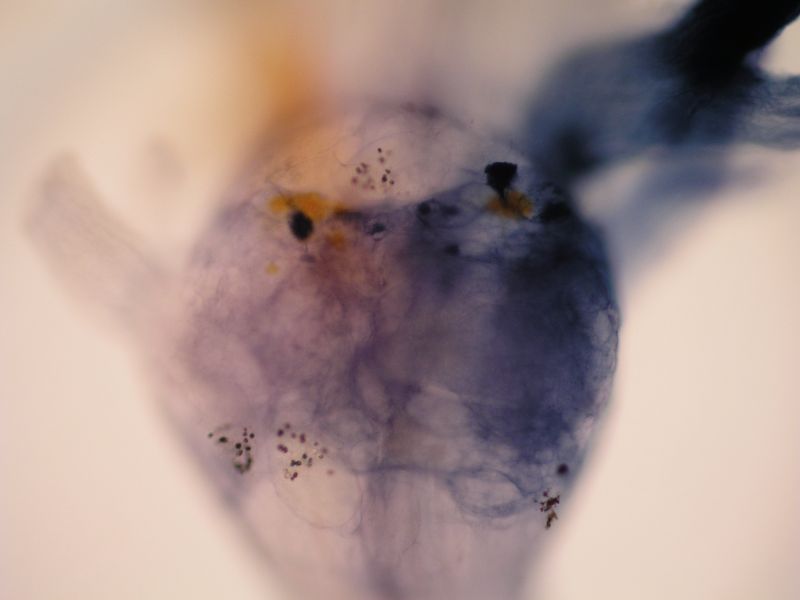

Supplement: Supplemental Information 2 — Micrographs of N2 backfills. Images have been reduced in size. [file peerj-03-1112-s002.zip › A4N2p 2009 0708 (8).jpg]

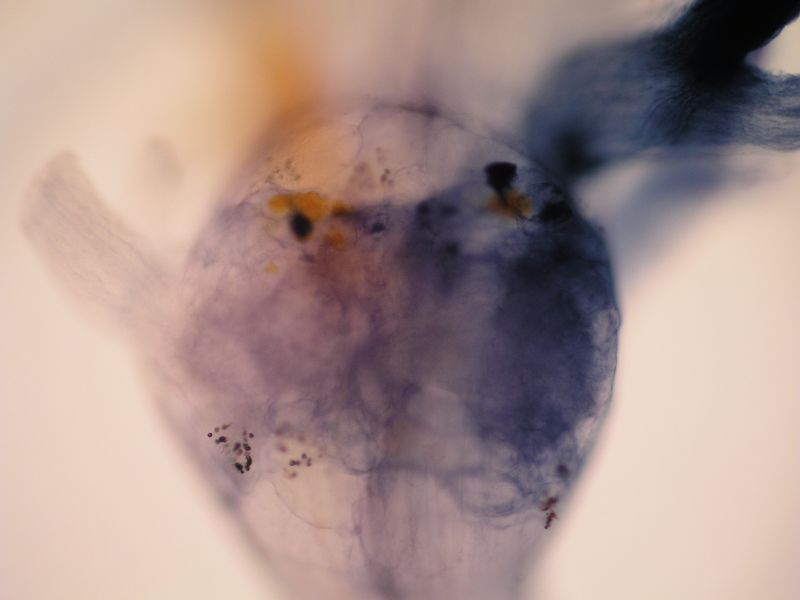

Supplement: Supplemental Information 2 — Micrographs of N2 backfills. Images have been reduced in size. [file peerj-03-1112-s002.zip › A4N2p 2009 0708 (9).jpg]

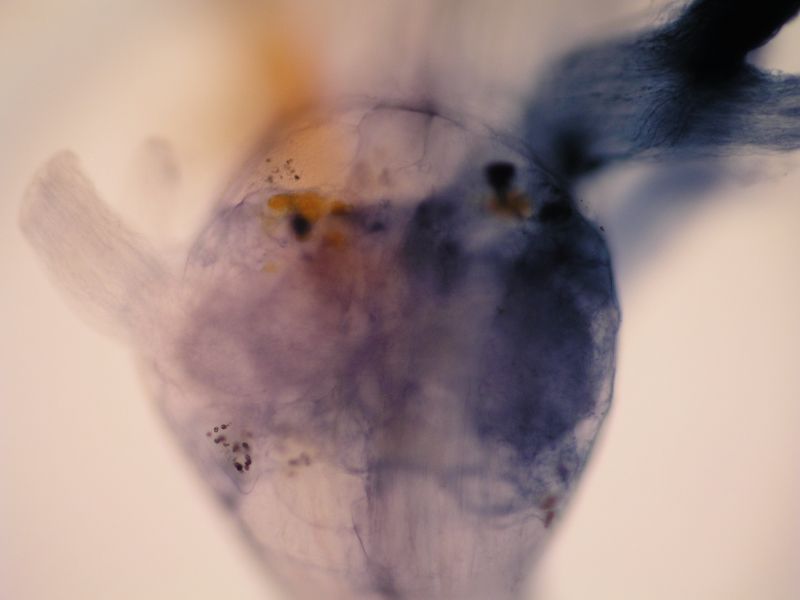

Supplement: Supplemental Information 2 — Micrographs of N2 backfills. Images have been reduced in size. [file peerj-03-1112-s002.zip › A4N2p 2009 0708 (10).jpg]

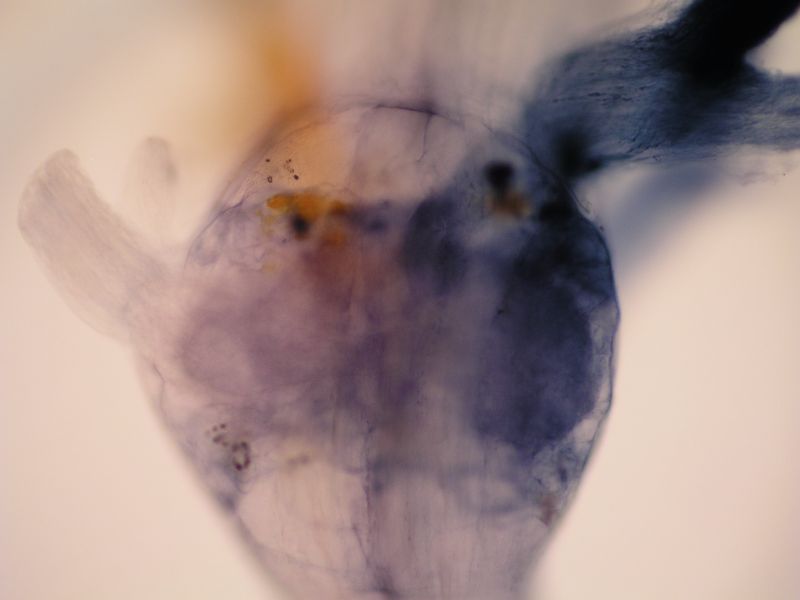

Supplement: Supplemental Information 2 — Micrographs of N2 backfills. Images have been reduced in size. [file peerj-03-1112-s002.zip › A4N2p 2009 0708 (11).jpg]

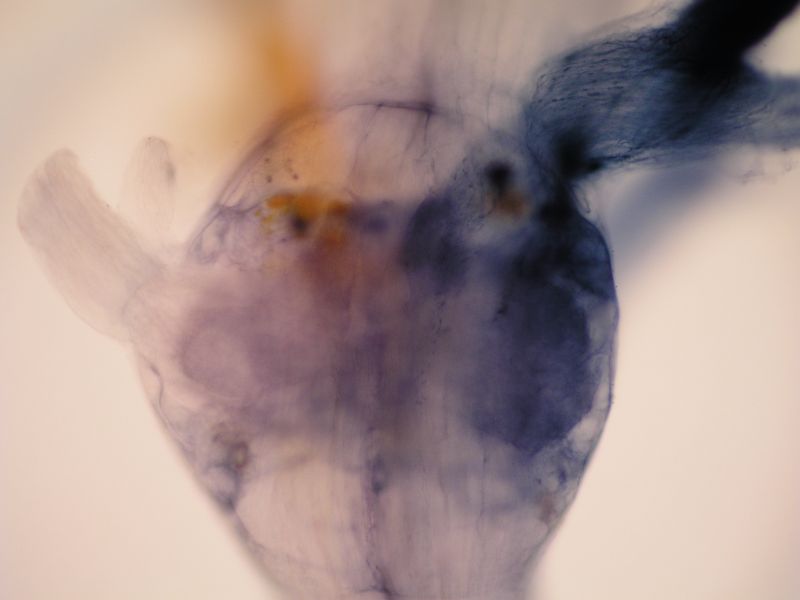

Supplement: Supplemental Information 2 — Micrographs of N2 backfills. Images have been reduced in size. [file peerj-03-1112-s002.zip › A4N2p 2009 0708 (12).jpg]

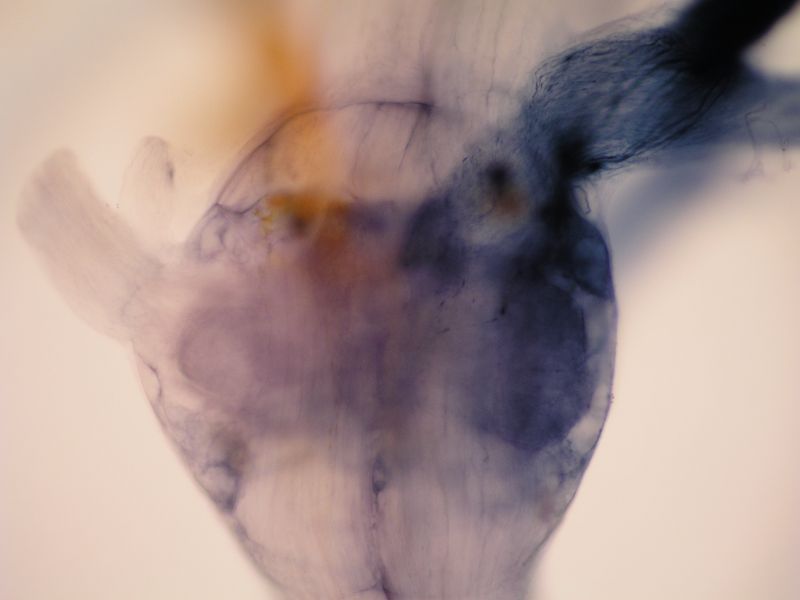

Supplement: Supplemental Information 2 — Micrographs of N2 backfills. Images have been reduced in size. [file peerj-03-1112-s002.zip › A4N2p 2009 0708 (13).jpg]

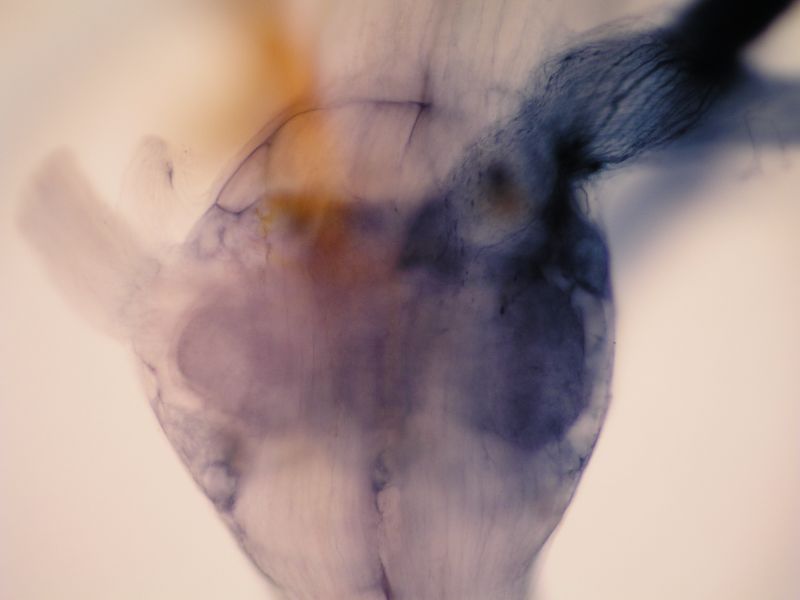

Supplement: Supplemental Information 2 — Micrographs of N2 backfills. Images have been reduced in size. [file peerj-03-1112-s002.zip › A4N2p 2009 0708 (14).jpg]

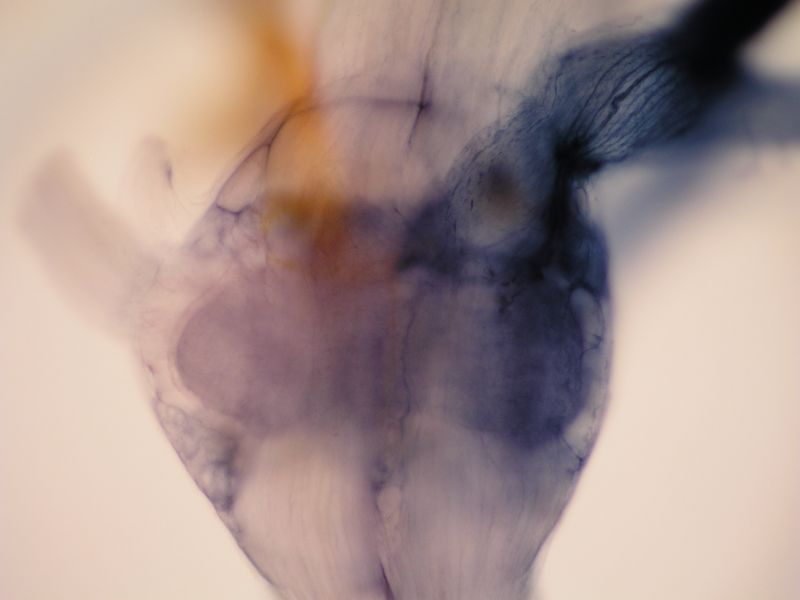

Supplement: Supplemental Information 2 — Micrographs of N2 backfills. Images have been reduced in size. [file peerj-03-1112-s002.zip › A4N2p 2009 0708 (15).jpg]

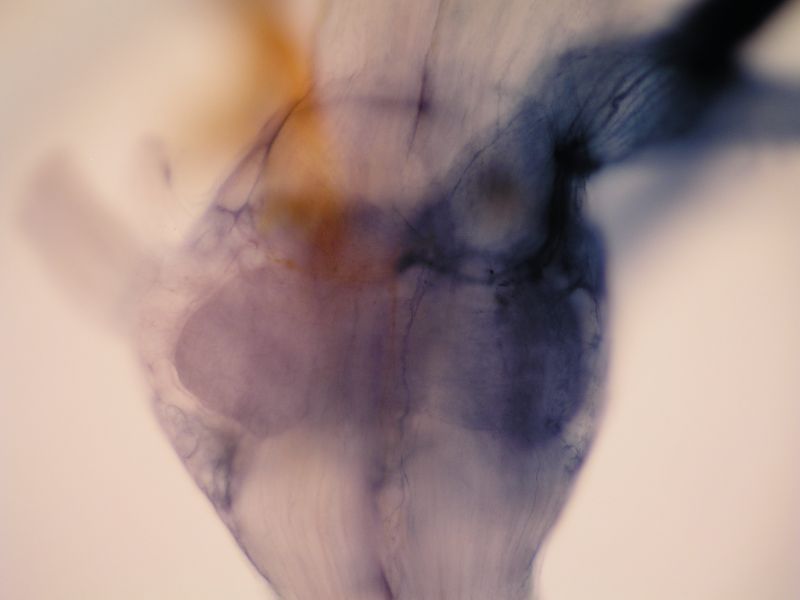

Supplement: Supplemental Information 2 — Micrographs of N2 backfills. Images have been reduced in size. [file peerj-03-1112-s002.zip › A4N2p 2009 0708 (16).jpg]

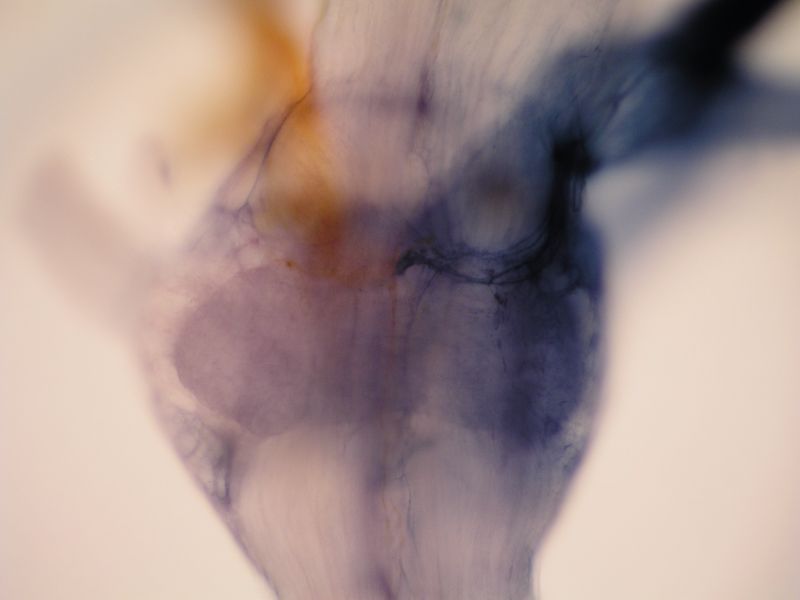

Supplement: Supplemental Information 2 — Micrographs of N2 backfills. Images have been reduced in size. [file peerj-03-1112-s002.zip › A4N2p 2009 0708 (17).jpg]

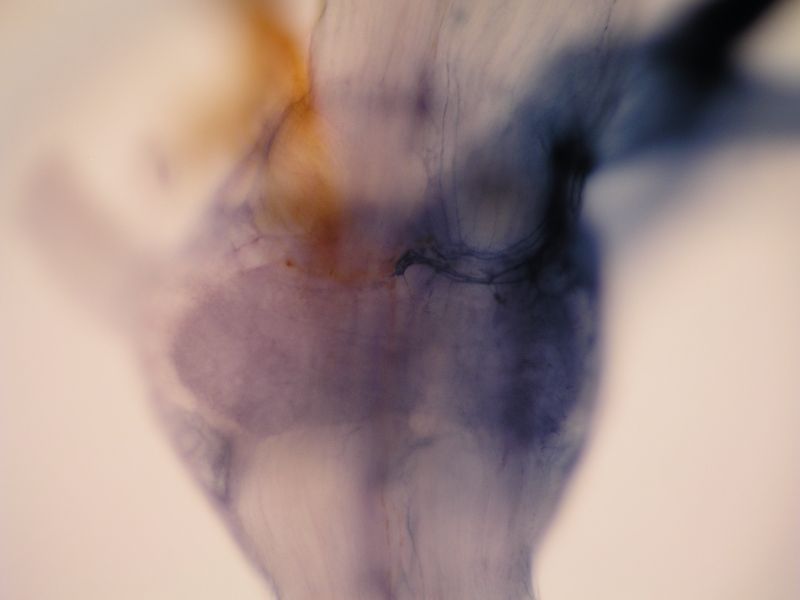

Supplement: Supplemental Information 2 — Micrographs of N2 backfills. Images have been reduced in size. [file peerj-03-1112-s002.zip › A4N2p 2009 0708 (18).jpg]

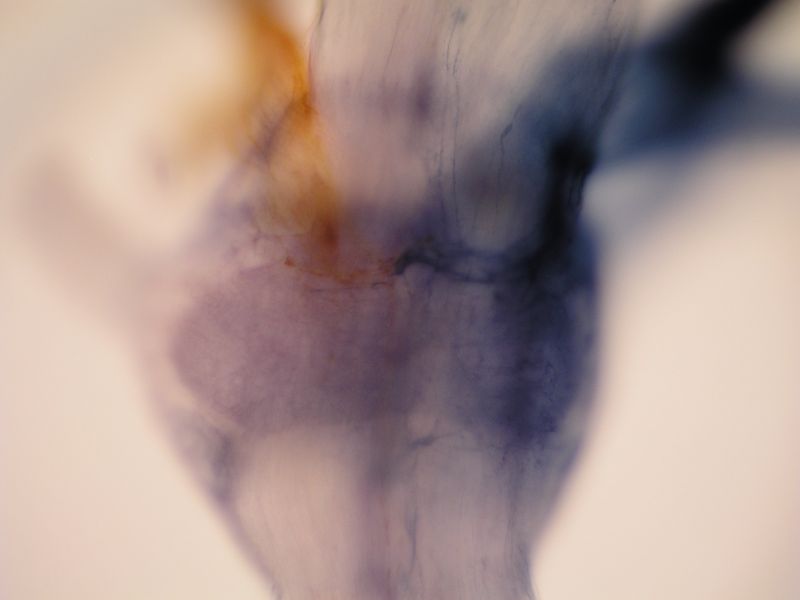

Supplement: Supplemental Information 2 — Micrographs of N2 backfills. Images have been reduced in size. [file peerj-03-1112-s002.zip › A4N2p 2009 0708 (19).jpg]

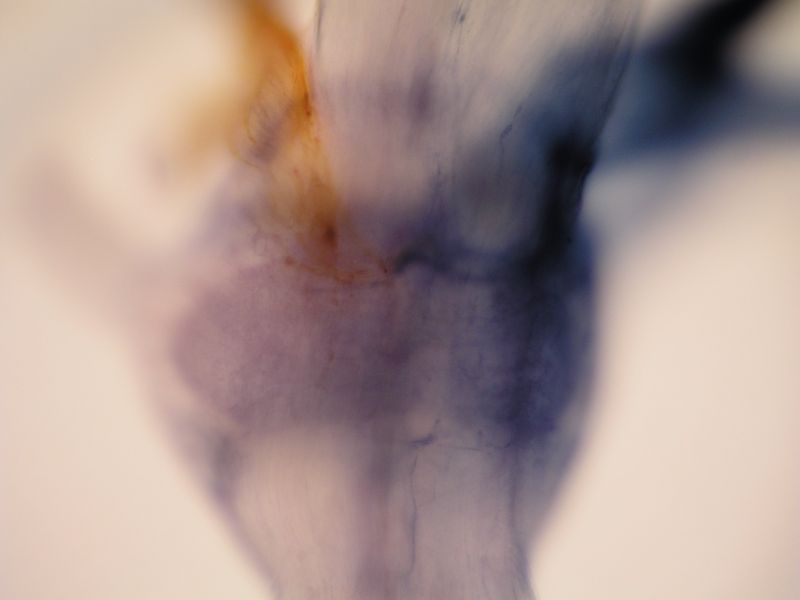

Supplement: Supplemental Information 2 — Micrographs of N2 backfills. Images have been reduced in size. [file peerj-03-1112-s002.zip › A4N2p 2009 0708 (20).jpg]

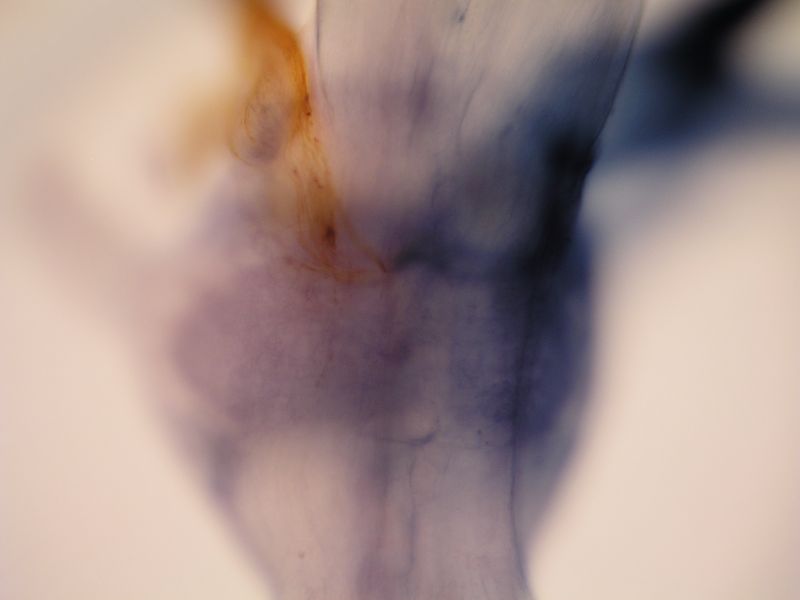

Supplement: Supplemental Information 2 — Micrographs of N2 backfills. Images have been reduced in size. [file peerj-03-1112-s002.zip › A4N2p 2009 0708 (21).jpg]

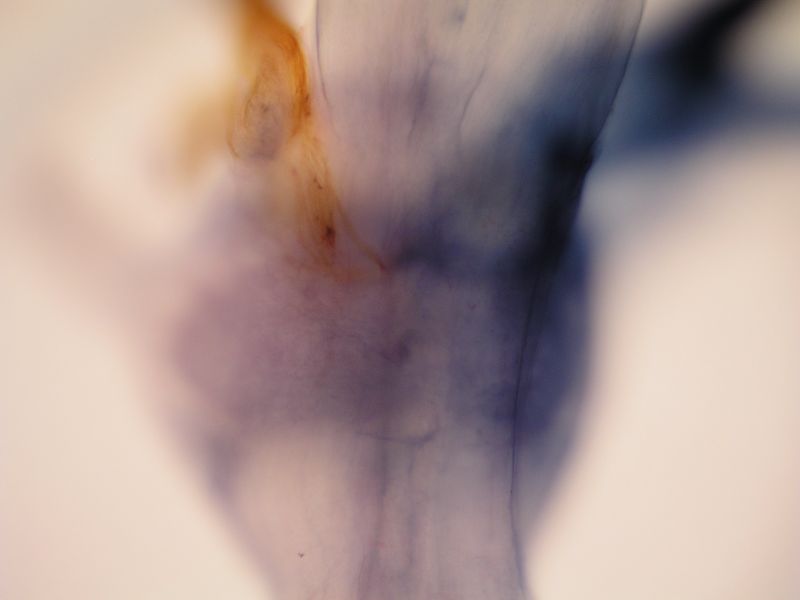

Supplement: Supplemental Information 2 — Micrographs of N2 backfills. Images have been reduced in size. [file peerj-03-1112-s002.zip › A4N2p 2009 0708 (22).jpg]

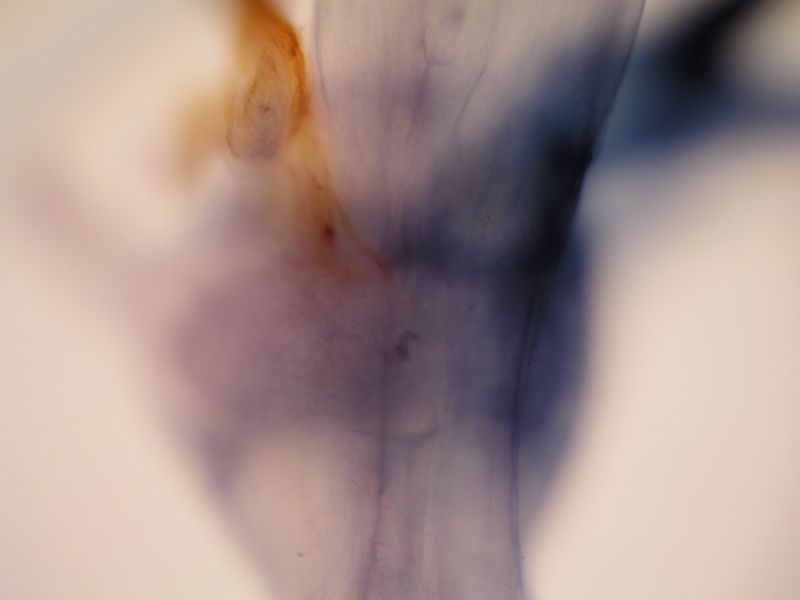

Supplement: Supplemental Information 2 — Micrographs of N2 backfills. Images have been reduced in size. [file peerj-03-1112-s002.zip › A4N2p 2009 0708 (23).jpg]

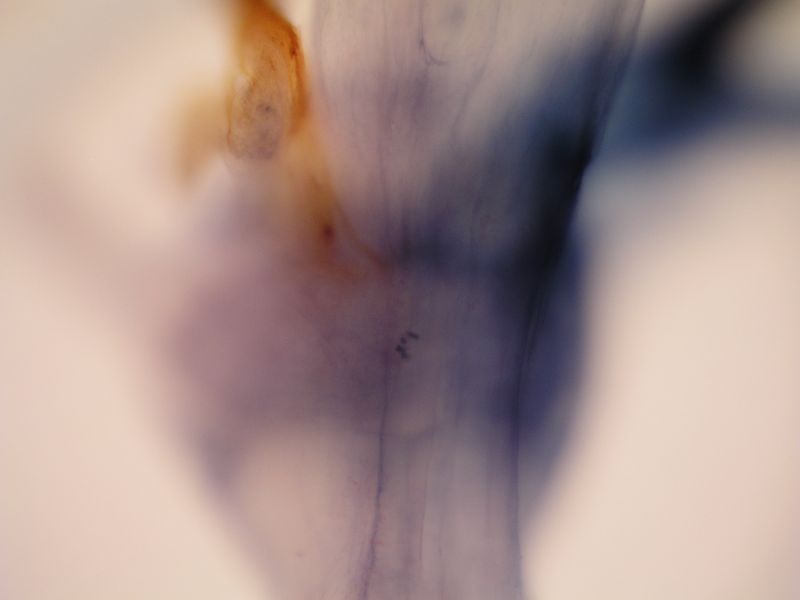

Supplement: Supplemental Information 2 — Micrographs of N2 backfills. Images have been reduced in size. [file peerj-03-1112-s002.zip › A4N2p 2009 0708 (24).jpg]

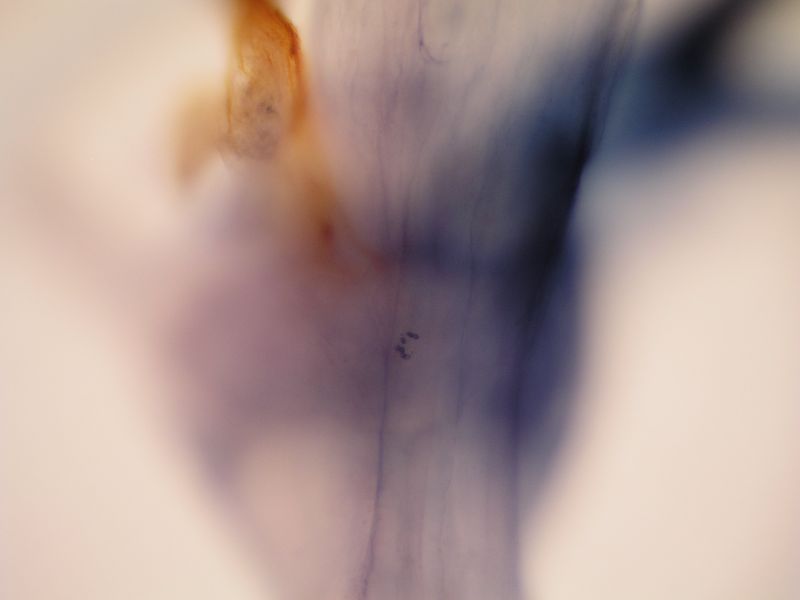

Supplement: Supplemental Information 2 — Micrographs of N2 backfills. Images have been reduced in size. [file peerj-03-1112-s002.zip › A4N2p 2009 0708 (25).jpg]

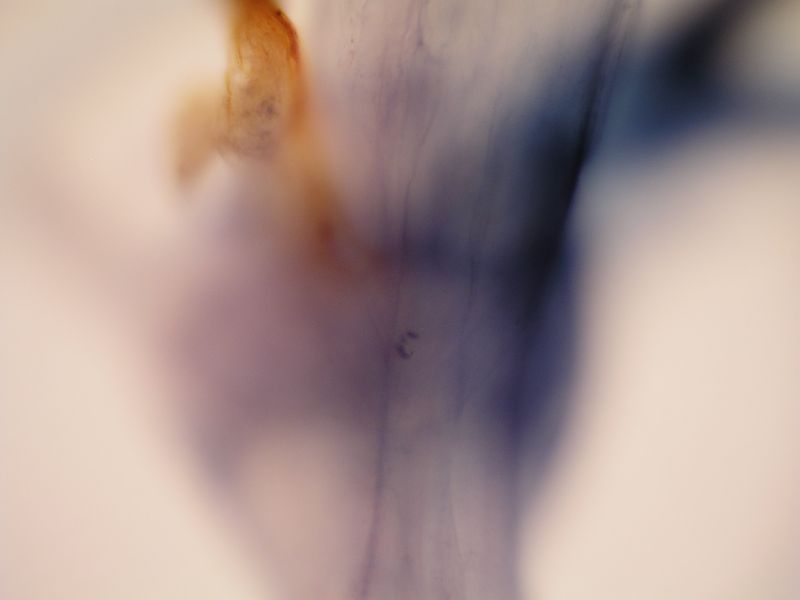

Supplement: Supplemental Information 2 — Micrographs of N2 backfills. Images have been reduced in size. [file peerj-03-1112-s002.zip › A4N2p 2009 0708 (26).jpg]

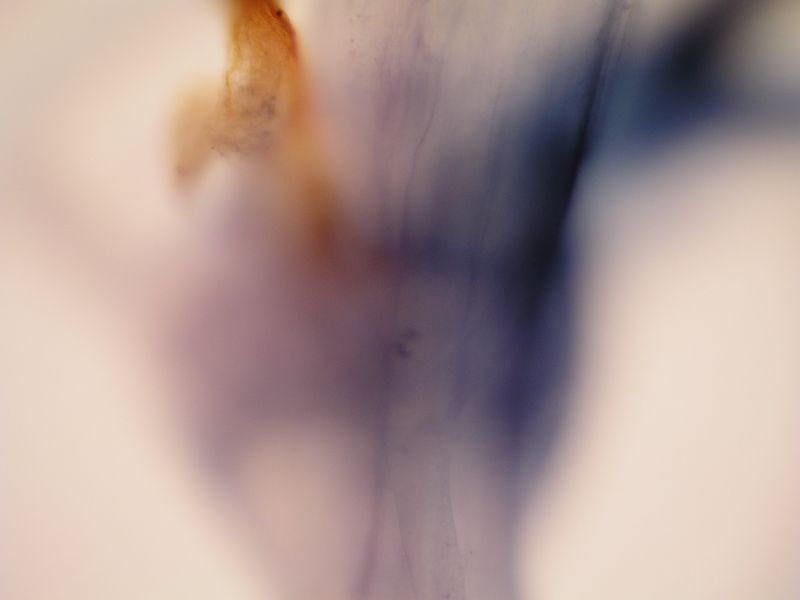

Supplement: Supplemental Information 2 — Micrographs of N2 backfills. Images have been reduced in size. [file peerj-03-1112-s002.zip › A4N2p 2009 0708.jpg]

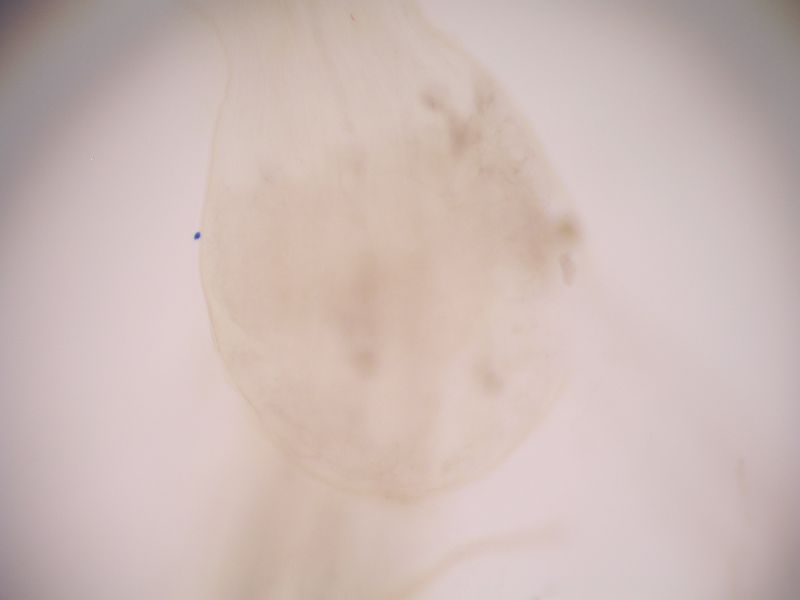

Supplement: Supplemental Information 2 — Micrographs of N2 backfills. Images have been reduced in size. [file peerj-03-1112-s002.zip › A5N2 2009 07 09 (1).jpg]

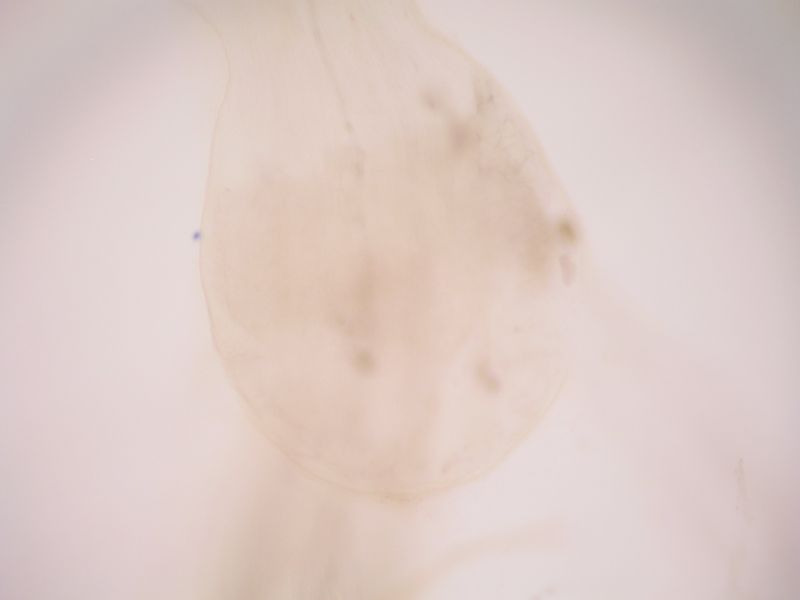

Supplement: Supplemental Information 2 — Micrographs of N2 backfills. Images have been reduced in size. [file peerj-03-1112-s002.zip › A5N2 2009 07 09 (2).jpg]

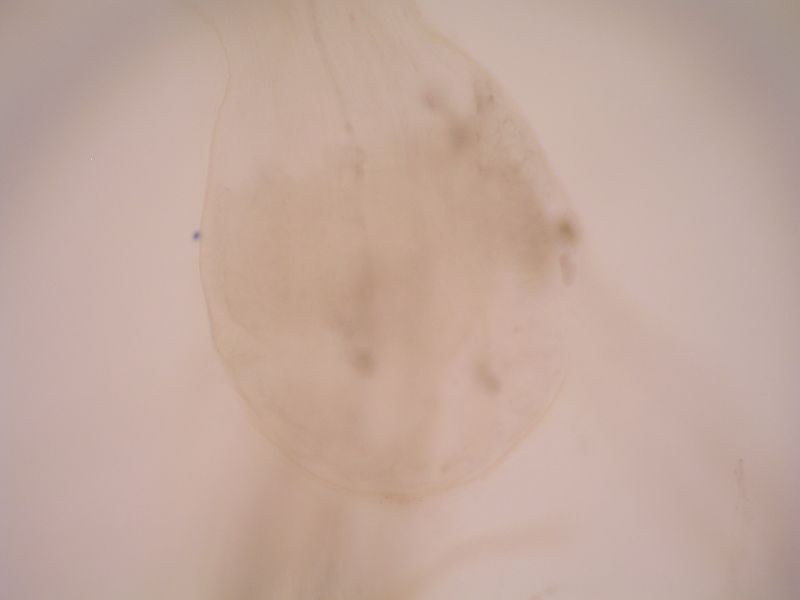

Supplement: Supplemental Information 2 — Micrographs of N2 backfills. Images have been reduced in size. [file peerj-03-1112-s002.zip › A5N2 2009 07 09 (3).jpg]

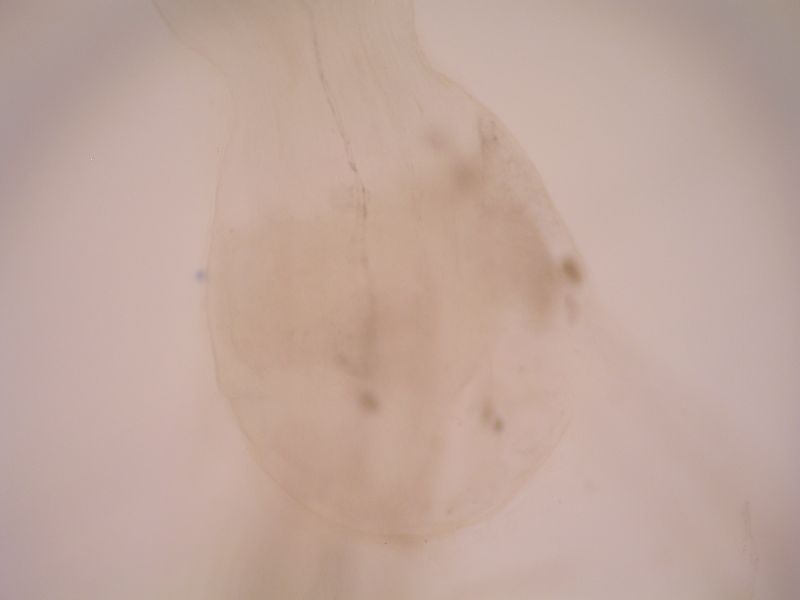

Supplement: Supplemental Information 2 — Micrographs of N2 backfills. Images have been reduced in size. [file peerj-03-1112-s002.zip › A5N2 2009 07 09 (4).jpg]

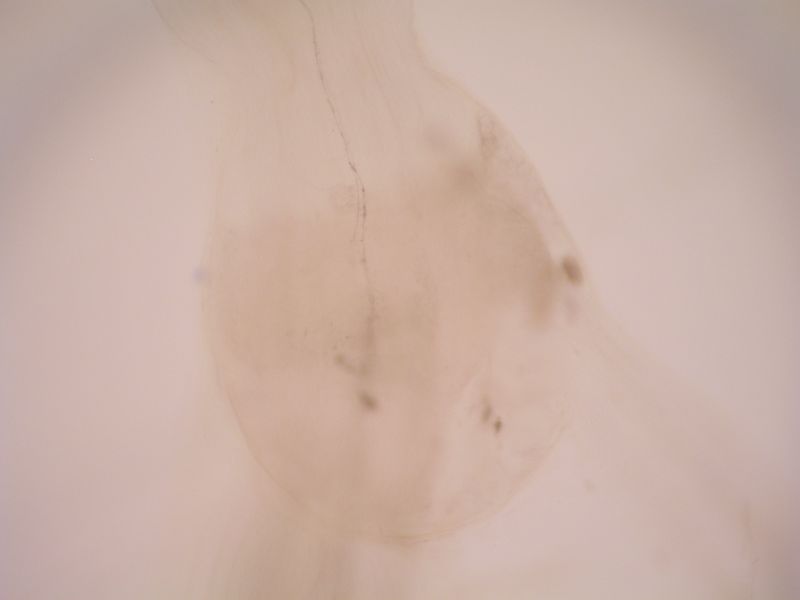

Supplement: Supplemental Information 2 — Micrographs of N2 backfills. Images have been reduced in size. [file peerj-03-1112-s002.zip › A5N2 2009 07 09 (5).jpg]

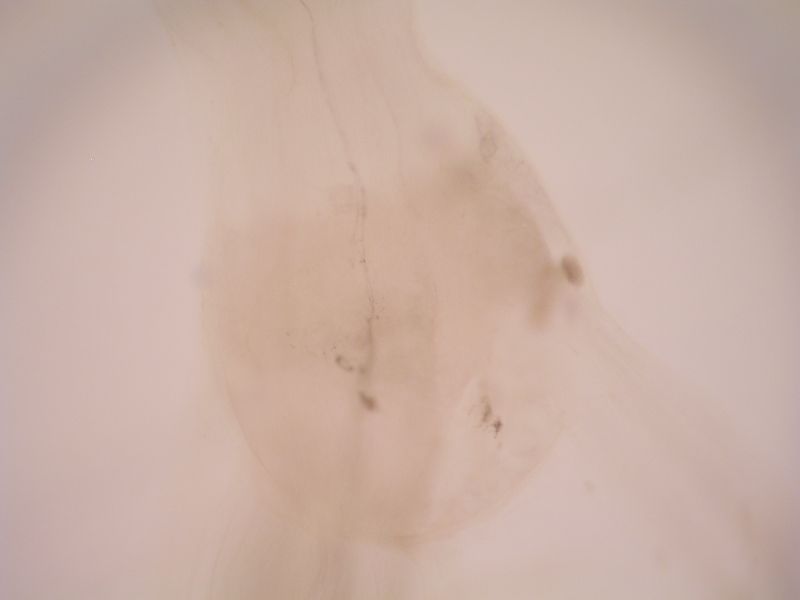

Supplement: Supplemental Information 2 — Micrographs of N2 backfills. Images have been reduced in size. [file peerj-03-1112-s002.zip › A5N2 2009 07 09 (6).jpg]

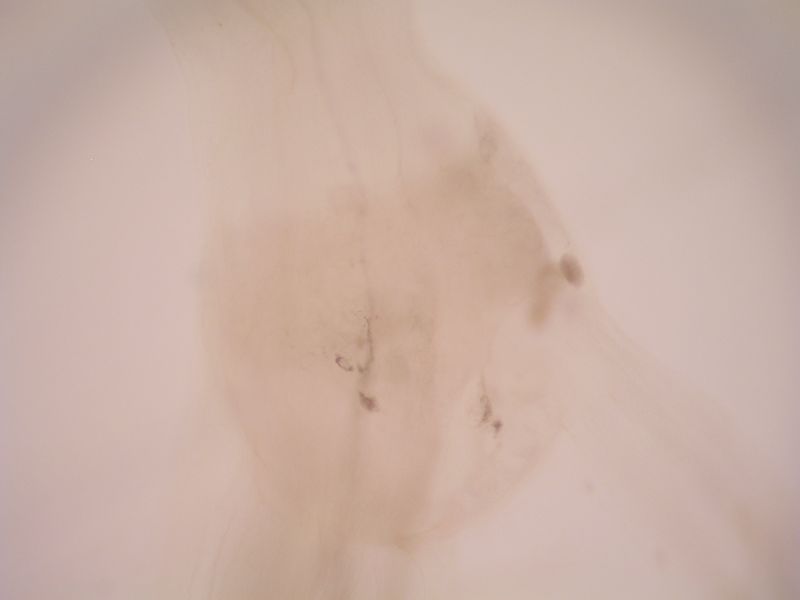

Supplement: Supplemental Information 2 — Micrographs of N2 backfills. Images have been reduced in size. [file peerj-03-1112-s002.zip › A5N2 2009 07 09 (7).jpg]

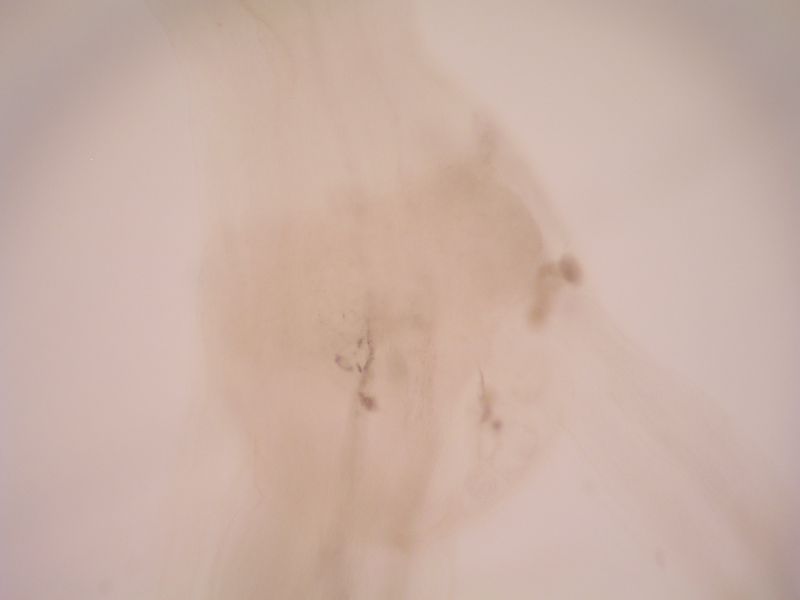

Supplement: Supplemental Information 2 — Micrographs of N2 backfills. Images have been reduced in size. [file peerj-03-1112-s002.zip › A5N2 2009 07 09 (8).jpg]

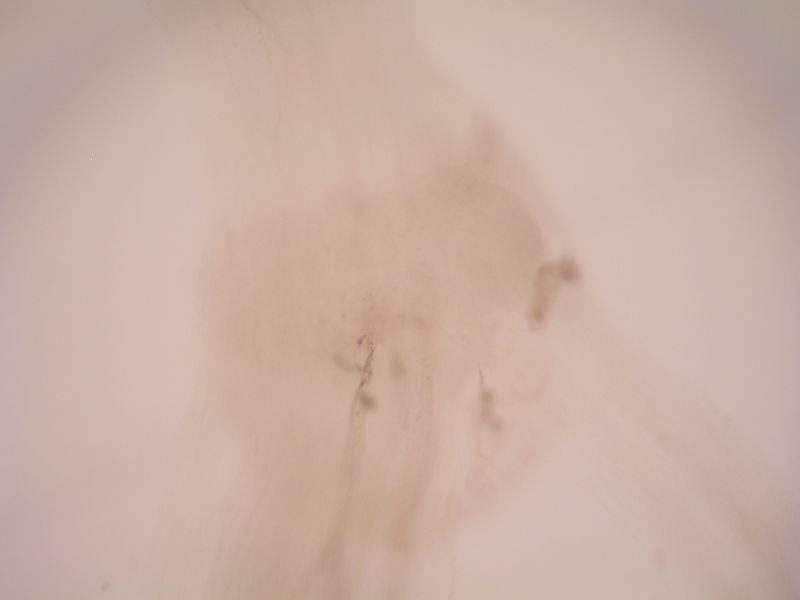

Supplement: Supplemental Information 2 — Micrographs of N2 backfills. Images have been reduced in size. [file peerj-03-1112-s002.zip › A5N2 2009 07 09 (9).jpg]

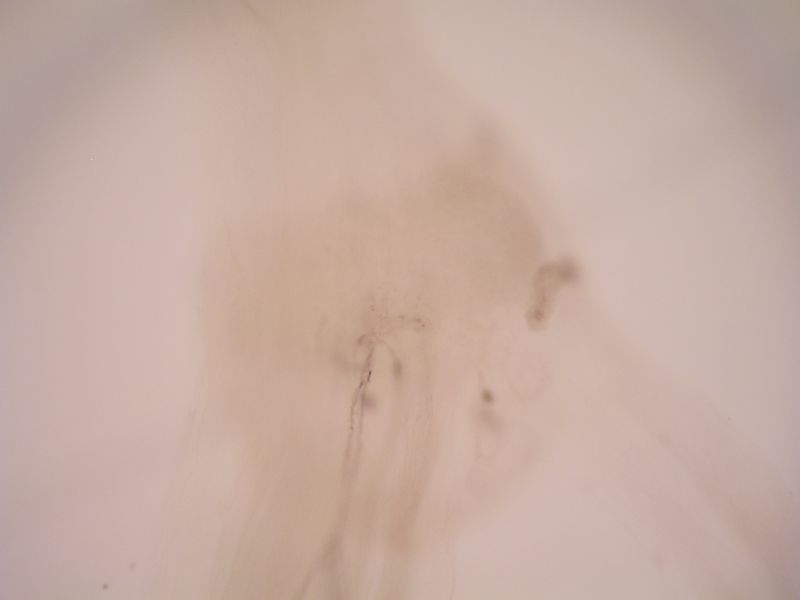

Supplement: Supplemental Information 2 — Micrographs of N2 backfills. Images have been reduced in size. [file peerj-03-1112-s002.zip › A5N2 2009 07 09 (10).jpg]

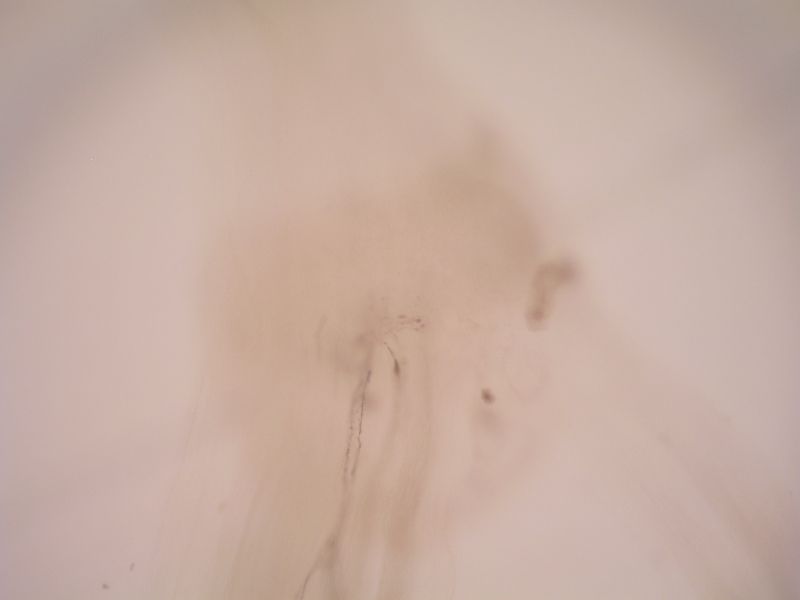

Supplement: Supplemental Information 2 — Micrographs of N2 backfills. Images have been reduced in size. [file peerj-03-1112-s002.zip › A5N2 2009 07 09 (11).jpg]

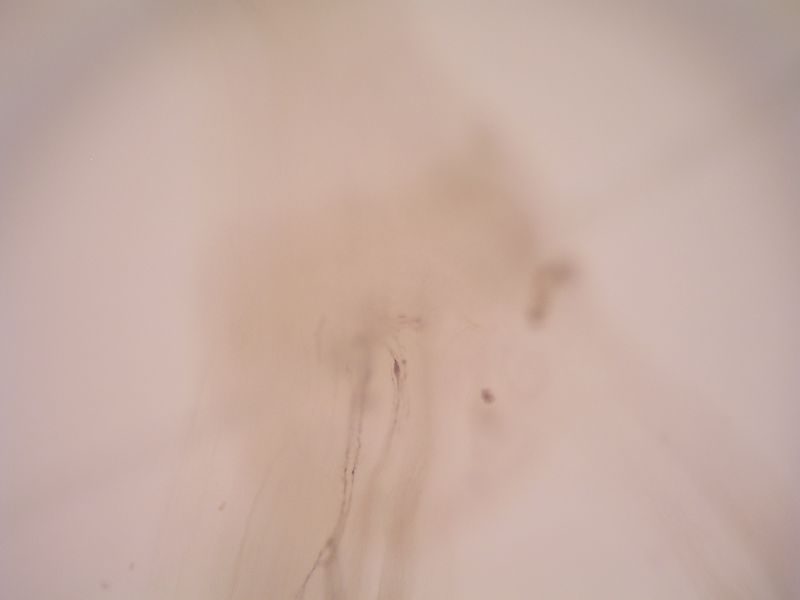

Supplement: Supplemental Information 2 — Micrographs of N2 backfills. Images have been reduced in size. [file peerj-03-1112-s002.zip › A5N2 2009 07 09 (12).jpg]

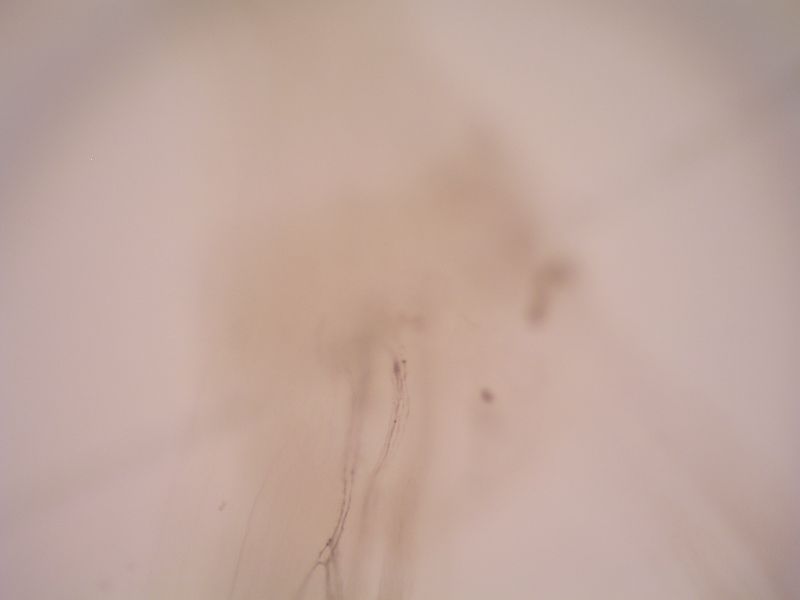

Supplement: Supplemental Information 2 — Micrographs of N2 backfills. Images have been reduced in size. [file peerj-03-1112-s002.zip › A5N2 2009 07 09 (13).jpg]

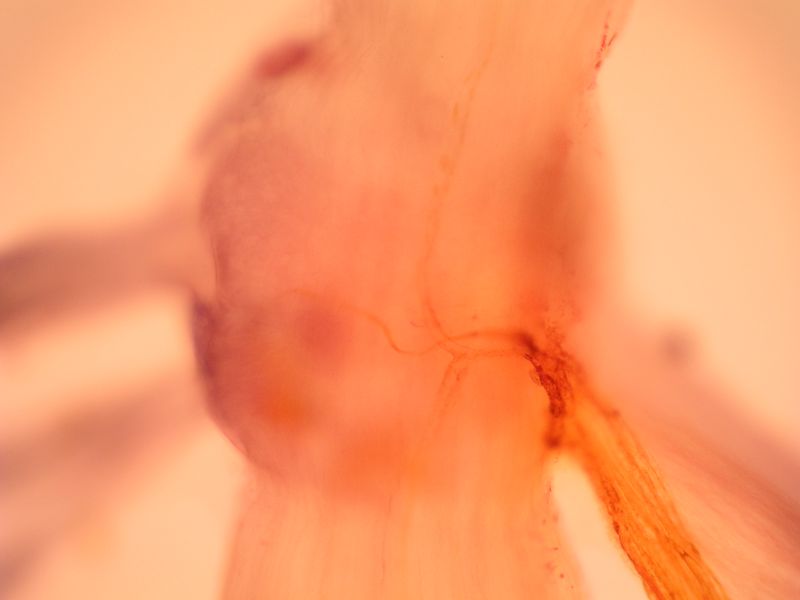

Supplement: Supplemental Information 2 — Micrographs of N3 backfills. Images have been reduced in size. [file peerj-03-1112-s003.zip › A1 N2 P1010065.jpg]
