# Supplementary figures and images for: Motor neurons in the escape response circuit of white shrimp (Litopenaeus setiferus) (part 1 of 4)
Source: PeerJ. 2015 Jul 21;3:e1112. doi: 10.7717/peerj.1112 (PMC4517965; doi:10.7717/peerj.1112)

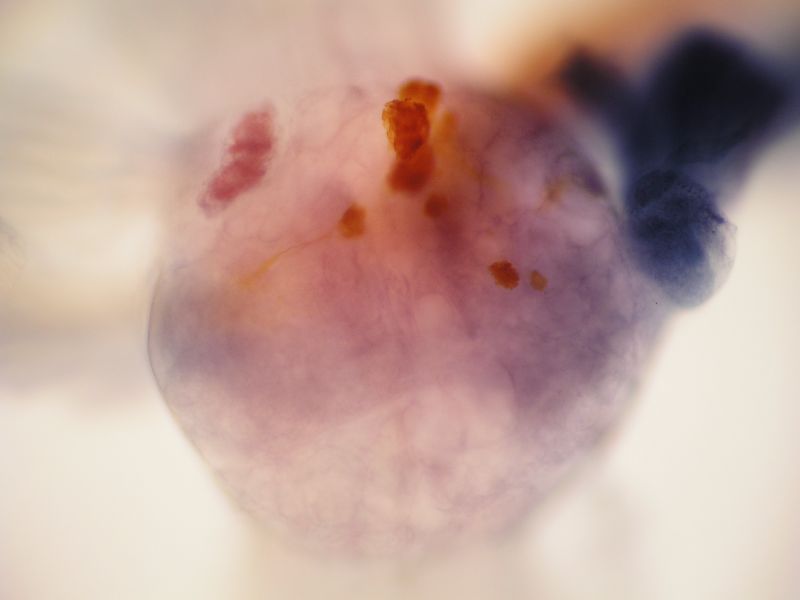

Supplement: Supplemental Information 2 — Micrographs of N2 backfills. Images have been reduced in size. [file peerj-03-1112-s002.zip › 2009 06 26 A2N2 a.jpg]

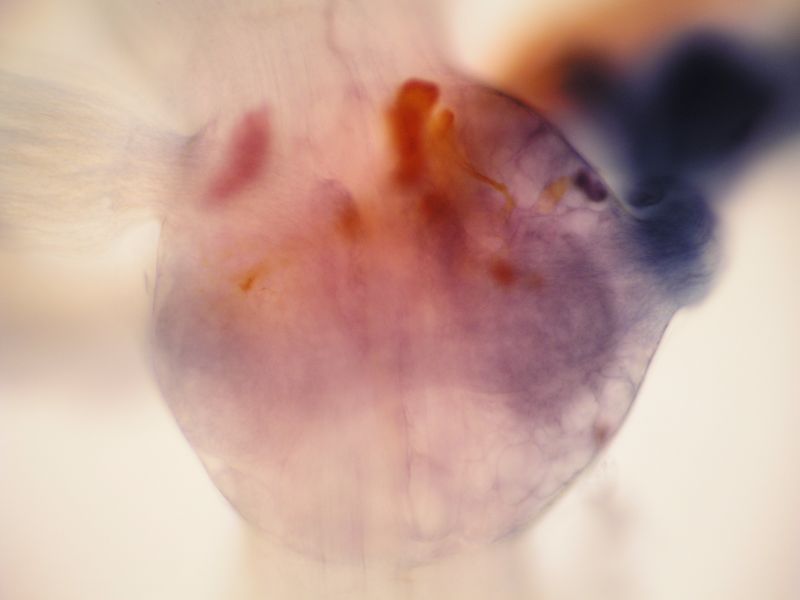

Supplement: Supplemental Information 2 — Micrographs of N2 backfills. Images have been reduced in size. [file peerj-03-1112-s002.zip › 2009 06 26 A2N2 b.jpg]

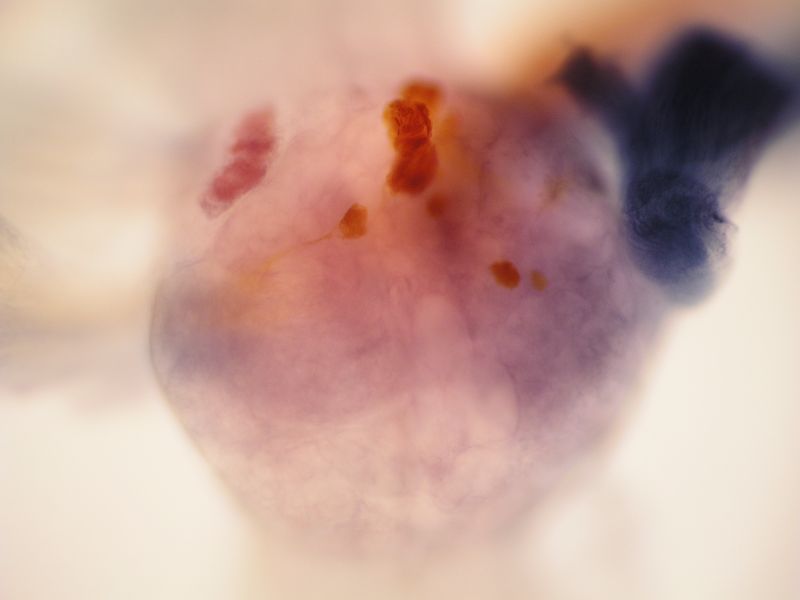

Supplement: Supplemental Information 2 — Micrographs of N2 backfills. Images have been reduced in size. [file peerj-03-1112-s002.zip › 2009 06 26 A2N2 c.jpg]

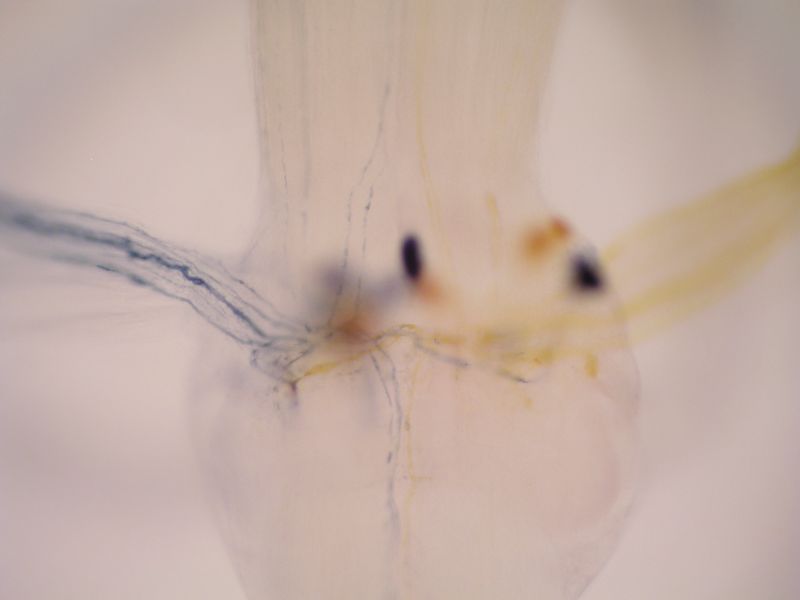

Supplement: Supplemental Information 2 — Micrographs of N2 backfills. Images have been reduced in size. [file peerj-03-1112-s002.zip › 2009 07 07 A1N2 (1).jpg]

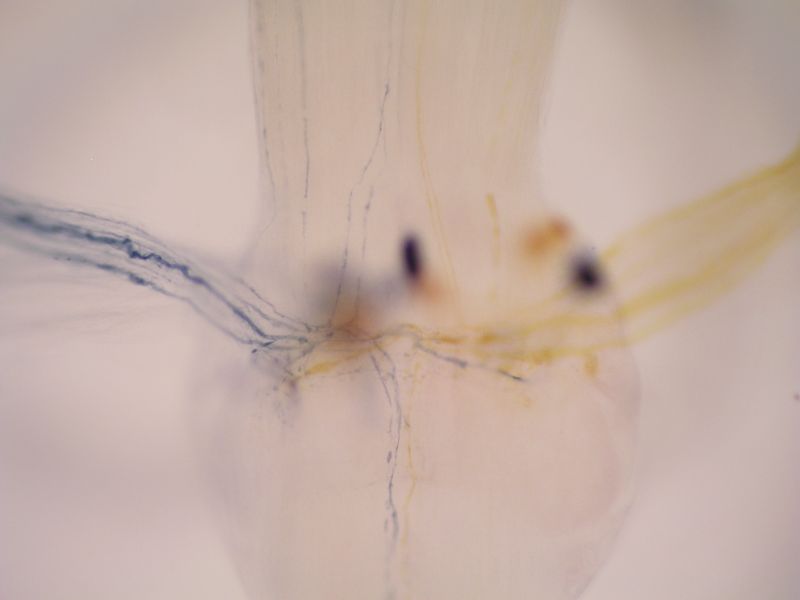

Supplement: Supplemental Information 2 — Micrographs of N2 backfills. Images have been reduced in size. [file peerj-03-1112-s002.zip › 2009 07 07 A1N2 (2).jpg]

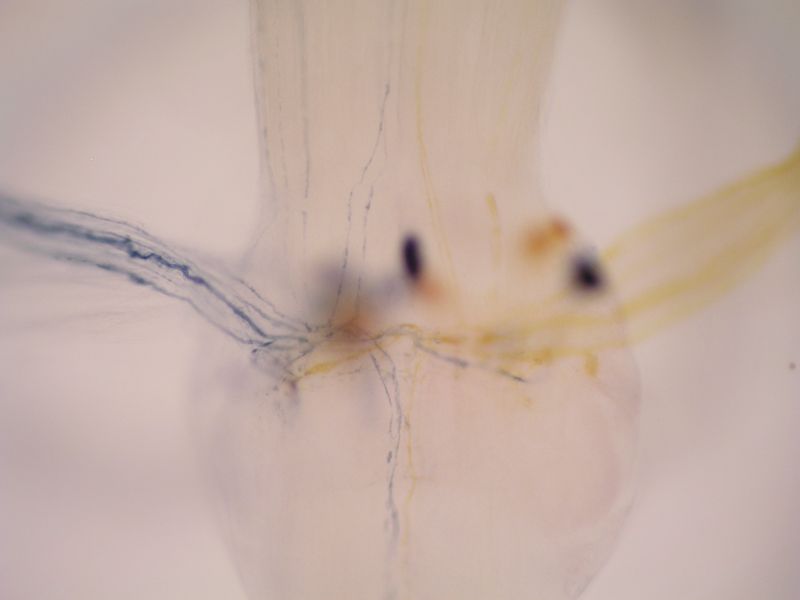

Supplement: Supplemental Information 2 — Micrographs of N2 backfills. Images have been reduced in size. [file peerj-03-1112-s002.zip › 2009 07 07 A1N2 (3).jpg]

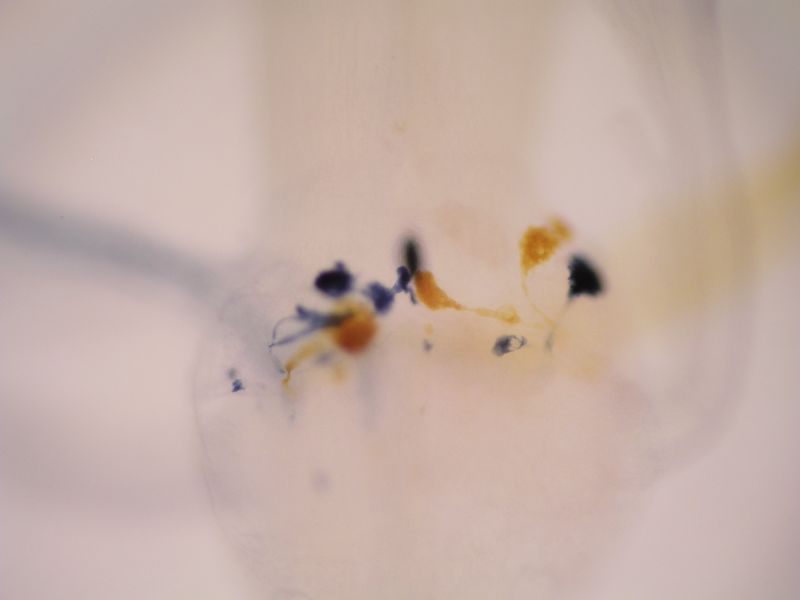

Supplement: Supplemental Information 2 — Micrographs of N2 backfills. Images have been reduced in size. [file peerj-03-1112-s002.zip › 2009 07 07 A1N2 (4).jpg]

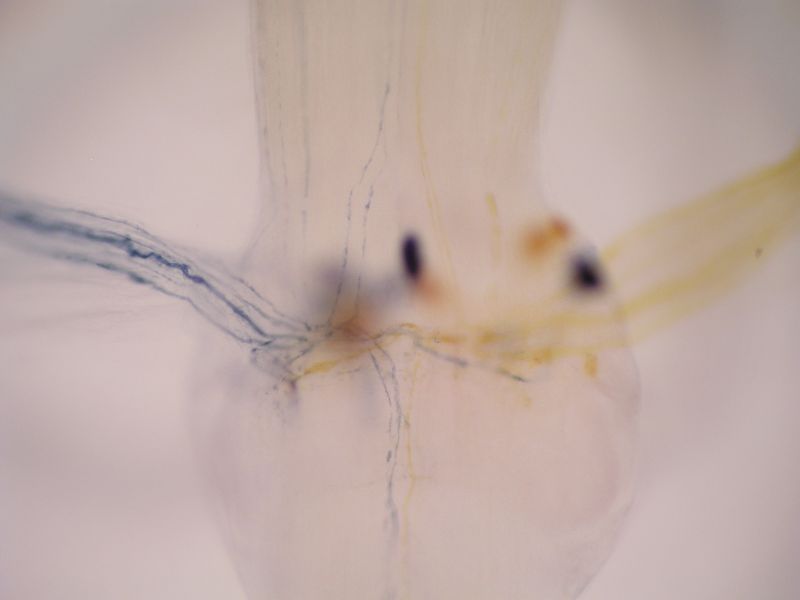

Supplement: Supplemental Information 2 — Micrographs of N2 backfills. Images have been reduced in size. [file peerj-03-1112-s002.zip › 2009 07 07 A1N2.jpg]

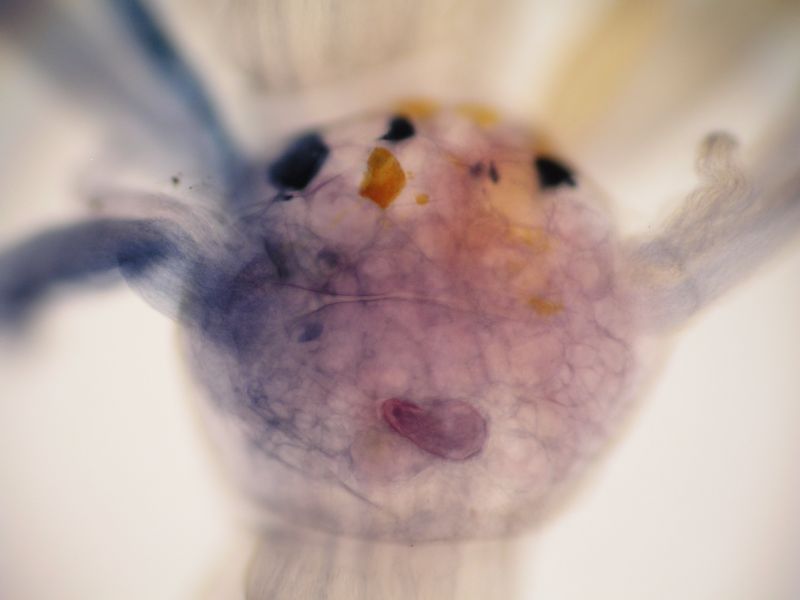

Supplement: Supplemental Information 2 — Micrographs of N2 backfills. Images have been reduced in size. [file peerj-03-1112-s002.zip › 2009 07 07 A2N2 (1).jpg]

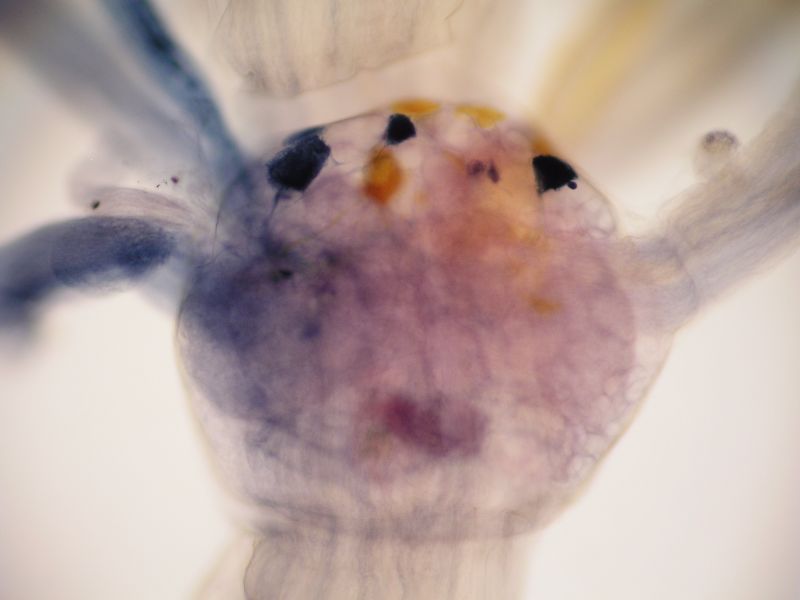

Supplement: Supplemental Information 2 — Micrographs of N2 backfills. Images have been reduced in size. [file peerj-03-1112-s002.zip › 2009 07 07 A2N2 (2).jpg]

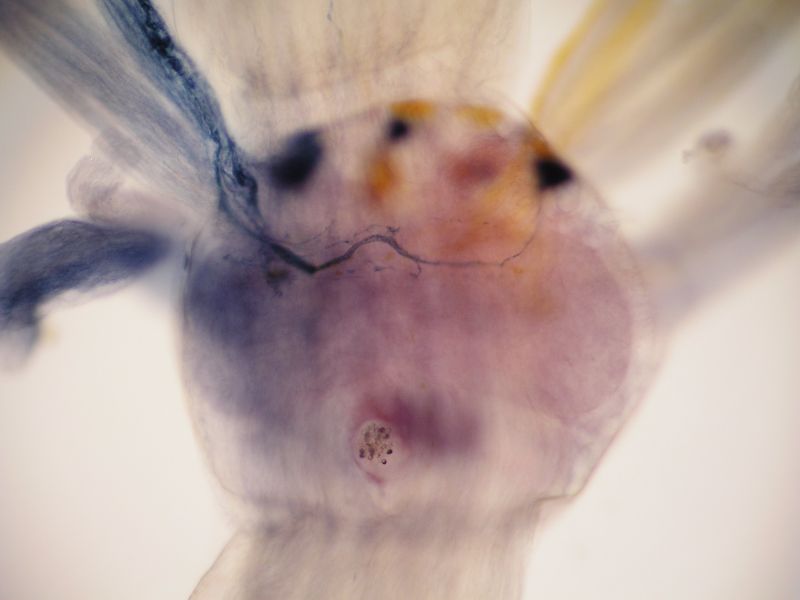

Supplement: Supplemental Information 2 — Micrographs of N2 backfills. Images have been reduced in size. [file peerj-03-1112-s002.zip › 2009 07 07 A2N2 (3).jpg]

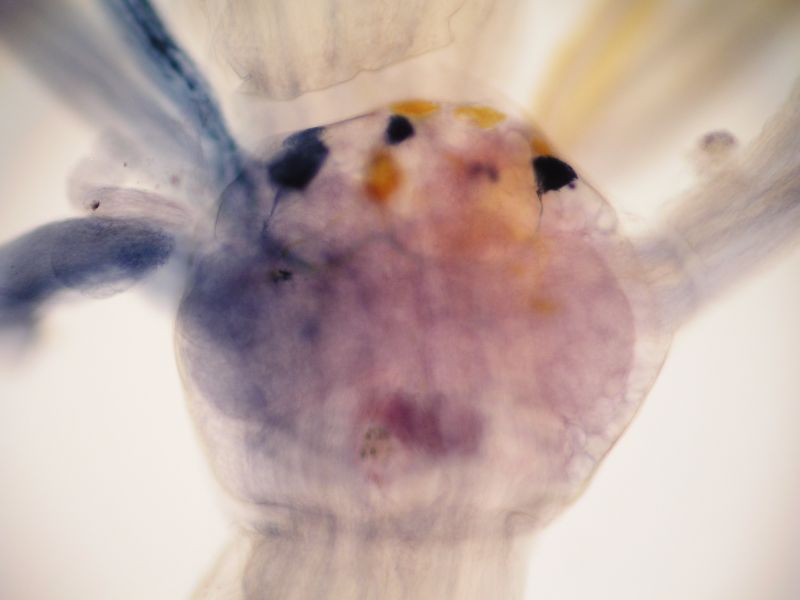

Supplement: Supplemental Information 2 — Micrographs of N2 backfills. Images have been reduced in size. [file peerj-03-1112-s002.zip › 2009 07 07 A2N2 (4).jpg]

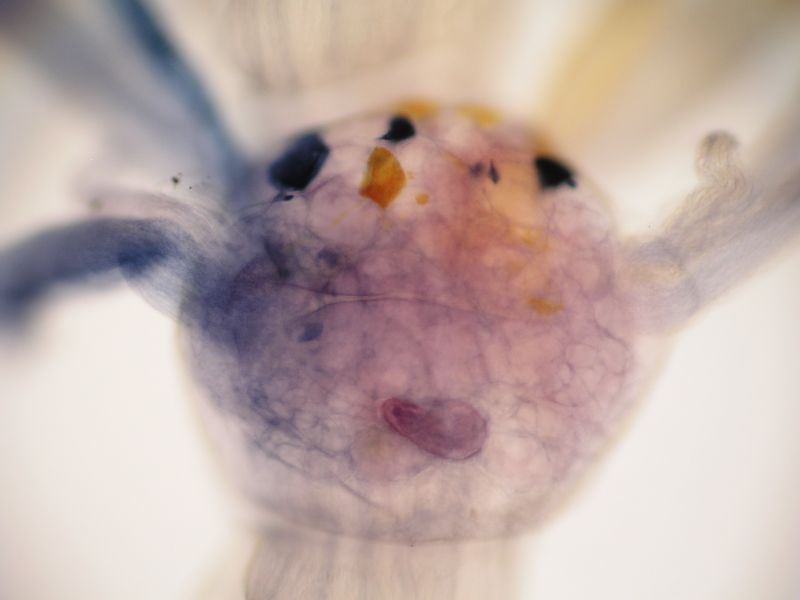

Supplement: Supplemental Information 2 — Micrographs of N2 backfills. Images have been reduced in size. [file peerj-03-1112-s002.zip › 2009 07 07 A2N2 (5).jpg]

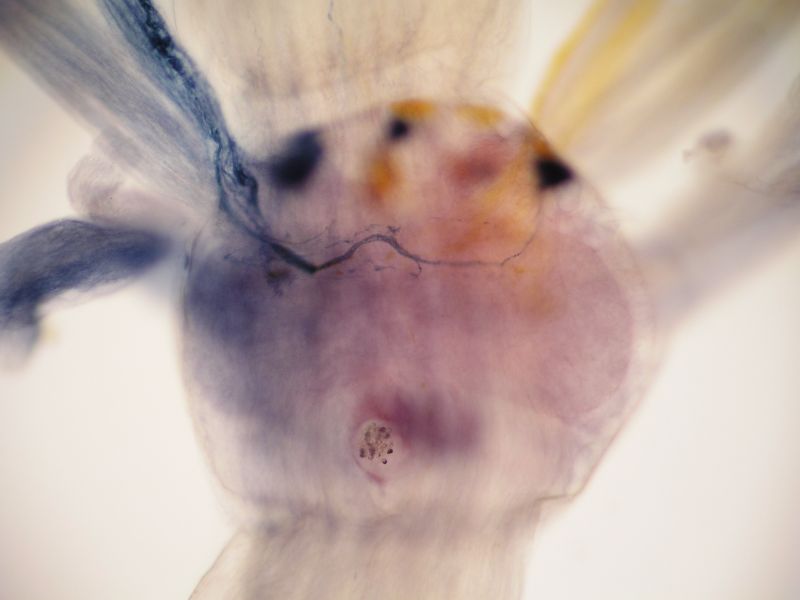

Supplement: Supplemental Information 2 — Micrographs of N2 backfills. Images have been reduced in size. [file peerj-03-1112-s002.zip › 2009 07 07 A2N2.jpg]

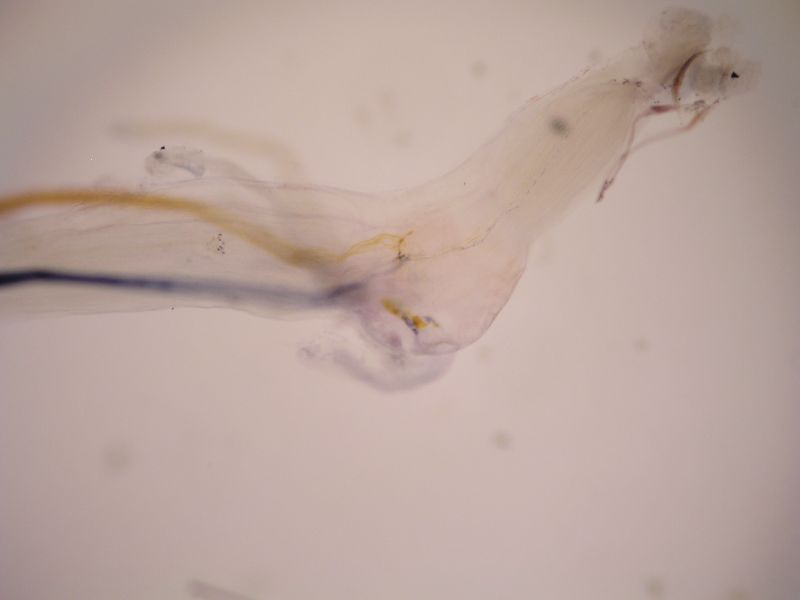

Supplement: Supplemental Information 2 — Micrographs of N2 backfills. Images have been reduced in size. [file peerj-03-1112-s002.zip › 2009 07 07 A4N2 (1).jpg]

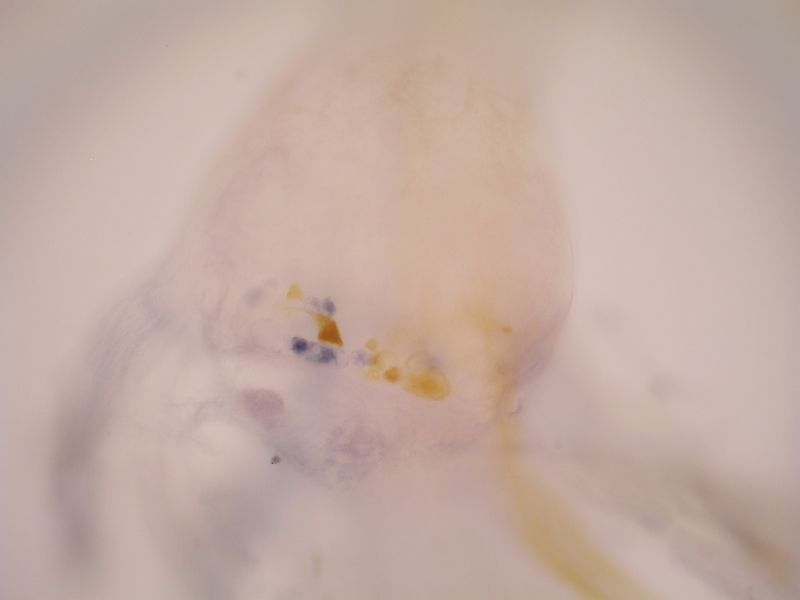

Supplement: Supplemental Information 2 — Micrographs of N2 backfills. Images have been reduced in size. [file peerj-03-1112-s002.zip › 2009 07 07 A4N2 (2).jpg]

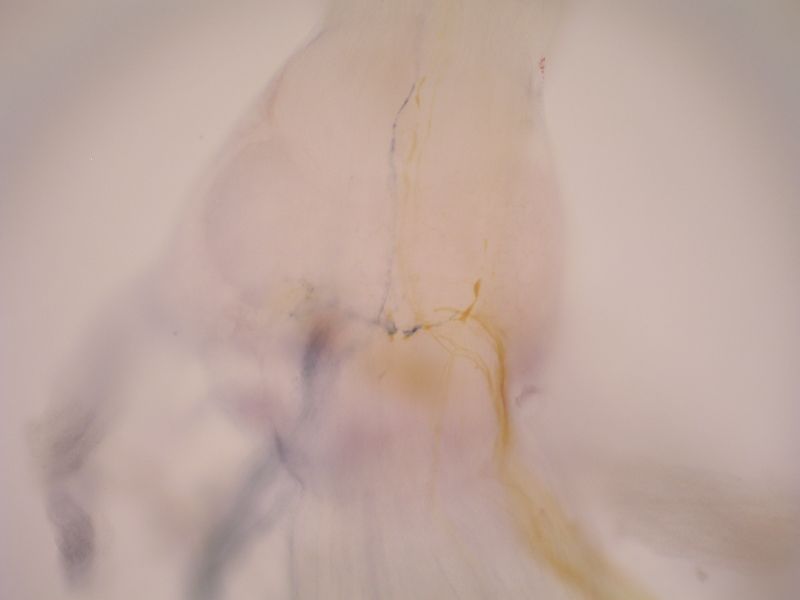

Supplement: Supplemental Information 2 — Micrographs of N2 backfills. Images have been reduced in size. [file peerj-03-1112-s002.zip › 2009 07 07 A4N2 (3).jpg]

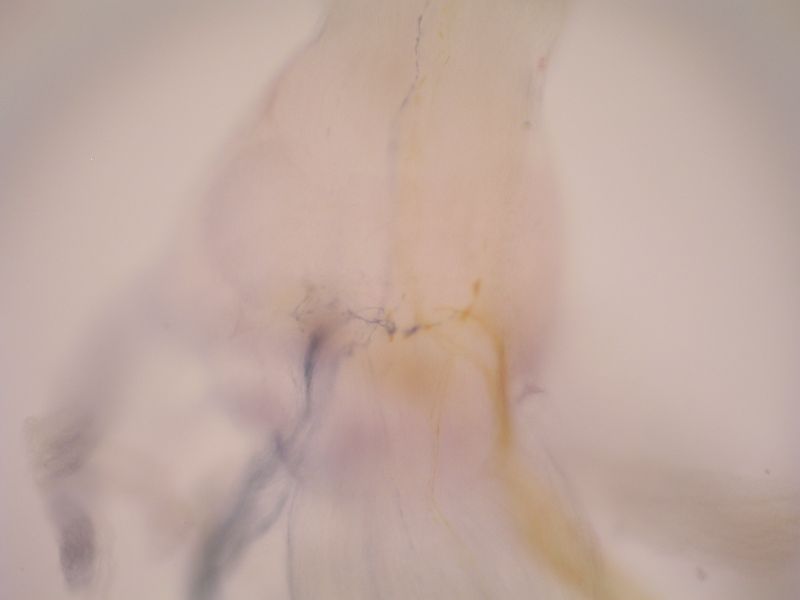

Supplement: Supplemental Information 2 — Micrographs of N2 backfills. Images have been reduced in size. [file peerj-03-1112-s002.zip › 2009 07 07 A4N2.jpg]

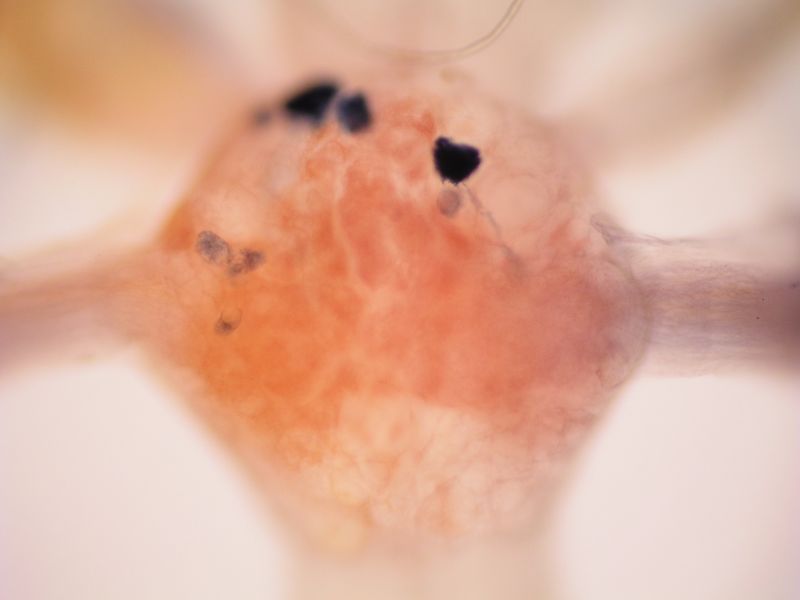

Supplement: Supplemental Information 2 — Micrographs of N2 backfills. Images have been reduced in size. [file peerj-03-1112-s002.zip › 20090623 A2N2 a.jpg]

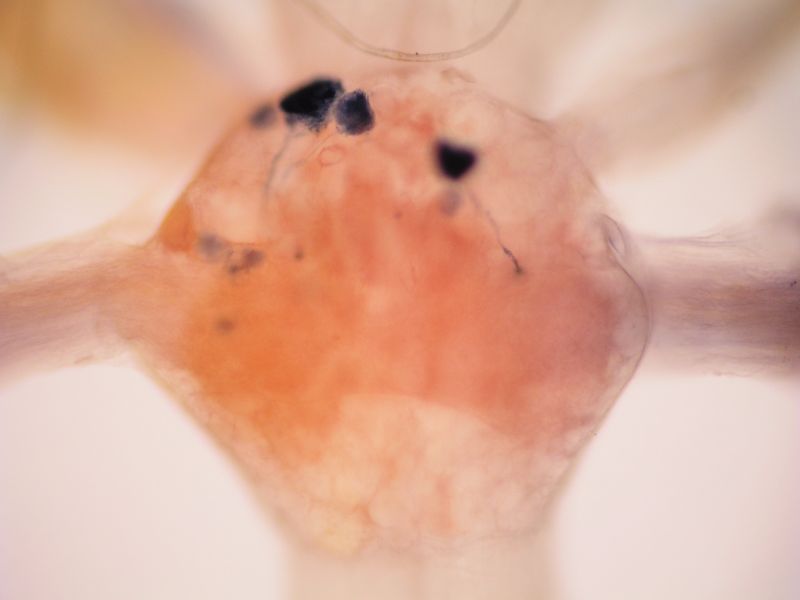

Supplement: Supplemental Information 2 — Micrographs of N2 backfills. Images have been reduced in size. [file peerj-03-1112-s002.zip › 20090623 A2N2 b.jpg]

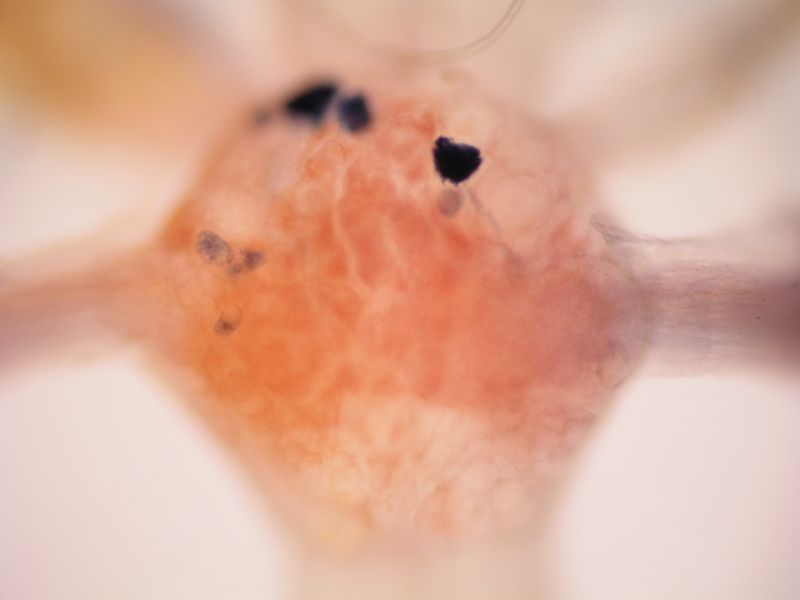

Supplement: Supplemental Information 2 — Micrographs of N2 backfills. Images have been reduced in size. [file peerj-03-1112-s002.zip › 20090623 A2N2 c.jpg]

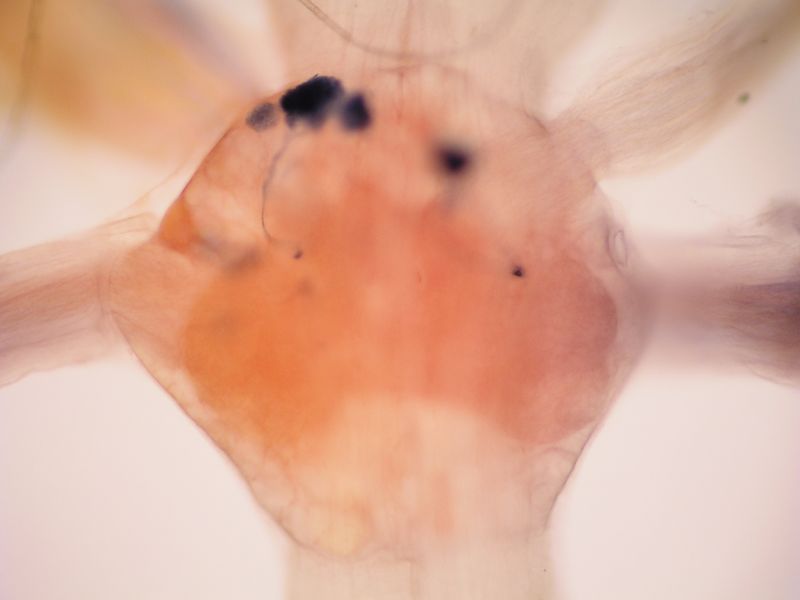

Supplement: Supplemental Information 2 — Micrographs of N2 backfills. Images have been reduced in size. [file peerj-03-1112-s002.zip › 20090623 A2N2 d.jpg]

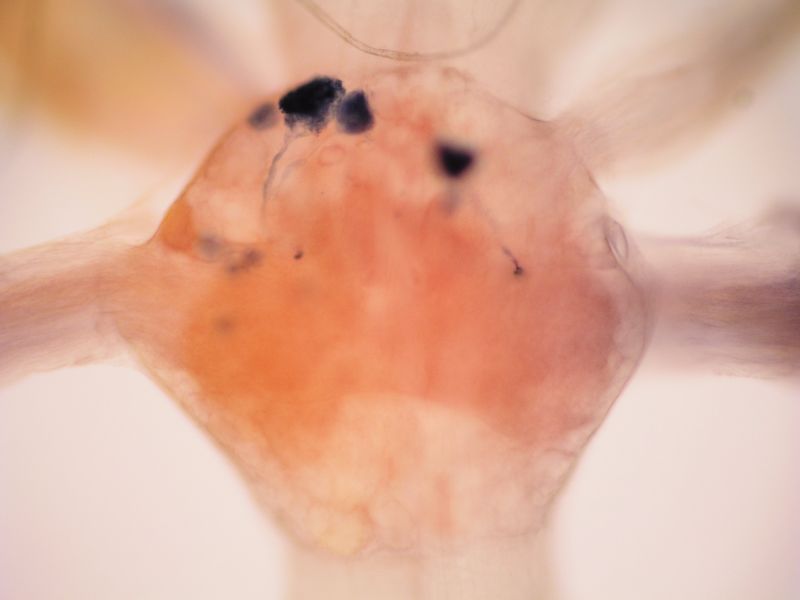

Supplement: Supplemental Information 2 — Micrographs of N2 backfills. Images have been reduced in size. [file peerj-03-1112-s002.zip › 20090623 A2N2 e.jpg]

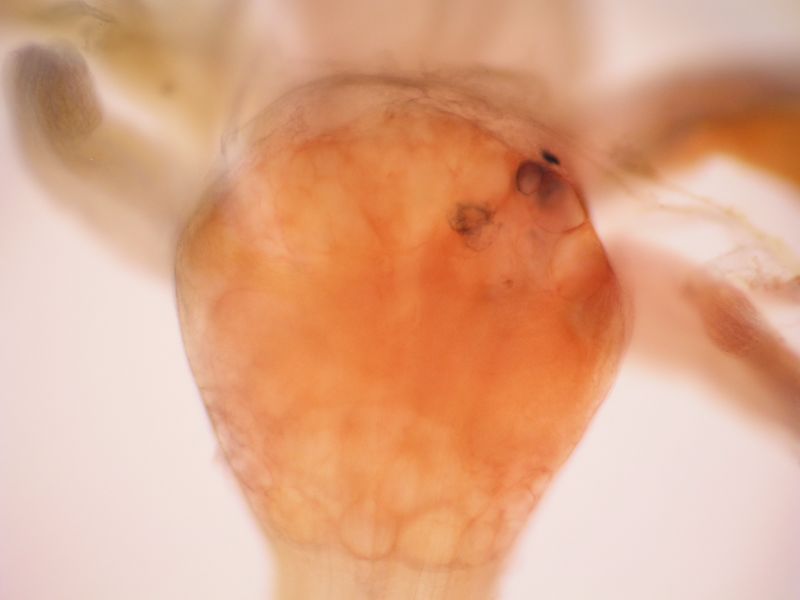

Supplement: Supplemental Information 2 — Micrographs of N2 backfills. Images have been reduced in size. [file peerj-03-1112-s002.zip › 20090623 A4N2 a.jpg]

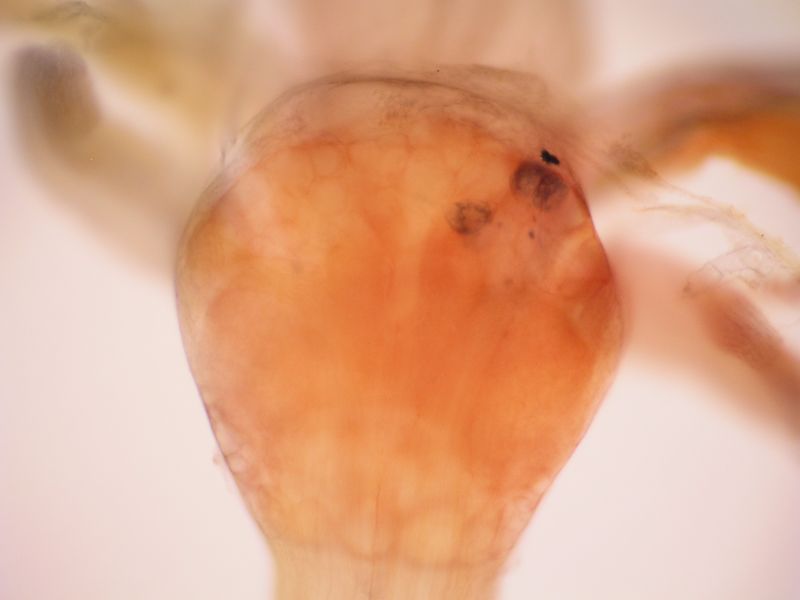

Supplement: Supplemental Information 2 — Micrographs of N2 backfills. Images have been reduced in size. [file peerj-03-1112-s002.zip › 20090623 A4N2 b.jpg]

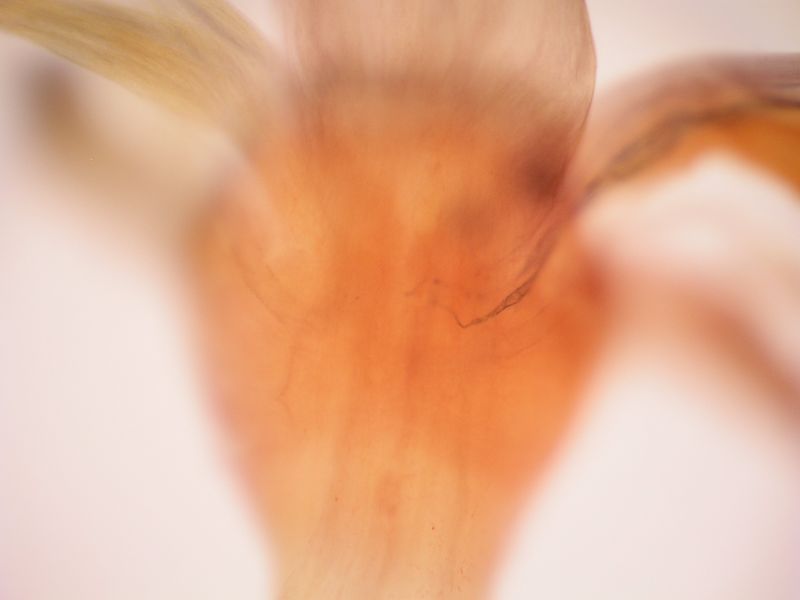

Supplement: Supplemental Information 2 — Micrographs of N2 backfills. Images have been reduced in size. [file peerj-03-1112-s002.zip › 20090623 A4N2 c.jpg]

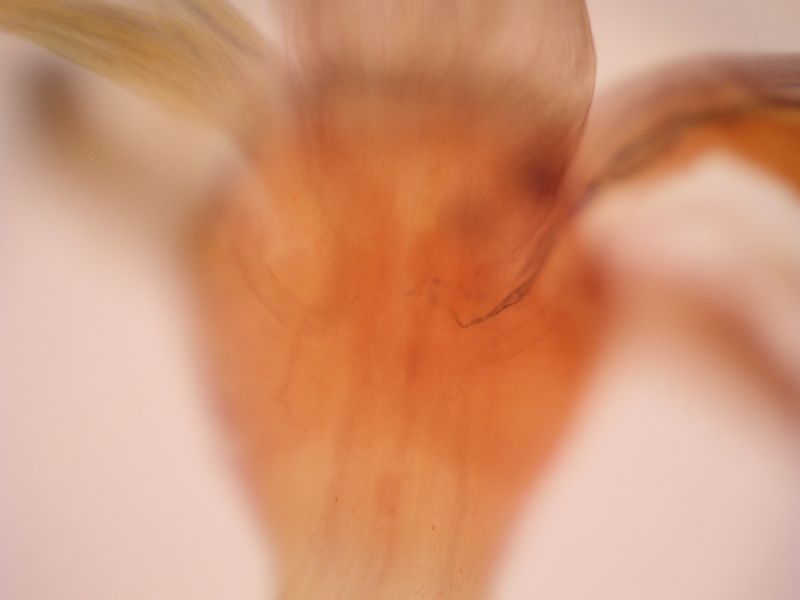

Supplement: Supplemental Information 2 — Micrographs of N2 backfills. Images have been reduced in size. [file peerj-03-1112-s002.zip › 20090623 A4N2 d.jpg]

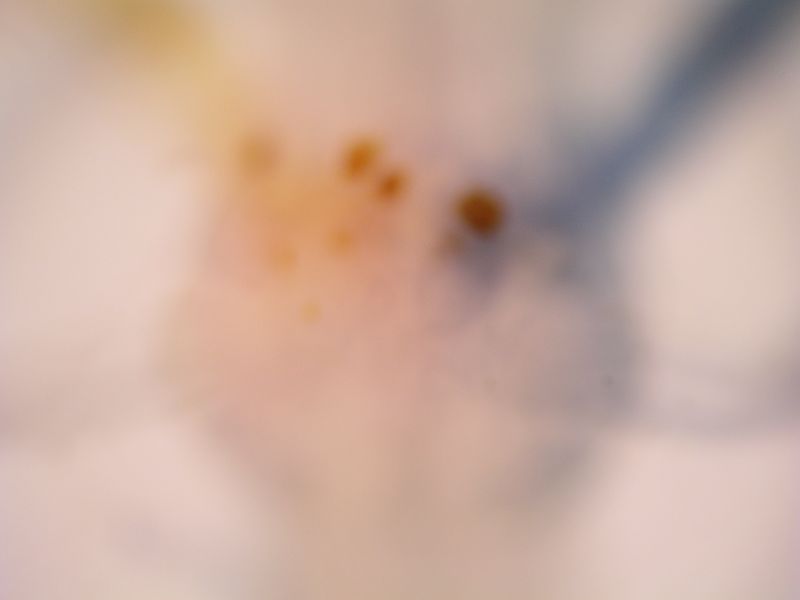

Supplement: Supplemental Information 2 — Micrographs of N2 backfills. Images have been reduced in size. [file peerj-03-1112-s002.zip › A1N2 2008 07 08 (1).jpg]

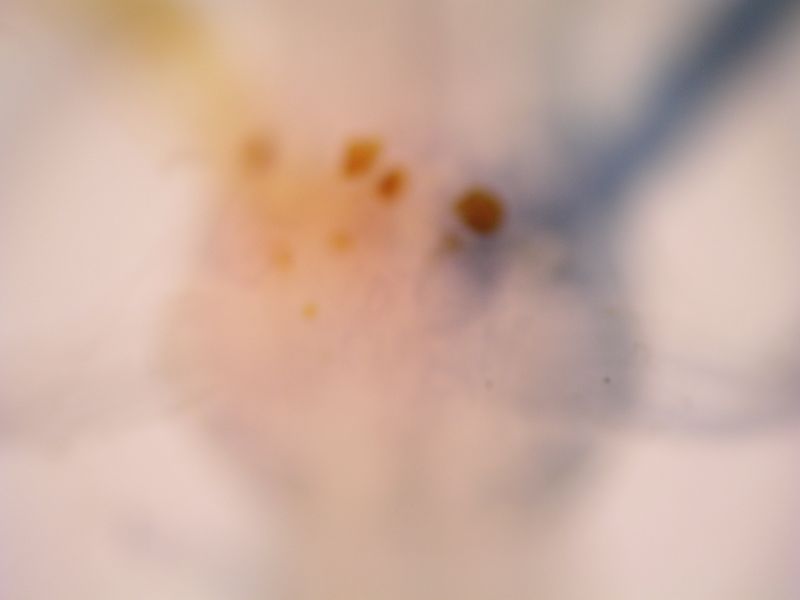

Supplement: Supplemental Information 2 — Micrographs of N2 backfills. Images have been reduced in size. [file peerj-03-1112-s002.zip › A1N2 2008 07 08 (2).jpg]

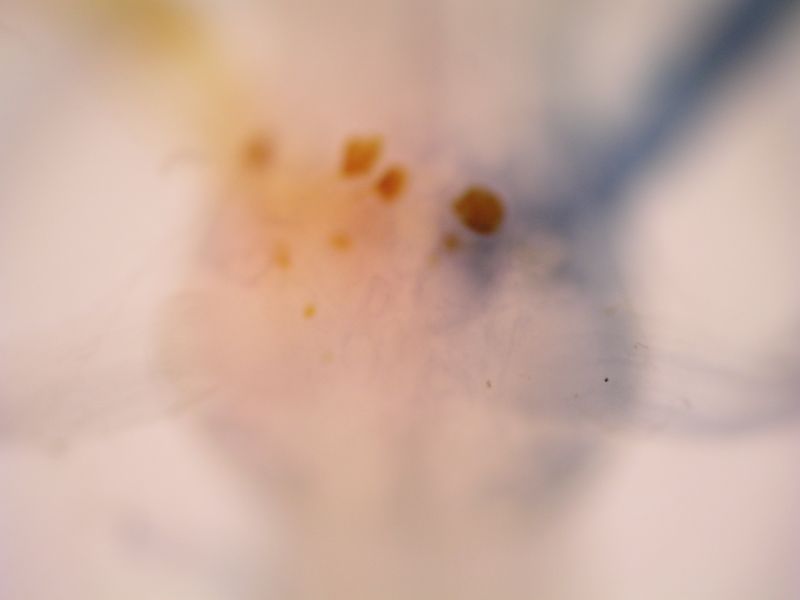

Supplement: Supplemental Information 2 — Micrographs of N2 backfills. Images have been reduced in size. [file peerj-03-1112-s002.zip › A1N2 2008 07 08 (3).jpg]

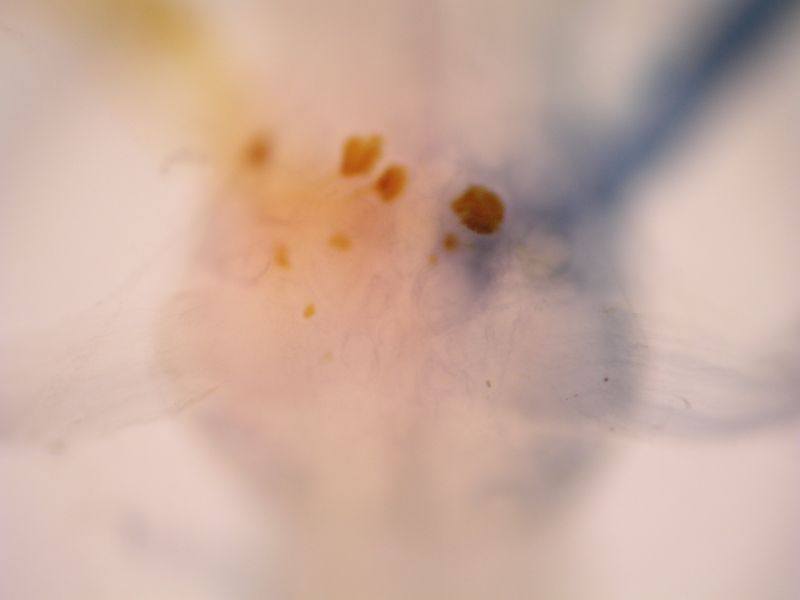

Supplement: Supplemental Information 2 — Micrographs of N2 backfills. Images have been reduced in size. [file peerj-03-1112-s002.zip › A1N2 2008 07 08 (4).jpg]

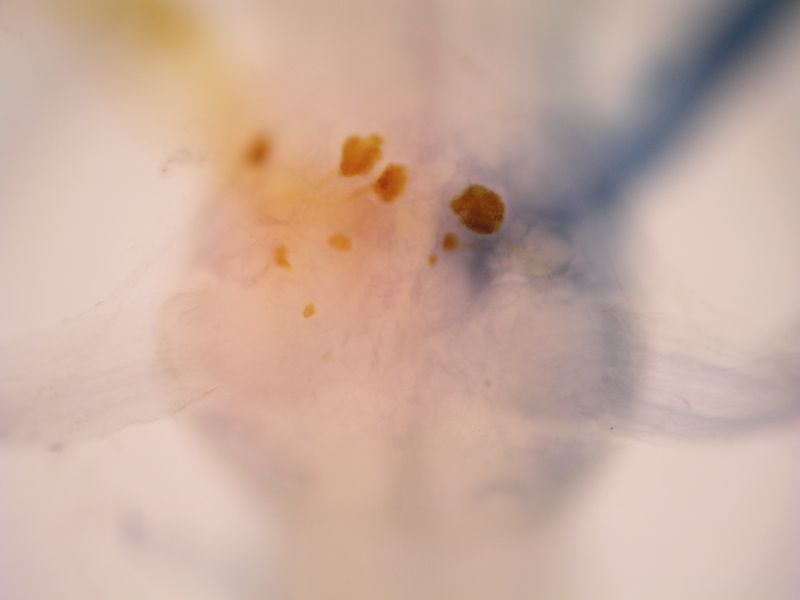

Supplement: Supplemental Information 2 — Micrographs of N2 backfills. Images have been reduced in size. [file peerj-03-1112-s002.zip › A1N2 2008 07 08 (5).jpg]

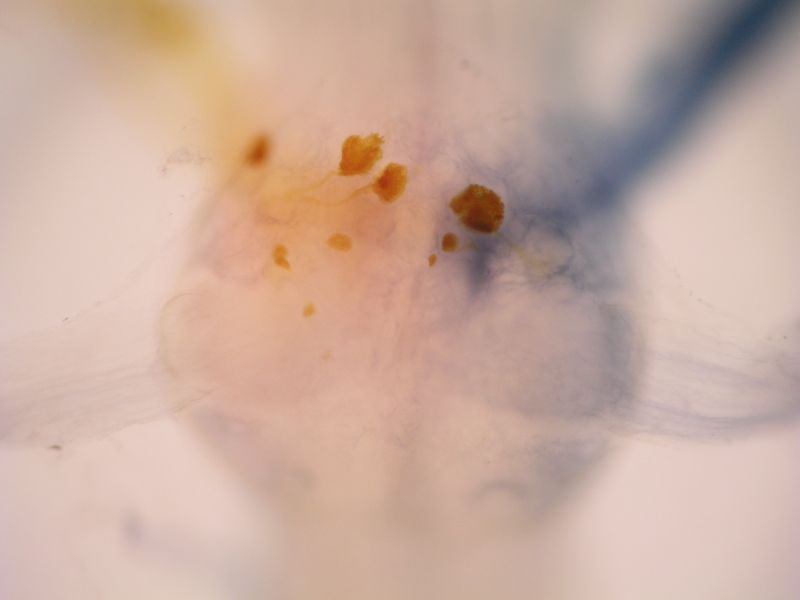

Supplement: Supplemental Information 2 — Micrographs of N2 backfills. Images have been reduced in size. [file peerj-03-1112-s002.zip › A1N2 2008 07 08 (6).jpg]

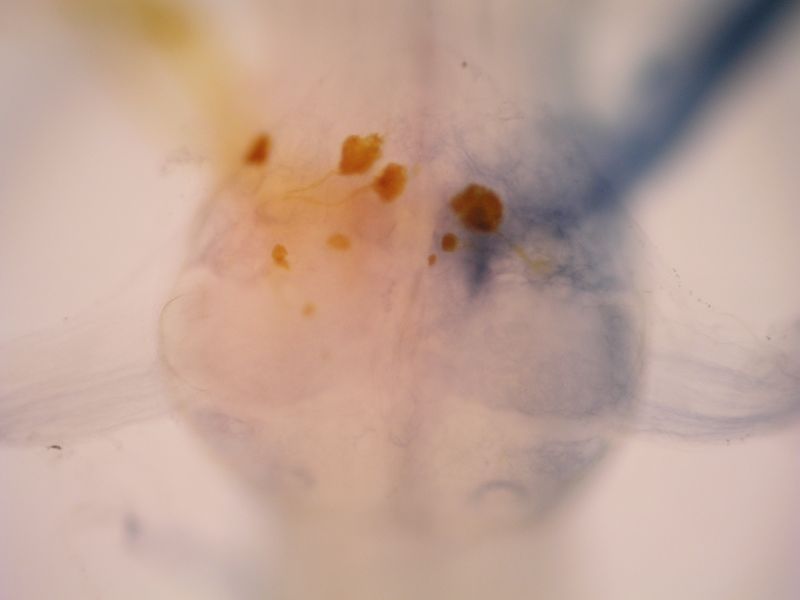

Supplement: Supplemental Information 2 — Micrographs of N2 backfills. Images have been reduced in size. [file peerj-03-1112-s002.zip › A1N2 2008 07 08 (7).jpg]

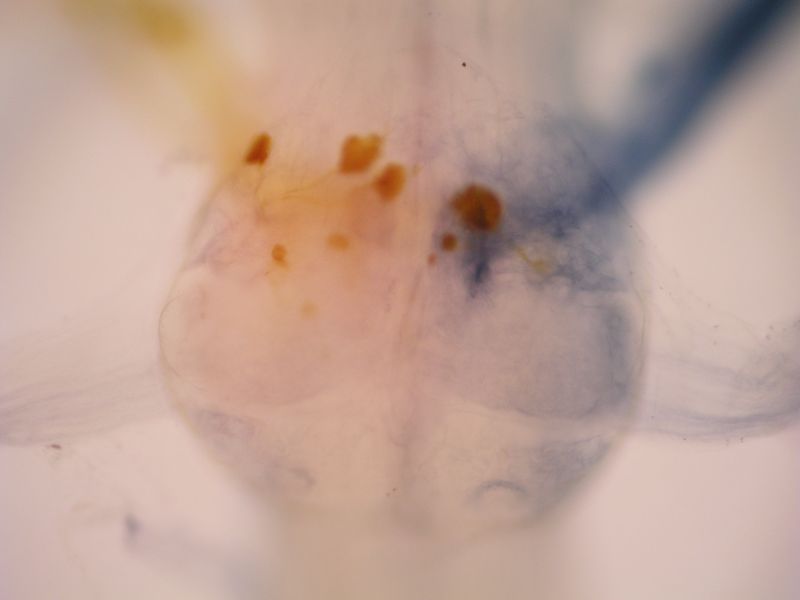

Supplement: Supplemental Information 2 — Micrographs of N2 backfills. Images have been reduced in size. [file peerj-03-1112-s002.zip › A1N2 2008 07 08 (8).jpg]

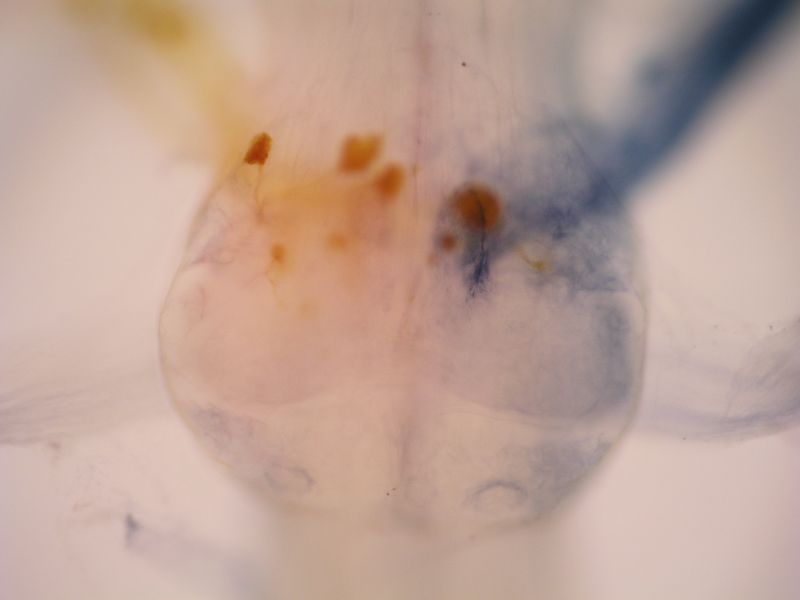

Supplement: Supplemental Information 2 — Micrographs of N2 backfills. Images have been reduced in size. [file peerj-03-1112-s002.zip › A1N2 2008 07 08 (9).jpg]

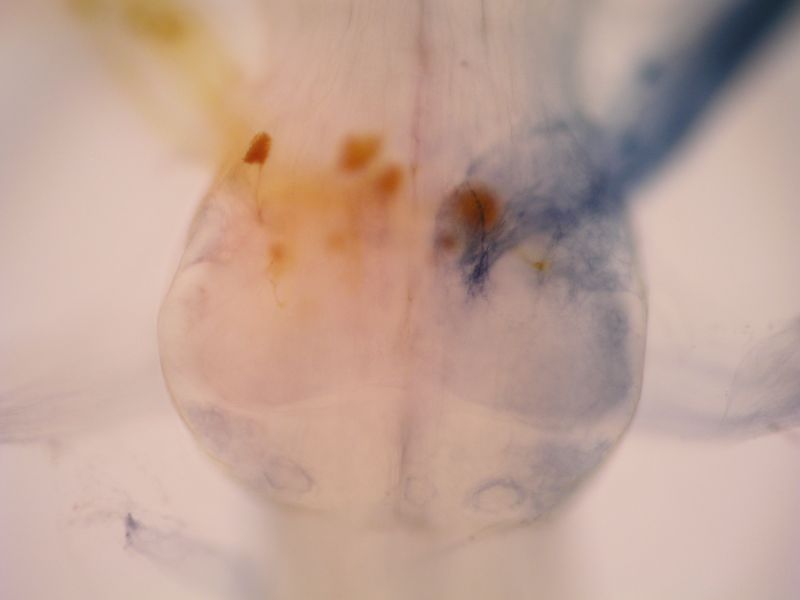

Supplement: Supplemental Information 2 — Micrographs of N2 backfills. Images have been reduced in size. [file peerj-03-1112-s002.zip › A1N2 2008 07 08 (10).jpg]

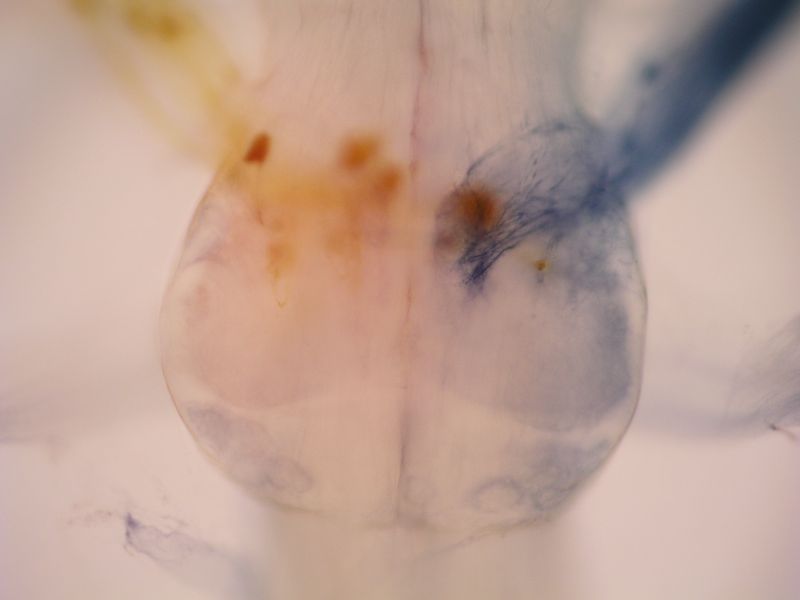

Supplement: Supplemental Information 2 — Micrographs of N2 backfills. Images have been reduced in size. [file peerj-03-1112-s002.zip › A1N2 2008 07 08 (11).jpg]

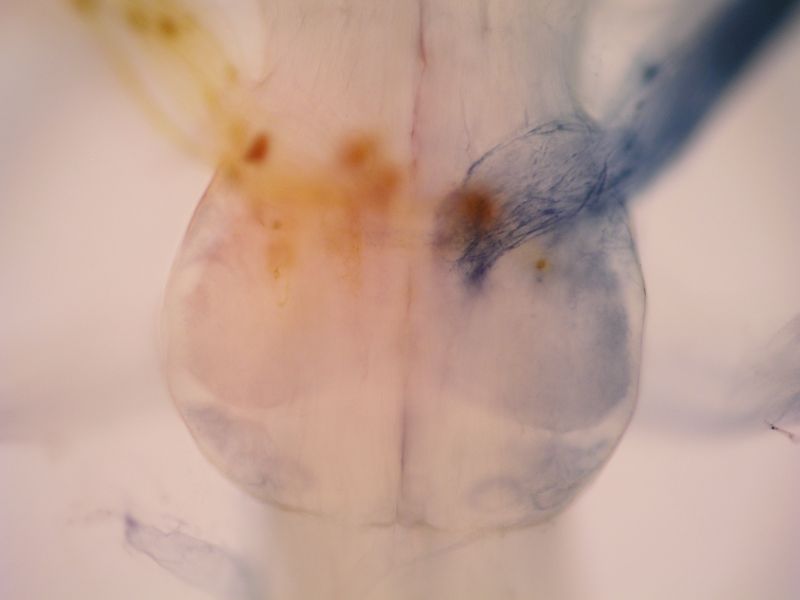

Supplement: Supplemental Information 2 — Micrographs of N2 backfills. Images have been reduced in size. [file peerj-03-1112-s002.zip › A1N2 2008 07 08 (12).jpg]

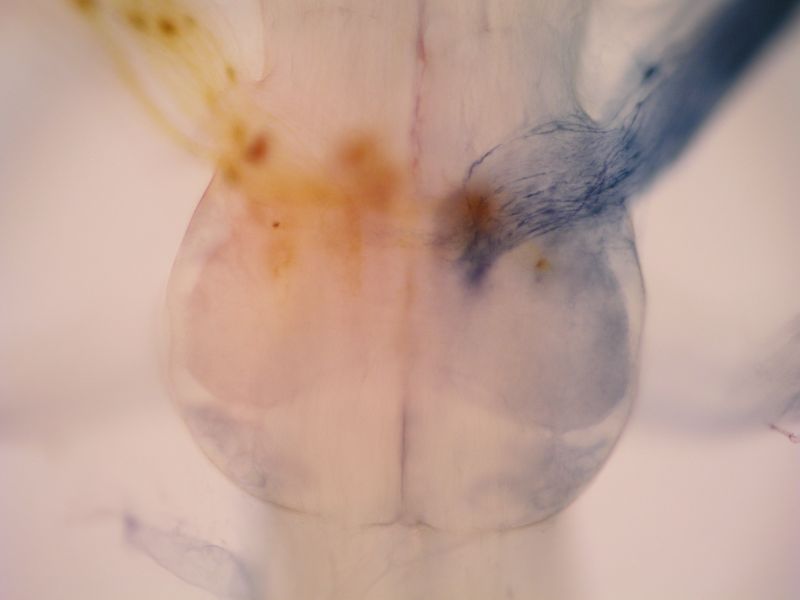

Supplement: Supplemental Information 2 — Micrographs of N2 backfills. Images have been reduced in size. [file peerj-03-1112-s002.zip › A1N2 2008 07 08 (13).jpg]

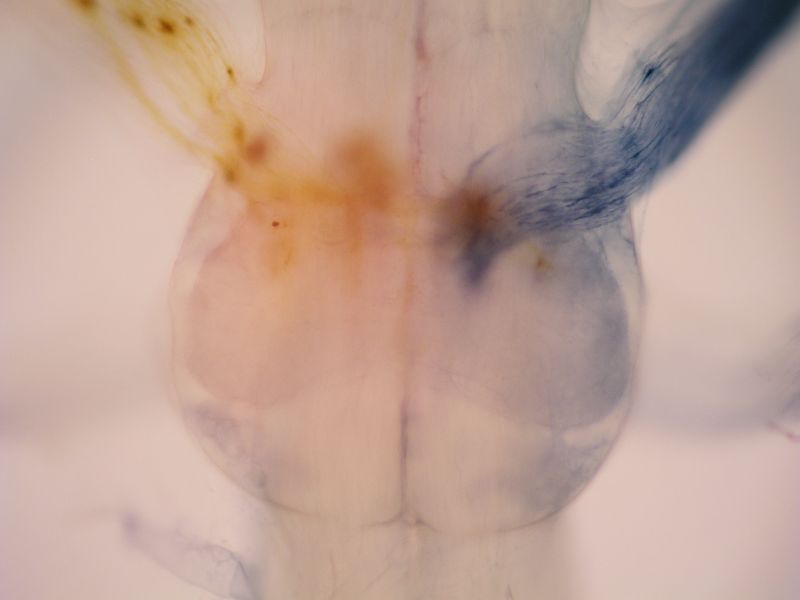

Supplement: Supplemental Information 2 — Micrographs of N2 backfills. Images have been reduced in size. [file peerj-03-1112-s002.zip › A1N2 2008 07 08 (14).jpg]

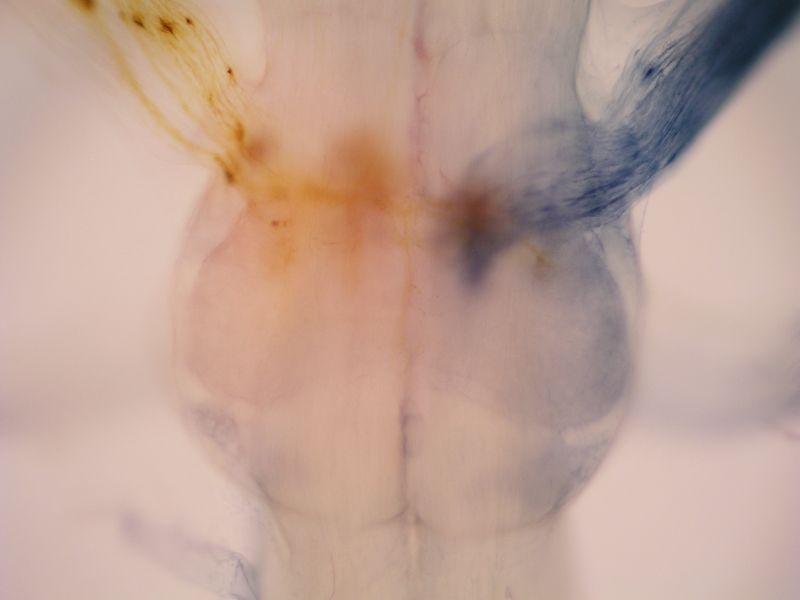

Supplement: Supplemental Information 2 — Micrographs of N2 backfills. Images have been reduced in size. [file peerj-03-1112-s002.zip › A1N2 2008 07 08 (15).jpg]

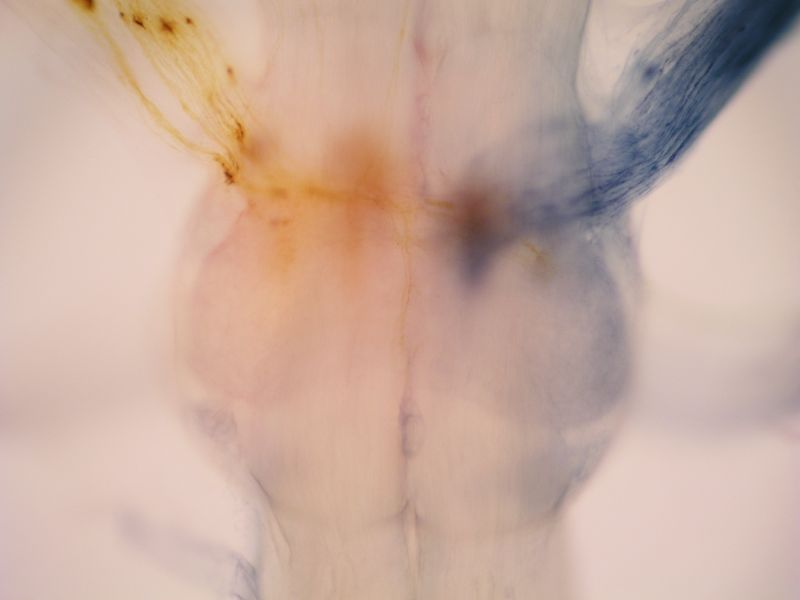

Supplement: Supplemental Information 2 — Micrographs of N2 backfills. Images have been reduced in size. [file peerj-03-1112-s002.zip › A1N2 2008 07 08 (16).jpg]

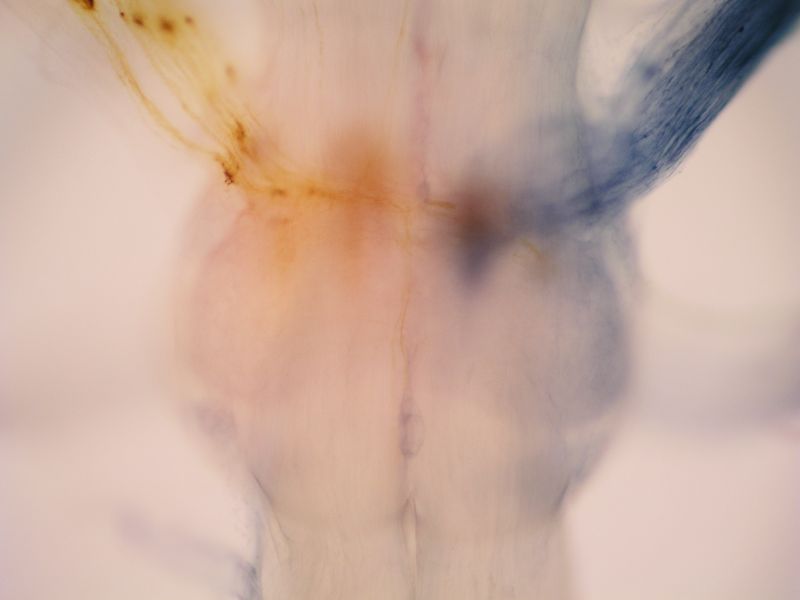

Supplement: Supplemental Information 2 — Micrographs of N2 backfills. Images have been reduced in size. [file peerj-03-1112-s002.zip › A1N2 2008 07 08 (17).jpg]

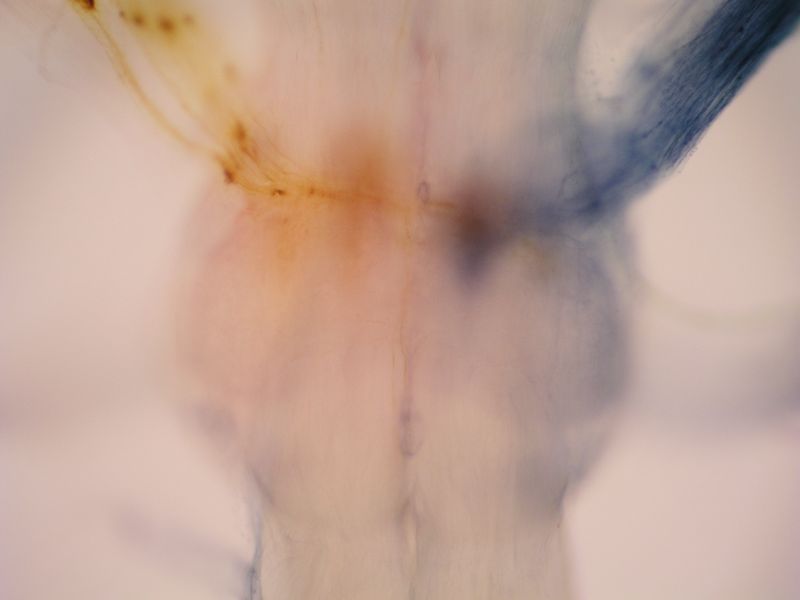

Supplement: Supplemental Information 2 — Micrographs of N2 backfills. Images have been reduced in size. [file peerj-03-1112-s002.zip › A1N2 2008 07 08 (18).jpg]

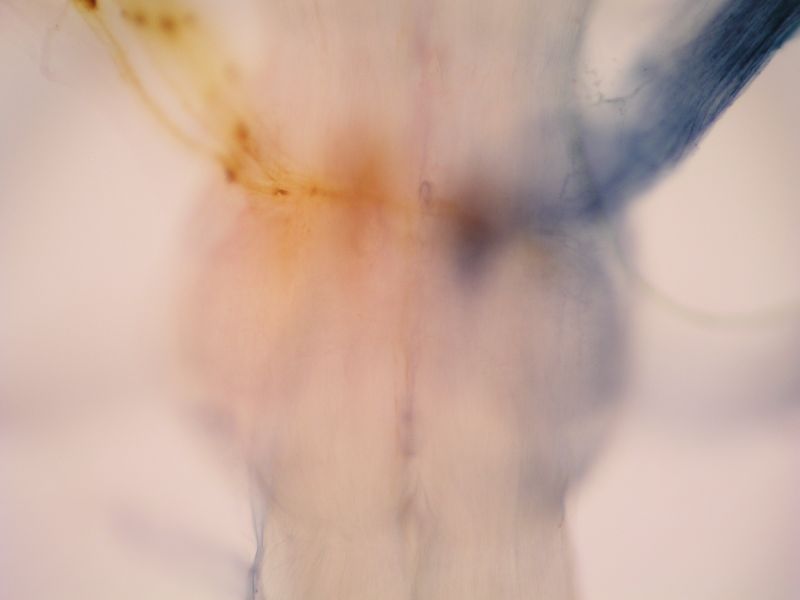

Supplement: Supplemental Information 2 — Micrographs of N2 backfills. Images have been reduced in size. [file peerj-03-1112-s002.zip › A1N2 2008 07 08 (19).jpg]

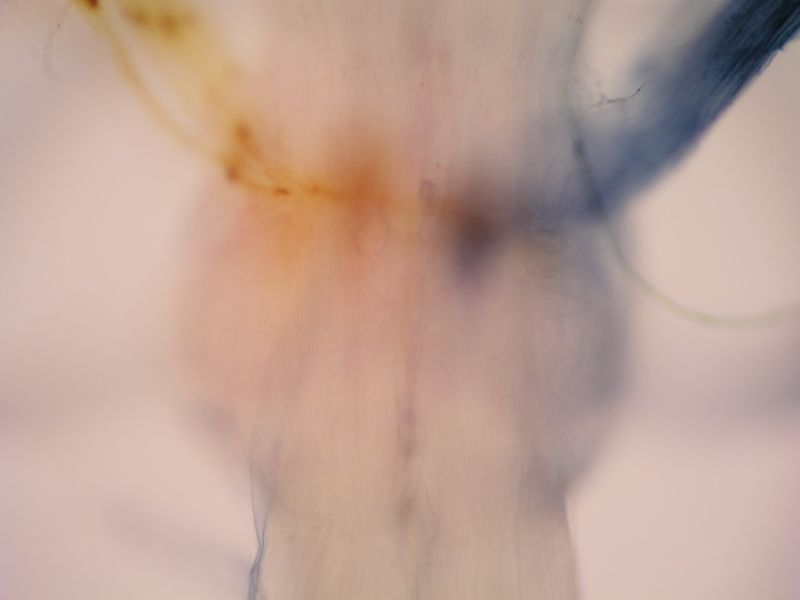

Supplement: Supplemental Information 2 — Micrographs of N2 backfills. Images have been reduced in size. [file peerj-03-1112-s002.zip › A1N2 2008 07 08 (20).jpg]

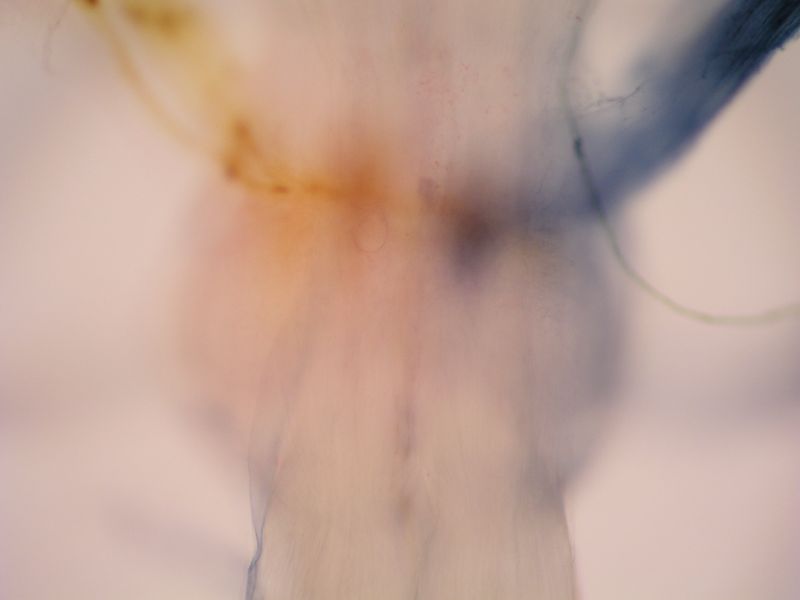

Supplement: Supplemental Information 2 — Micrographs of N2 backfills. Images have been reduced in size. [file peerj-03-1112-s002.zip › A1N2 2008 07 08.jpg]

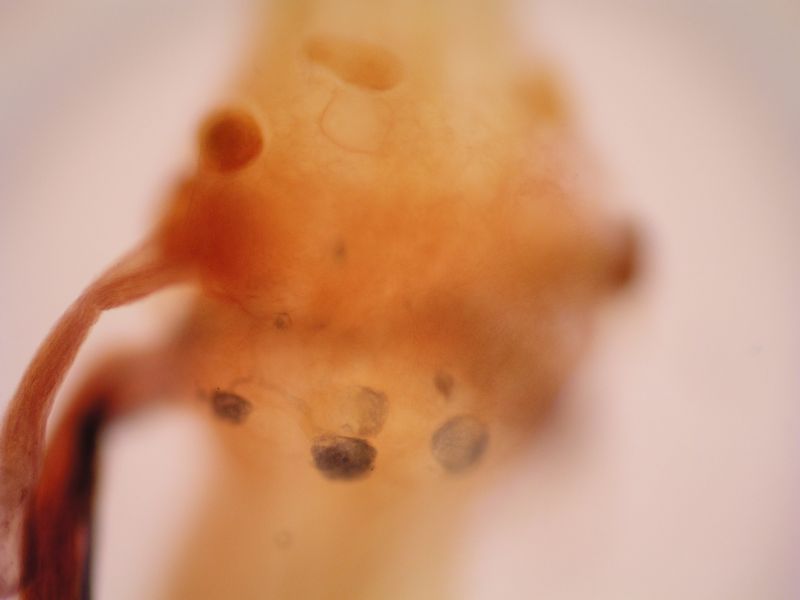

Supplement: Supplemental Information 2 — Micrographs of N2 backfills. Images have been reduced in size. [file peerj-03-1112-s002.zip › A1N2 2009 07 04 a.jpg]

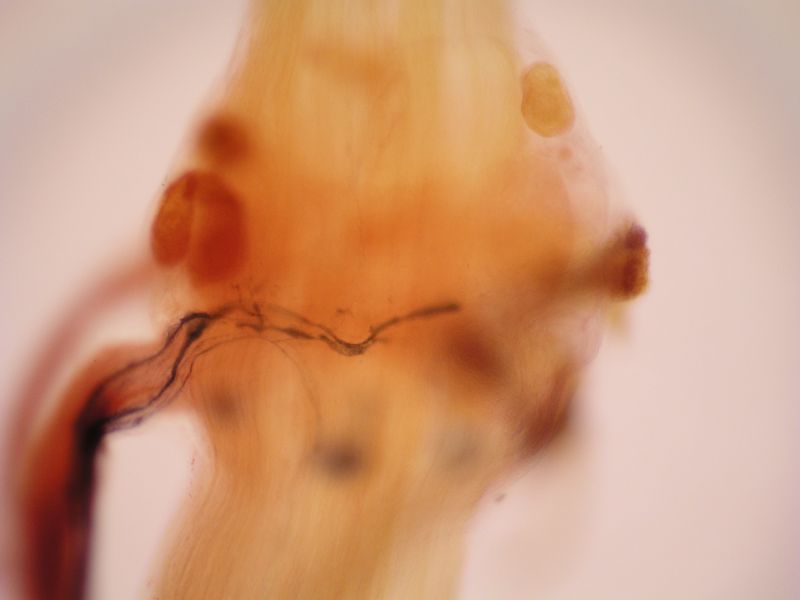

Supplement: Supplemental Information 2 — Micrographs of N2 backfills. Images have been reduced in size. [file peerj-03-1112-s002.zip › A1N2 2009 07 04 b.jpg]

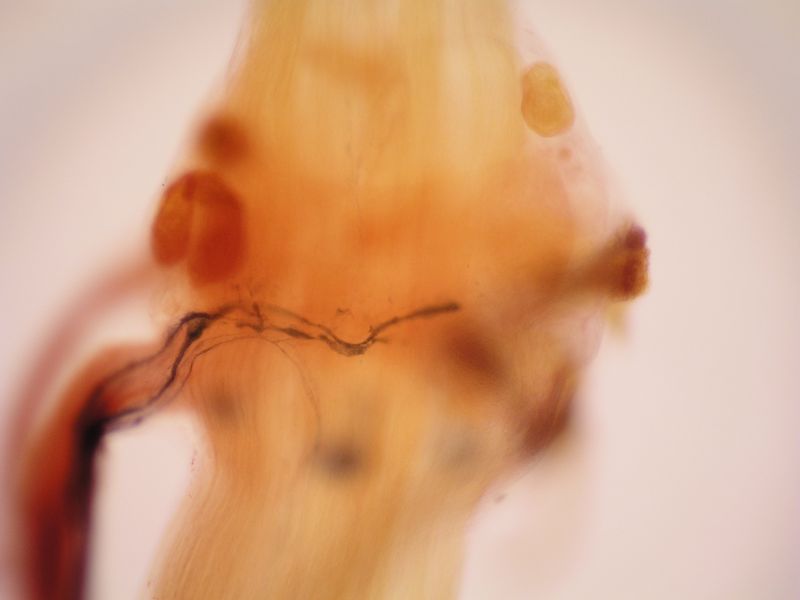

Supplement: Supplemental Information 2 — Micrographs of N2 backfills. Images have been reduced in size. [file peerj-03-1112-s002.zip › A1N2 2009 07 04 c.jpg]

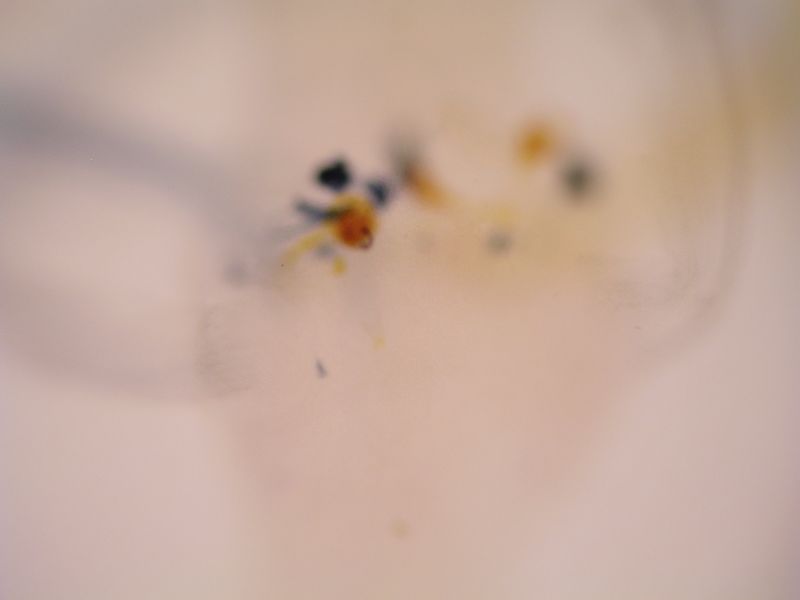

Supplement: Supplemental Information 2 — Micrographs of N2 backfills. Images have been reduced in size. [file peerj-03-1112-s002.zip › A1N2 2009 07 07 b stack (1).jpg]

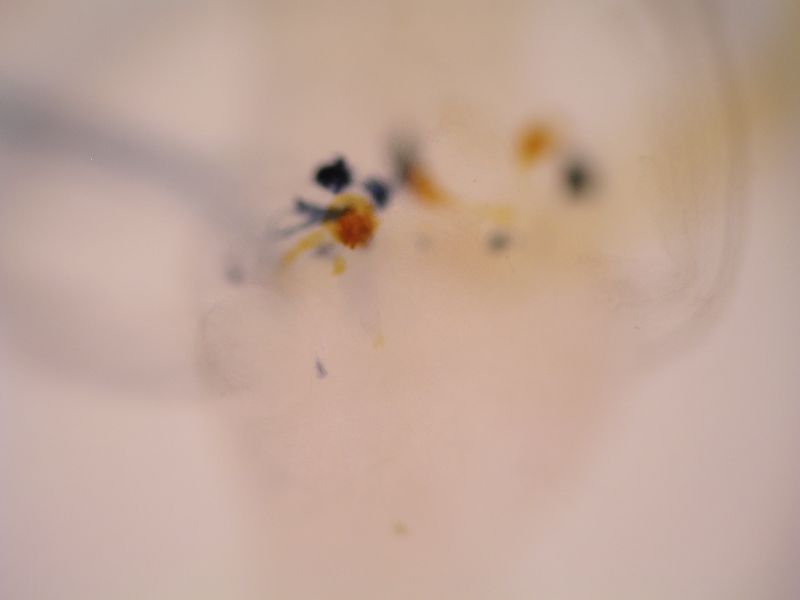

Supplement: Supplemental Information 2 — Micrographs of N2 backfills. Images have been reduced in size. [file peerj-03-1112-s002.zip › A1N2 2009 07 07 b stack (2).jpg]

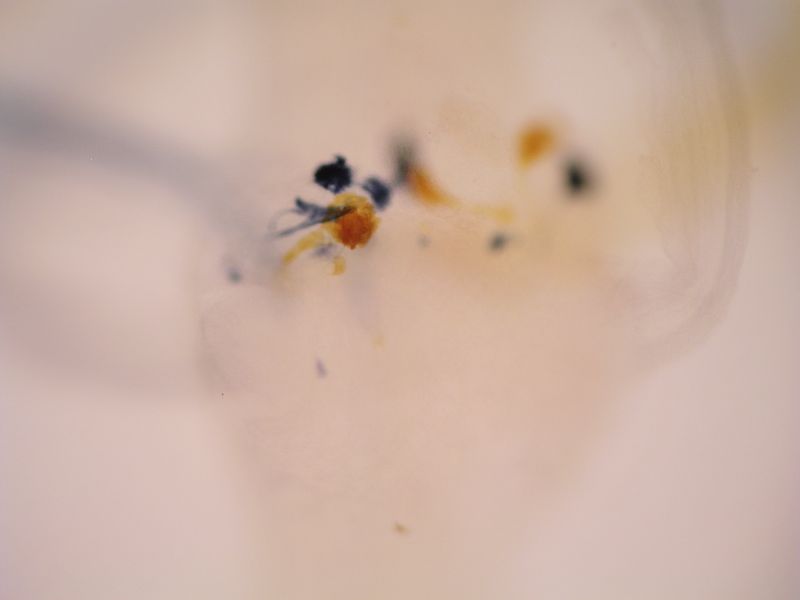

Supplement: Supplemental Information 2 — Micrographs of N2 backfills. Images have been reduced in size. [file peerj-03-1112-s002.zip › A1N2 2009 07 07 b stack (3).jpg]

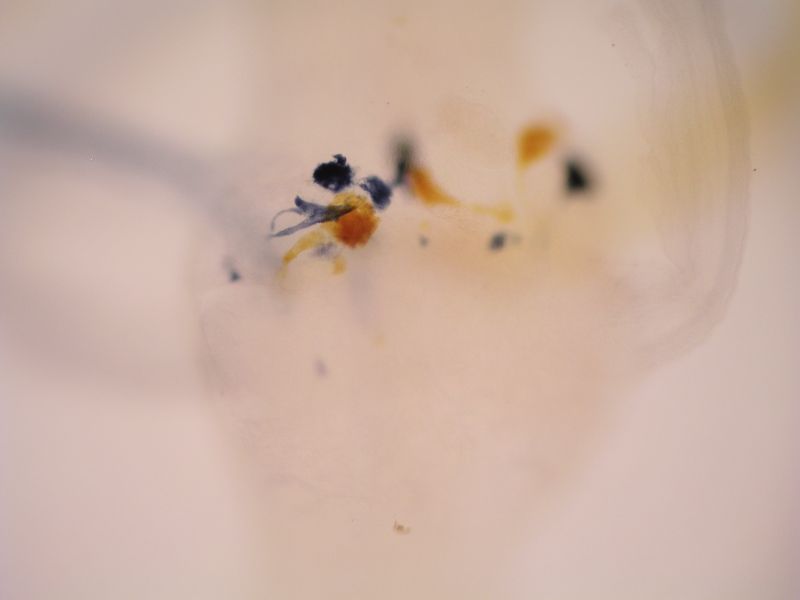

Supplement: Supplemental Information 2 — Micrographs of N2 backfills. Images have been reduced in size. [file peerj-03-1112-s002.zip › A1N2 2009 07 07 b stack (4).jpg]

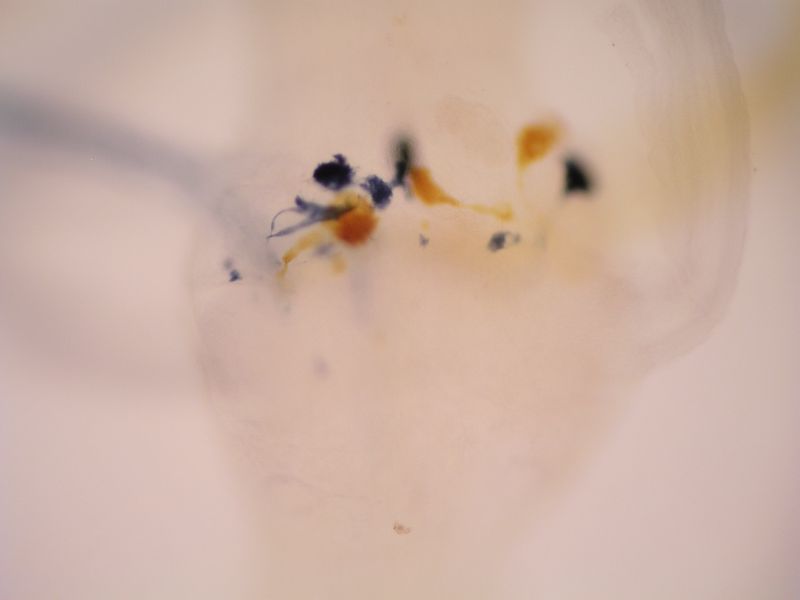

Supplement: Supplemental Information 2 — Micrographs of N2 backfills. Images have been reduced in size. [file peerj-03-1112-s002.zip › A1N2 2009 07 07 b stack (5).jpg]

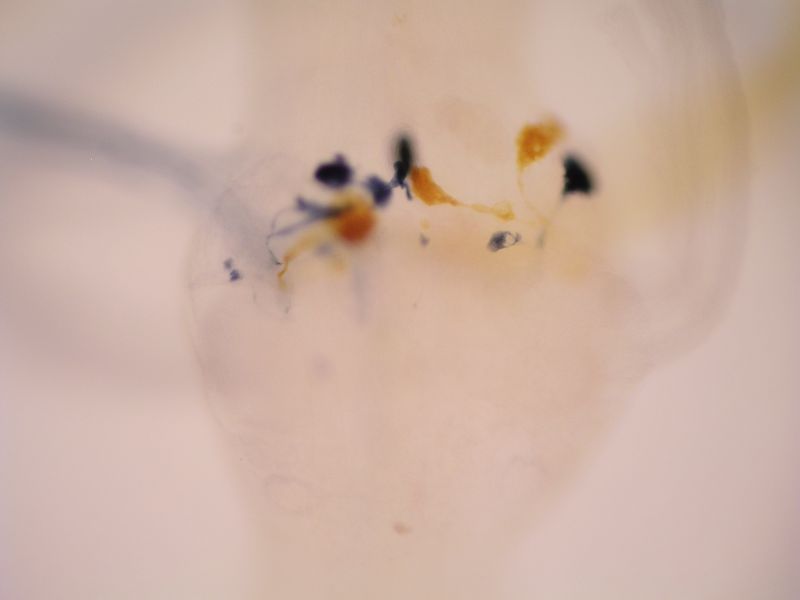

Supplement: Supplemental Information 2 — Micrographs of N2 backfills. Images have been reduced in size. [file peerj-03-1112-s002.zip › A1N2 2009 07 07 b stack (6).jpg]

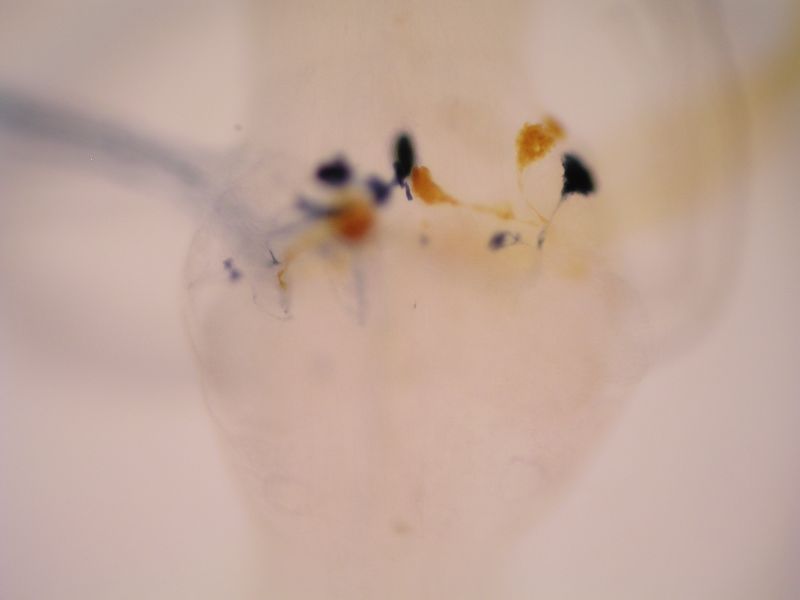

Supplement: Supplemental Information 2 — Micrographs of N2 backfills. Images have been reduced in size. [file peerj-03-1112-s002.zip › A1N2 2009 07 07 b stack (7).jpg]

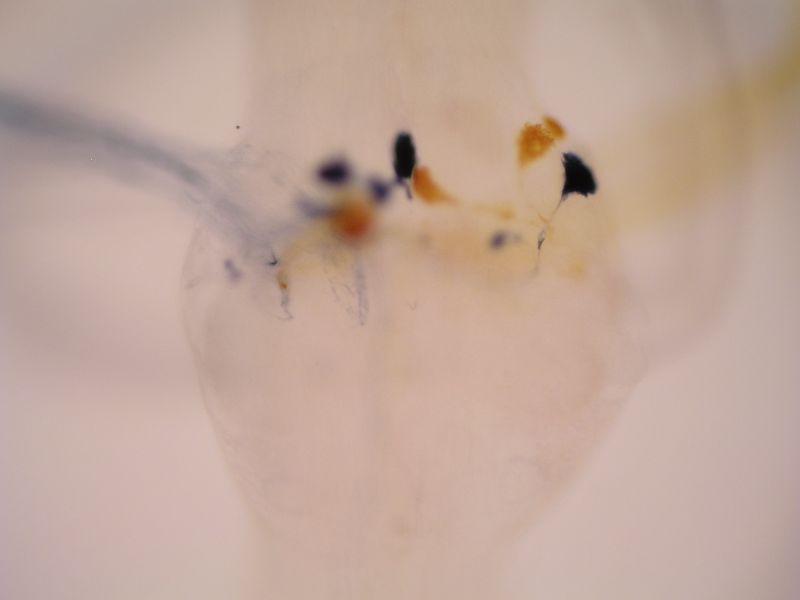

Supplement: Supplemental Information 2 — Micrographs of N2 backfills. Images have been reduced in size. [file peerj-03-1112-s002.zip › A1N2 2009 07 07 b stack (8).jpg]

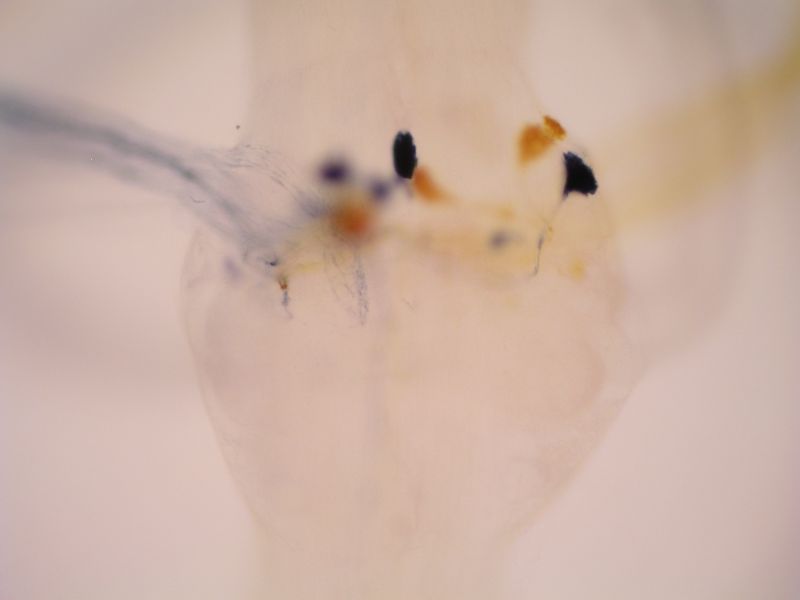

Supplement: Supplemental Information 2 — Micrographs of N2 backfills. Images have been reduced in size. [file peerj-03-1112-s002.zip › A1N2 2009 07 07 b stack (9).jpg]

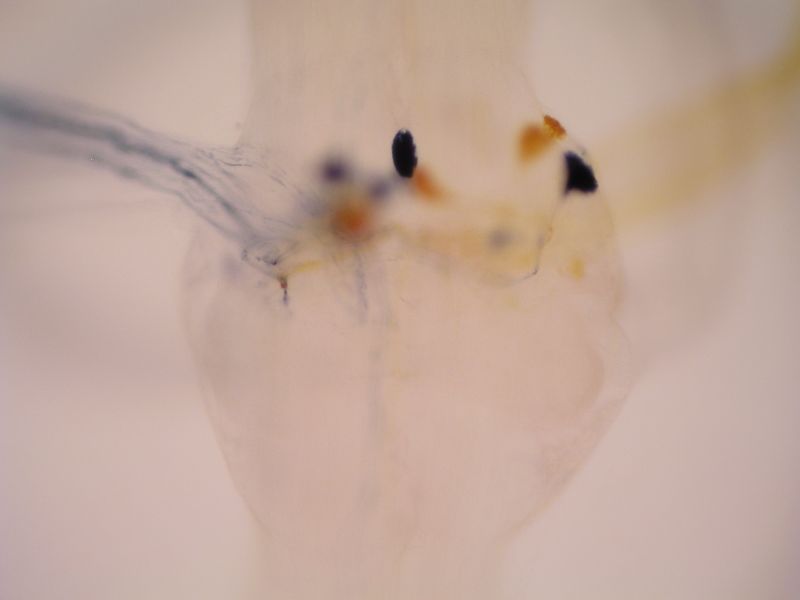

Supplement: Supplemental Information 2 — Micrographs of N2 backfills. Images have been reduced in size. [file peerj-03-1112-s002.zip › A1N2 2009 07 07 b stack (10).jpg]

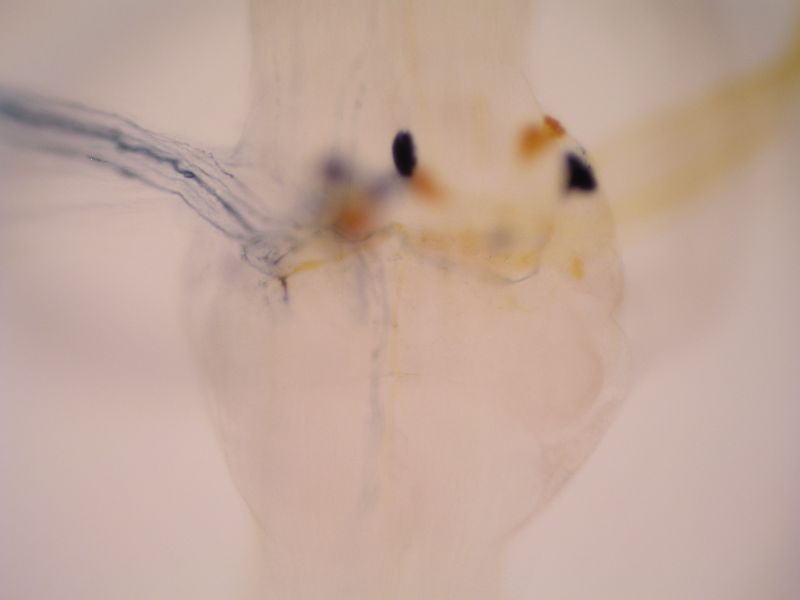

Supplement: Supplemental Information 2 — Micrographs of N2 backfills. Images have been reduced in size. [file peerj-03-1112-s002.zip › A1N2 2009 07 07 b stack (11).jpg]

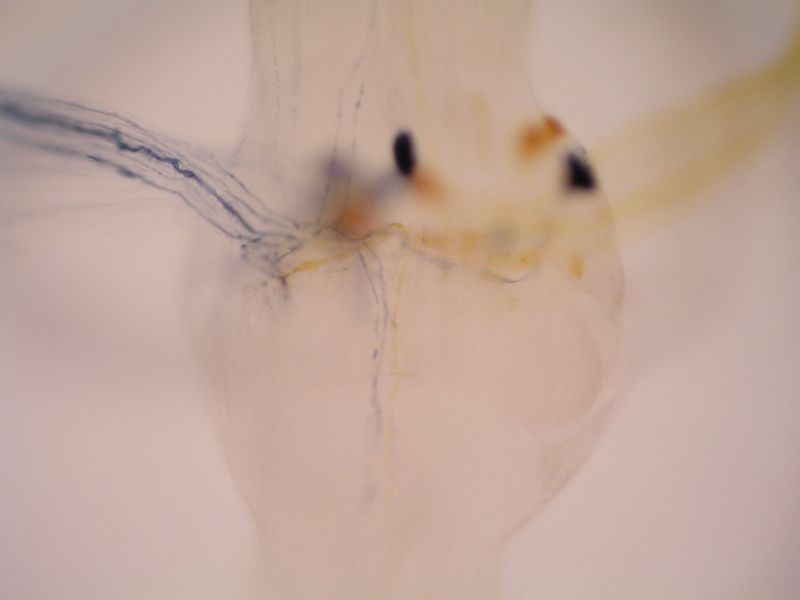

Supplement: Supplemental Information 2 — Micrographs of N2 backfills. Images have been reduced in size. [file peerj-03-1112-s002.zip › A1N2 2009 07 07 b stack (12).jpg]

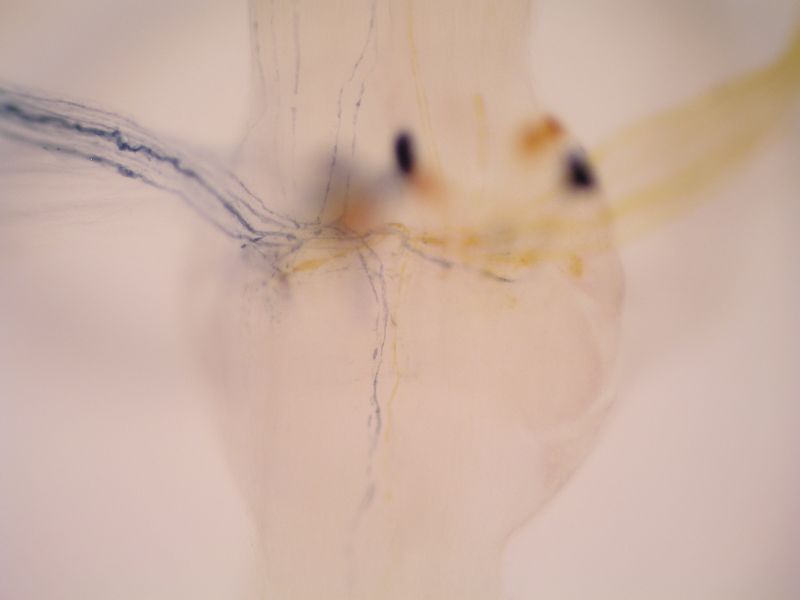

Supplement: Supplemental Information 2 — Micrographs of N2 backfills. Images have been reduced in size. [file peerj-03-1112-s002.zip › A1N2 2009 07 07 b stack (13).jpg]

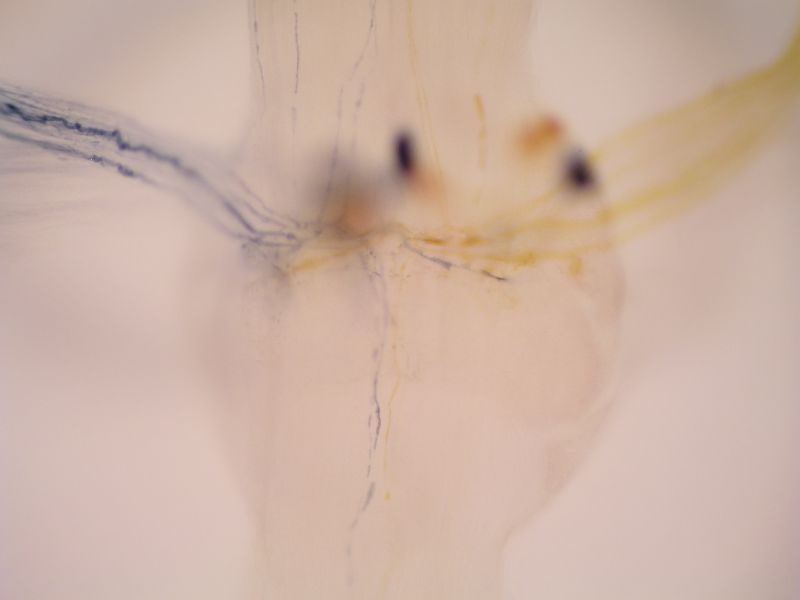

Supplement: Supplemental Information 2 — Micrographs of N2 backfills. Images have been reduced in size. [file peerj-03-1112-s002.zip › A1N2 2009 07 07 b stack (14).jpg]

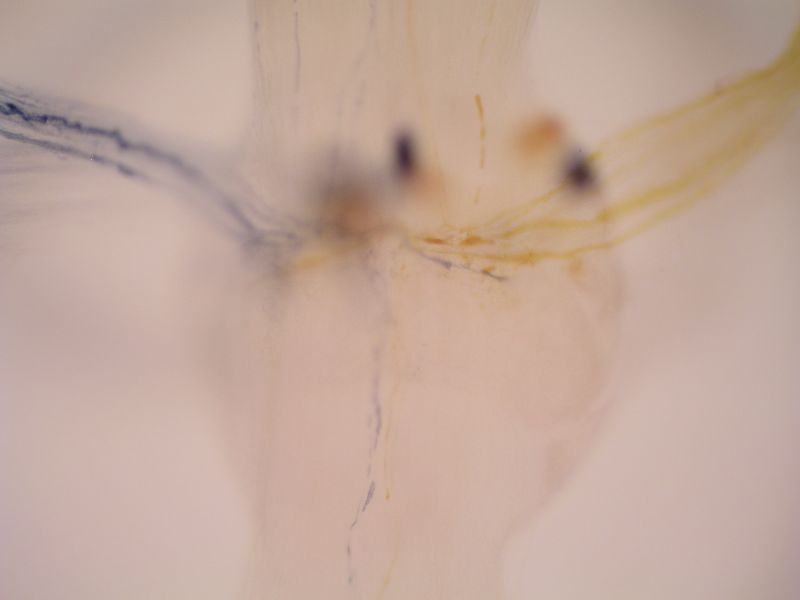

Supplement: Supplemental Information 2 — Micrographs of N2 backfills. Images have been reduced in size. [file peerj-03-1112-s002.zip › A1N2 2009 07 07 b stack (15).jpg]

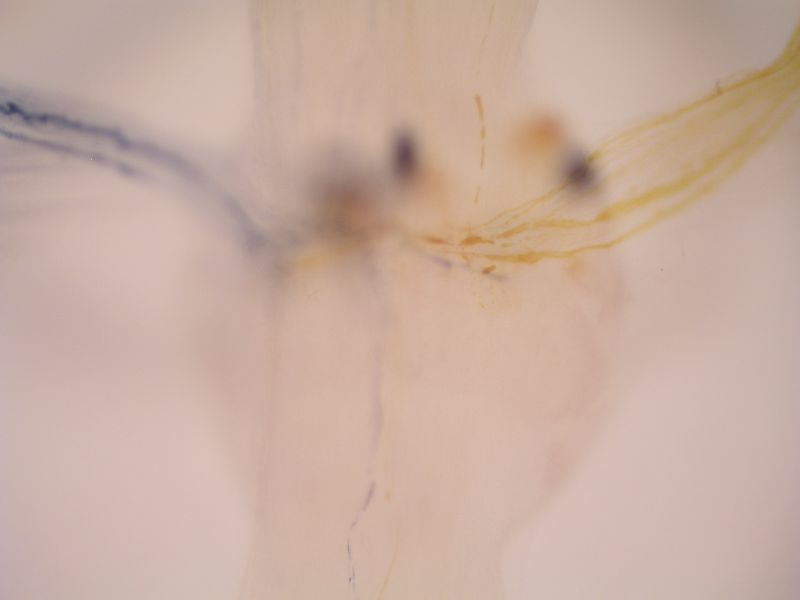

Supplement: Supplemental Information 2 — Micrographs of N2 backfills. Images have been reduced in size. [file peerj-03-1112-s002.zip › A1N2 2009 07 07 b stack (16).jpg]

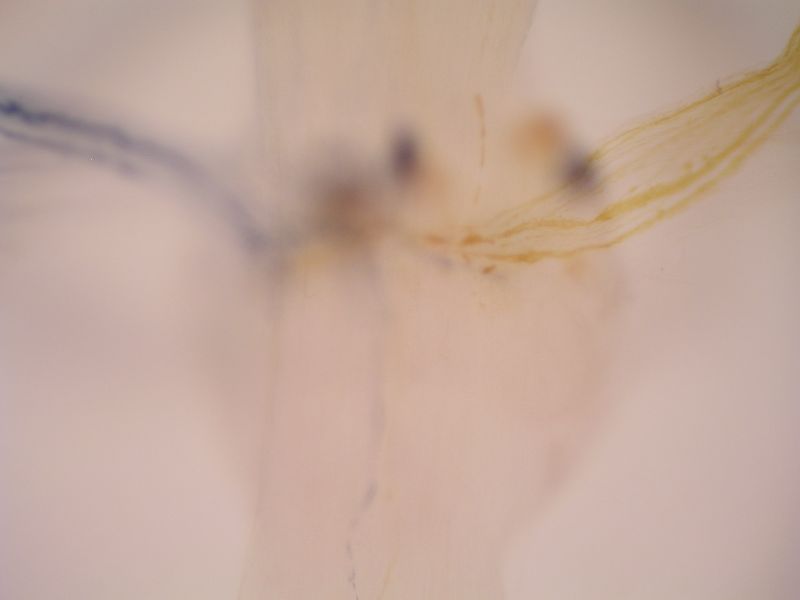

Supplement: Supplemental Information 2 — Micrographs of N2 backfills. Images have been reduced in size. [file peerj-03-1112-s002.zip › A1N2 2009 07 07 b stack (17).jpg]

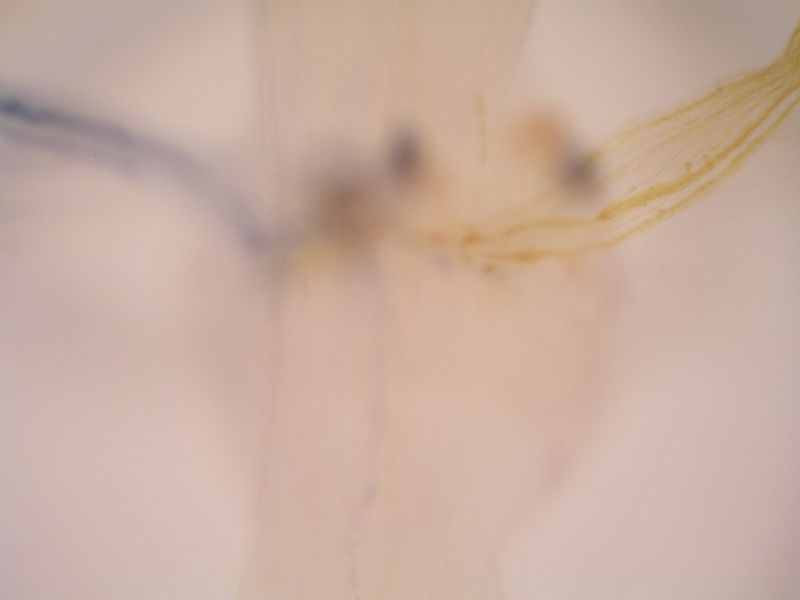

Supplement: Supplemental Information 2 — Micrographs of N2 backfills. Images have been reduced in size. [file peerj-03-1112-s002.zip › A1N2 2009 07 07 b stack (18).jpg]

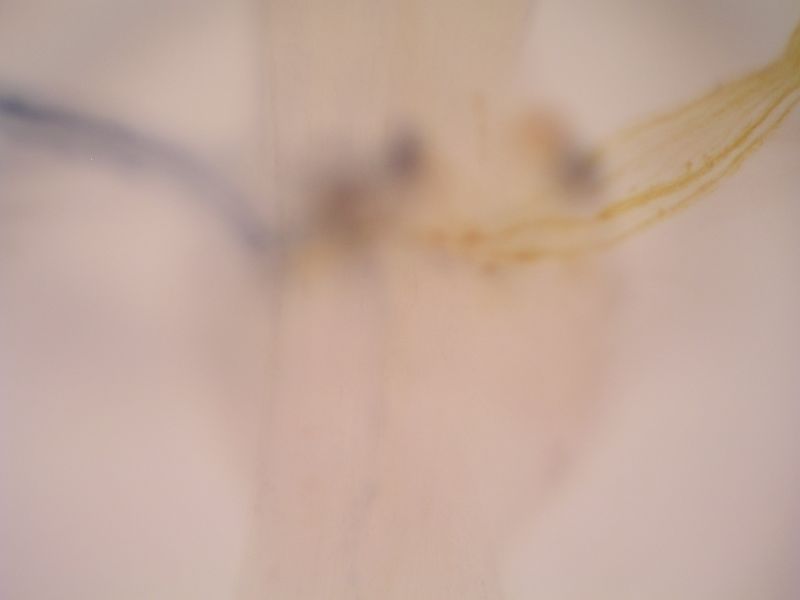

Supplement: Supplemental Information 2 — Micrographs of N2 backfills. Images have been reduced in size. [file peerj-03-1112-s002.zip › A1N2 2009 07 07 b stack (19).jpg]

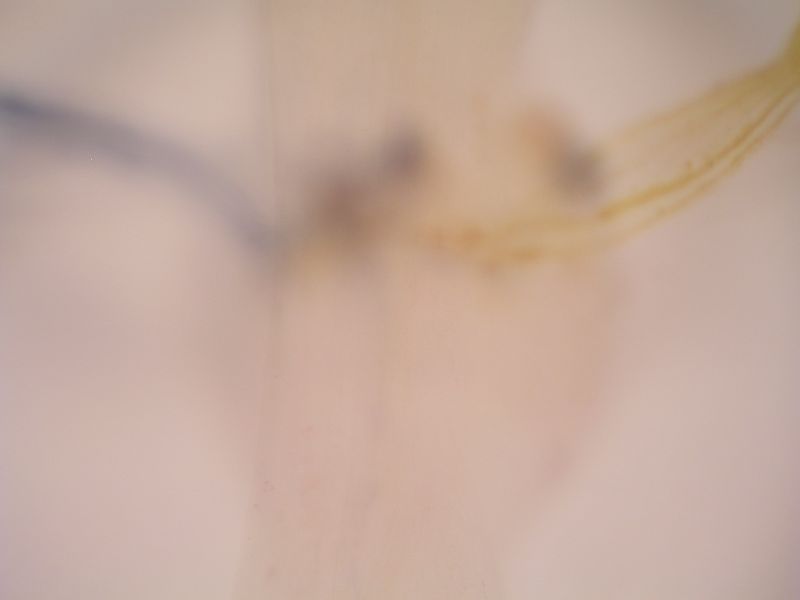

Supplement: Supplemental Information 2 — Micrographs of N2 backfills. Images have been reduced in size. [file peerj-03-1112-s002.zip › A1N2 2009 07 07 b stack (20).jpg]

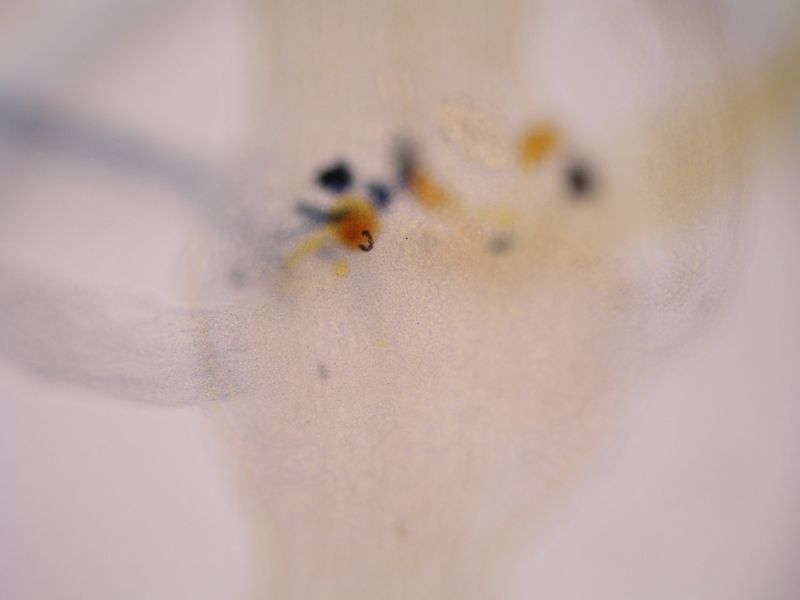

Supplement: Supplemental Information 2 — Micrographs of N2 backfills. Images have been reduced in size. [file peerj-03-1112-s002.zip › A1N2 2009 07 07 stack a (1).jpg]

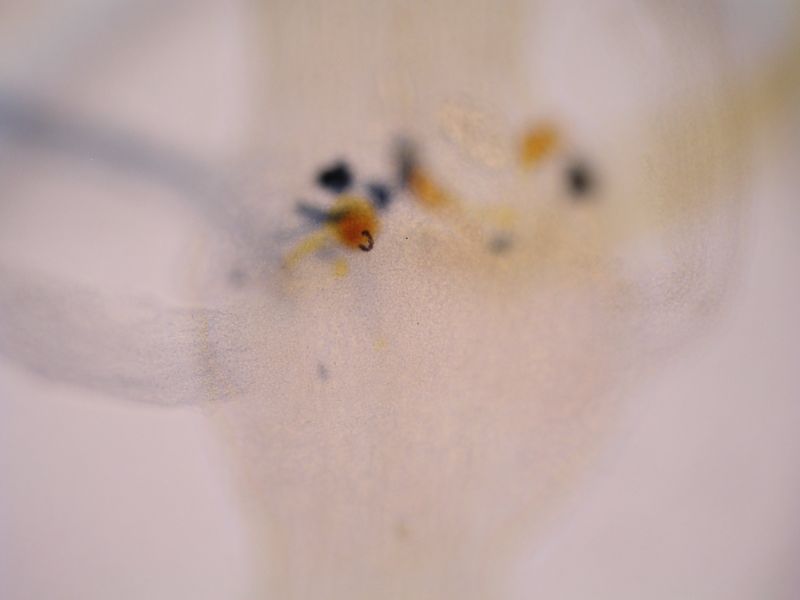

Supplement: Supplemental Information 2 — Micrographs of N2 backfills. Images have been reduced in size. [file peerj-03-1112-s002.zip › A1N2 2009 07 07 stack a (2).jpg]

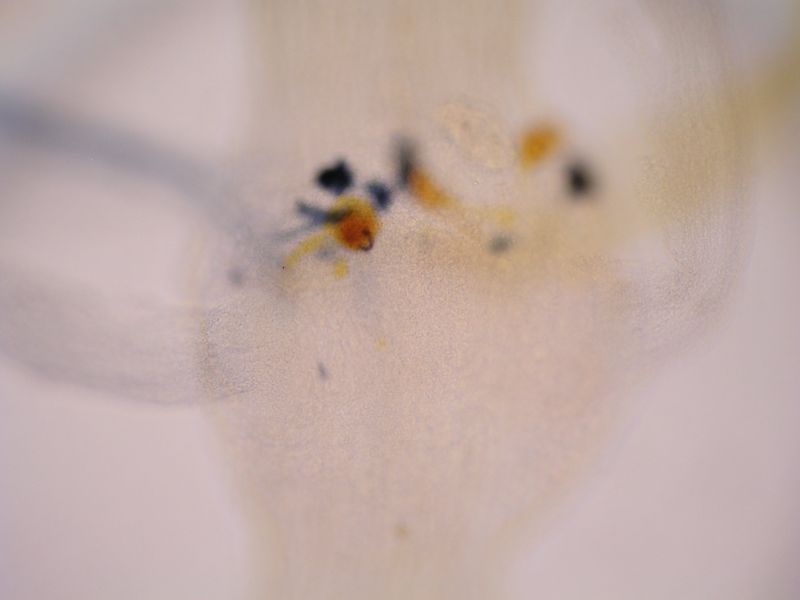

Supplement: Supplemental Information 2 — Micrographs of N2 backfills. Images have been reduced in size. [file peerj-03-1112-s002.zip › A1N2 2009 07 07 stack a (3).jpg]

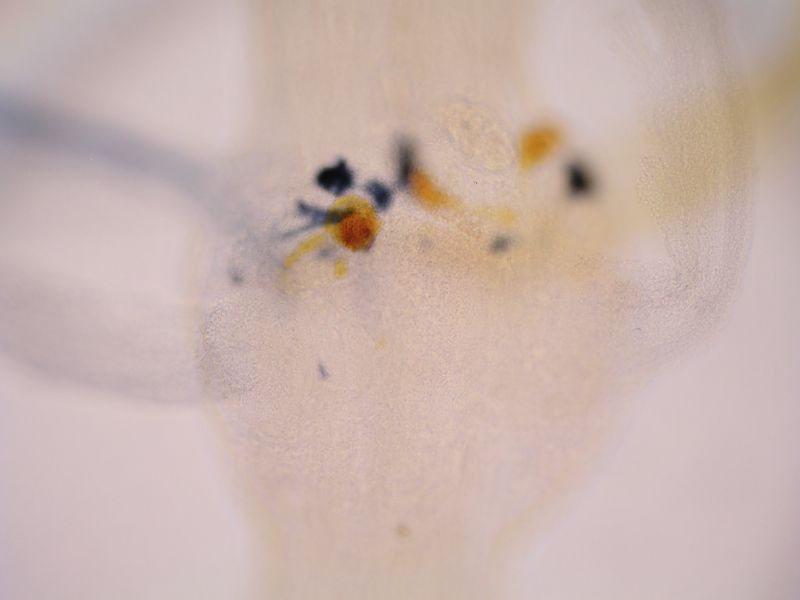

Supplement: Supplemental Information 2 — Micrographs of N2 backfills. Images have been reduced in size. [file peerj-03-1112-s002.zip › A1N2 2009 07 07 stack a (4).jpg]

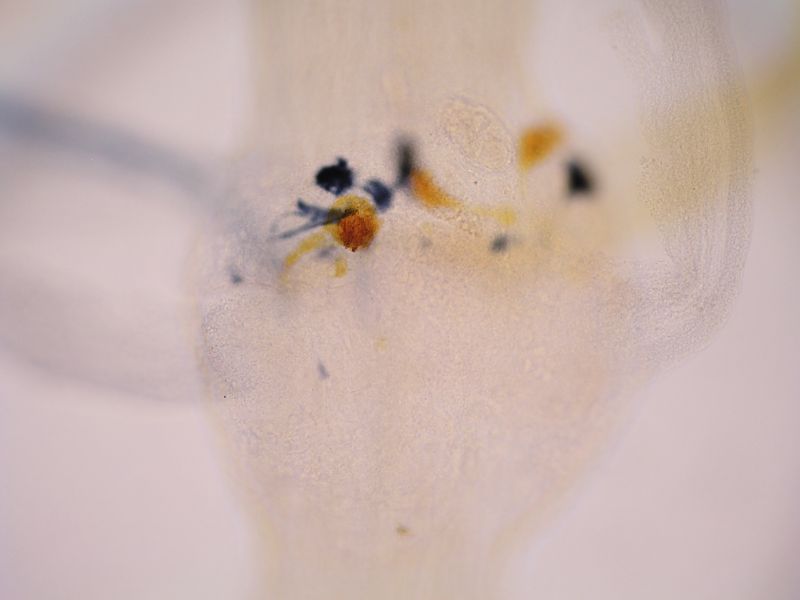

Supplement: Supplemental Information 2 — Micrographs of N2 backfills. Images have been reduced in size. [file peerj-03-1112-s002.zip › A1N2 2009 07 07 stack a (5).jpg]

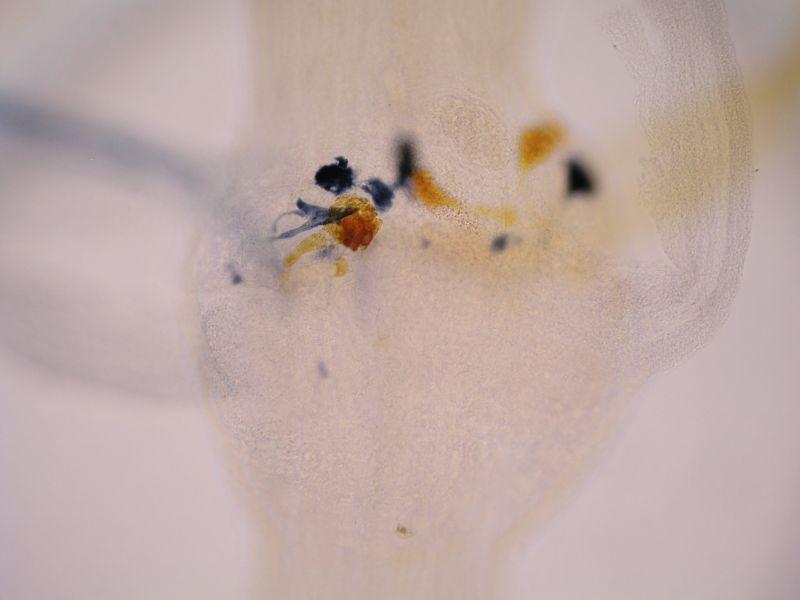

Supplement: Supplemental Information 2 — Micrographs of N2 backfills. Images have been reduced in size. [file peerj-03-1112-s002.zip › A1N2 2009 07 07 stack a (6).jpg]

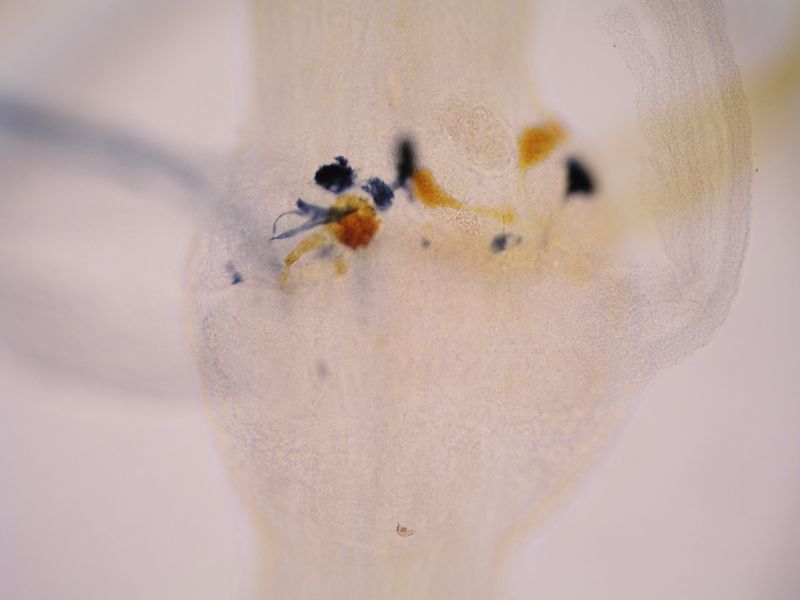

Supplement: Supplemental Information 2 — Micrographs of N2 backfills. Images have been reduced in size. [file peerj-03-1112-s002.zip › A1N2 2009 07 07 stack a (7).jpg]

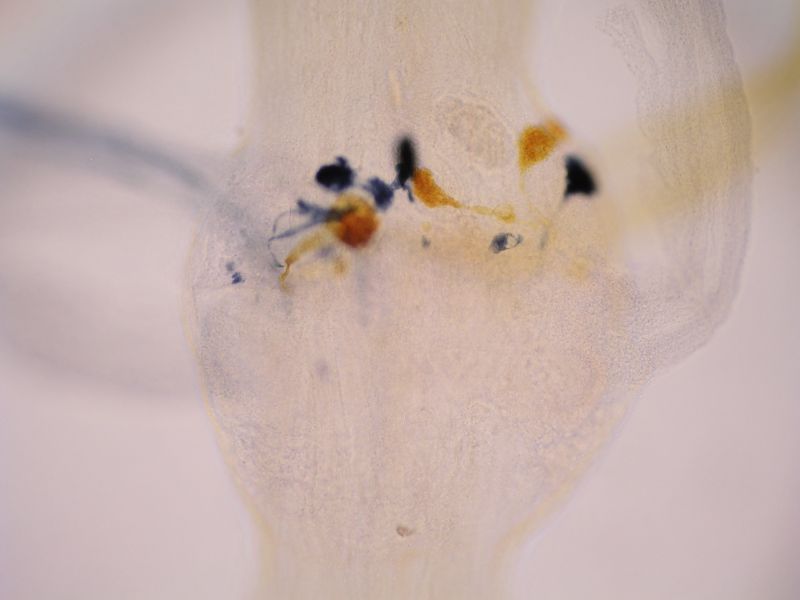

Supplement: Supplemental Information 2 — Micrographs of N2 backfills. Images have been reduced in size. [file peerj-03-1112-s002.zip › A1N2 2009 07 07 stack a (8).jpg]

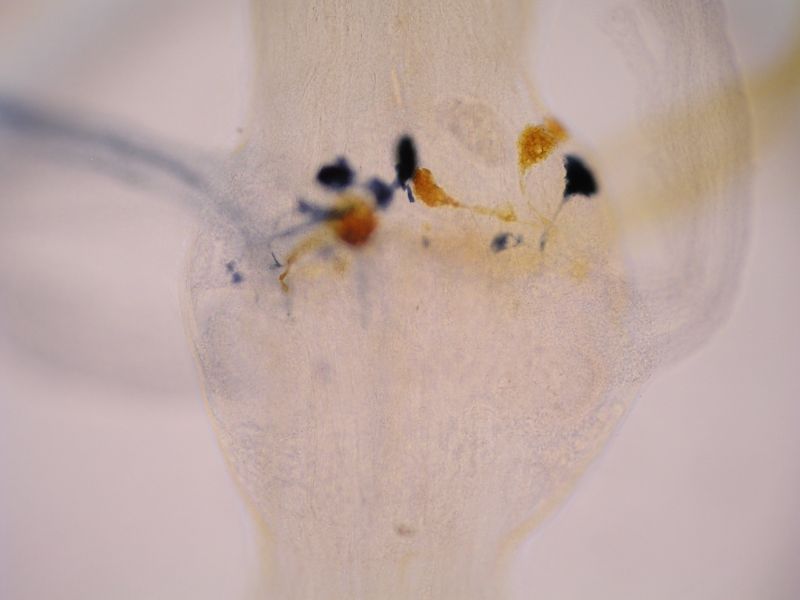

Supplement: Supplemental Information 2 — Micrographs of N2 backfills. Images have been reduced in size. [file peerj-03-1112-s002.zip › A1N2 2009 07 07 stack a (9).jpg]

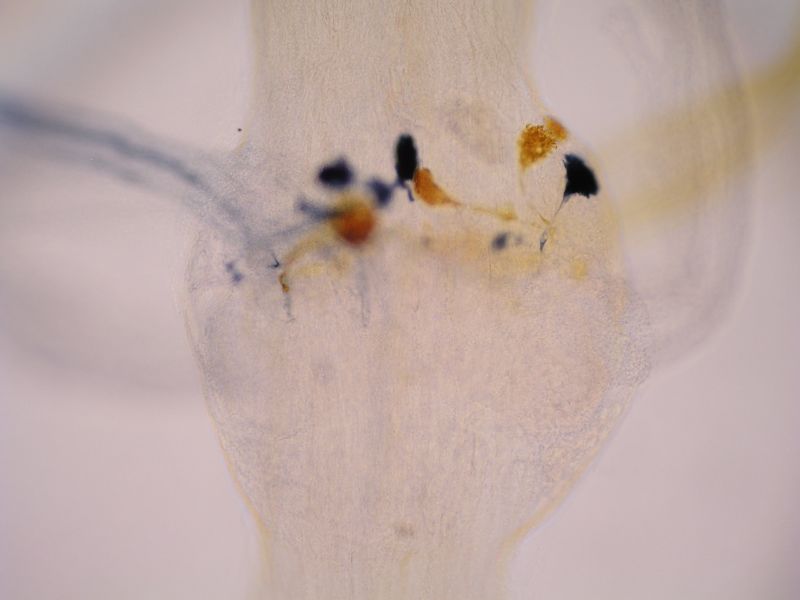

Supplement: Supplemental Information 2 — Micrographs of N2 backfills. Images have been reduced in size. [file peerj-03-1112-s002.zip › A1N2 2009 07 07 stack a (10).jpg]

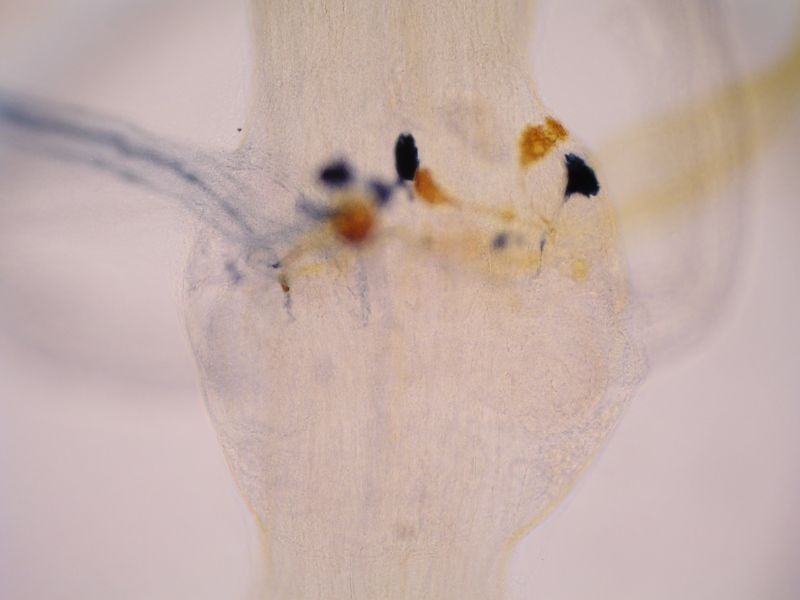

Supplement: Supplemental Information 2 — Micrographs of N2 backfills. Images have been reduced in size. [file peerj-03-1112-s002.zip › A1N2 2009 07 07 stack a (11).jpg]

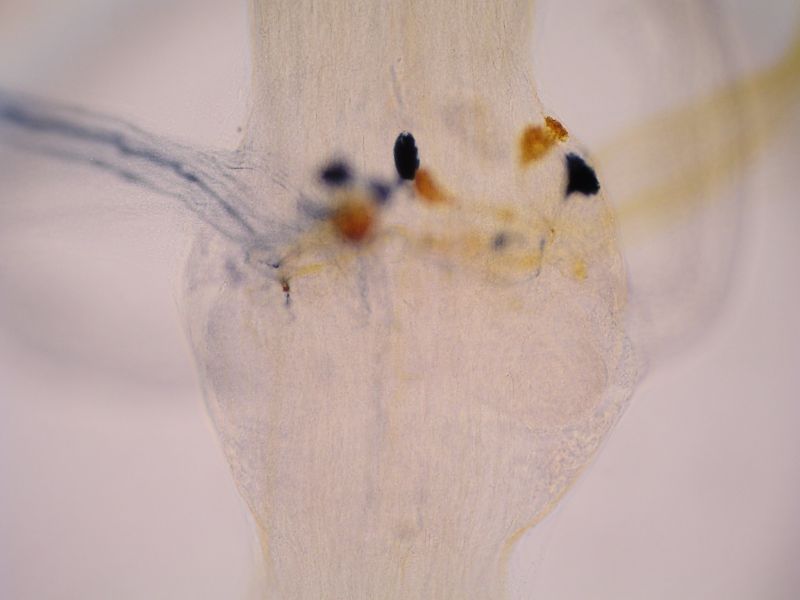

Supplement: Supplemental Information 2 — Micrographs of N2 backfills. Images have been reduced in size. [file peerj-03-1112-s002.zip › A1N2 2009 07 07 stack a (12).jpg]

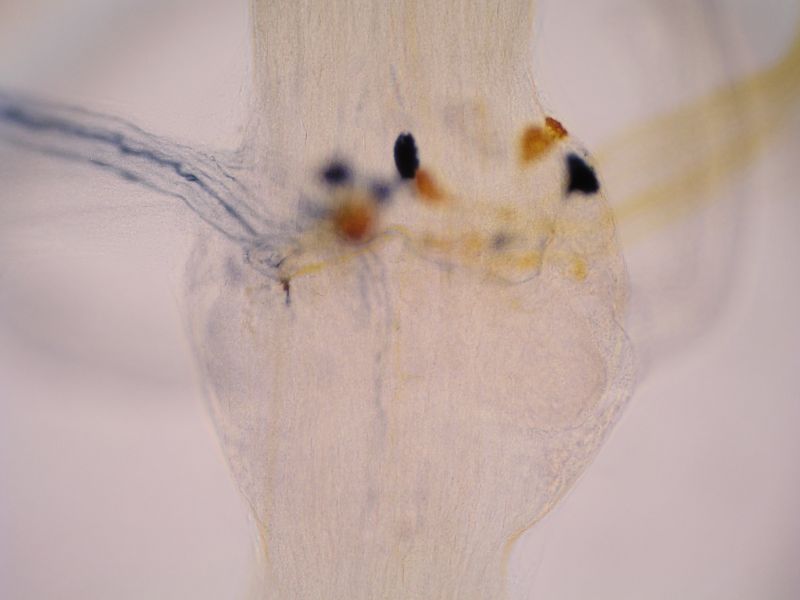

Supplement: Supplemental Information 2 — Micrographs of N2 backfills. Images have been reduced in size. [file peerj-03-1112-s002.zip › A1N2 2009 07 07 stack a (13).jpg]

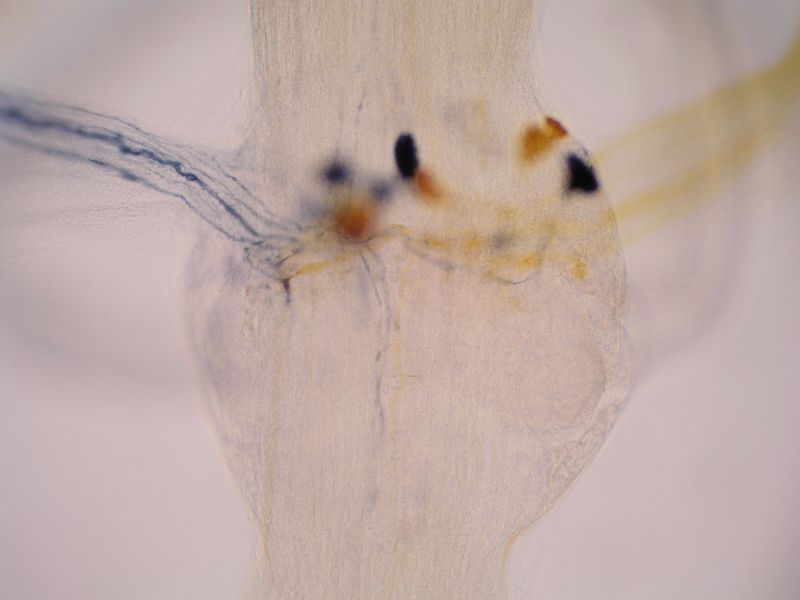

Supplement: Supplemental Information 2 — Micrographs of N2 backfills. Images have been reduced in size. [file peerj-03-1112-s002.zip › A1N2 2009 07 07 stack a (14).jpg]

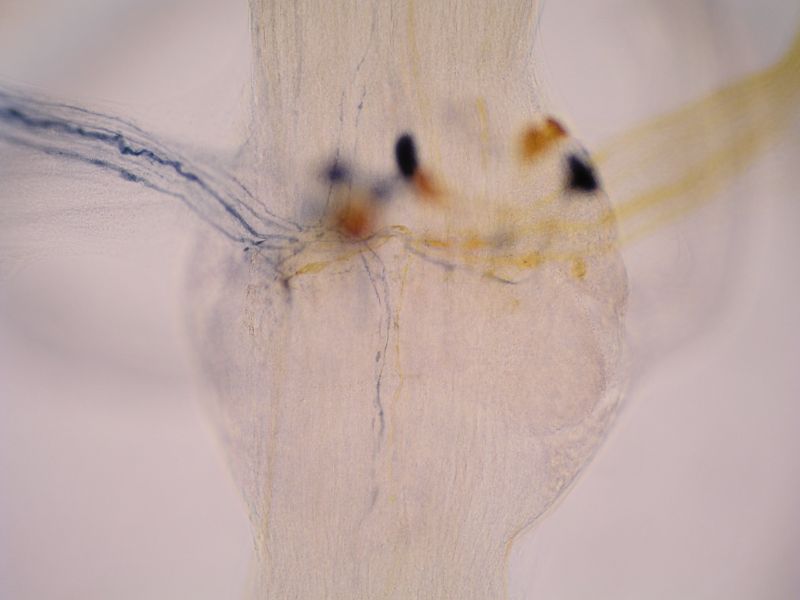

Supplement: Supplemental Information 2 — Micrographs of N2 backfills. Images have been reduced in size. [file peerj-03-1112-s002.zip › A1N2 2009 07 07 stack a (15).jpg]

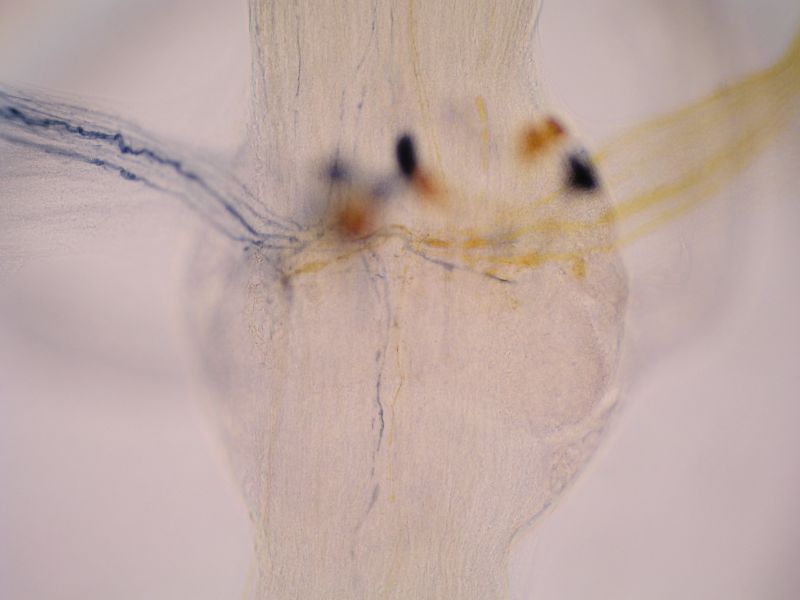

Supplement: Supplemental Information 2 — Micrographs of N2 backfills. Images have been reduced in size. [file peerj-03-1112-s002.zip › A1N2 2009 07 07 stack a (16).jpg]

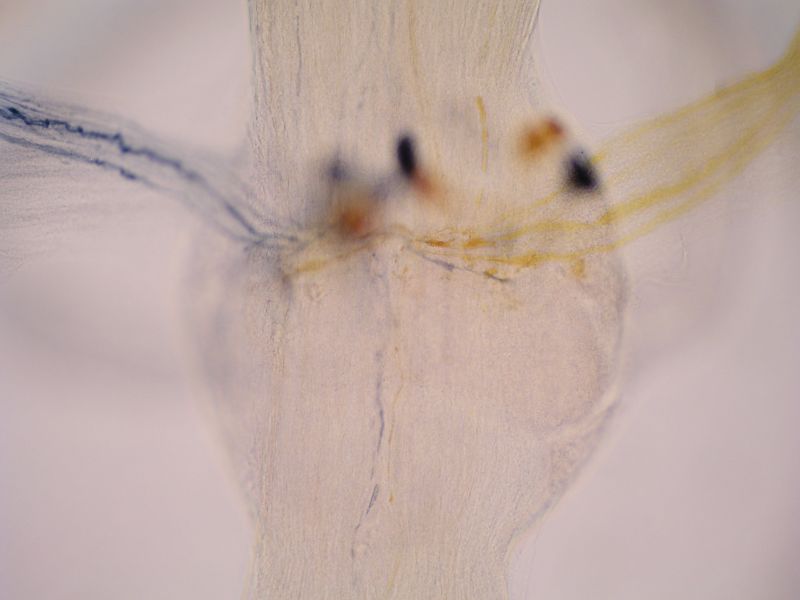

Supplement: Supplemental Information 2 — Micrographs of N2 backfills. Images have been reduced in size. [file peerj-03-1112-s002.zip › A1N2 2009 07 07 stack a (17).jpg]

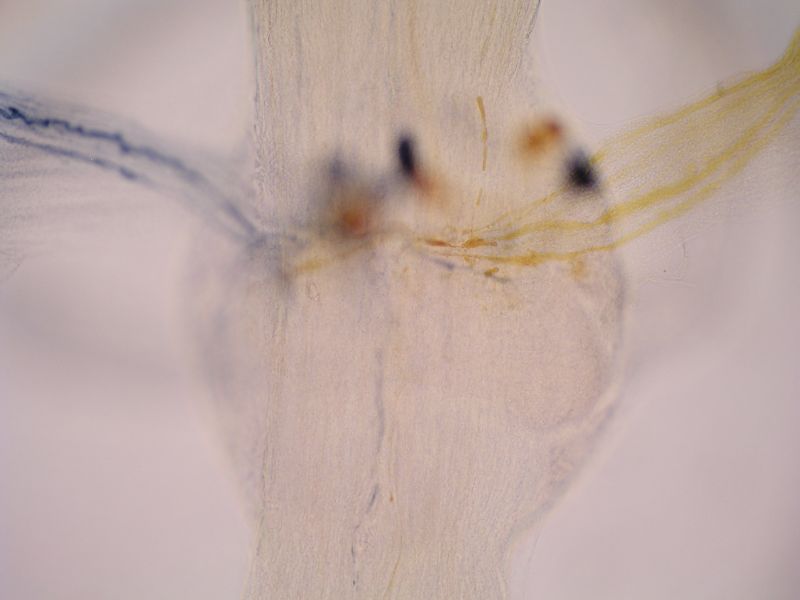

Supplement: Supplemental Information 2 — Micrographs of N2 backfills. Images have been reduced in size. [file peerj-03-1112-s002.zip › A1N2 2009 07 07 stack a (18).jpg]

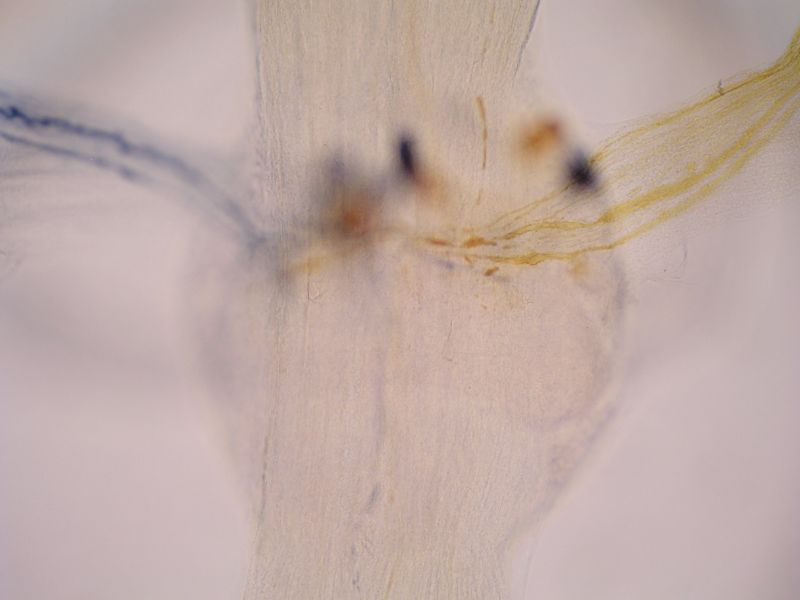

Supplement: Supplemental Information 2 — Micrographs of N2 backfills. Images have been reduced in size. [file peerj-03-1112-s002.zip › A1N2 2009 07 07 stack a (19).jpg]

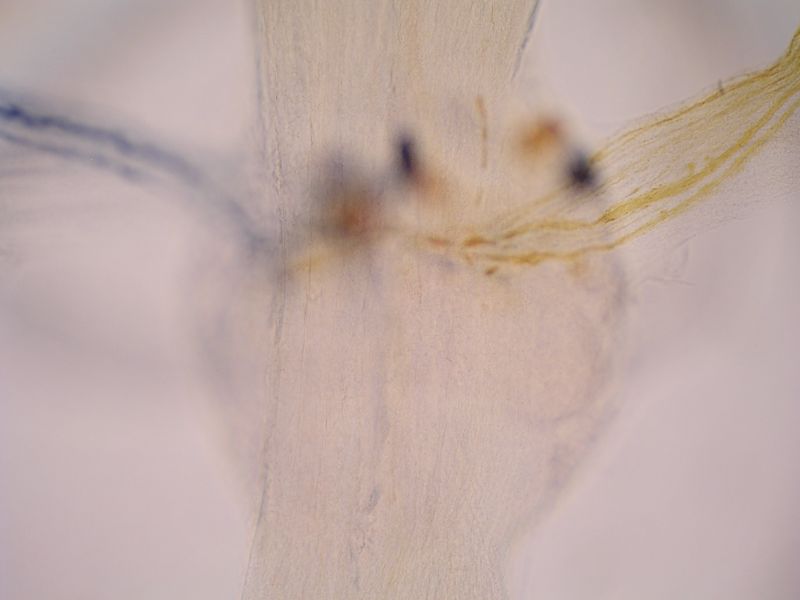

Supplement: Supplemental Information 2 — Micrographs of N2 backfills. Images have been reduced in size. [file peerj-03-1112-s002.zip › A1N2 2009 07 07 stack a (20).jpg]

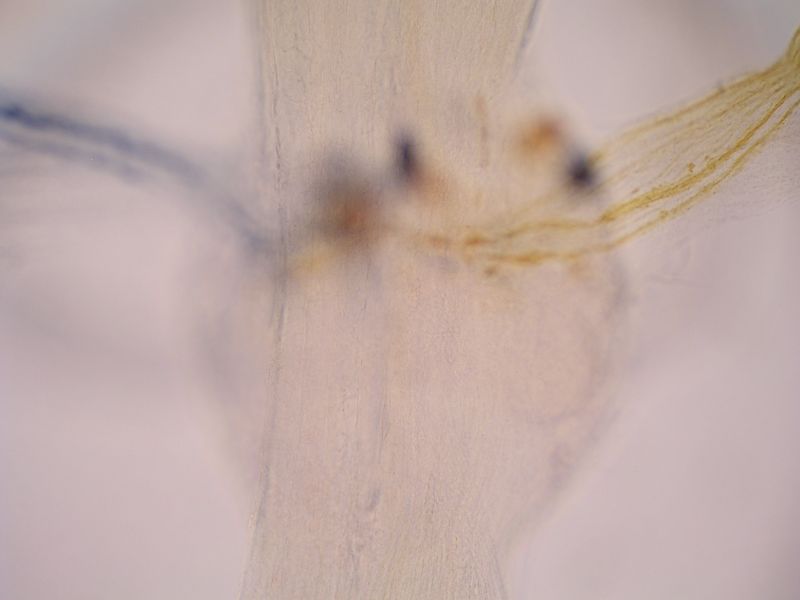

Supplement: Supplemental Information 2 — Micrographs of N2 backfills. Images have been reduced in size. [file peerj-03-1112-s002.zip › A1N2 2009 07 07 stack a (21).jpg]

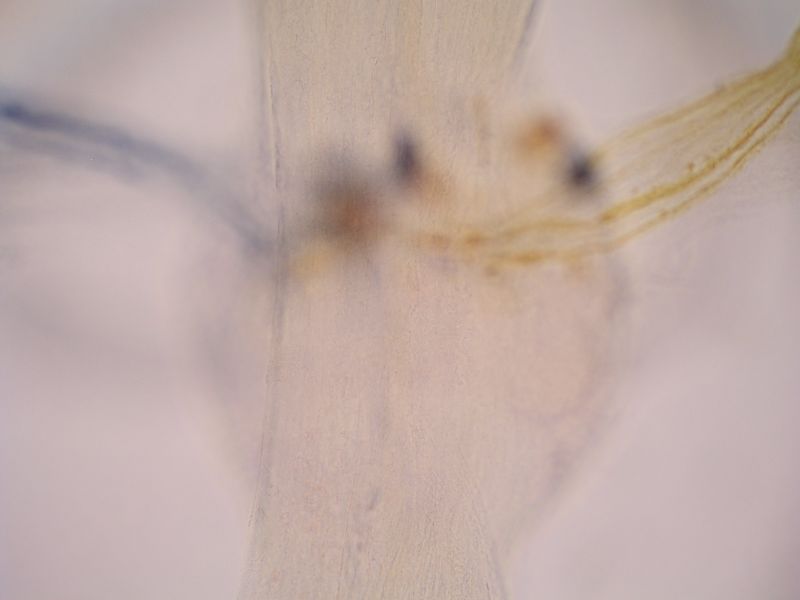

Supplement: Supplemental Information 2 — Micrographs of N2 backfills. Images have been reduced in size. [file peerj-03-1112-s002.zip › A1N2 2009 07 07 stack a (22).jpg]

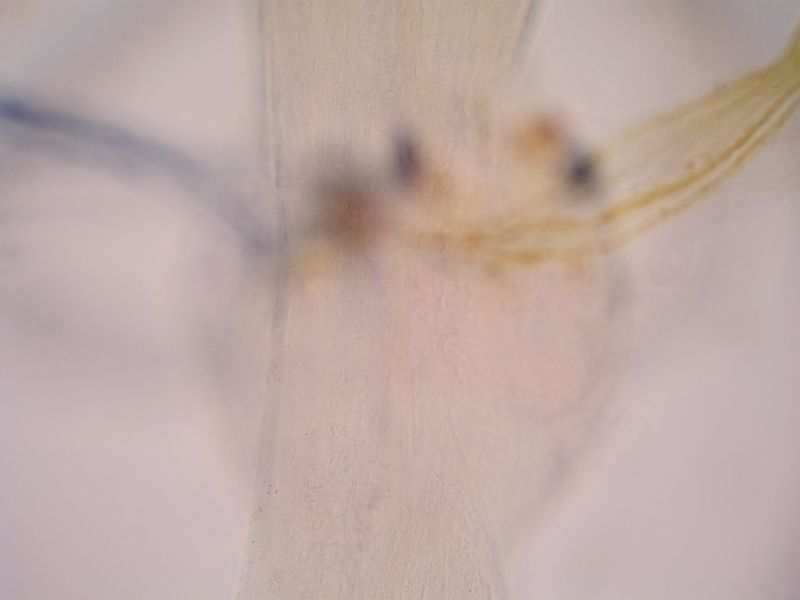

Supplement: Supplemental Information 2 — Micrographs of N2 backfills. Images have been reduced in size. [file peerj-03-1112-s002.zip › A1N2 2009 07 07 stack a (23).jpg]

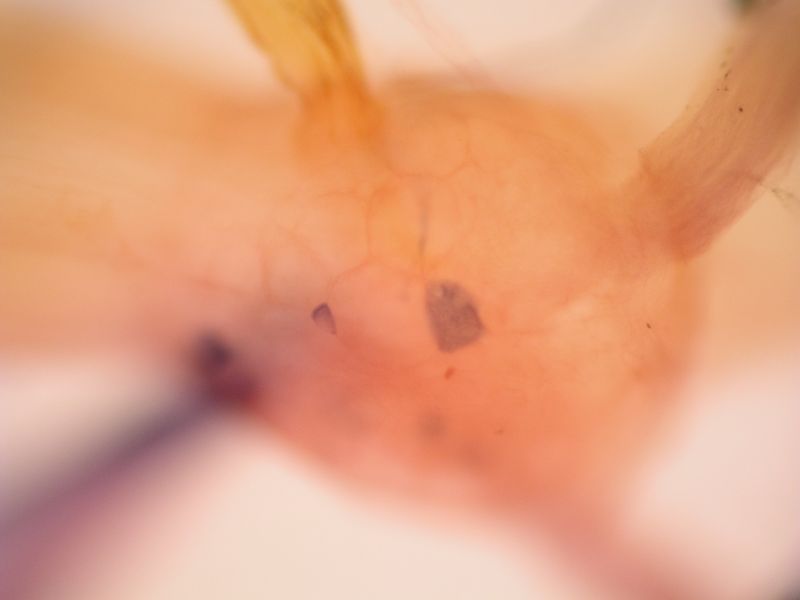

Supplement: Supplemental Information 2 — Micrographs of N2 backfills. Images have been reduced in size. [file peerj-03-1112-s002.zip › A2 P1010029.jpg]

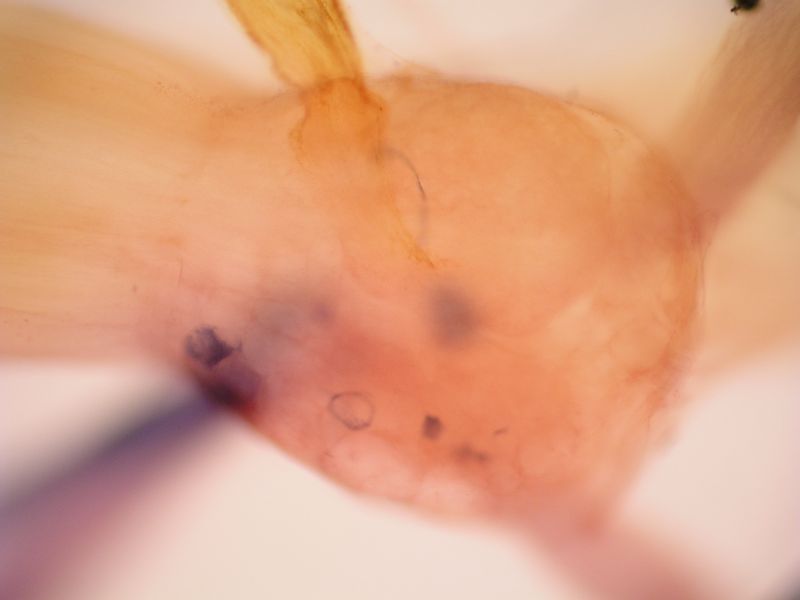

Supplement: Supplemental Information 2 — Micrographs of N2 backfills. Images have been reduced in size. [file peerj-03-1112-s002.zip › A2 P1010030.jpg]

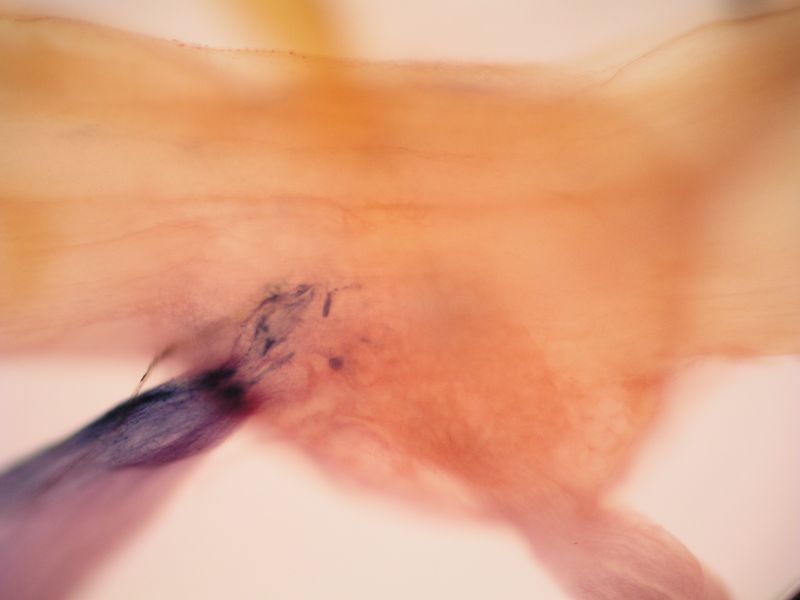

Supplement: Supplemental Information 2 — Micrographs of N2 backfills. Images have been reduced in size. [file peerj-03-1112-s002.zip › A2 P1010031.jpg]

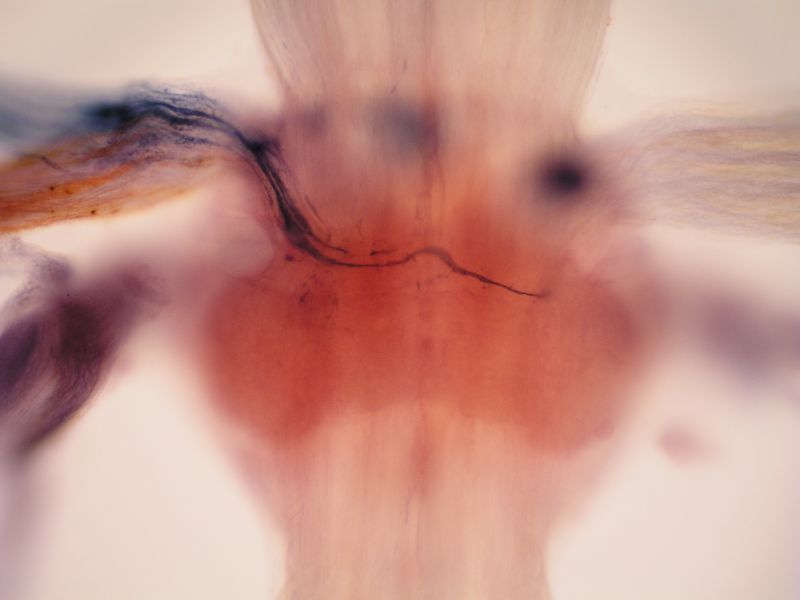

Supplement: Supplemental Information 2 — Micrographs of N2 backfills. Images have been reduced in size. [file peerj-03-1112-s002.zip › A2N2 2009 06 21 a.jpg]

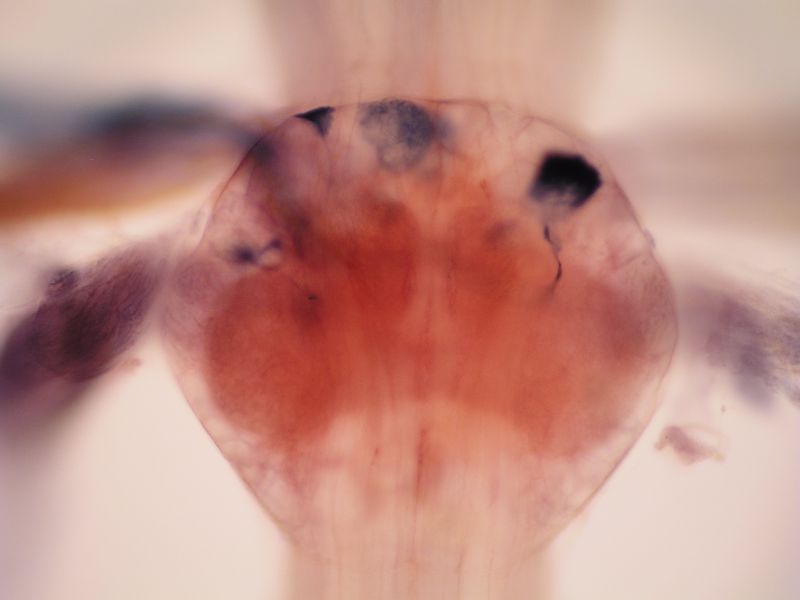

Supplement: Supplemental Information 2 — Micrographs of N2 backfills. Images have been reduced in size. [file peerj-03-1112-s002.zip › A2N2 2009 06 21 b.jpg]

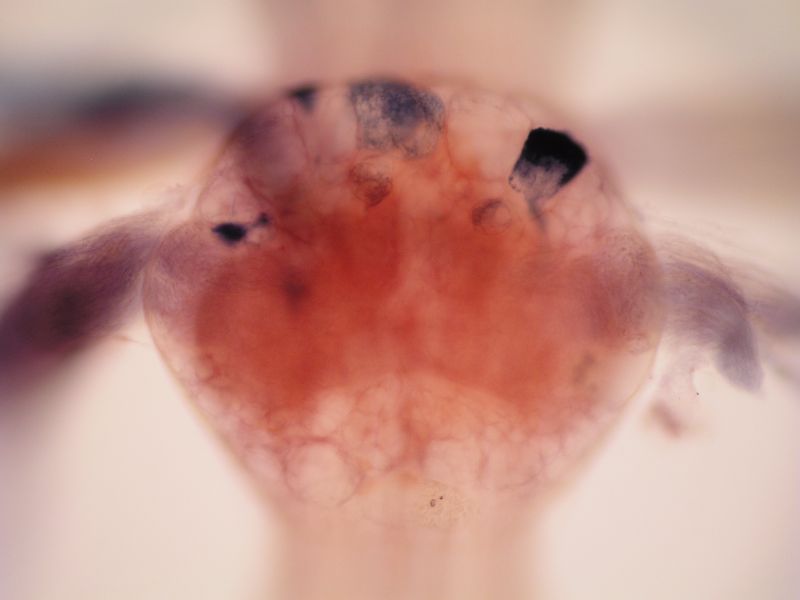

Supplement: Supplemental Information 2 — Micrographs of N2 backfills. Images have been reduced in size. [file peerj-03-1112-s002.zip › A2N2 2009 06 21 c.jpg]
