# Supplementary figures and images for: Motor neurons in the escape response circuit of white shrimp (Litopenaeus setiferus) (part 4 of 4)
Source: PeerJ. 2015 Jul 21;3:e1112. doi: 10.7717/peerj.1112 (PMC4517965; doi:10.7717/peerj.1112)

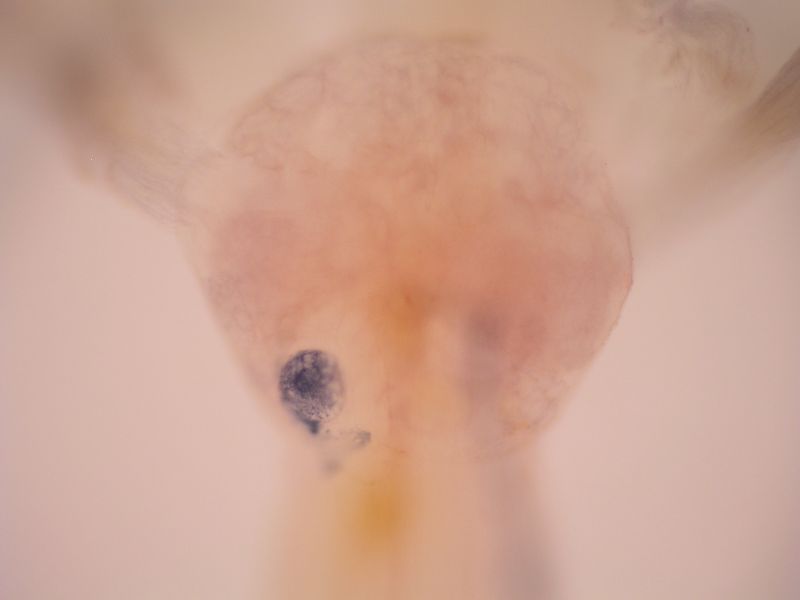

Supplement: Supplemental Information 2 — Micrographs of N3 backfills. Images have been reduced in size. [file peerj-03-1112-s003.zip › A1N3 posterior 2009 07 13 (1).jpg]

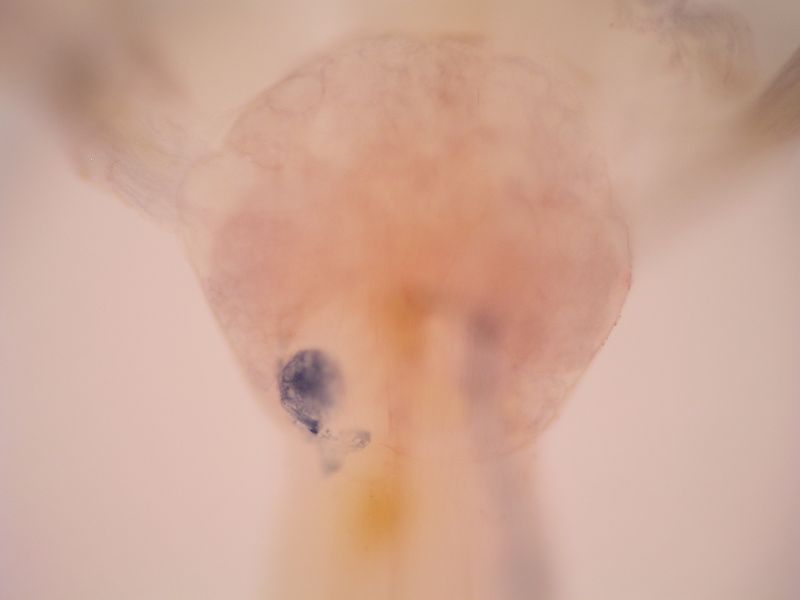

Supplement: Supplemental Information 2 — Micrographs of N3 backfills. Images have been reduced in size. [file peerj-03-1112-s003.zip › A1N3 posterior 2009 07 13 (2).jpg]

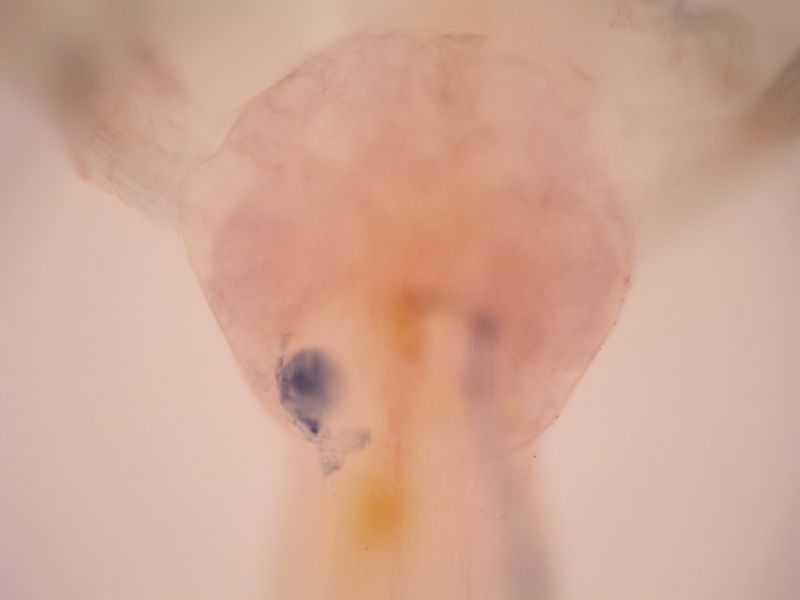

Supplement: Supplemental Information 2 — Micrographs of N3 backfills. Images have been reduced in size. [file peerj-03-1112-s003.zip › A1N3 posterior 2009 07 13 (3).jpg]

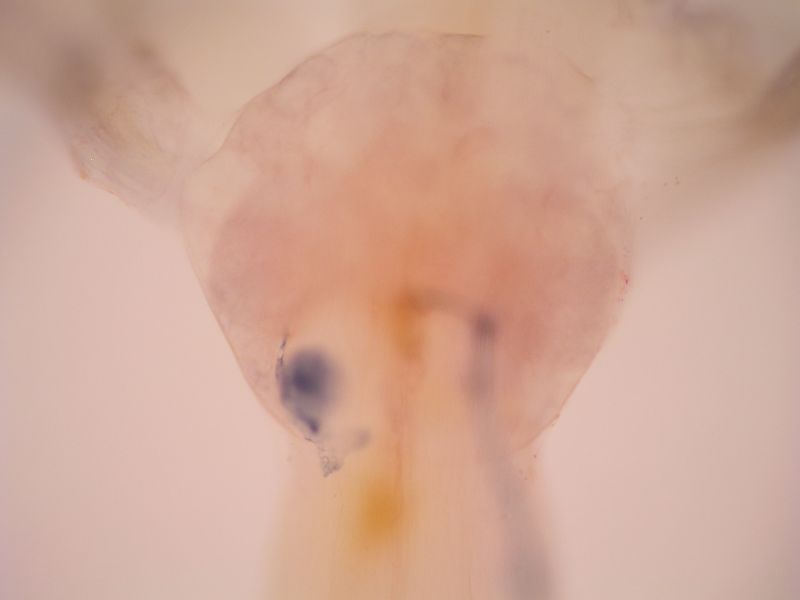

Supplement: Supplemental Information 2 — Micrographs of N3 backfills. Images have been reduced in size. [file peerj-03-1112-s003.zip › A1N3 posterior 2009 07 13 (4).jpg]

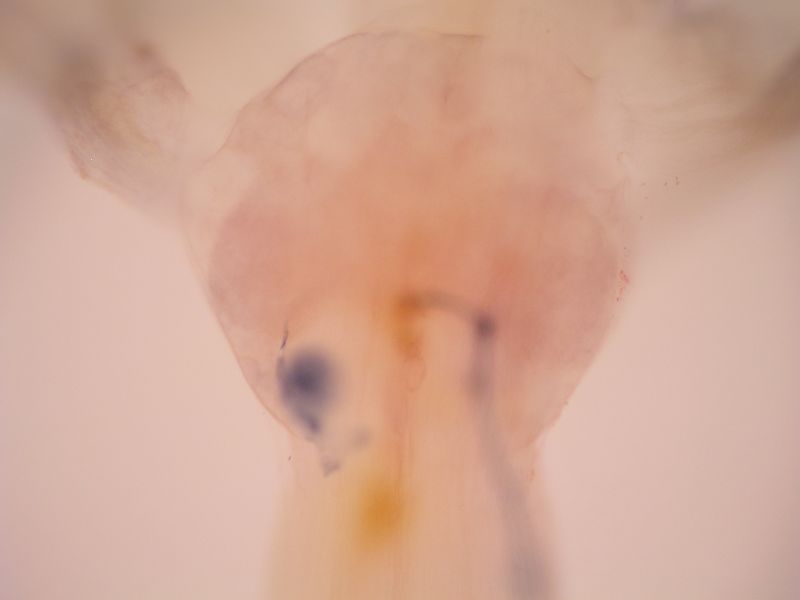

Supplement: Supplemental Information 2 — Micrographs of N3 backfills. Images have been reduced in size. [file peerj-03-1112-s003.zip › A1N3 posterior 2009 07 13 (5).jpg]

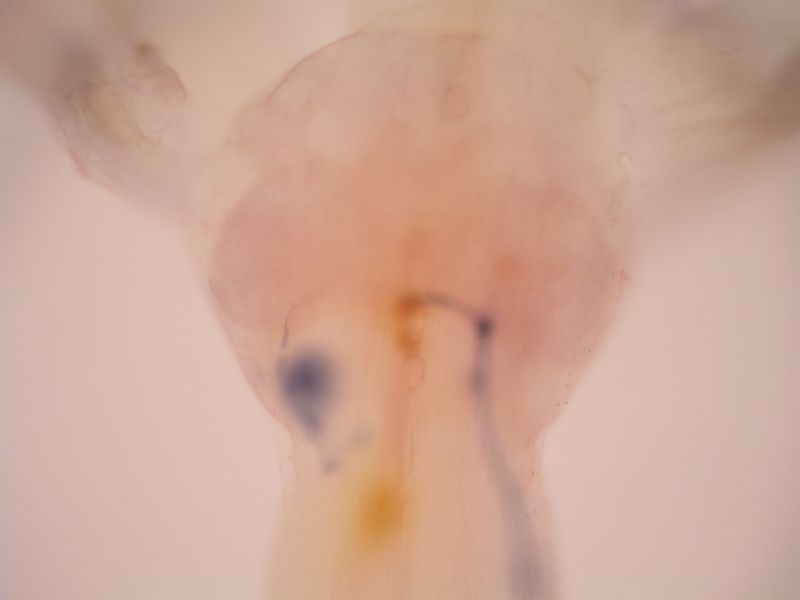

Supplement: Supplemental Information 2 — Micrographs of N3 backfills. Images have been reduced in size. [file peerj-03-1112-s003.zip › A1N3 posterior 2009 07 13 (6).jpg]

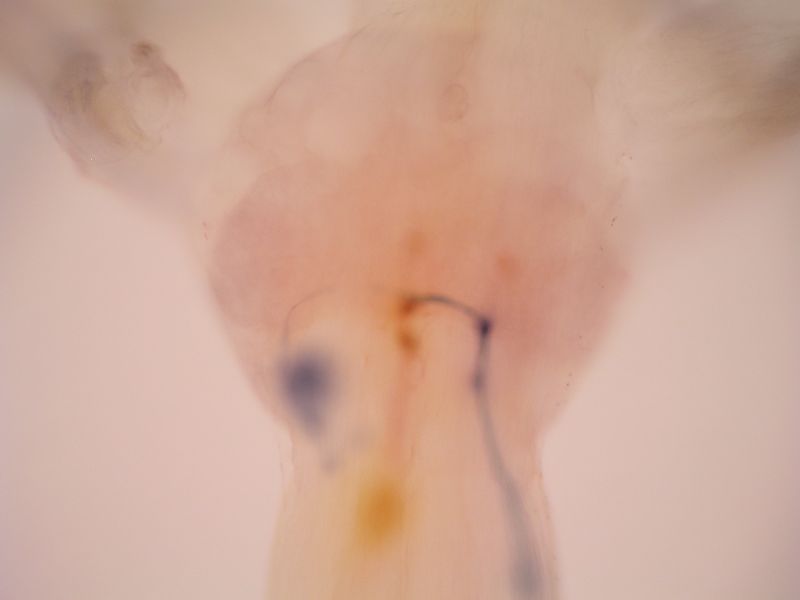

Supplement: Supplemental Information 2 — Micrographs of N3 backfills. Images have been reduced in size. [file peerj-03-1112-s003.zip › A1N3 posterior 2009 07 13 (7).jpg]

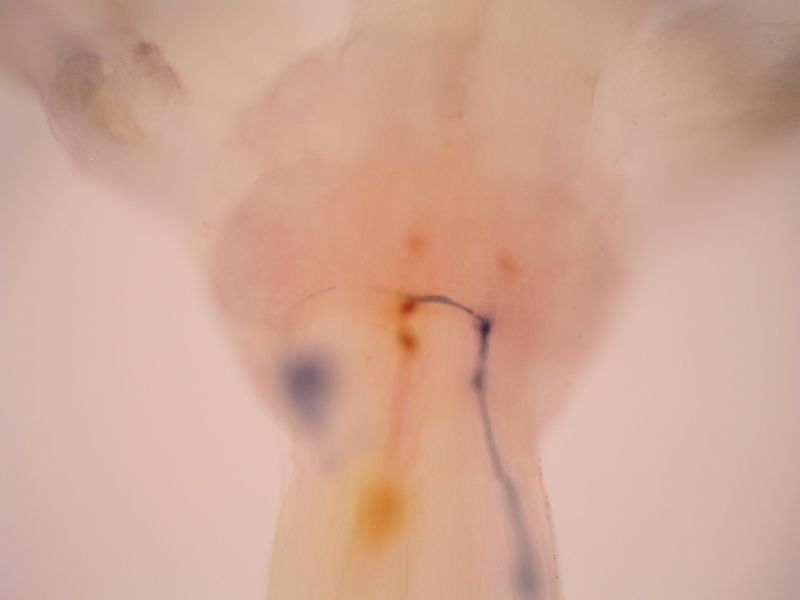

Supplement: Supplemental Information 2 — Micrographs of N3 backfills. Images have been reduced in size. [file peerj-03-1112-s003.zip › A1N3 posterior 2009 07 13 (8).jpg]

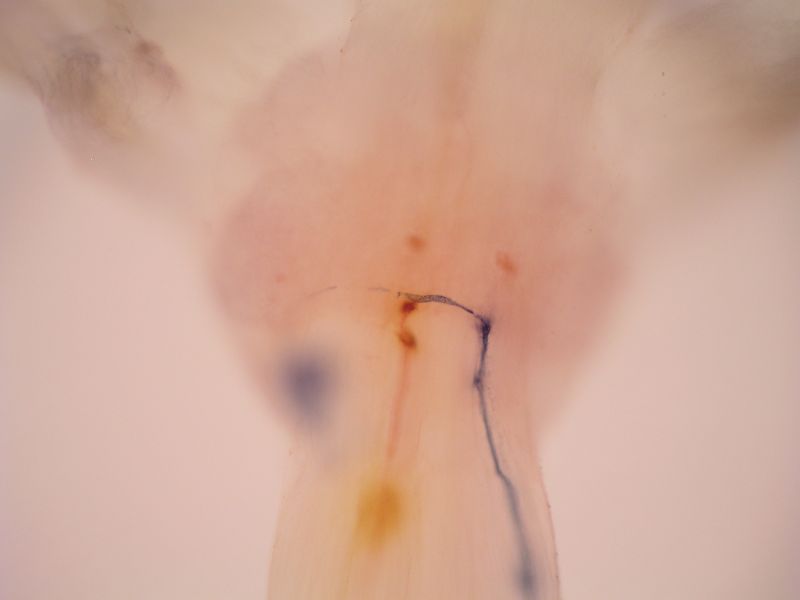

Supplement: Supplemental Information 2 — Micrographs of N3 backfills. Images have been reduced in size. [file peerj-03-1112-s003.zip › A1N3 posterior 2009 07 13 (9).jpg]

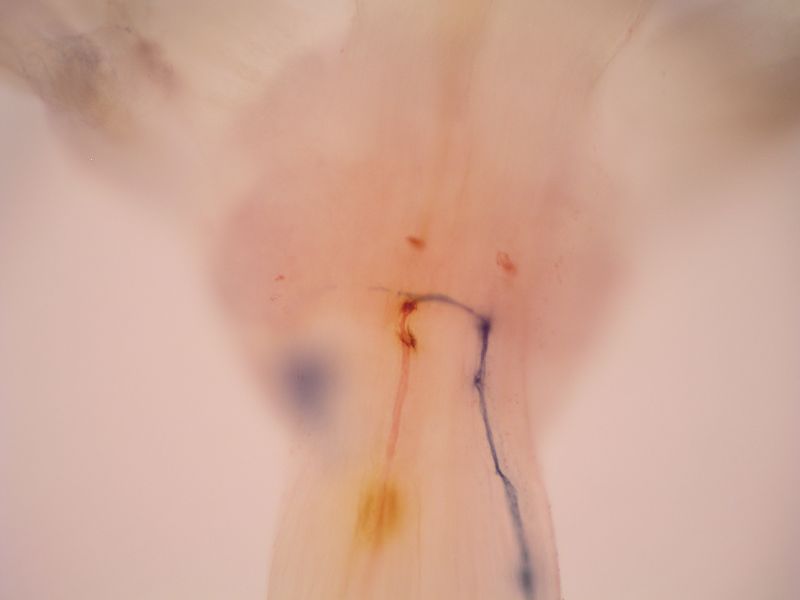

Supplement: Supplemental Information 2 — Micrographs of N3 backfills. Images have been reduced in size. [file peerj-03-1112-s003.zip › A1N3 posterior 2009 07 13.jpg]

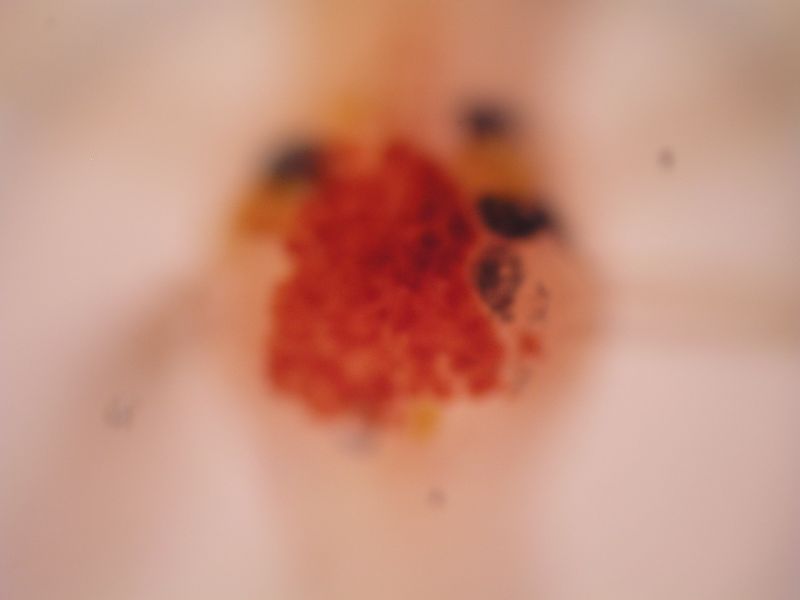

Supplement: Supplemental Information 2 — Micrographs of N3 backfills. Images have been reduced in size. [file peerj-03-1112-s003.zip › A1N3 redone 2009 07 13 (1).jpg]

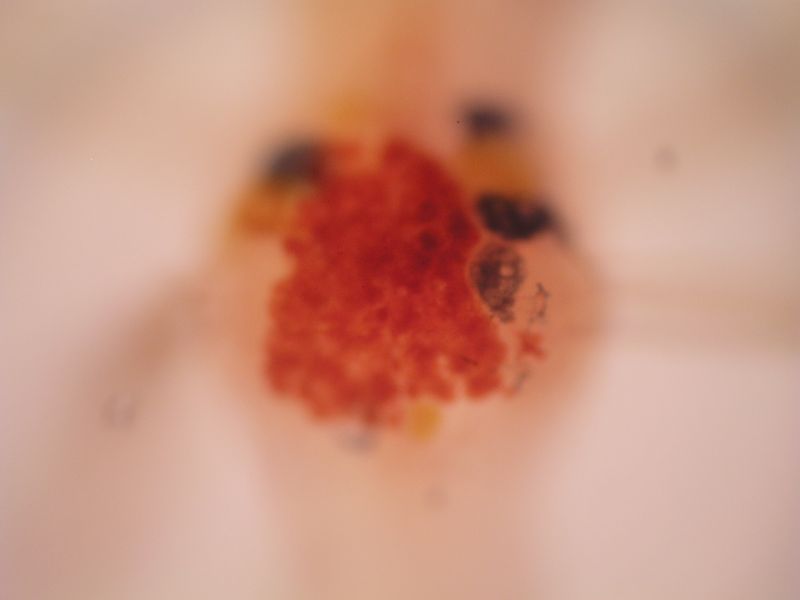

Supplement: Supplemental Information 2 — Micrographs of N3 backfills. Images have been reduced in size. [file peerj-03-1112-s003.zip › A1N3 redone 2009 07 13 (2).jpg]

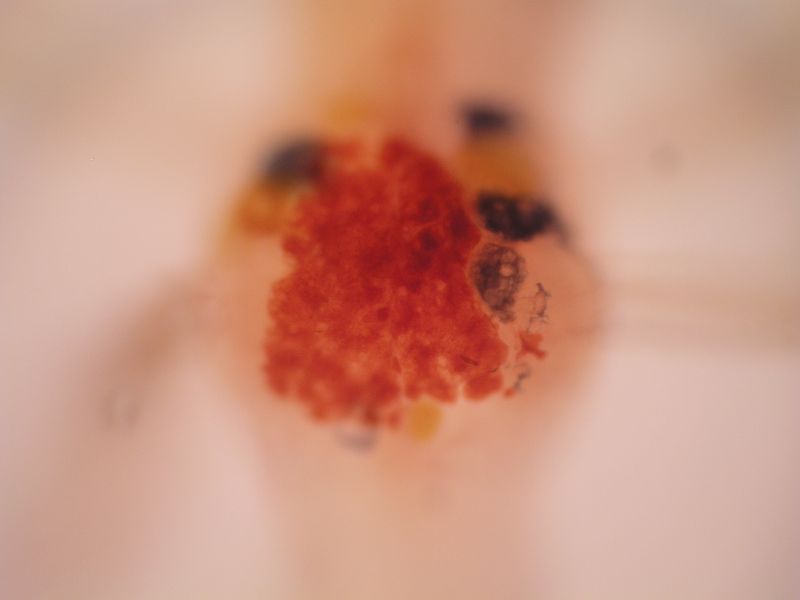

Supplement: Supplemental Information 2 — Micrographs of N3 backfills. Images have been reduced in size. [file peerj-03-1112-s003.zip › A1N3 redone 2009 07 13 (3).jpg]

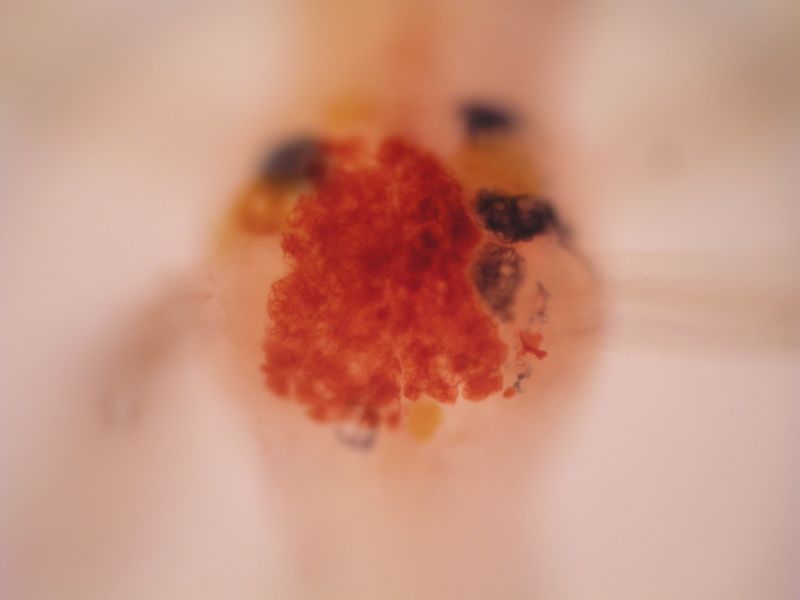

Supplement: Supplemental Information 2 — Micrographs of N3 backfills. Images have been reduced in size. [file peerj-03-1112-s003.zip › A1N3 redone 2009 07 13 (4).jpg]

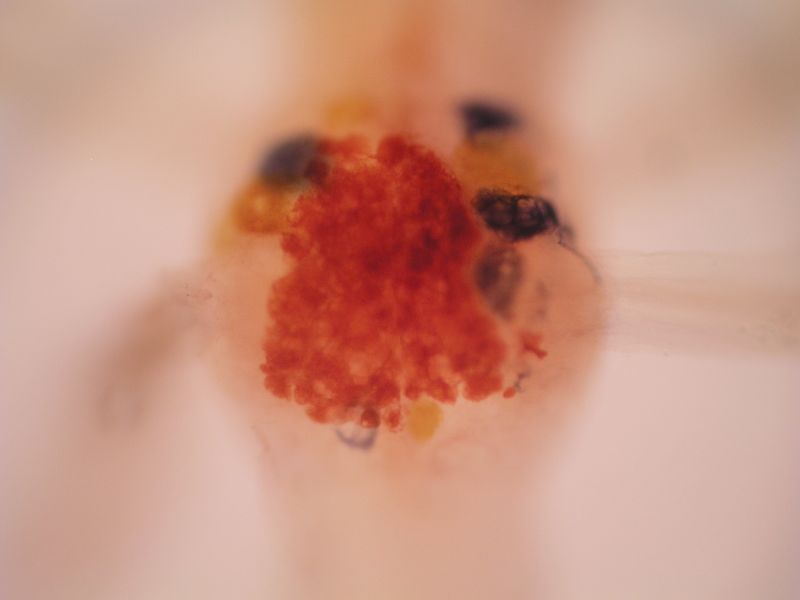

Supplement: Supplemental Information 2 — Micrographs of N3 backfills. Images have been reduced in size. [file peerj-03-1112-s003.zip › A1N3 redone 2009 07 13 (5).jpg]

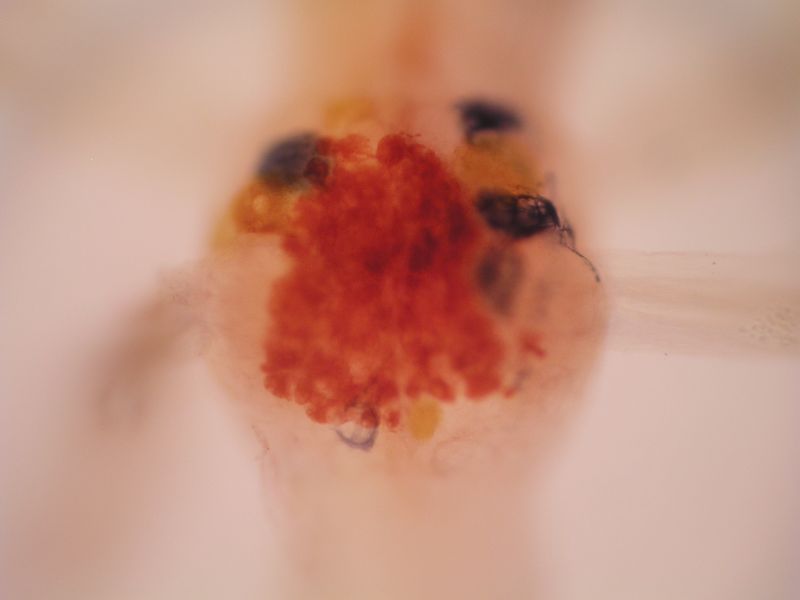

Supplement: Supplemental Information 2 — Micrographs of N3 backfills. Images have been reduced in size. [file peerj-03-1112-s003.zip › A1N3 redone 2009 07 13 (6).jpg]

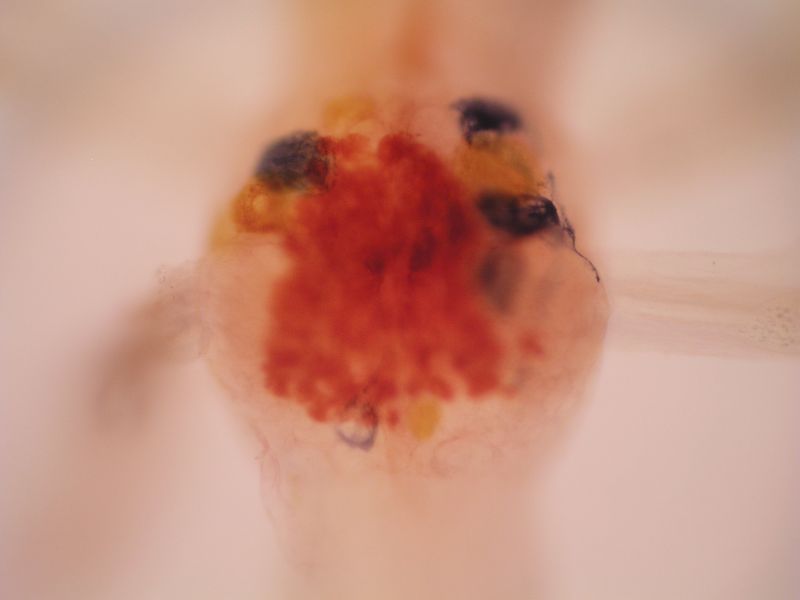

Supplement: Supplemental Information 2 — Micrographs of N3 backfills. Images have been reduced in size. [file peerj-03-1112-s003.zip › A1N3 redone 2009 07 13 (7).jpg]

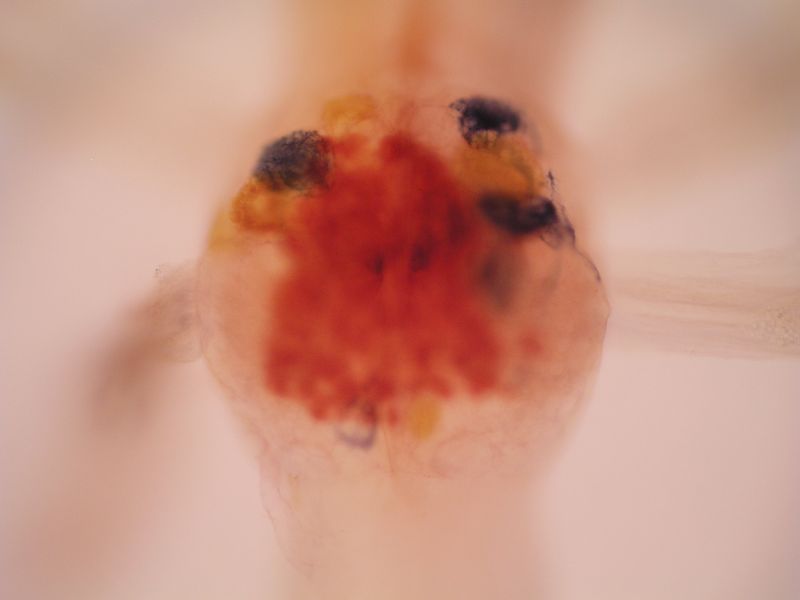

Supplement: Supplemental Information 2 — Micrographs of N3 backfills. Images have been reduced in size. [file peerj-03-1112-s003.zip › A1N3 redone 2009 07 13 (8).jpg]

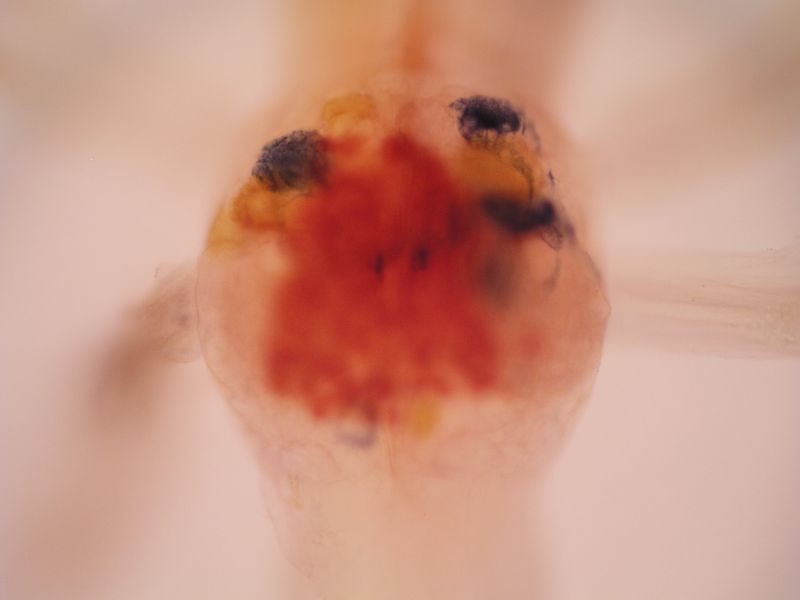

Supplement: Supplemental Information 2 — Micrographs of N3 backfills. Images have been reduced in size. [file peerj-03-1112-s003.zip › A1N3 redone 2009 07 13 (9).jpg]

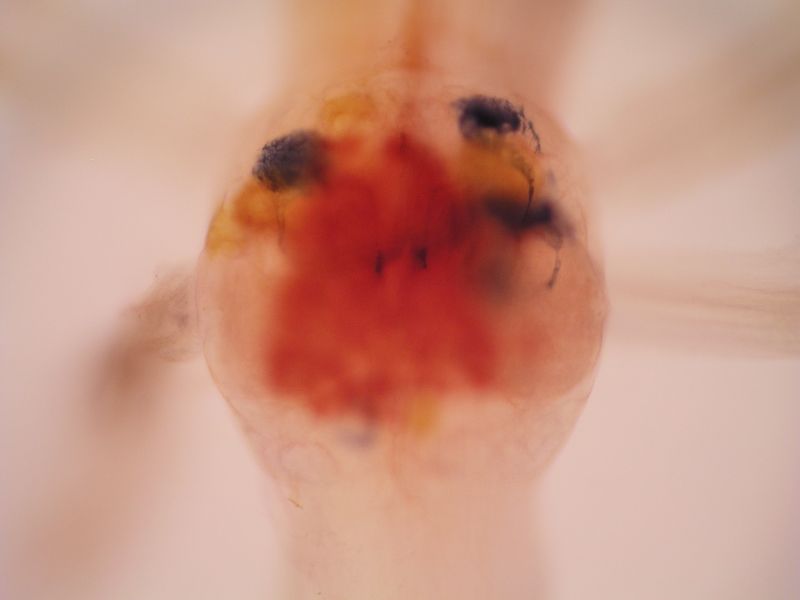

Supplement: Supplemental Information 2 — Micrographs of N3 backfills. Images have been reduced in size. [file peerj-03-1112-s003.zip › A1N3 redone 2009 07 13 (10).jpg]

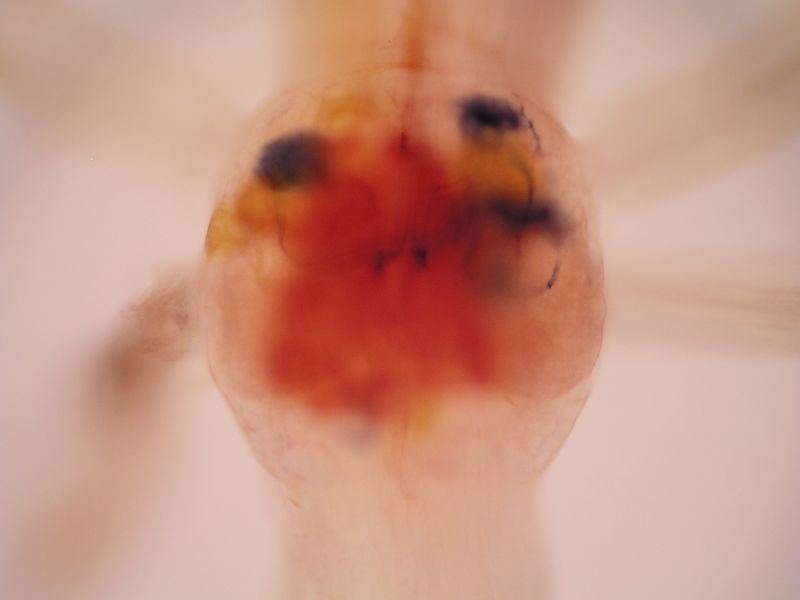

Supplement: Supplemental Information 2 — Micrographs of N3 backfills. Images have been reduced in size. [file peerj-03-1112-s003.zip › A1N3 redone 2009 07 13 (11).jpg]

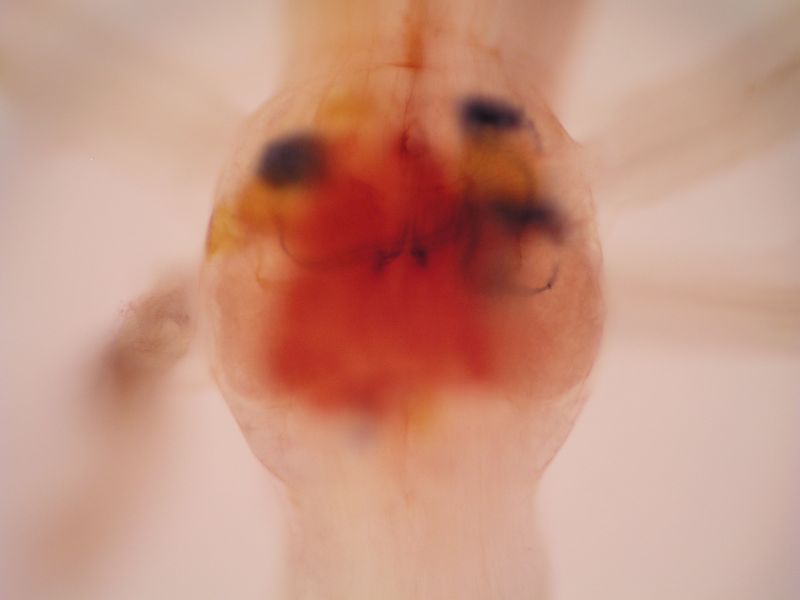

Supplement: Supplemental Information 2 — Micrographs of N3 backfills. Images have been reduced in size. [file peerj-03-1112-s003.zip › A1N3 redone 2009 07 13 (12).jpg]

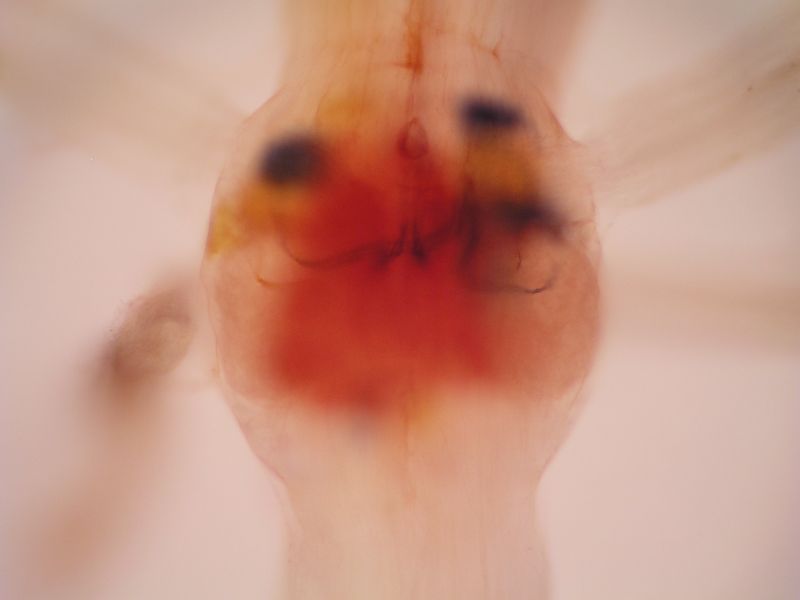

Supplement: Supplemental Information 2 — Micrographs of N3 backfills. Images have been reduced in size. [file peerj-03-1112-s003.zip › A1N3 redone 2009 07 13 (13).jpg]

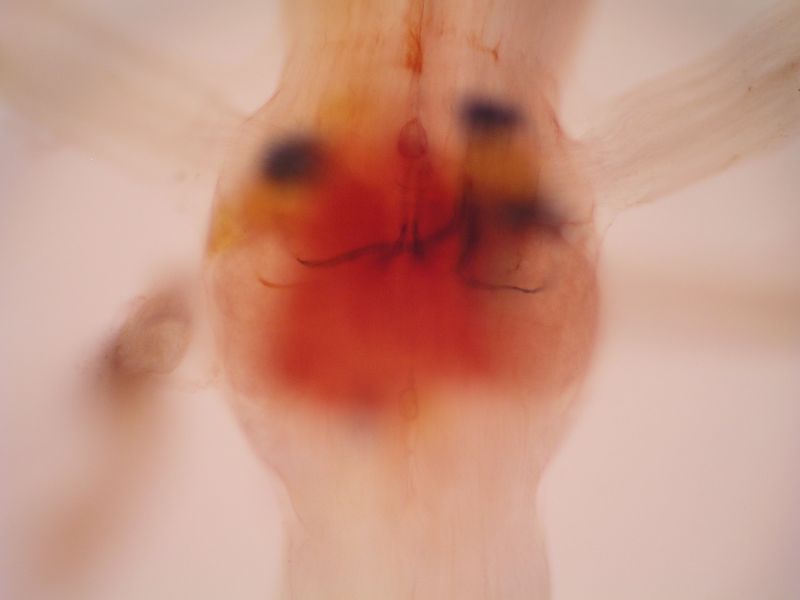

Supplement: Supplemental Information 2 — Micrographs of N3 backfills. Images have been reduced in size. [file peerj-03-1112-s003.zip › A1N3 redone 2009 07 13 (14).jpg]

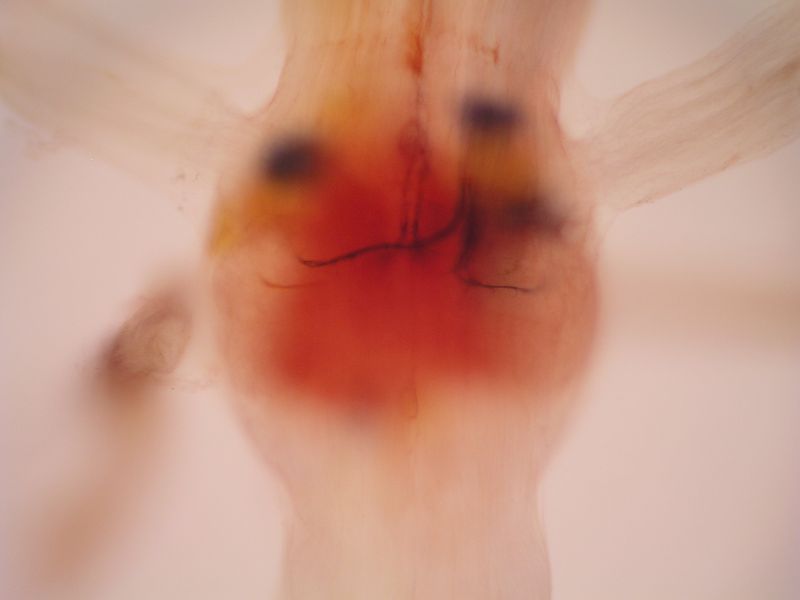

Supplement: Supplemental Information 2 — Micrographs of N3 backfills. Images have been reduced in size. [file peerj-03-1112-s003.zip › A1N3 redone 2009 07 13 (15).jpg]

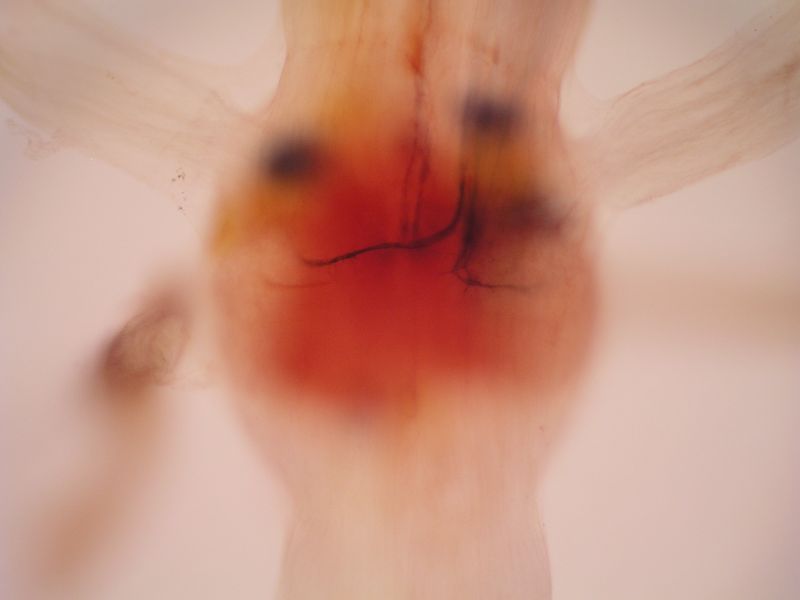

Supplement: Supplemental Information 2 — Micrographs of N3 backfills. Images have been reduced in size. [file peerj-03-1112-s003.zip › A1N3 redone 2009 07 13 (16).jpg]

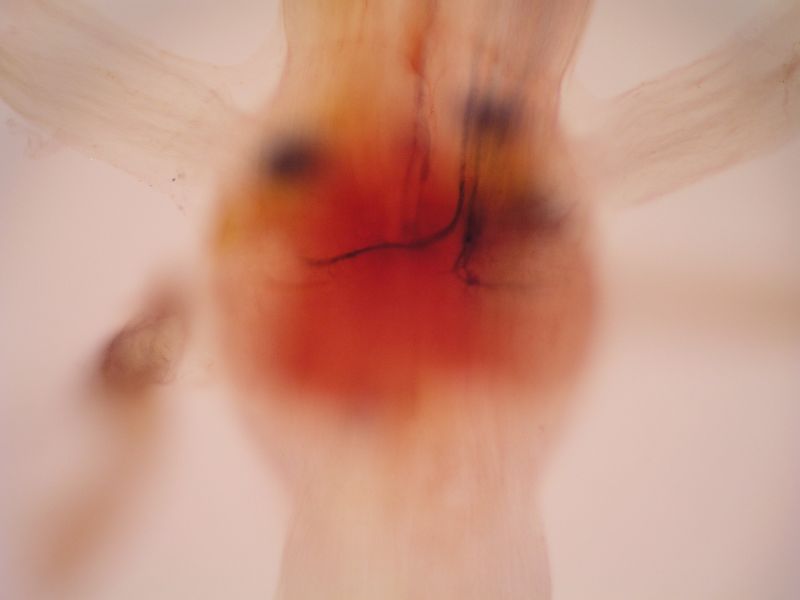

Supplement: Supplemental Information 2 — Micrographs of N3 backfills. Images have been reduced in size. [file peerj-03-1112-s003.zip › A1N3 redone 2009 07 13 (17).jpg]

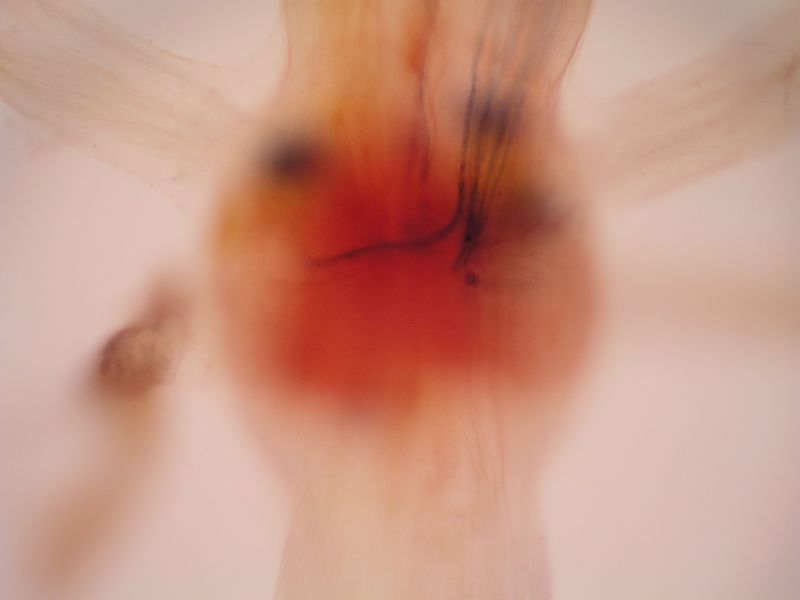

Supplement: Supplemental Information 2 — Micrographs of N3 backfills. Images have been reduced in size. [file peerj-03-1112-s003.zip › A1N3 redone 2009 07 13 (18).jpg]

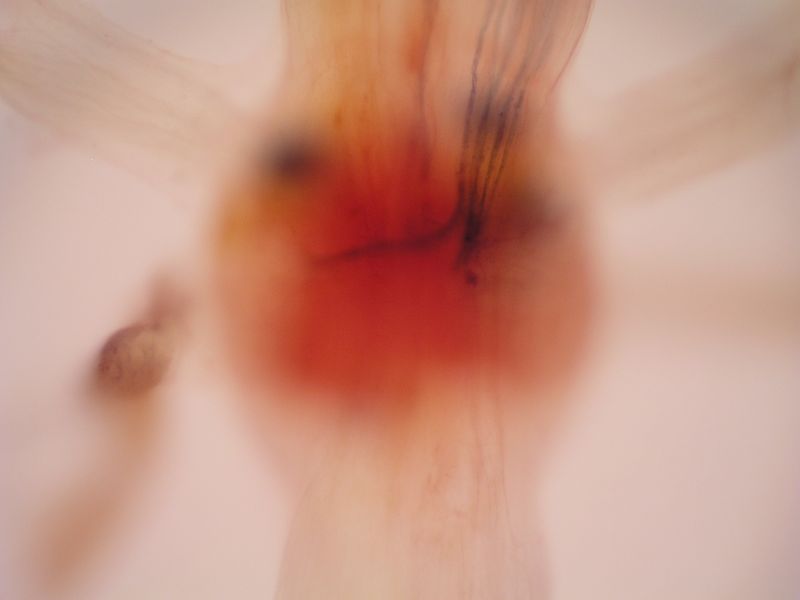

Supplement: Supplemental Information 2 — Micrographs of N3 backfills. Images have been reduced in size. [file peerj-03-1112-s003.zip › A1N3 redone 2009 07 13 (19).jpg]

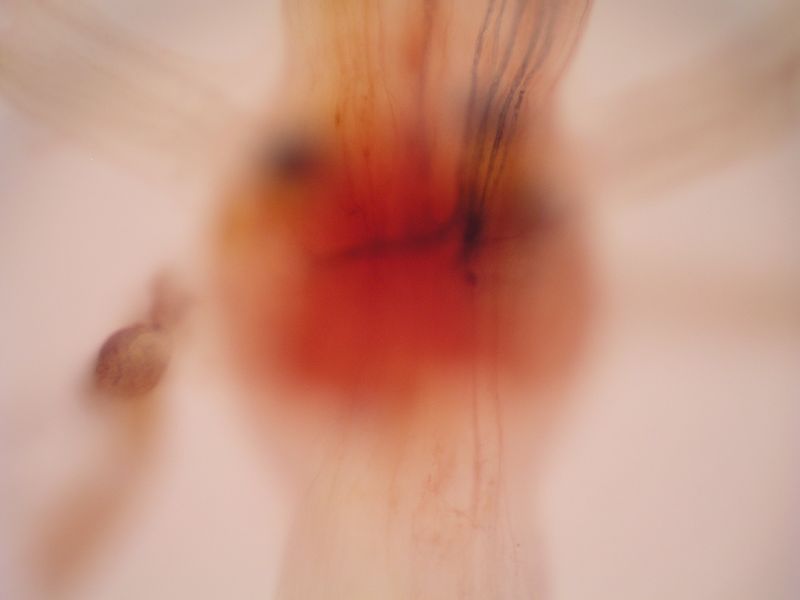

Supplement: Supplemental Information 2 — Micrographs of N3 backfills. Images have been reduced in size. [file peerj-03-1112-s003.zip › A1N3 redone 2009 07 13 (20).jpg]

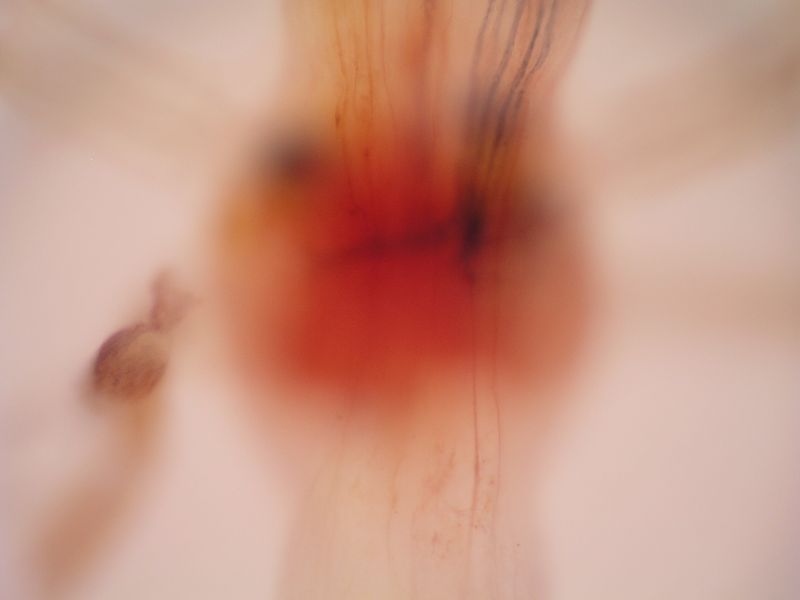

Supplement: Supplemental Information 2 — Micrographs of N3 backfills. Images have been reduced in size. [file peerj-03-1112-s003.zip › A1N3 redone 2009 07 13 (21).jpg]

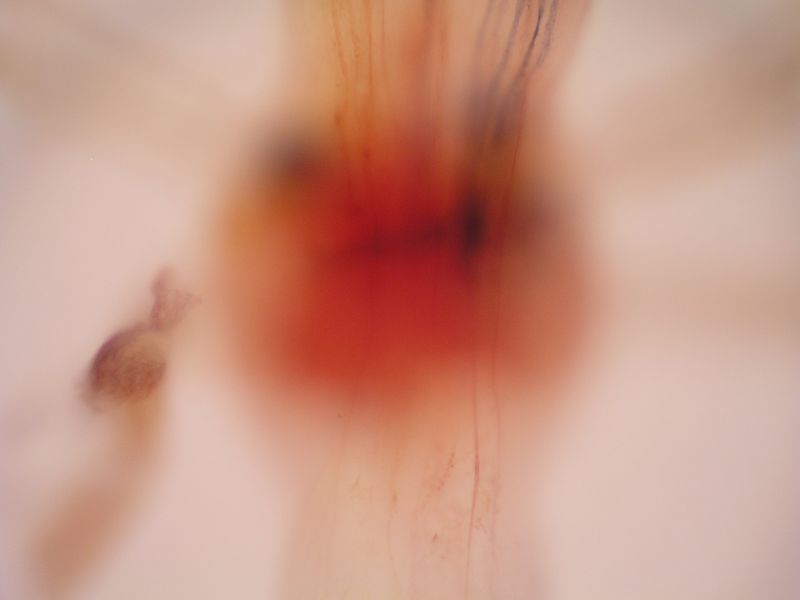

Supplement: Supplemental Information 2 — Micrographs of N3 backfills. Images have been reduced in size. [file peerj-03-1112-s003.zip › A1N3 redone 2009 07 13 (22).jpg]

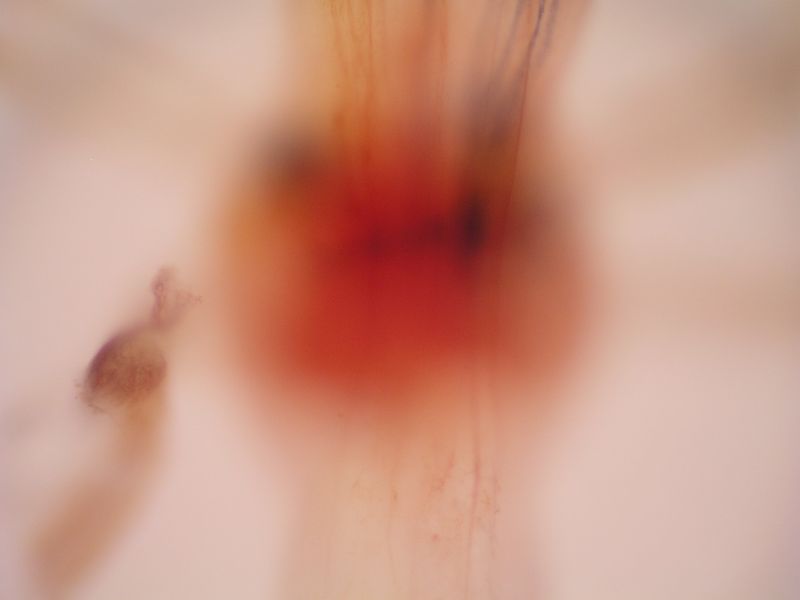

Supplement: Supplemental Information 2 — Micrographs of N3 backfills. Images have been reduced in size. [file peerj-03-1112-s003.zip › A1N3 redone 2009 07 13 (23).jpg]

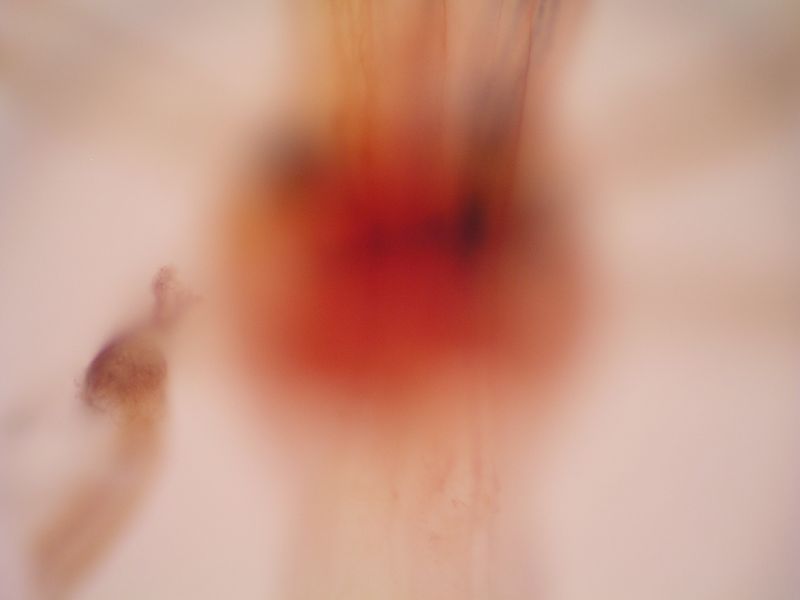

Supplement: Supplemental Information 2 — Micrographs of N3 backfills. Images have been reduced in size. [file peerj-03-1112-s003.zip › A1N3 redone 2009 07 13.jpg]

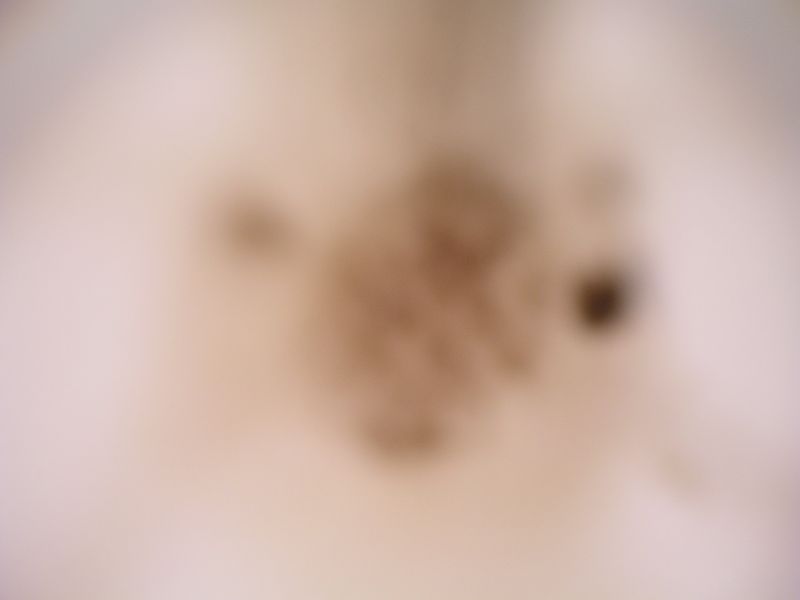

Supplement: Supplemental Information 2 — Micrographs of N3 backfills. Images have been reduced in size. [file peerj-03-1112-s003.zip › A1N3 unilateral 2009 07 09 (1).jpg]

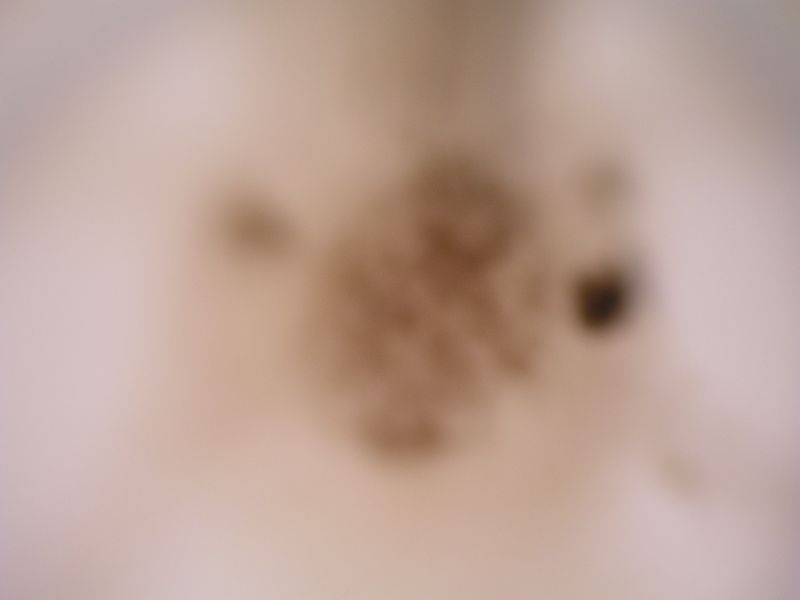

Supplement: Supplemental Information 2 — Micrographs of N3 backfills. Images have been reduced in size. [file peerj-03-1112-s003.zip › A1N3 unilateral 2009 07 09 (2).jpg]

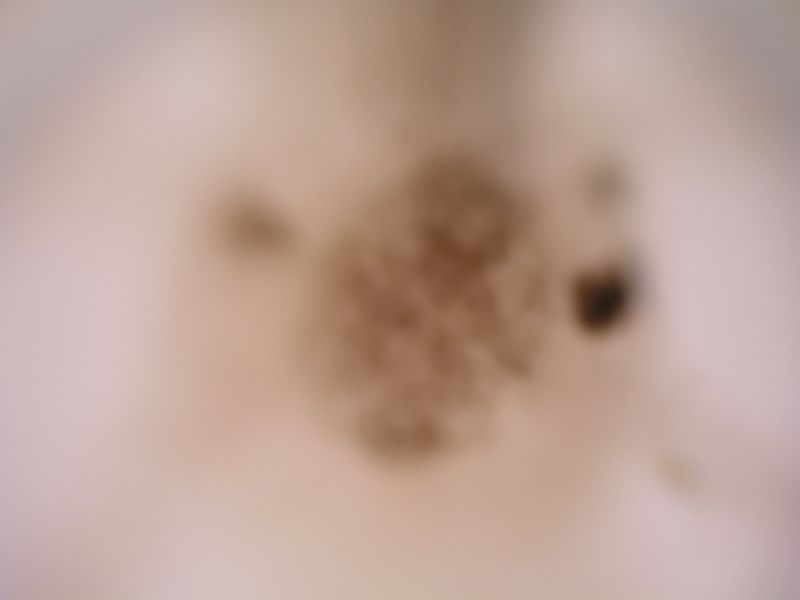

Supplement: Supplemental Information 2 — Micrographs of N3 backfills. Images have been reduced in size. [file peerj-03-1112-s003.zip › A1N3 unilateral 2009 07 09 (3).jpg]

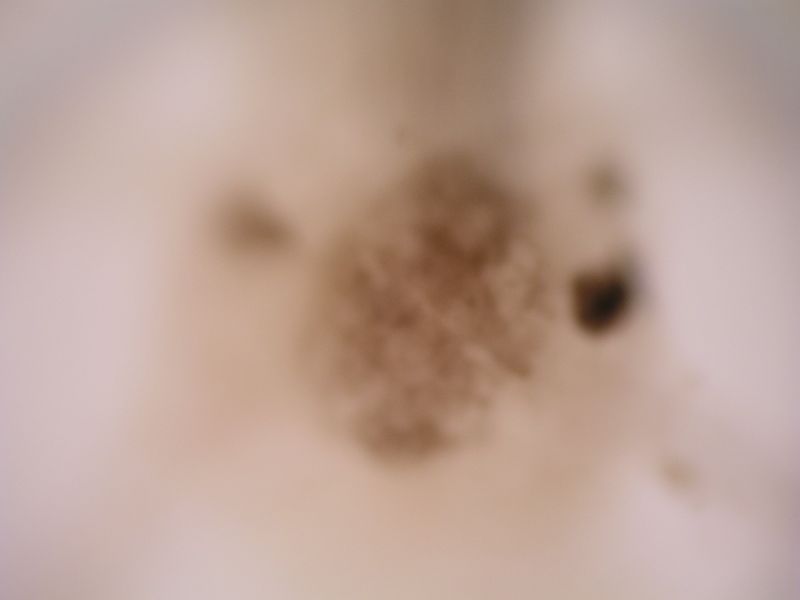

Supplement: Supplemental Information 2 — Micrographs of N3 backfills. Images have been reduced in size. [file peerj-03-1112-s003.zip › A1N3 unilateral 2009 07 09 (4).jpg]

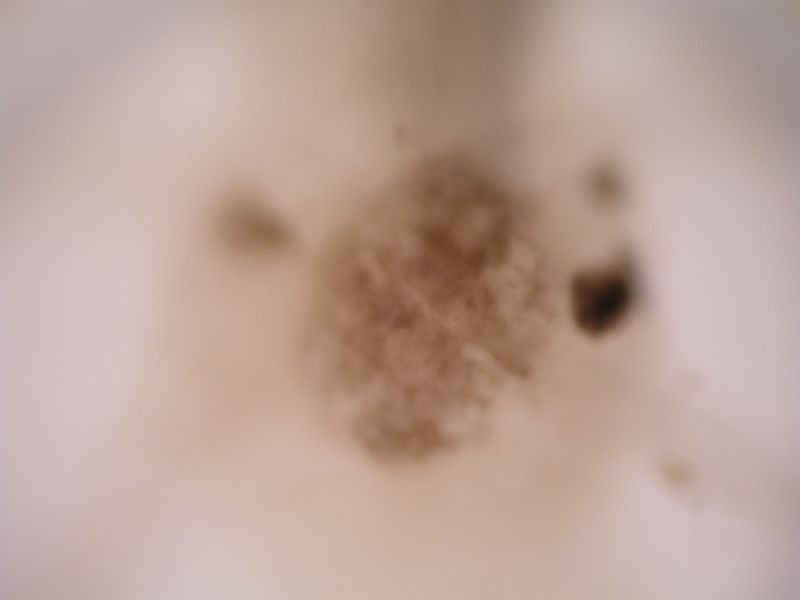

Supplement: Supplemental Information 2 — Micrographs of N3 backfills. Images have been reduced in size. [file peerj-03-1112-s003.zip › A1N3 unilateral 2009 07 09 (5).jpg]

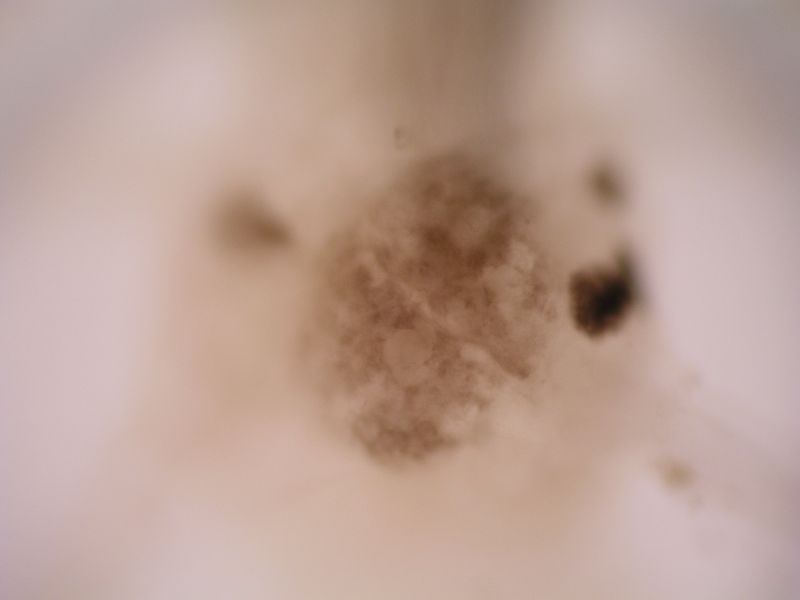

Supplement: Supplemental Information 2 — Micrographs of N3 backfills. Images have been reduced in size. [file peerj-03-1112-s003.zip › A1N3 unilateral 2009 07 09 (6).jpg]

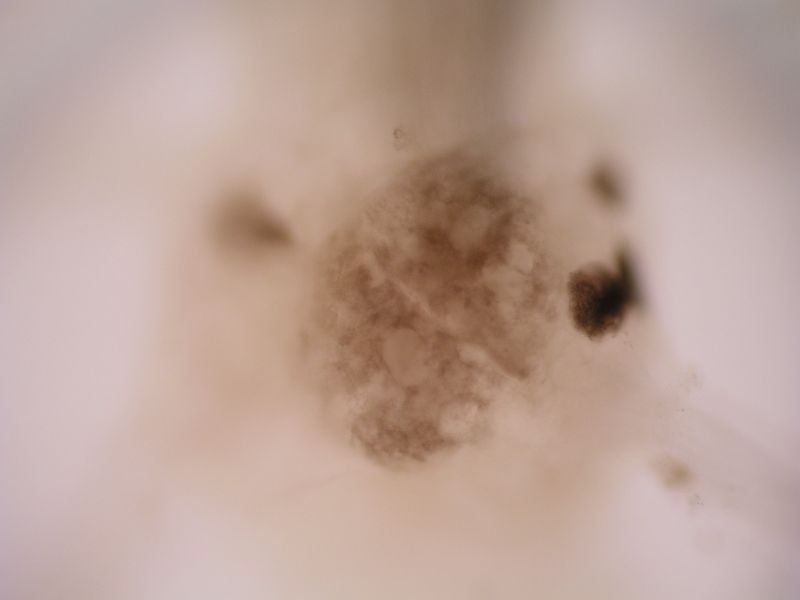

Supplement: Supplemental Information 2 — Micrographs of N3 backfills. Images have been reduced in size. [file peerj-03-1112-s003.zip › A1N3 unilateral 2009 07 09 (7).jpg]

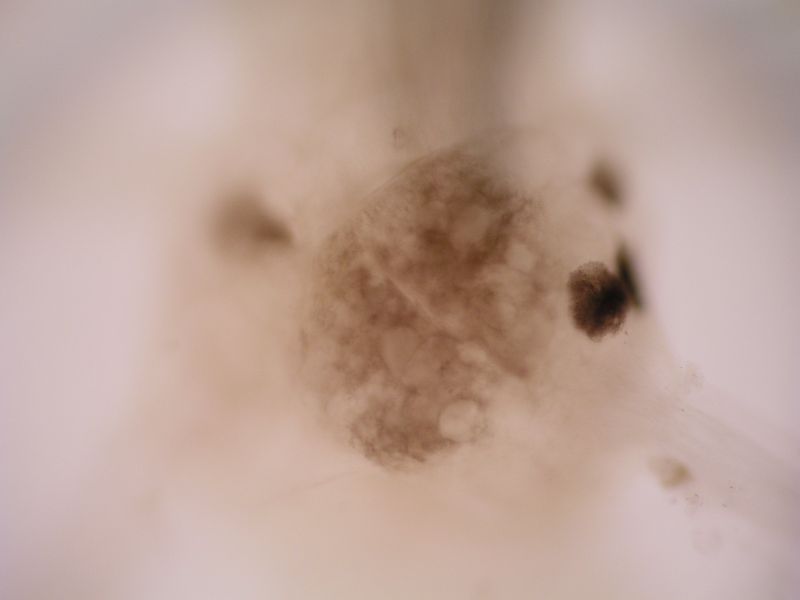

Supplement: Supplemental Information 2 — Micrographs of N3 backfills. Images have been reduced in size. [file peerj-03-1112-s003.zip › A1N3 unilateral 2009 07 09 (8).jpg]

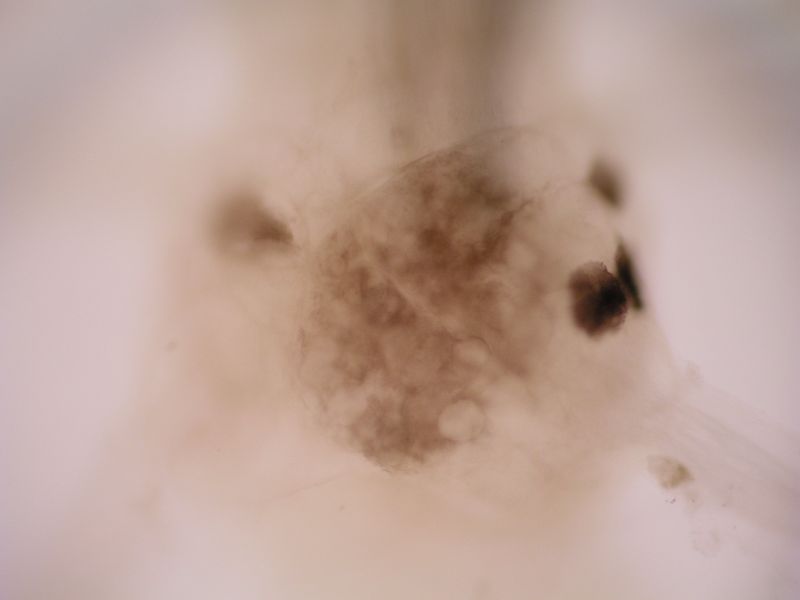

Supplement: Supplemental Information 2 — Micrographs of N3 backfills. Images have been reduced in size. [file peerj-03-1112-s003.zip › A1N3 unilateral 2009 07 09 (9).jpg]

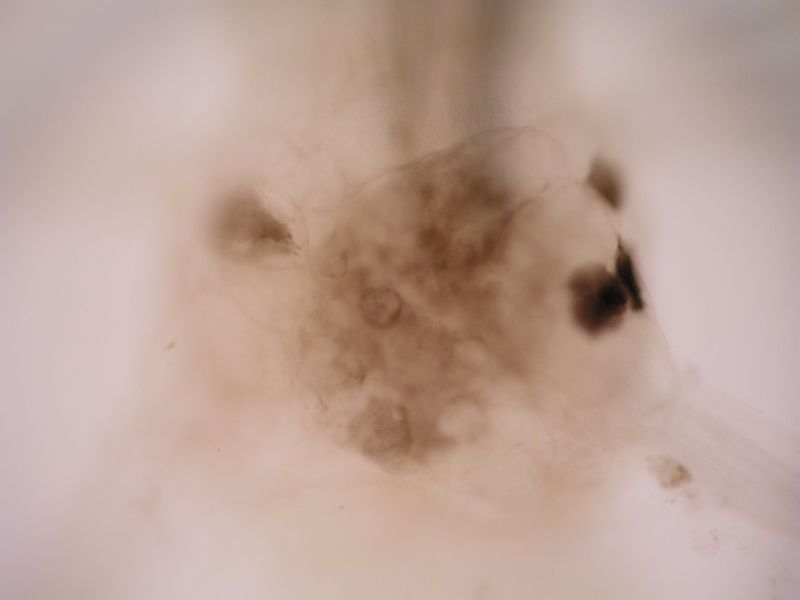

Supplement: Supplemental Information 2 — Micrographs of N3 backfills. Images have been reduced in size. [file peerj-03-1112-s003.zip › A1N3 unilateral 2009 07 09 (10).jpg]

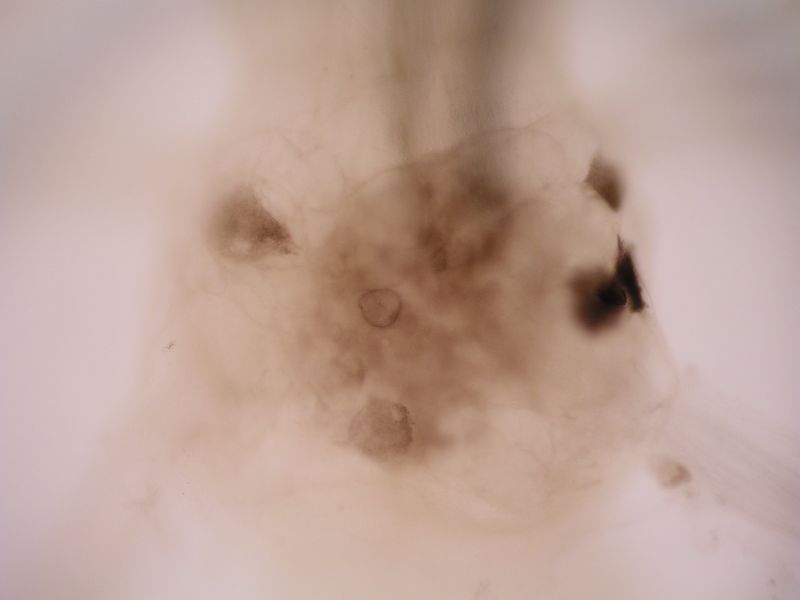

Supplement: Supplemental Information 2 — Micrographs of N3 backfills. Images have been reduced in size. [file peerj-03-1112-s003.zip › A1N3 unilateral 2009 07 09 (11).jpg]

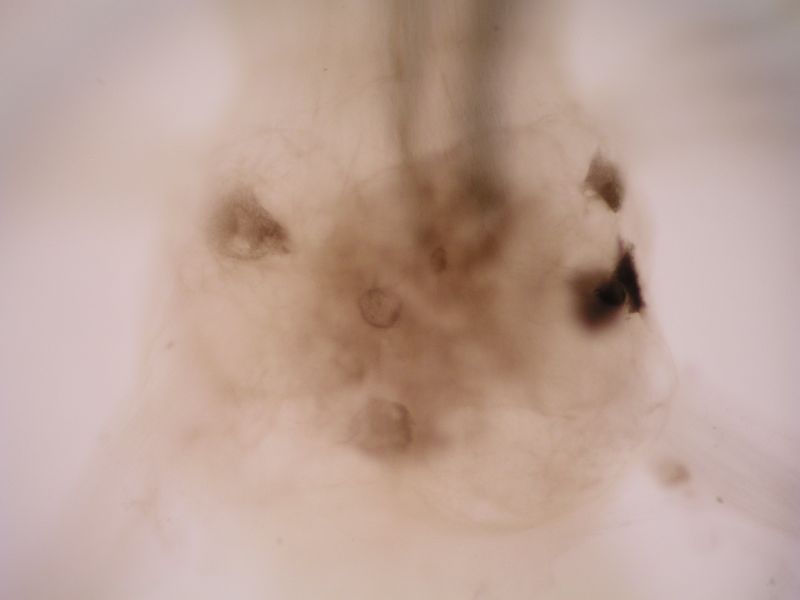

Supplement: Supplemental Information 2 — Micrographs of N3 backfills. Images have been reduced in size. [file peerj-03-1112-s003.zip › A1N3 unilateral 2009 07 09 (12).jpg]

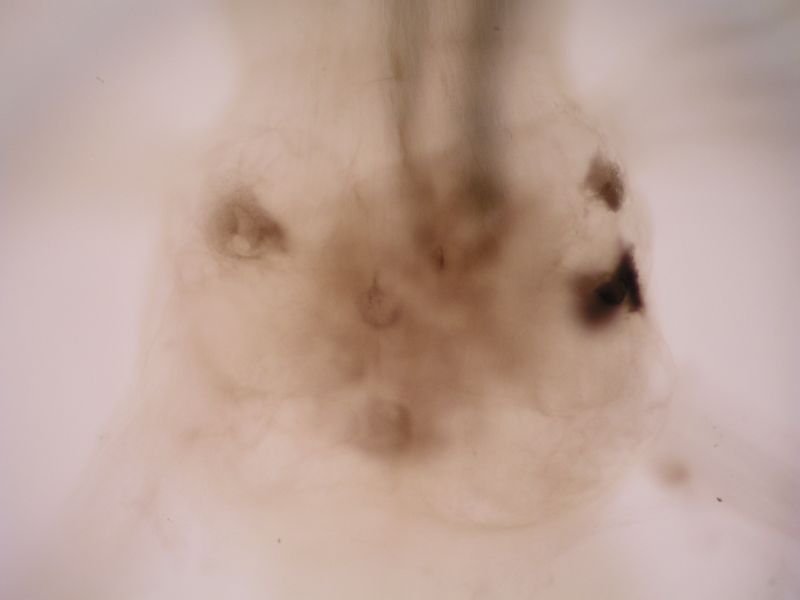

Supplement: Supplemental Information 2 — Micrographs of N3 backfills. Images have been reduced in size. [file peerj-03-1112-s003.zip › A1N3 unilateral 2009 07 09 (13).jpg]

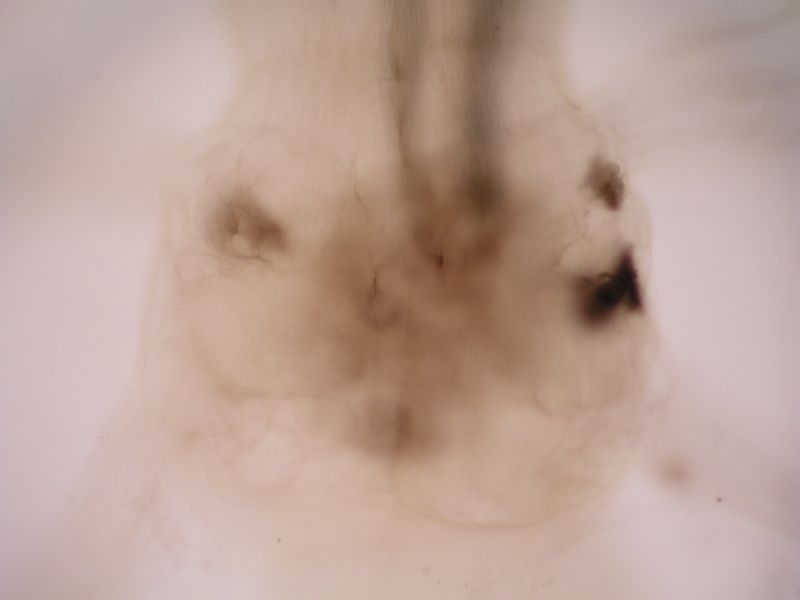

Supplement: Supplemental Information 2 — Micrographs of N3 backfills. Images have been reduced in size. [file peerj-03-1112-s003.zip › A1N3 unilateral 2009 07 09 (14).jpg]

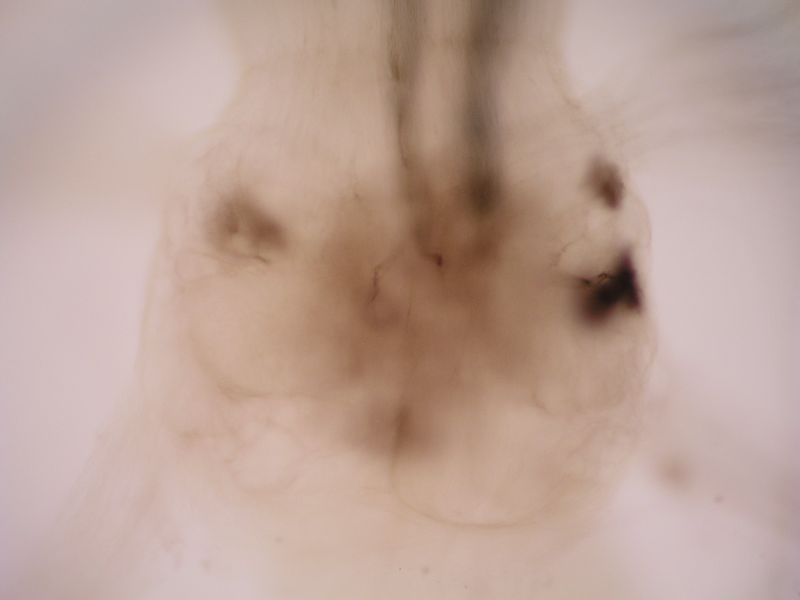

Supplement: Supplemental Information 2 — Micrographs of N3 backfills. Images have been reduced in size. [file peerj-03-1112-s003.zip › A1N3 unilateral 2009 07 09 (15).jpg]

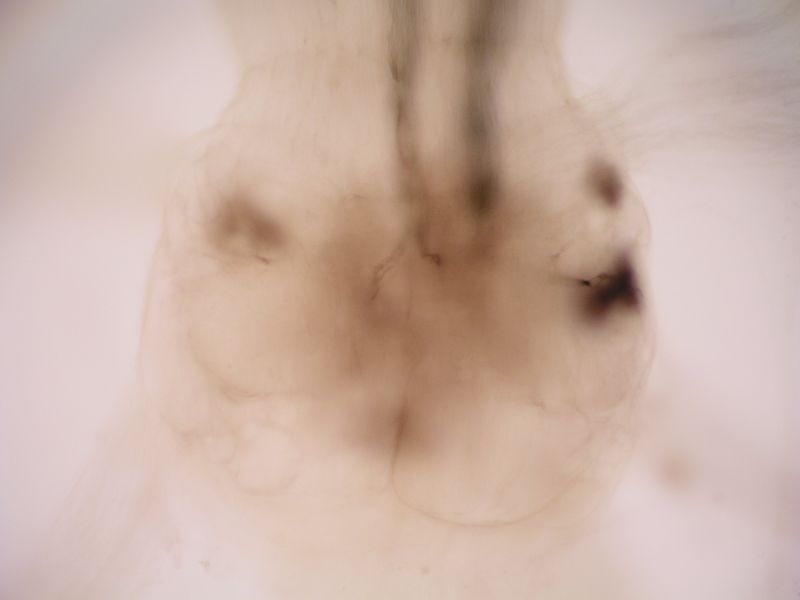

Supplement: Supplemental Information 2 — Micrographs of N3 backfills. Images have been reduced in size. [file peerj-03-1112-s003.zip › A1N3 unilateral 2009 07 09 (16).jpg]

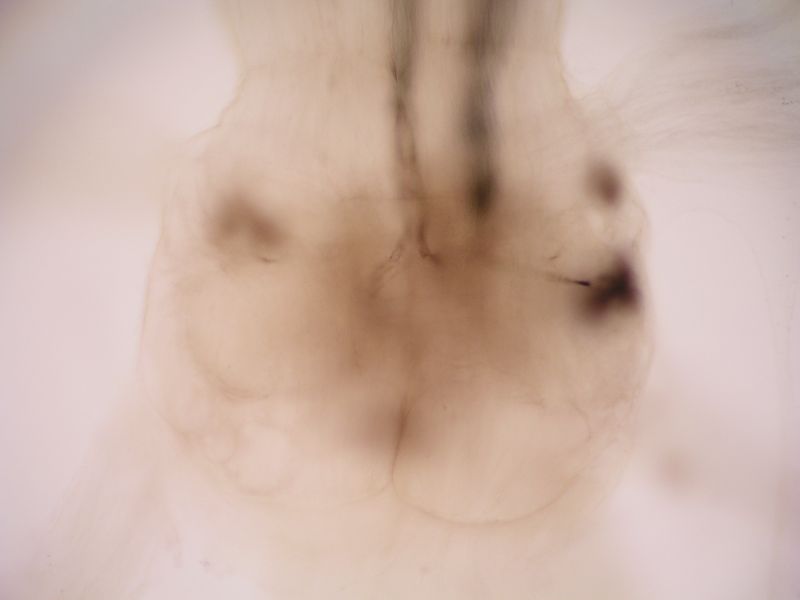

Supplement: Supplemental Information 2 — Micrographs of N3 backfills. Images have been reduced in size. [file peerj-03-1112-s003.zip › A1N3 unilateral 2009 07 09 (17).jpg]

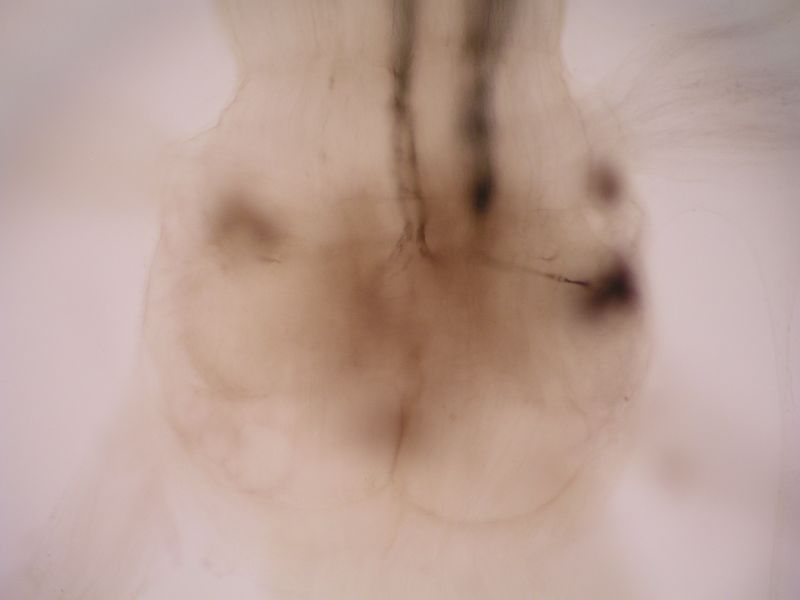

Supplement: Supplemental Information 2 — Micrographs of N3 backfills. Images have been reduced in size. [file peerj-03-1112-s003.zip › A1N3 unilateral 2009 07 09 (18).jpg]

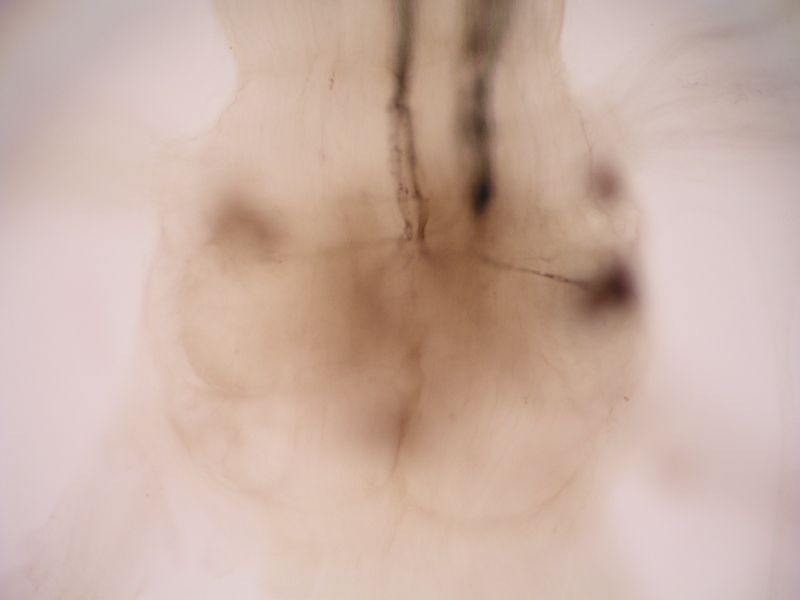

Supplement: Supplemental Information 2 — Micrographs of N3 backfills. Images have been reduced in size. [file peerj-03-1112-s003.zip › A1N3 unilateral 2009 07 09 (19).jpg]

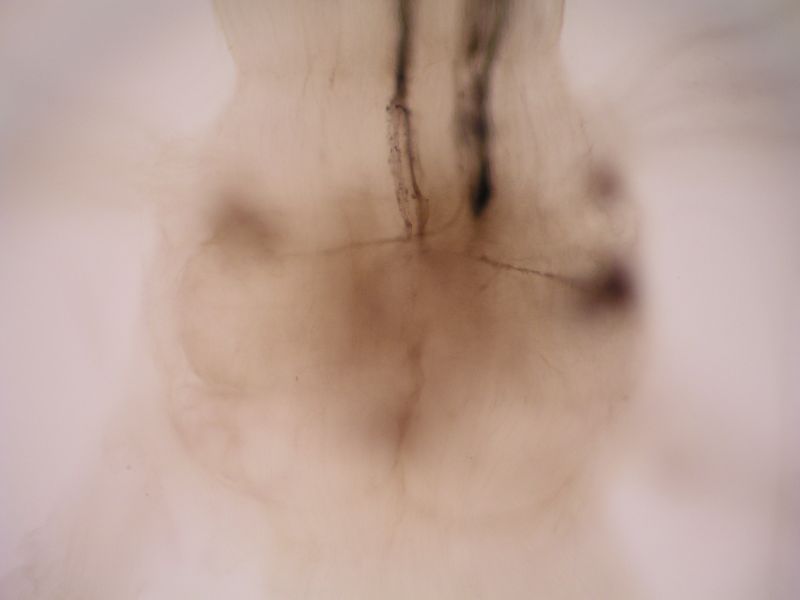

Supplement: Supplemental Information 2 — Micrographs of N3 backfills. Images have been reduced in size. [file peerj-03-1112-s003.zip › A1N3 unilateral 2009 07 09 (20).jpg]

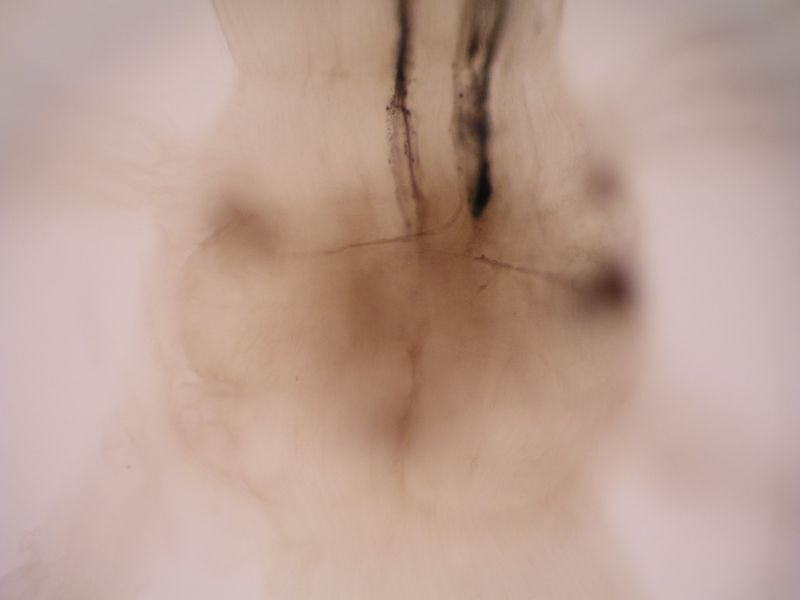

Supplement: Supplemental Information 2 — Micrographs of N3 backfills. Images have been reduced in size. [file peerj-03-1112-s003.zip › A1N3 unilateral 2009 07 09 (21).jpg]

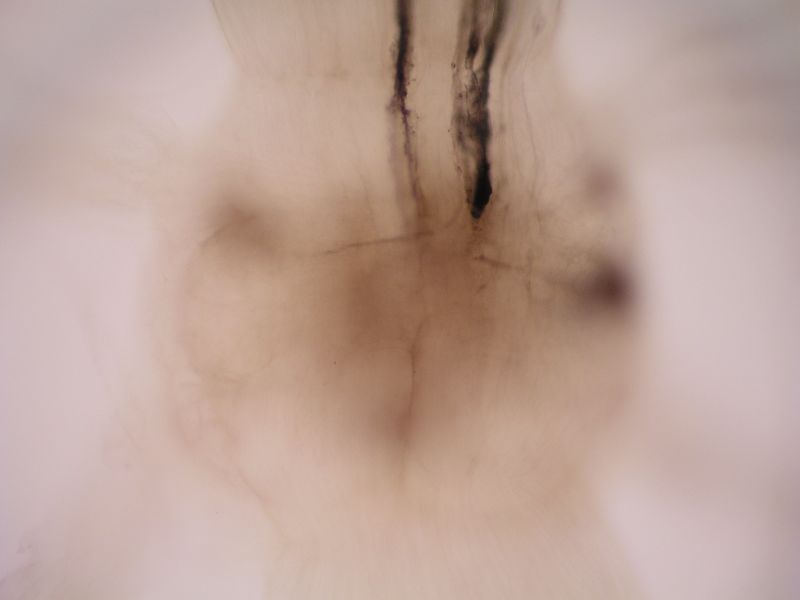

Supplement: Supplemental Information 2 — Micrographs of N3 backfills. Images have been reduced in size. [file peerj-03-1112-s003.zip › A1N3 unilateral 2009 07 09 (22).jpg]

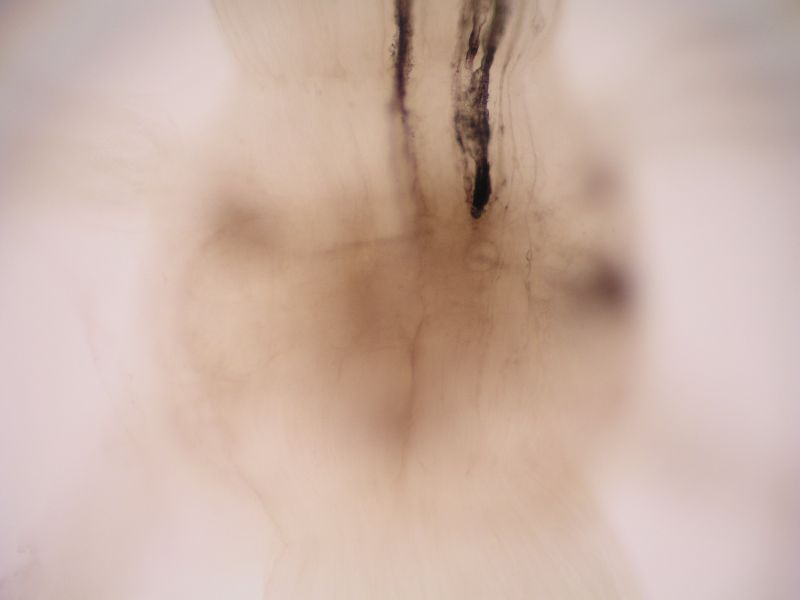

Supplement: Supplemental Information 2 — Micrographs of N3 backfills. Images have been reduced in size. [file peerj-03-1112-s003.zip › A1N3 unilateral 2009 07 09 (23).jpg]

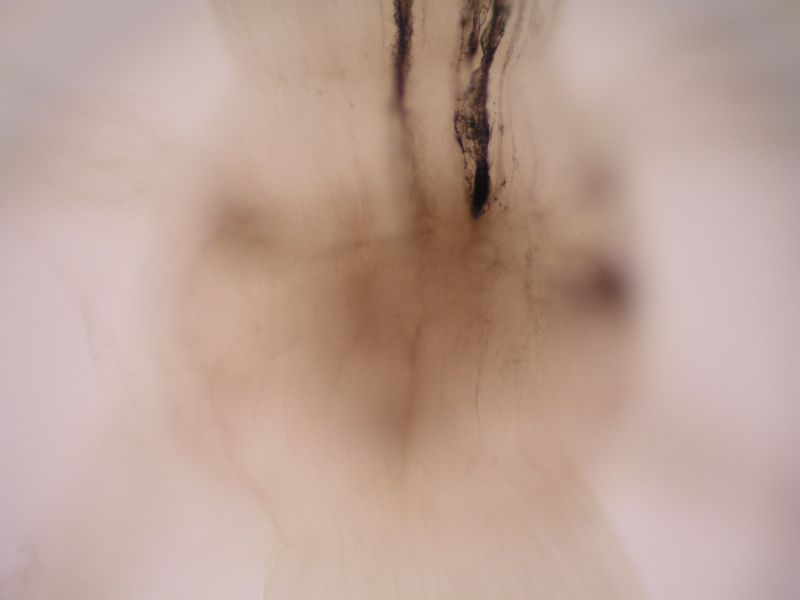

Supplement: Supplemental Information 2 — Micrographs of N3 backfills. Images have been reduced in size. [file peerj-03-1112-s003.zip › A1N3 unilateral 2009 07 09 (24).jpg]

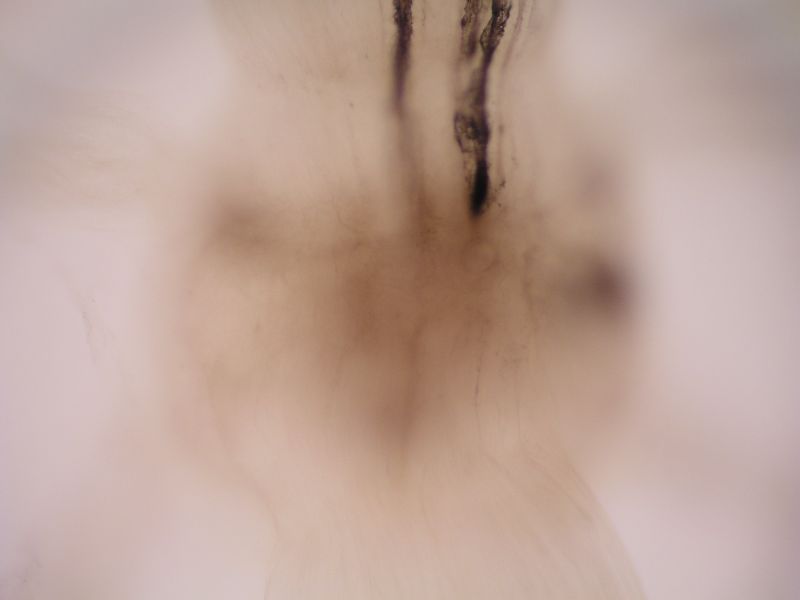

Supplement: Supplemental Information 2 — Micrographs of N3 backfills. Images have been reduced in size. [file peerj-03-1112-s003.zip › A1N3 unilateral 2009 07 09 (25).jpg]

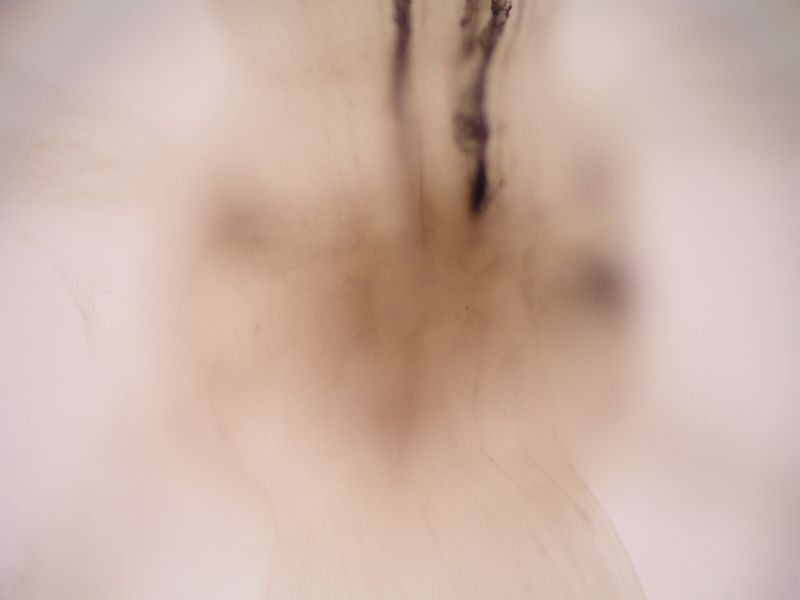

Supplement: Supplemental Information 2 — Micrographs of N3 backfills. Images have been reduced in size. [file peerj-03-1112-s003.zip › A1N3 unilateral 2009 07 09 (26).jpg]

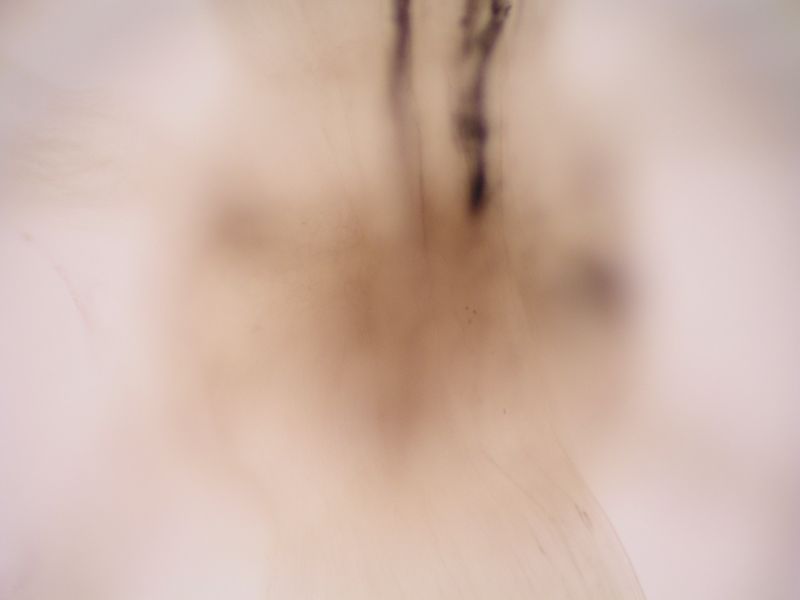

Supplement: Supplemental Information 2 — Micrographs of N3 backfills. Images have been reduced in size. [file peerj-03-1112-s003.zip › A1N3 unilateral 2009 07 09.jpg]

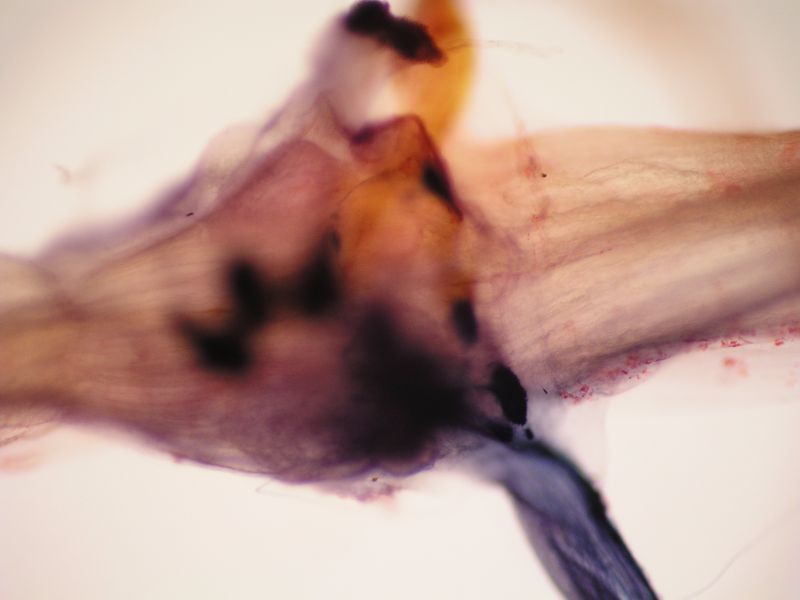

Supplement: Supplemental Information 2 — Micrographs of N3 backfills. Images have been reduced in size. [file peerj-03-1112-s003.zip › A2 N2 P1010070.jpg]

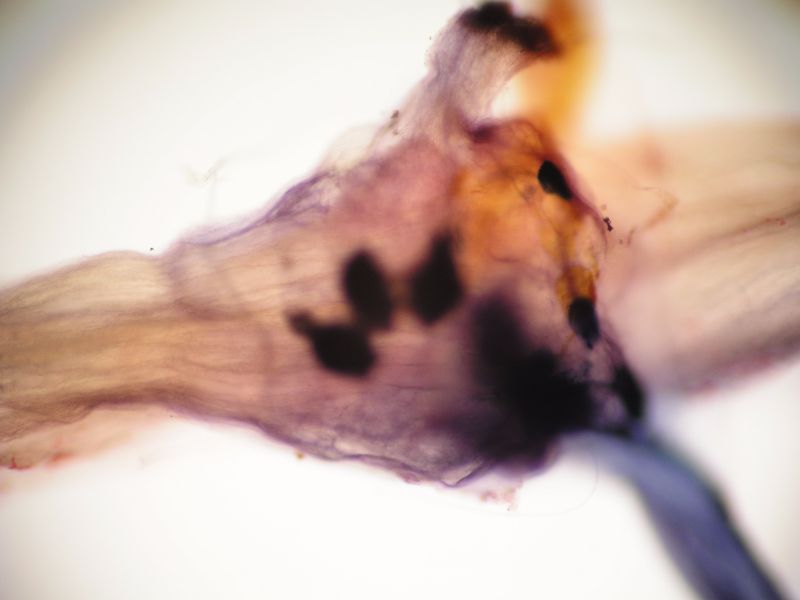

Supplement: Supplemental Information 2 — Micrographs of N3 backfills. Images have been reduced in size. [file peerj-03-1112-s003.zip › A2 N2 P1010071.jpg]

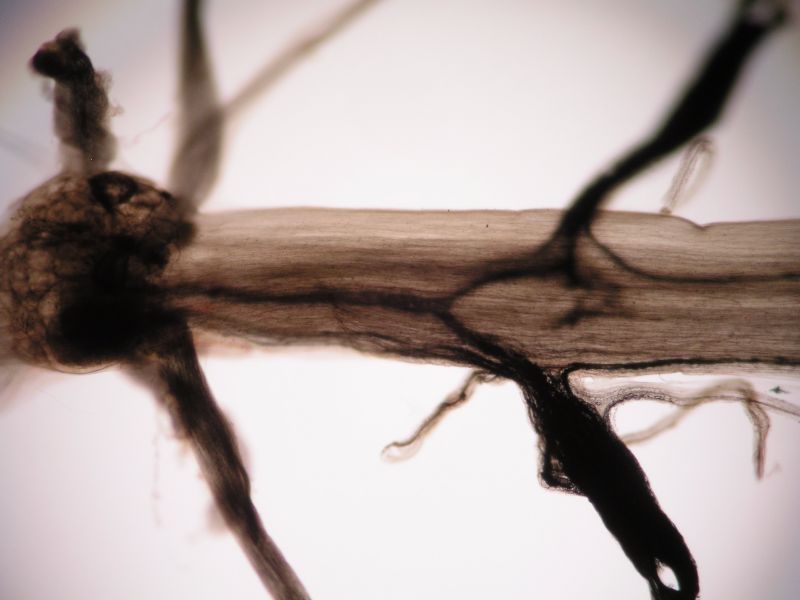

Supplement: Supplemental Information 2 — Micrographs of N3 backfills. Images have been reduced in size. [file peerj-03-1112-s003.zip › A2N3 2009 07 08 (1).jpg]

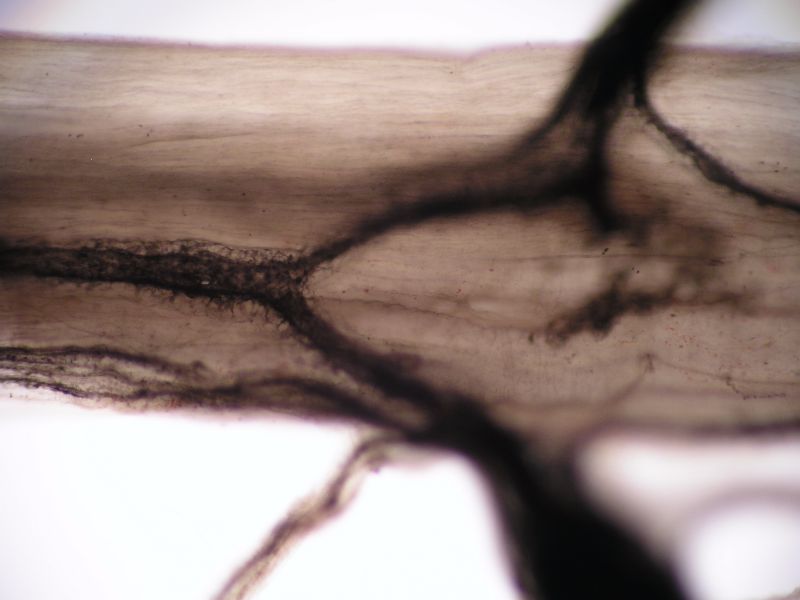

Supplement: Supplemental Information 2 — Micrographs of N3 backfills. Images have been reduced in size. [file peerj-03-1112-s003.zip › A2N3 2009 07 08 (2).jpg]

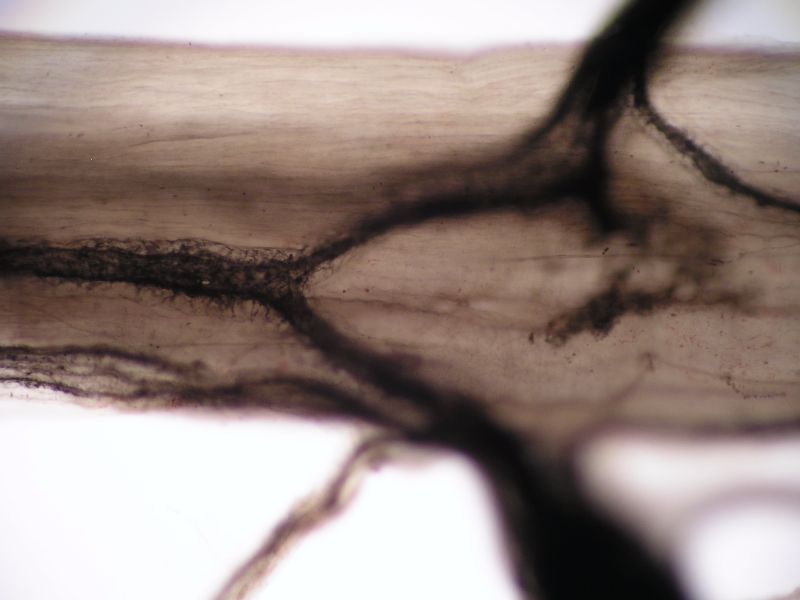

Supplement: Supplemental Information 2 — Micrographs of N3 backfills. Images have been reduced in size. [file peerj-03-1112-s003.zip › A2N3 2009 07 08 (3).jpg]

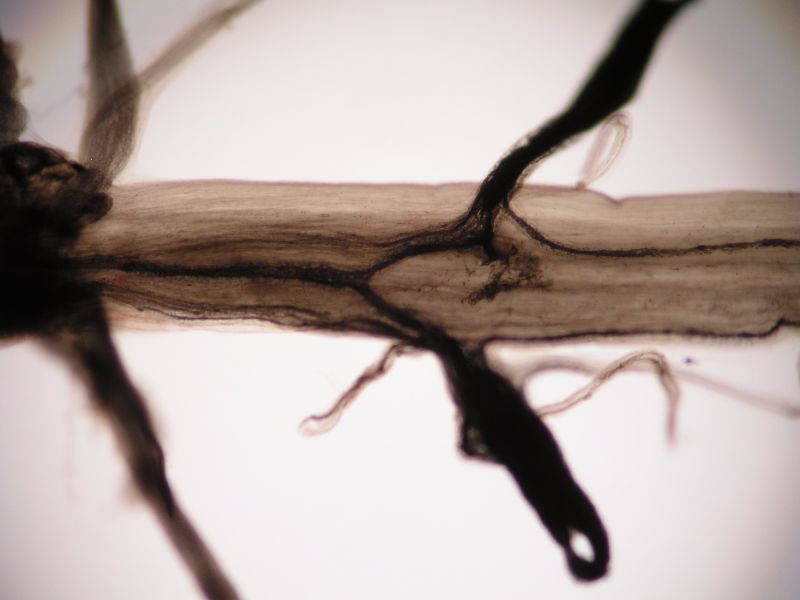

Supplement: Supplemental Information 2 — Micrographs of N3 backfills. Images have been reduced in size. [file peerj-03-1112-s003.zip › A2N3 2009 07 08 (4).jpg]

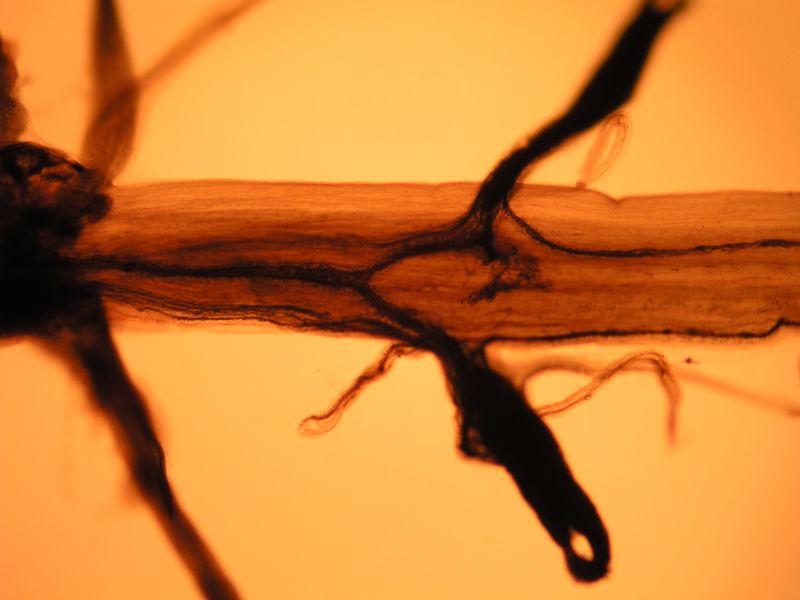

Supplement: Supplemental Information 2 — Micrographs of N3 backfills. Images have been reduced in size. [file peerj-03-1112-s003.zip › A2N3 2009 07 08 (5).jpg]

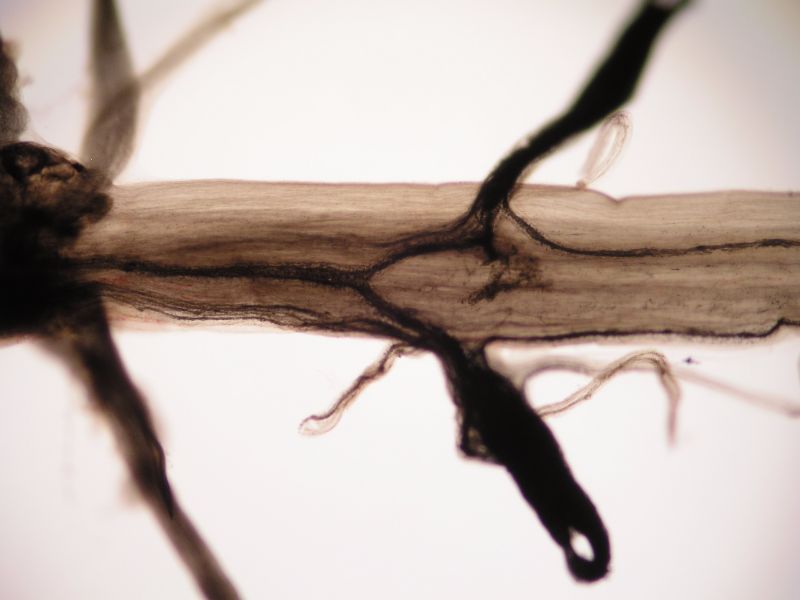

Supplement: Supplemental Information 2 — Micrographs of N3 backfills. Images have been reduced in size. [file peerj-03-1112-s003.zip › A2N3 2009 07 08 (6).jpg]

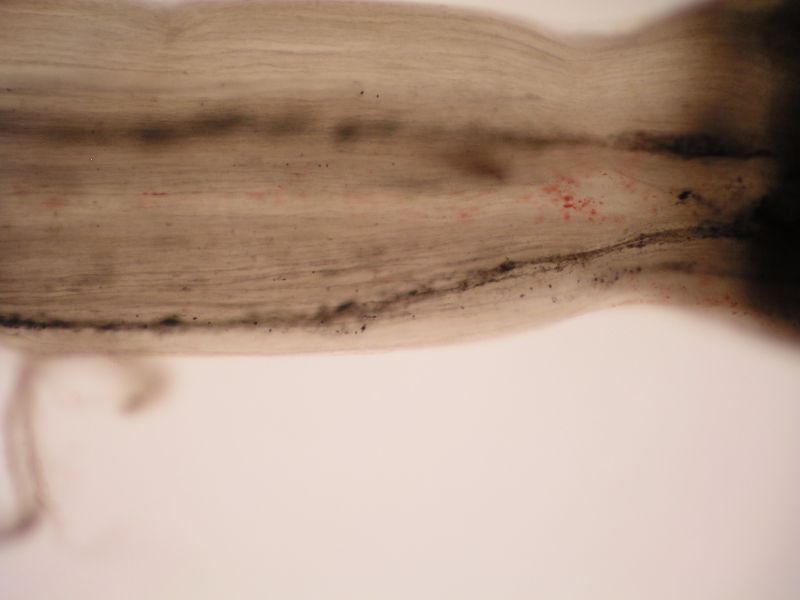

Supplement: Supplemental Information 2 — Micrographs of N3 backfills. Images have been reduced in size. [file peerj-03-1112-s003.zip › A2N3 2009 07 08.jpg]

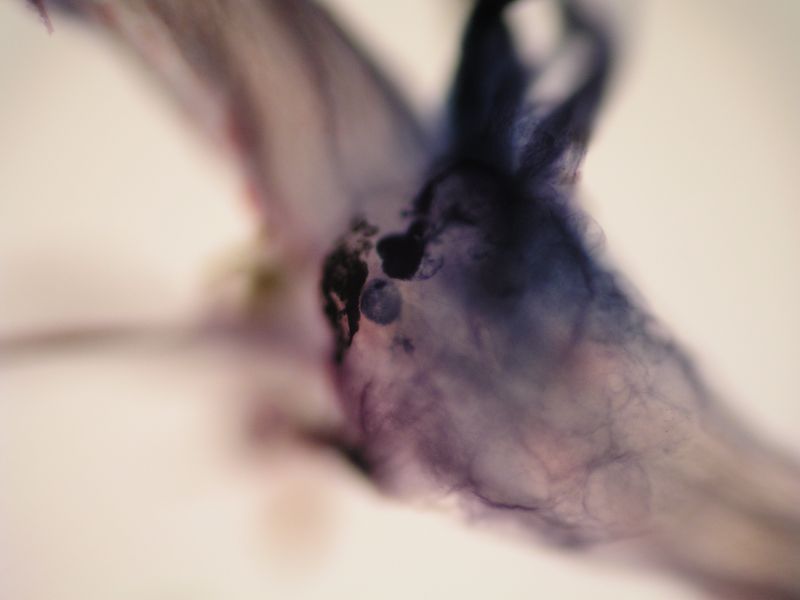

Supplement: Supplemental Information 2 — Micrographs of N3 backfills. Images have been reduced in size. [file peerj-03-1112-s003.zip › A3 N2 P1010072.jpg]

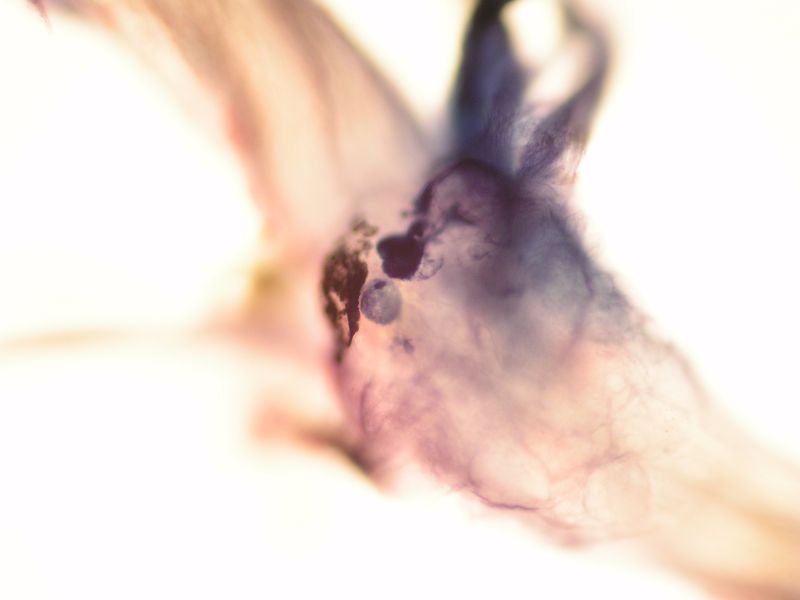

Supplement: Supplemental Information 2 — Micrographs of N3 backfills. Images have been reduced in size. [file peerj-03-1112-s003.zip › A3 N2 P1010073.jpg]

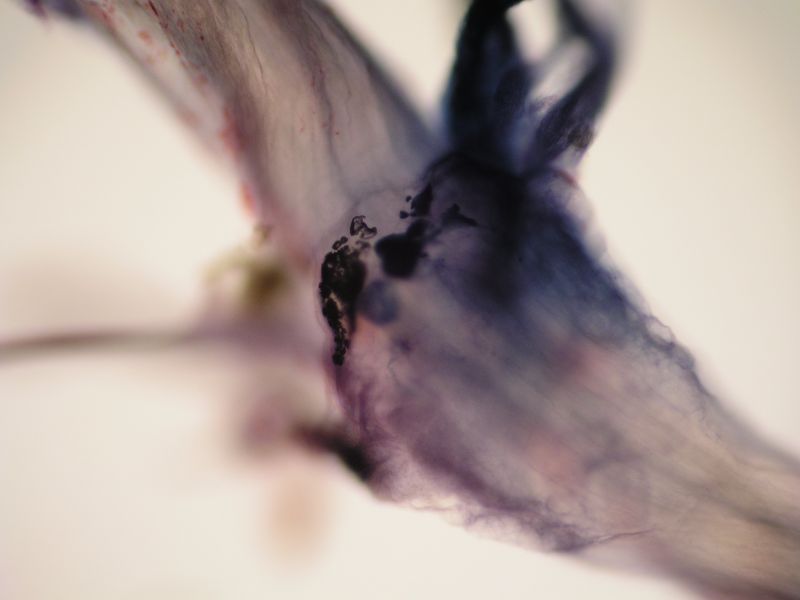

Supplement: Supplemental Information 2 — Micrographs of N3 backfills. Images have been reduced in size. [file peerj-03-1112-s003.zip › A3 N2 P1010074.jpg]

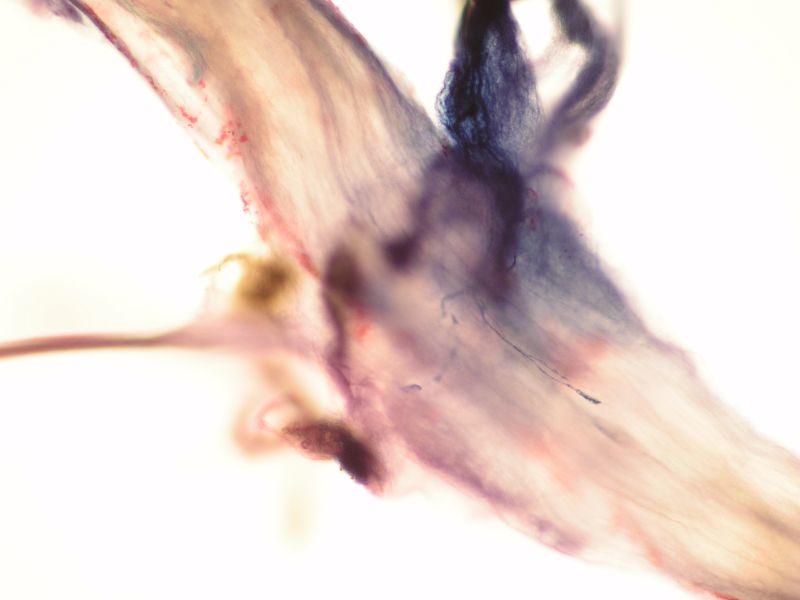

Supplement: Supplemental Information 2 — Micrographs of N3 backfills. Images have been reduced in size. [file peerj-03-1112-s003.zip › A3 N2 P1010075.jpg]

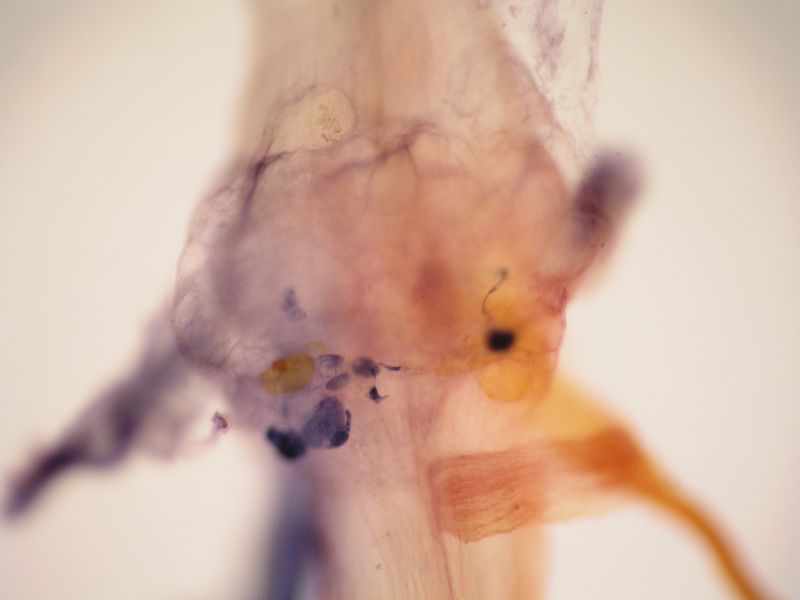

Supplement: Supplemental Information 2 — Micrographs of N3 backfills. Images have been reduced in size. [file peerj-03-1112-s003.zip › A3 N2 P1010077.jpg]

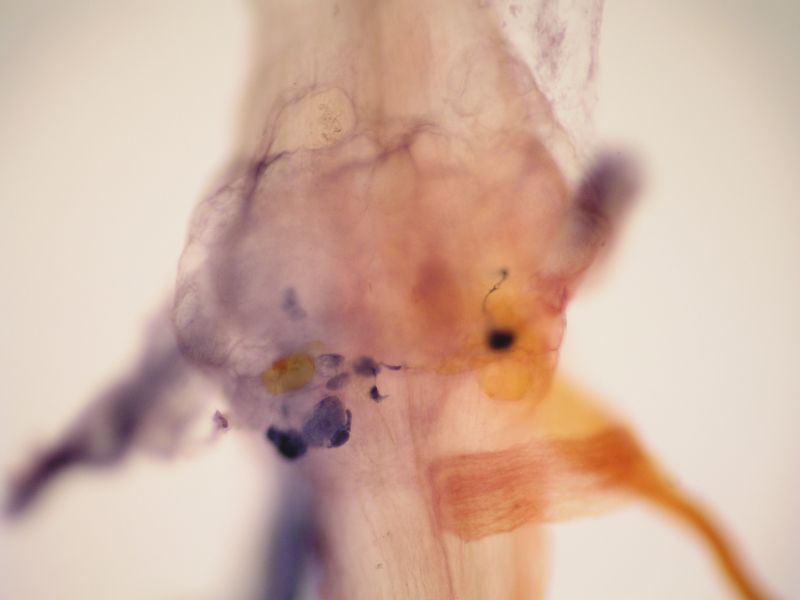

Supplement: Supplemental Information 2 — Micrographs of N3 backfills. Images have been reduced in size. [file peerj-03-1112-s003.zip › A3 N2 P1010078.jpg]

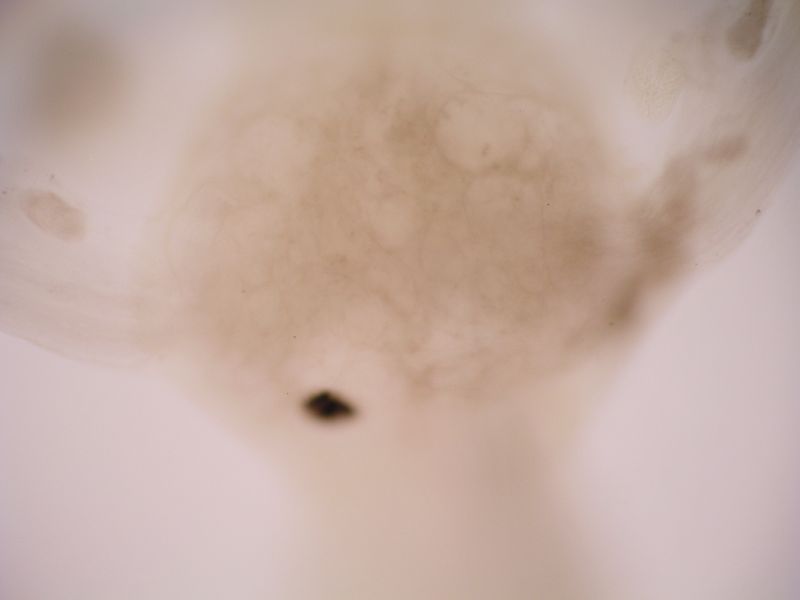

Supplement: Supplemental Information 2 — Micrographs of N3 backfills. Images have been reduced in size. [file peerj-03-1112-s003.zip › A3N2 2009 07 10 (1).jpg]

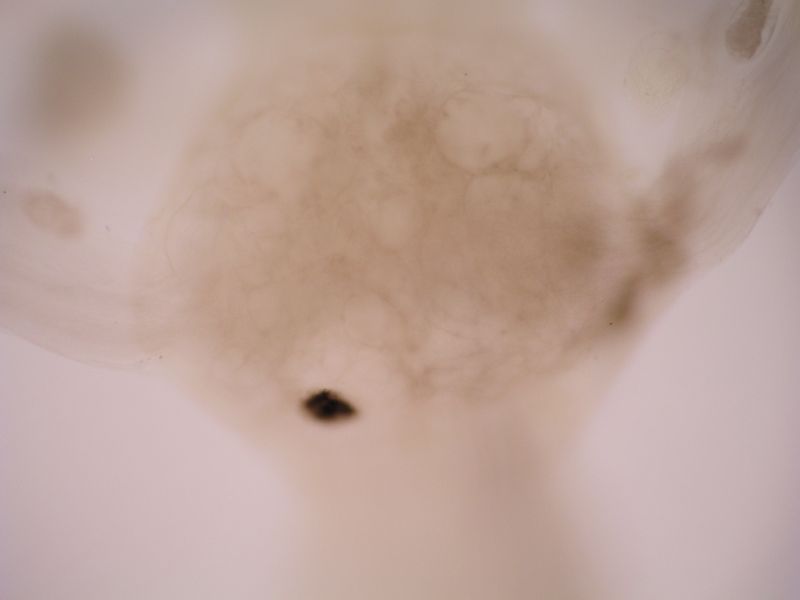

Supplement: Supplemental Information 2 — Micrographs of N3 backfills. Images have been reduced in size. [file peerj-03-1112-s003.zip › A3N2 2009 07 10 (2).jpg]

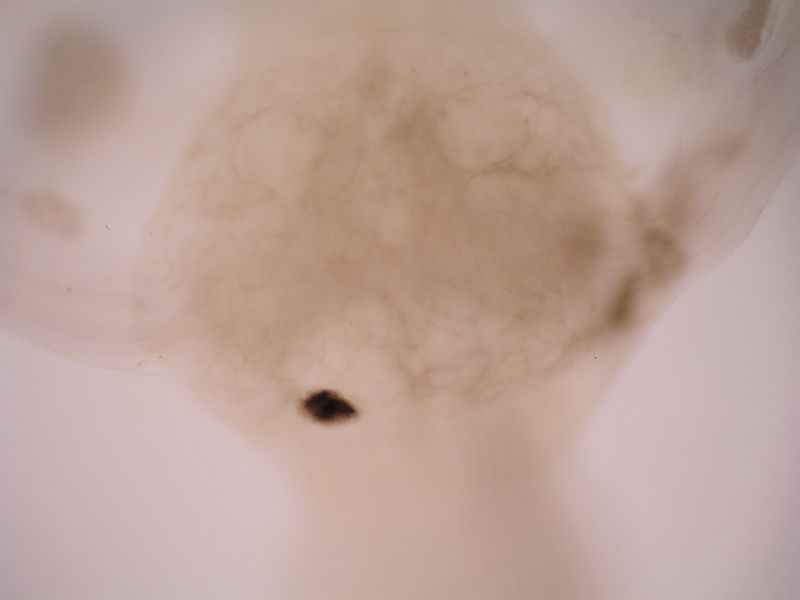

Supplement: Supplemental Information 2 — Micrographs of N3 backfills. Images have been reduced in size. [file peerj-03-1112-s003.zip › A3N2 2009 07 10 (3).jpg]

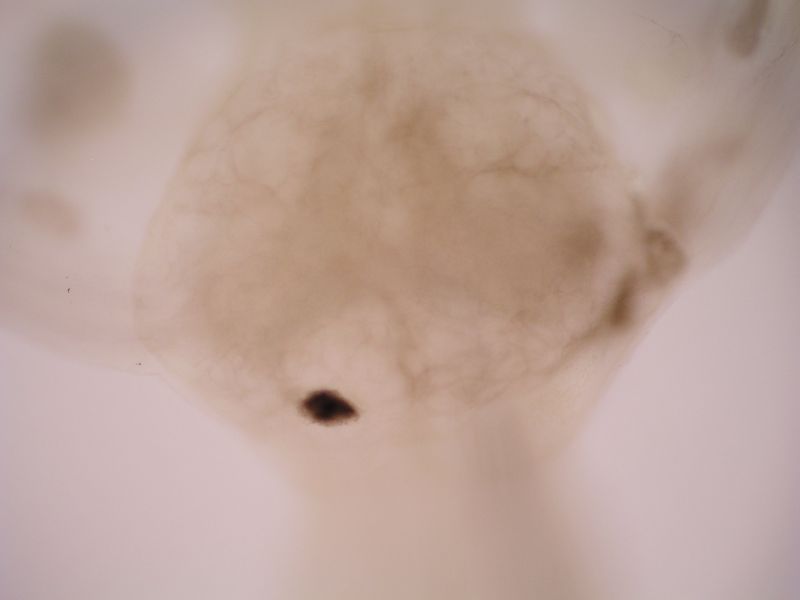

Supplement: Supplemental Information 2 — Micrographs of N3 backfills. Images have been reduced in size. [file peerj-03-1112-s003.zip › A3N2 2009 07 10 (4).jpg]

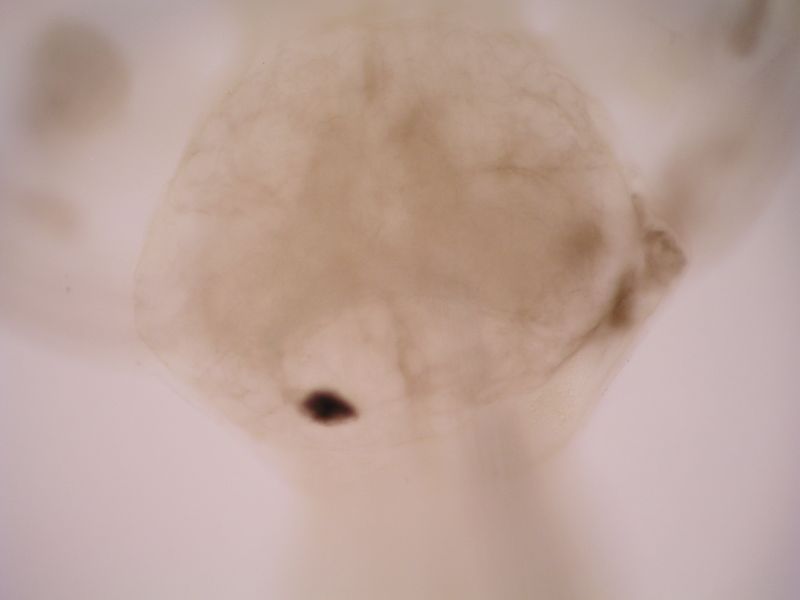

Supplement: Supplemental Information 2 — Micrographs of N3 backfills. Images have been reduced in size. [file peerj-03-1112-s003.zip › A3N2 2009 07 10 (5).jpg]

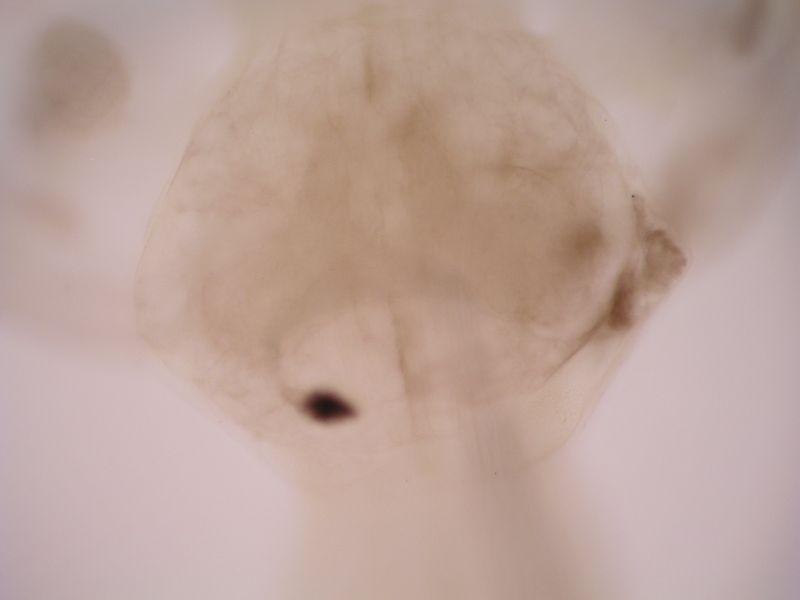

Supplement: Supplemental Information 2 — Micrographs of N3 backfills. Images have been reduced in size. [file peerj-03-1112-s003.zip › A3N2 2009 07 10 (6).jpg]

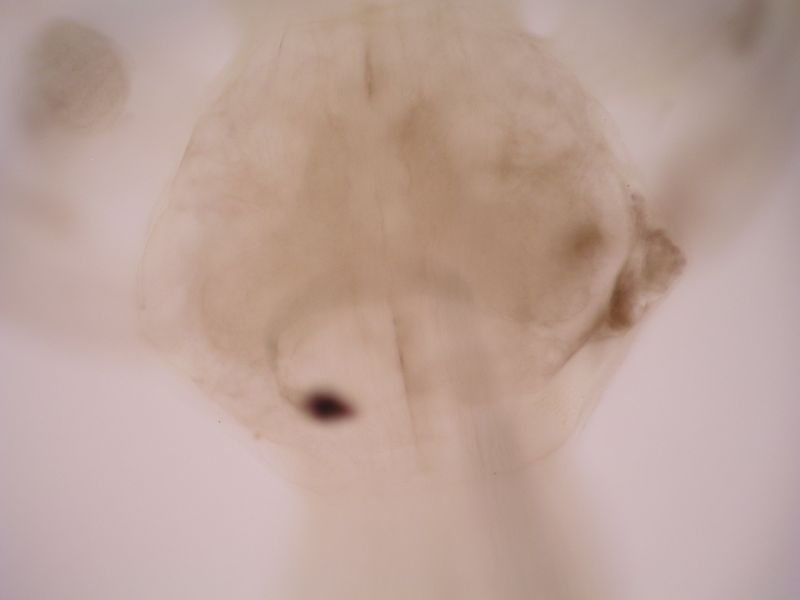

Supplement: Supplemental Information 2 — Micrographs of N3 backfills. Images have been reduced in size. [file peerj-03-1112-s003.zip › A3N2 2009 07 10 (7).jpg]

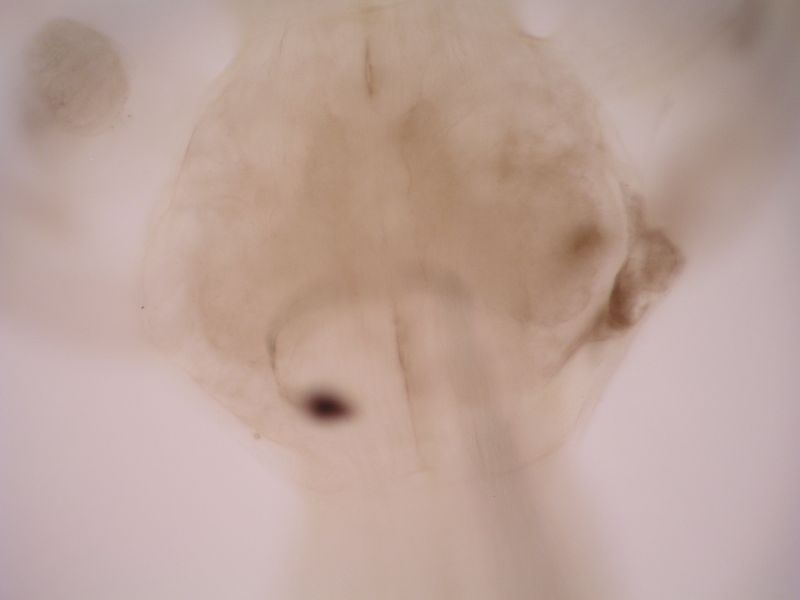

Supplement: Supplemental Information 2 — Micrographs of N3 backfills. Images have been reduced in size. [file peerj-03-1112-s003.zip › A3N2 2009 07 10 (8).jpg]

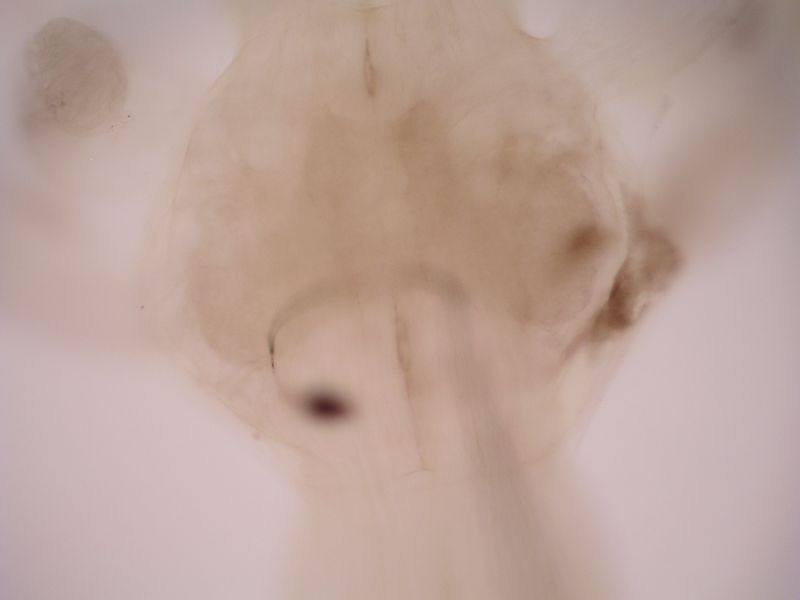

Supplement: Supplemental Information 2 — Micrographs of N3 backfills. Images have been reduced in size. [file peerj-03-1112-s003.zip › A3N2 2009 07 10 (9).jpg]

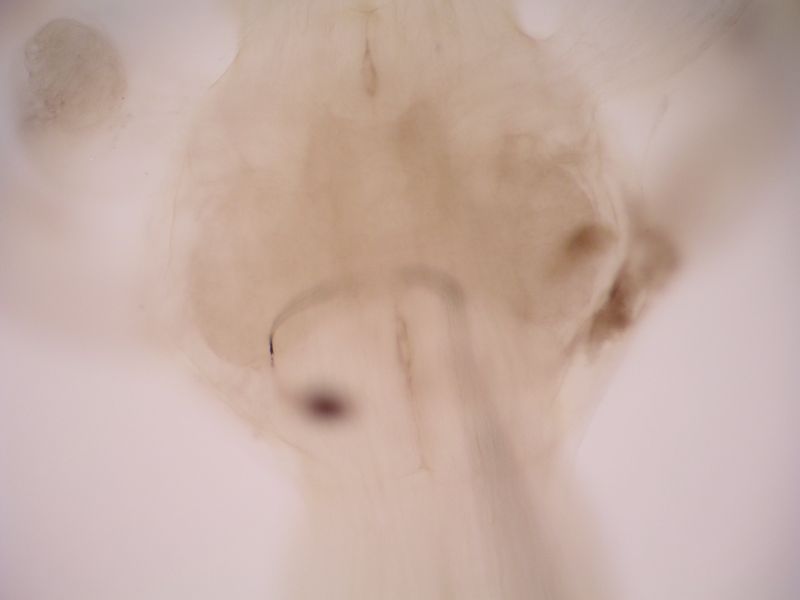

Supplement: Supplemental Information 2 — Micrographs of N3 backfills. Images have been reduced in size. [file peerj-03-1112-s003.zip › A3N2 2009 07 10 (10).jpg]

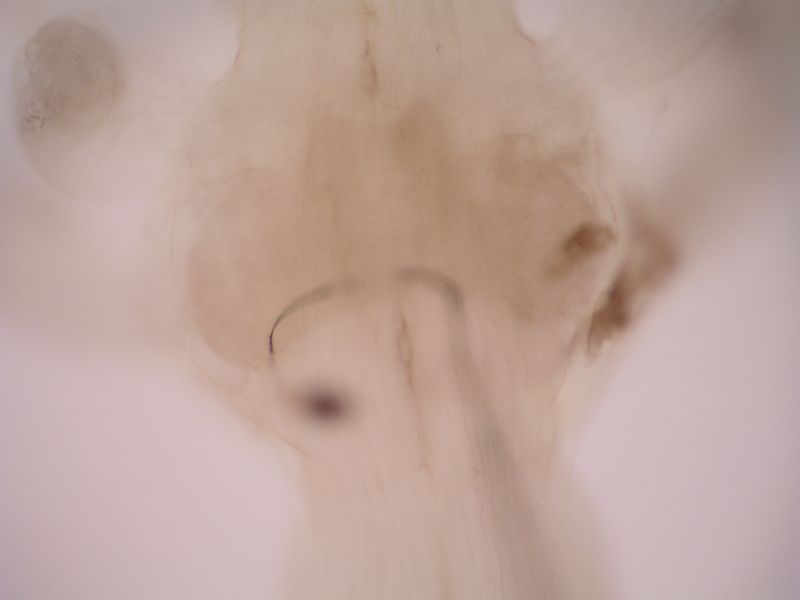

Supplement: Supplemental Information 2 — Micrographs of N3 backfills. Images have been reduced in size. [file peerj-03-1112-s003.zip › A3N2 2009 07 10 (11).jpg]

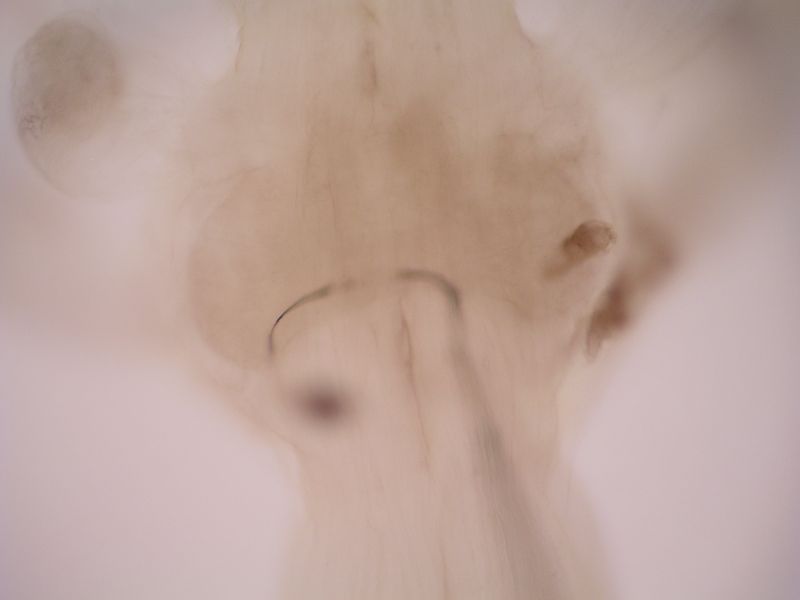

Supplement: Supplemental Information 2 — Micrographs of N3 backfills. Images have been reduced in size. [file peerj-03-1112-s003.zip › A3N2 2009 07 10 (12).jpg]

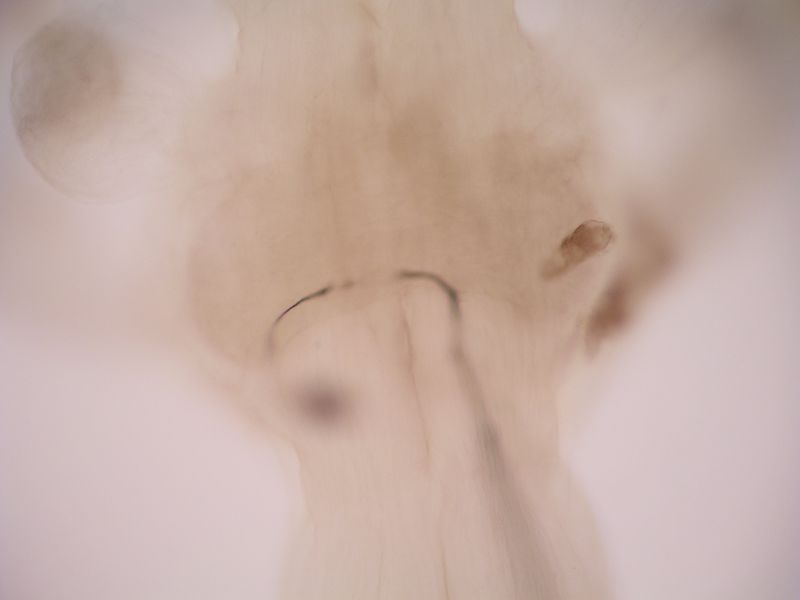

Supplement: Supplemental Information 2 — Micrographs of N3 backfills. Images have been reduced in size. [file peerj-03-1112-s003.zip › A3N2 2009 07 10 (13).jpg]

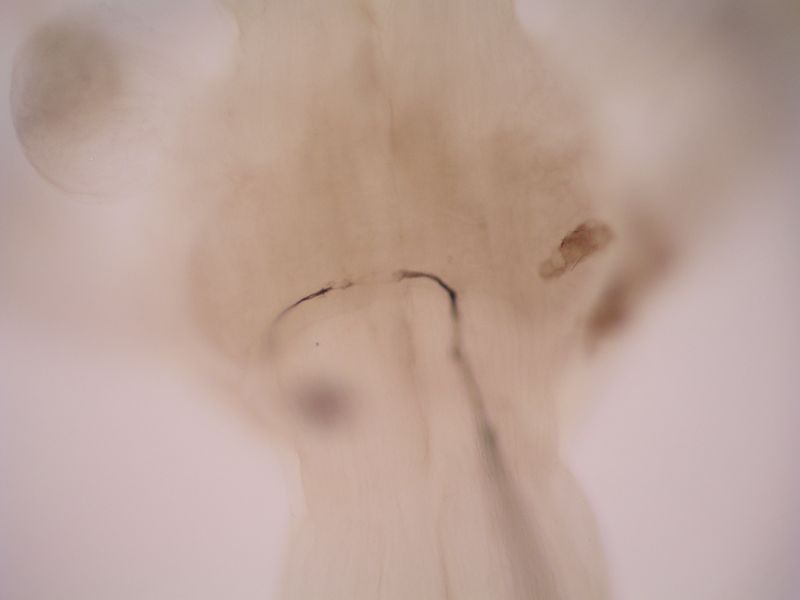

Supplement: Supplemental Information 2 — Micrographs of N3 backfills. Images have been reduced in size. [file peerj-03-1112-s003.zip › A3N2 2009 07 10 (14).jpg]

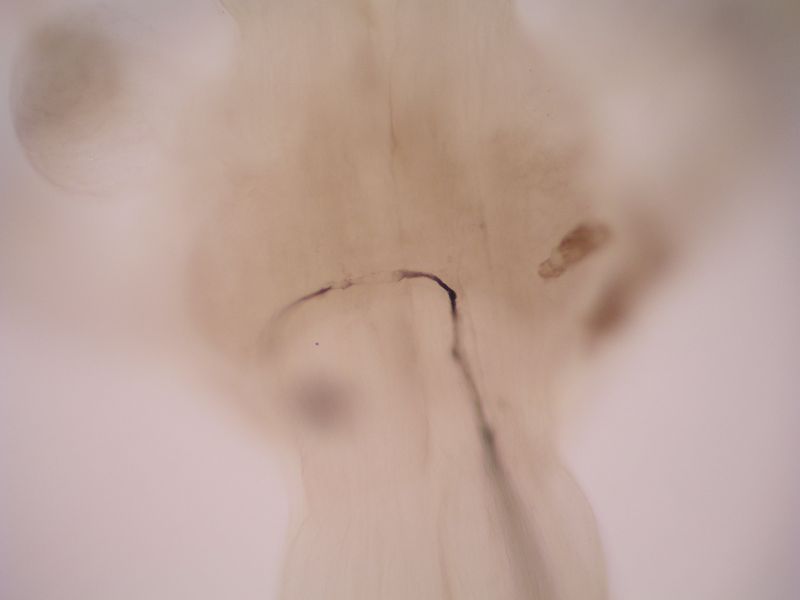

Supplement: Supplemental Information 2 — Micrographs of N3 backfills. Images have been reduced in size. [file peerj-03-1112-s003.zip › A3N2 2009 07 10 (15).jpg]

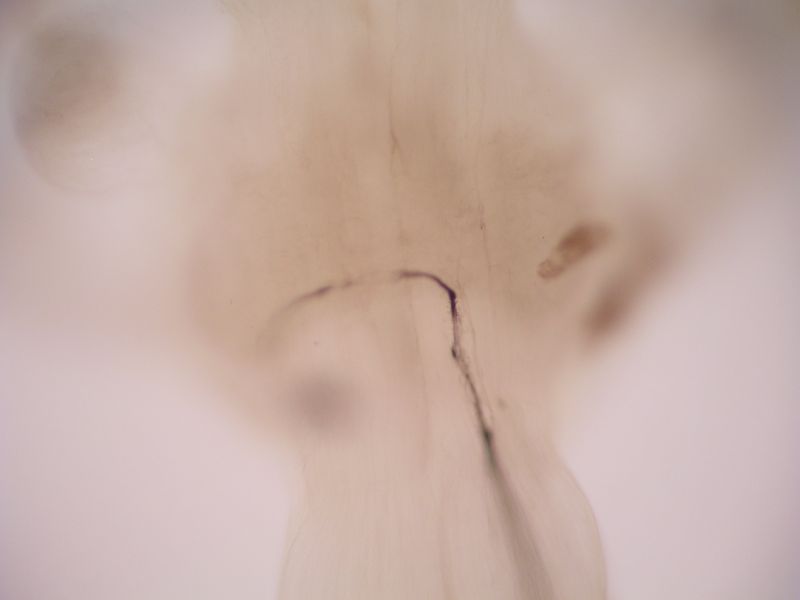

Supplement: Supplemental Information 2 — Micrographs of N3 backfills. Images have been reduced in size. [file peerj-03-1112-s003.zip › A3N2 2009 07 10 (16).jpg]

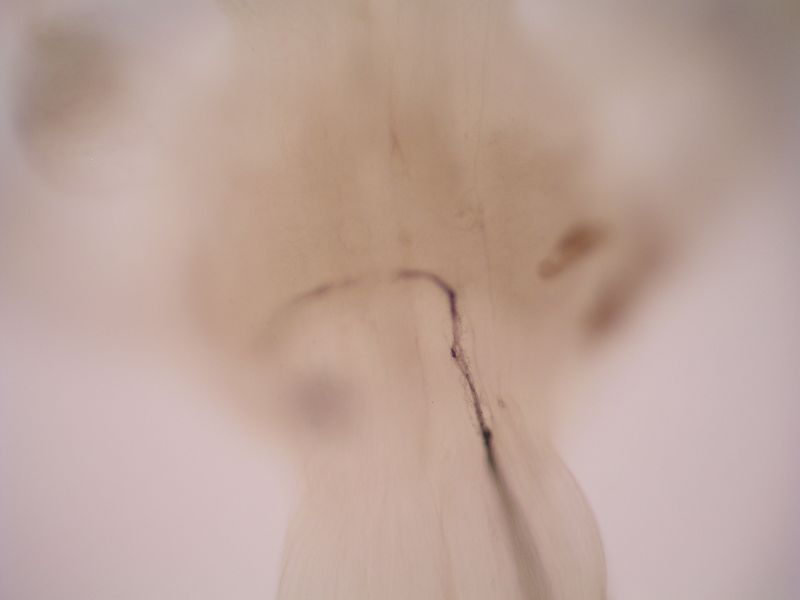

Supplement: Supplemental Information 2 — Micrographs of N3 backfills. Images have been reduced in size. [file peerj-03-1112-s003.zip › A3N2 2009 07 10 (17).jpg]

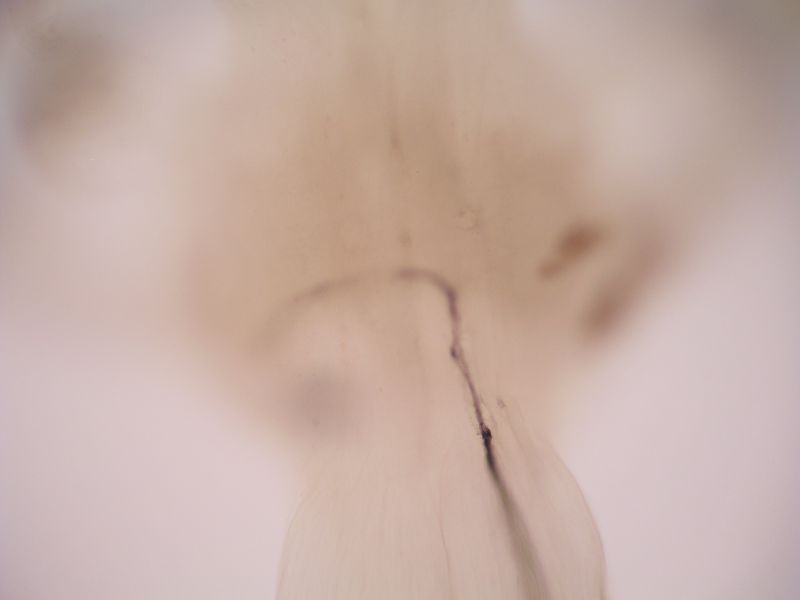

Supplement: Supplemental Information 2 — Micrographs of N3 backfills. Images have been reduced in size. [file peerj-03-1112-s003.zip › A3N2 2009 07 10 (18).jpg]

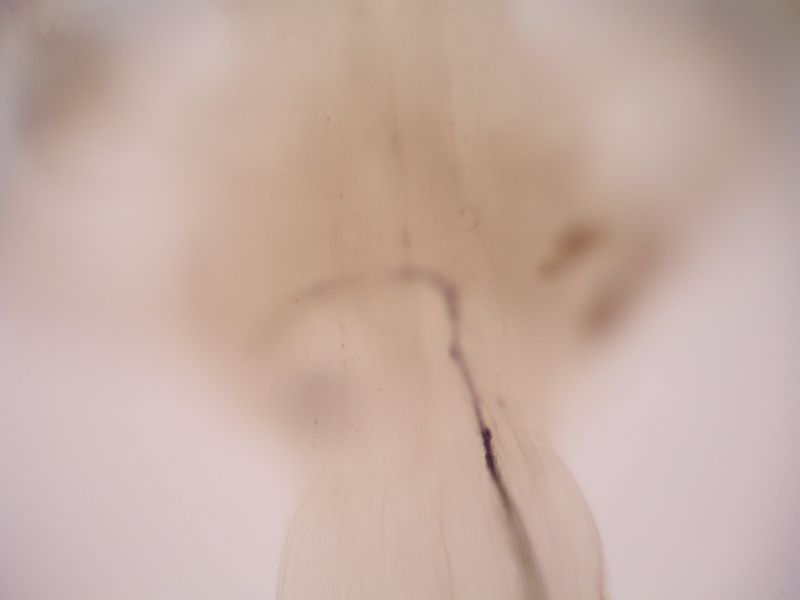

Supplement: Supplemental Information 2 — Micrographs of N3 backfills. Images have been reduced in size. [file peerj-03-1112-s003.zip › A3N2 2009 07 10 (19).jpg]

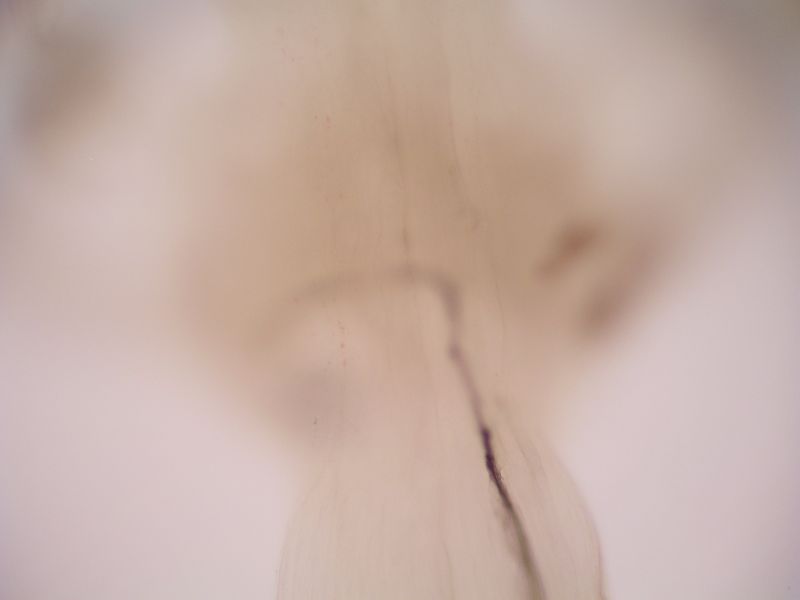

Supplement: Supplemental Information 2 — Micrographs of N3 backfills. Images have been reduced in size. [file peerj-03-1112-s003.zip › A3N2 2009 07 10 (20).jpg]

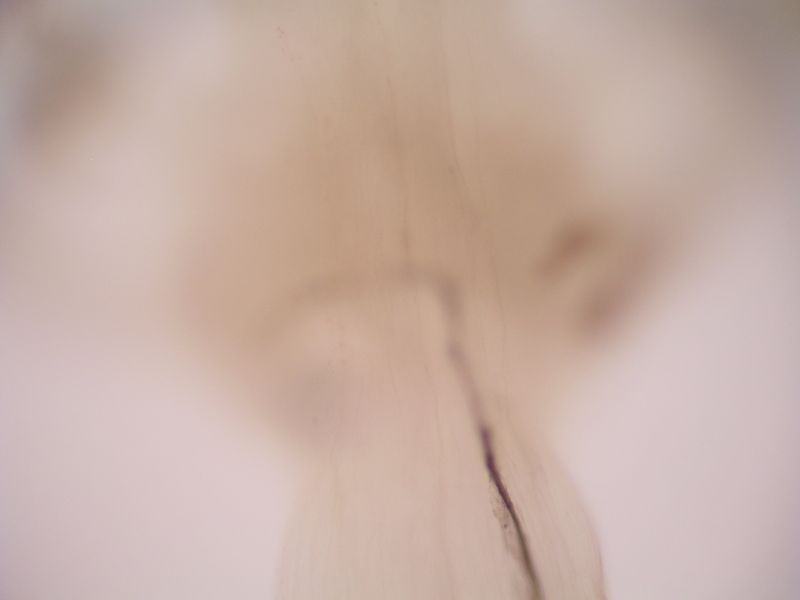

Supplement: Supplemental Information 2 — Micrographs of N3 backfills. Images have been reduced in size. [file peerj-03-1112-s003.zip › A3N2 2009 07 10 (21).jpg]

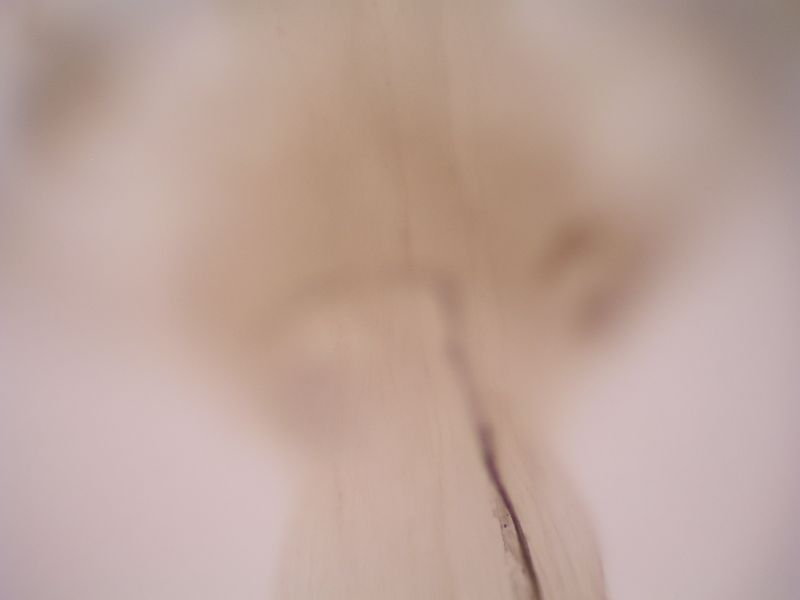

Supplement: Supplemental Information 2 — Micrographs of N3 backfills. Images have been reduced in size. [file peerj-03-1112-s003.zip › A3N2 2009 07 10 (22).jpg]
