# Supplementary figures and images for: Motor neurons in the escape response circuit of white shrimp (Litopenaeus setiferus) (part 3 of 4)
Source: PeerJ. 2015 Jul 21;3:e1112. doi: 10.7717/peerj.1112 (PMC4517965; doi:10.7717/peerj.1112)

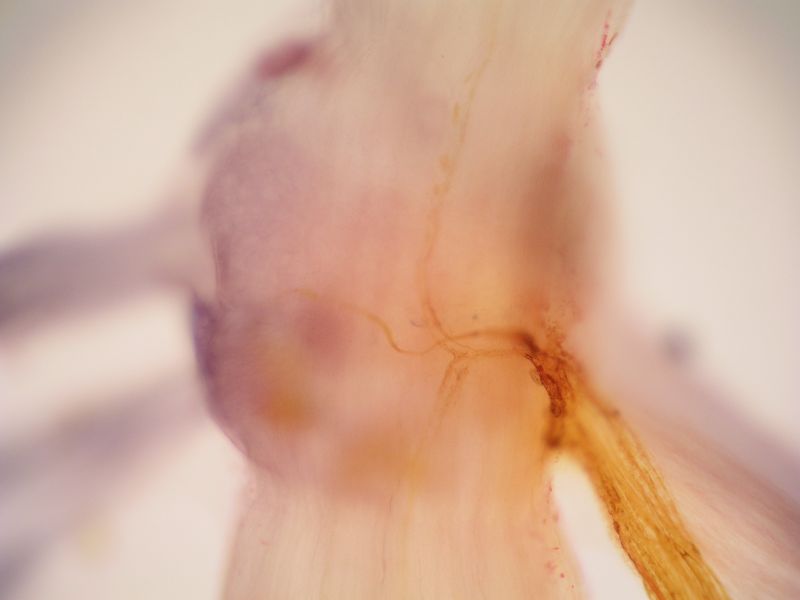

Supplement: Supplemental Information 2 — Micrographs of N3 backfills. Images have been reduced in size. [file peerj-03-1112-s003.zip › A1 N2 P1010066.jpg]

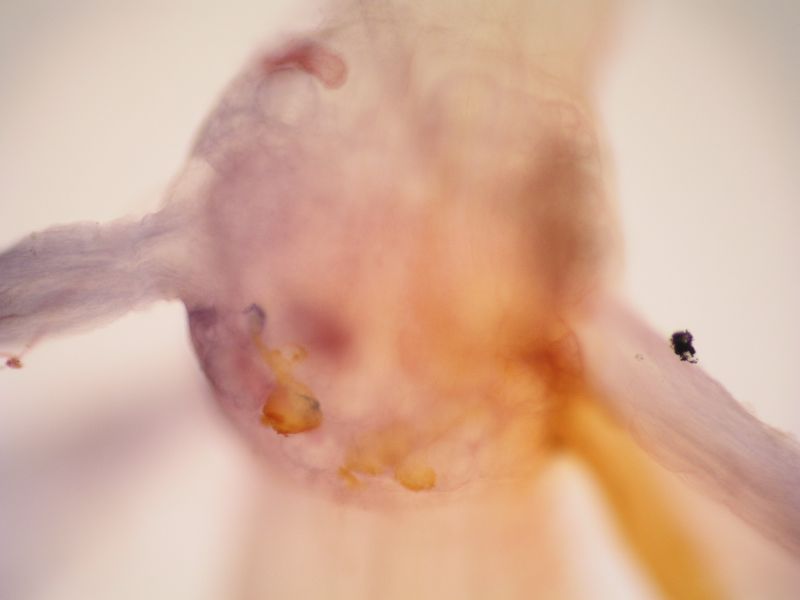

Supplement: Supplemental Information 2 — Micrographs of N3 backfills. Images have been reduced in size. [file peerj-03-1112-s003.zip › A1 N2 P1010067.jpg]

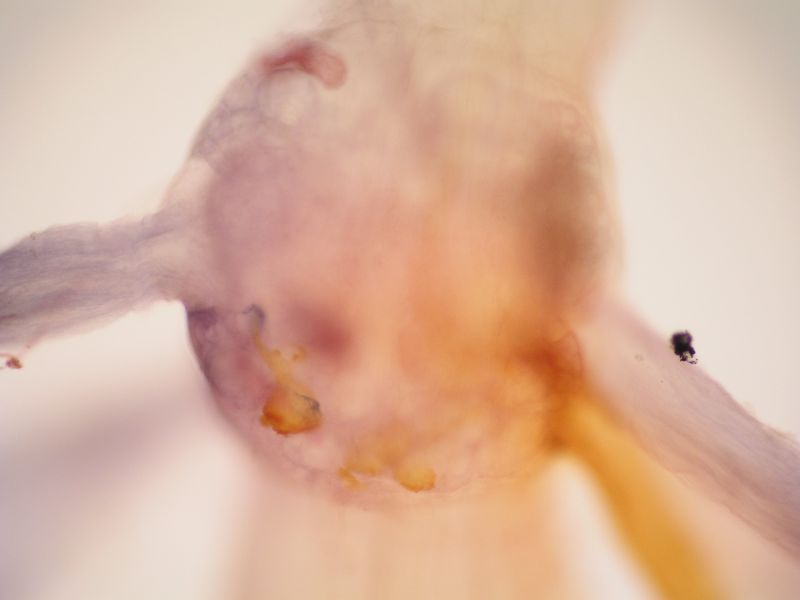

Supplement: Supplemental Information 2 — Micrographs of N3 backfills. Images have been reduced in size. [file peerj-03-1112-s003.zip › A1 N2 P1010068.jpg]

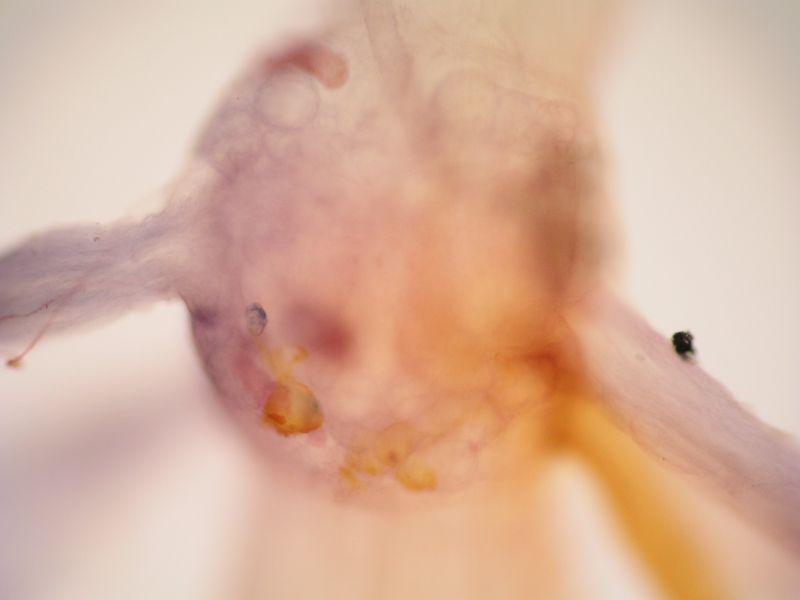

Supplement: Supplemental Information 2 — Micrographs of N3 backfills. Images have been reduced in size. [file peerj-03-1112-s003.zip › A1 N2 P1010069.jpg]

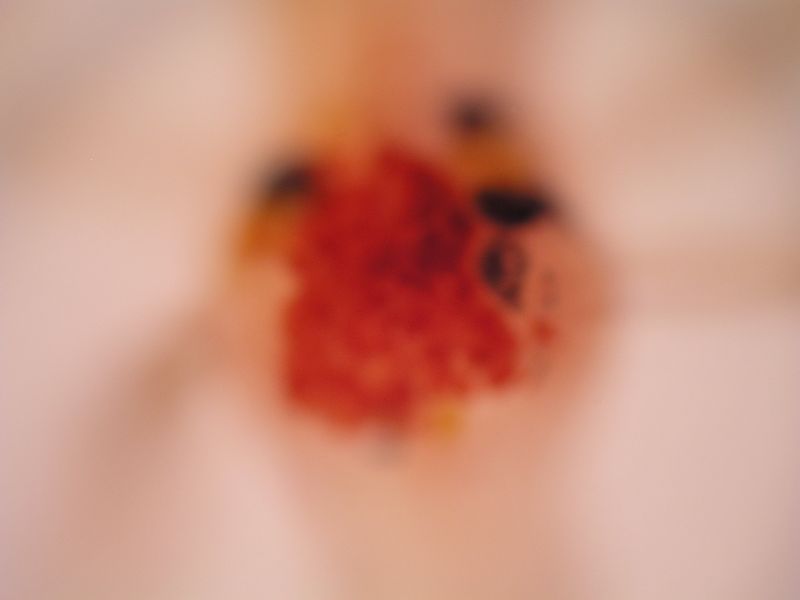

Supplement: Supplemental Information 2 — Micrographs of N3 backfills. Images have been reduced in size. [file peerj-03-1112-s003.zip › A1N3 2009 07 13 (1).jpg]

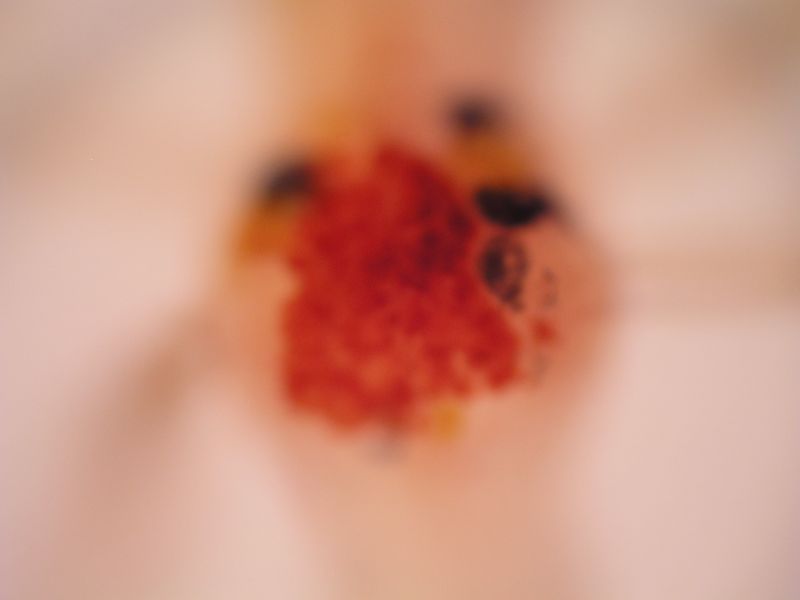

Supplement: Supplemental Information 2 — Micrographs of N3 backfills. Images have been reduced in size. [file peerj-03-1112-s003.zip › A1N3 2009 07 13 (2).jpg]

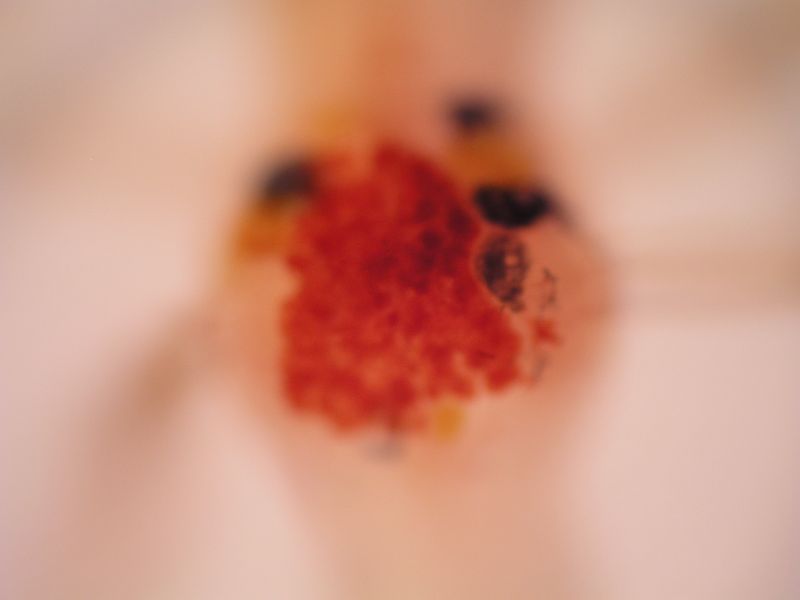

Supplement: Supplemental Information 2 — Micrographs of N3 backfills. Images have been reduced in size. [file peerj-03-1112-s003.zip › A1N3 2009 07 13 (3).jpg]

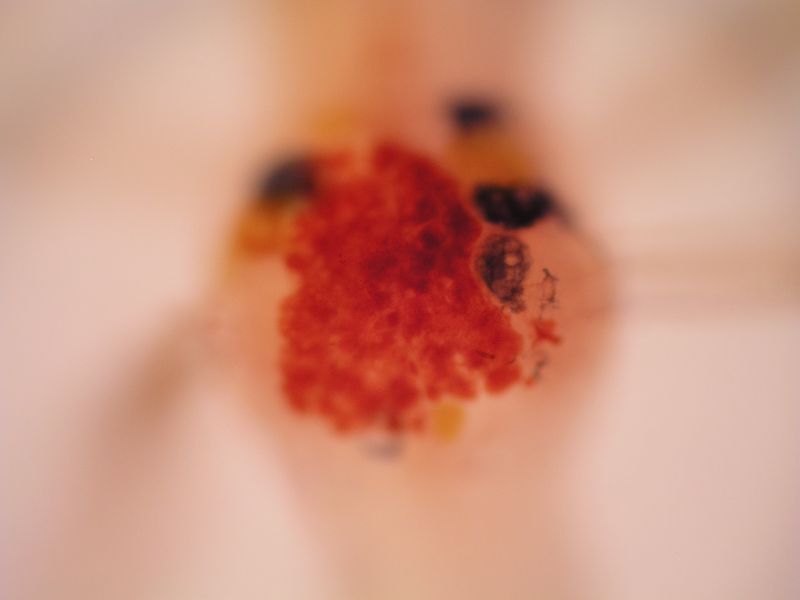

Supplement: Supplemental Information 2 — Micrographs of N3 backfills. Images have been reduced in size. [file peerj-03-1112-s003.zip › A1N3 2009 07 13 (4).jpg]

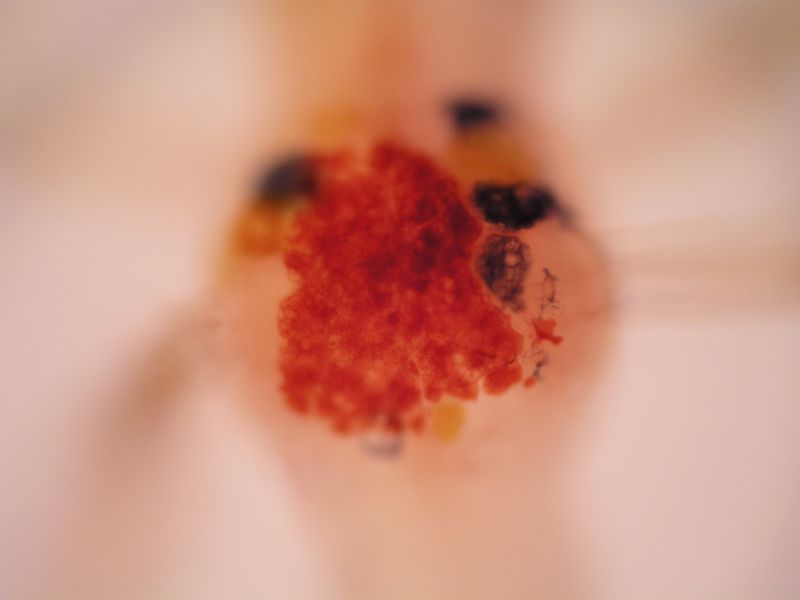

Supplement: Supplemental Information 2 — Micrographs of N3 backfills. Images have been reduced in size. [file peerj-03-1112-s003.zip › A1N3 2009 07 13 (5).jpg]

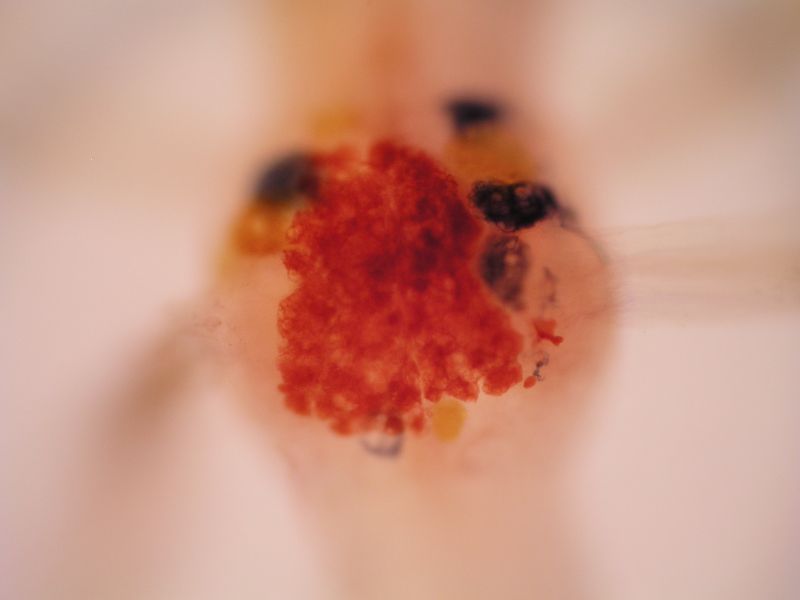

Supplement: Supplemental Information 2 — Micrographs of N3 backfills. Images have been reduced in size. [file peerj-03-1112-s003.zip › A1N3 2009 07 13 (6).jpg]

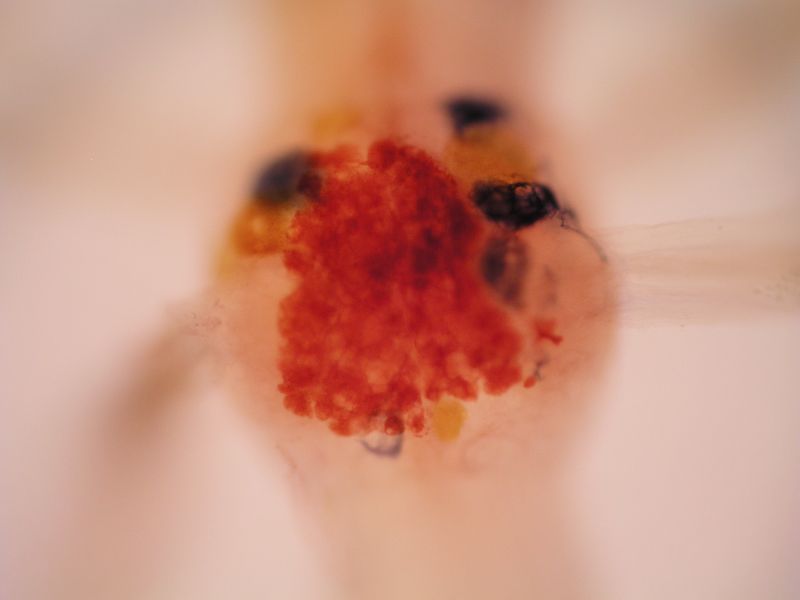

Supplement: Supplemental Information 2 — Micrographs of N3 backfills. Images have been reduced in size. [file peerj-03-1112-s003.zip › A1N3 2009 07 13 (7).jpg]

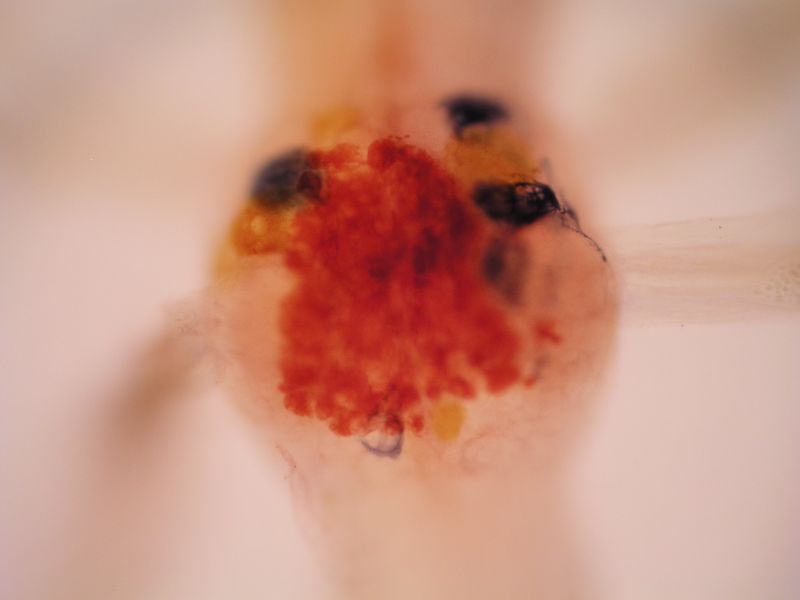

Supplement: Supplemental Information 2 — Micrographs of N3 backfills. Images have been reduced in size. [file peerj-03-1112-s003.zip › A1N3 2009 07 13 (8).jpg]

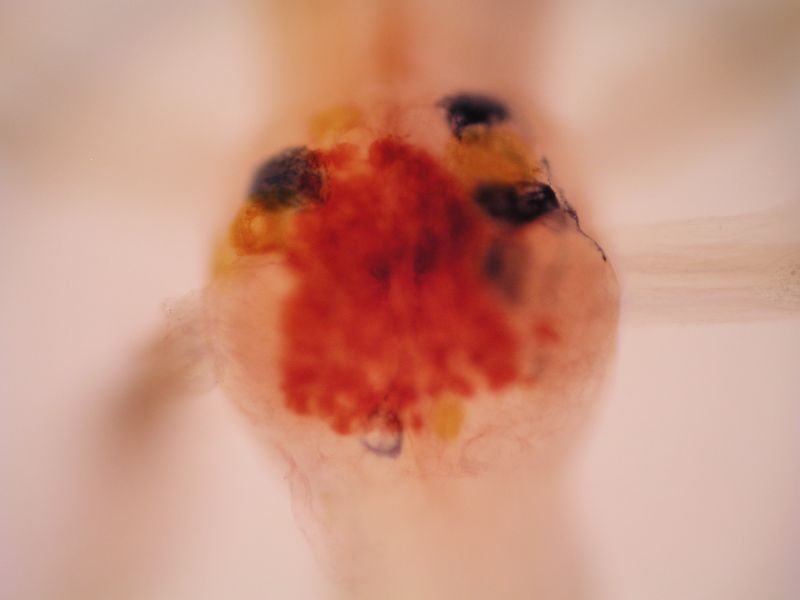

Supplement: Supplemental Information 2 — Micrographs of N3 backfills. Images have been reduced in size. [file peerj-03-1112-s003.zip › A1N3 2009 07 13 (9).jpg]

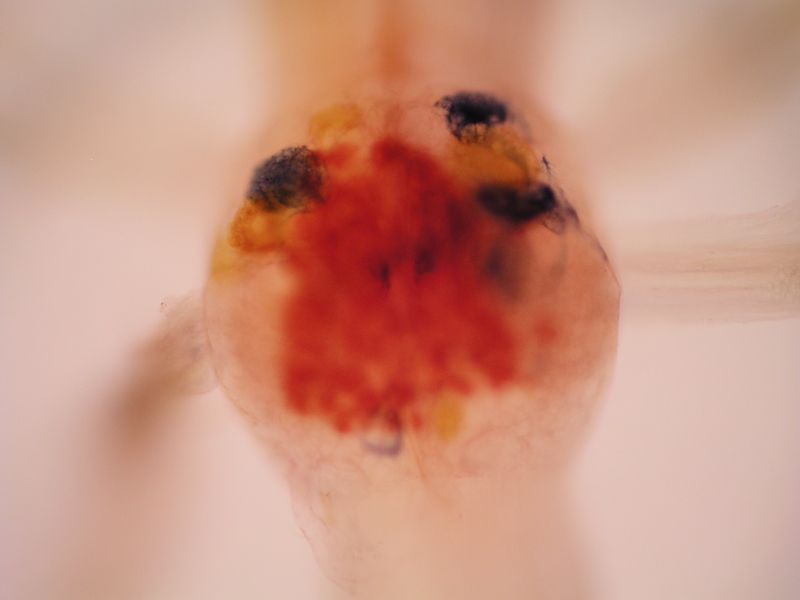

Supplement: Supplemental Information 2 — Micrographs of N3 backfills. Images have been reduced in size. [file peerj-03-1112-s003.zip › A1N3 2009 07 13 (10).jpg]

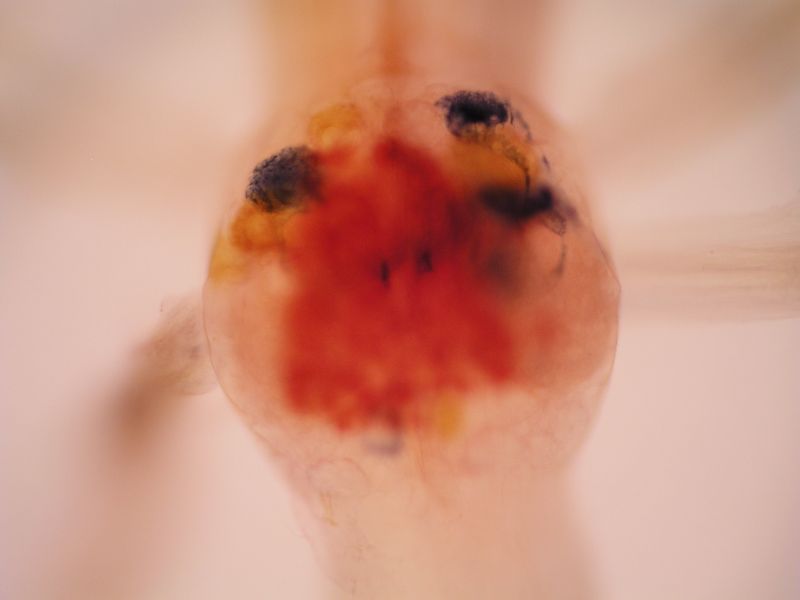

Supplement: Supplemental Information 2 — Micrographs of N3 backfills. Images have been reduced in size. [file peerj-03-1112-s003.zip › A1N3 2009 07 13 (11).jpg]

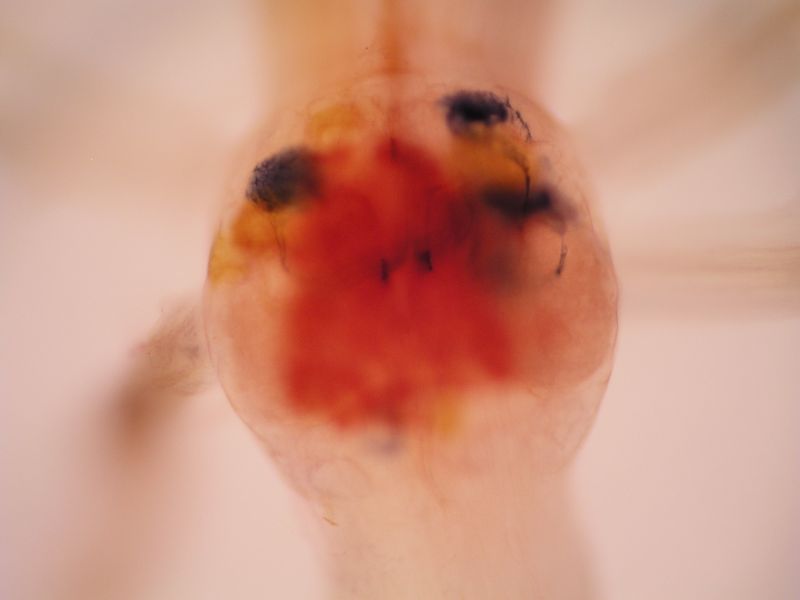

Supplement: Supplemental Information 2 — Micrographs of N3 backfills. Images have been reduced in size. [file peerj-03-1112-s003.zip › A1N3 2009 07 13 (12).jpg]

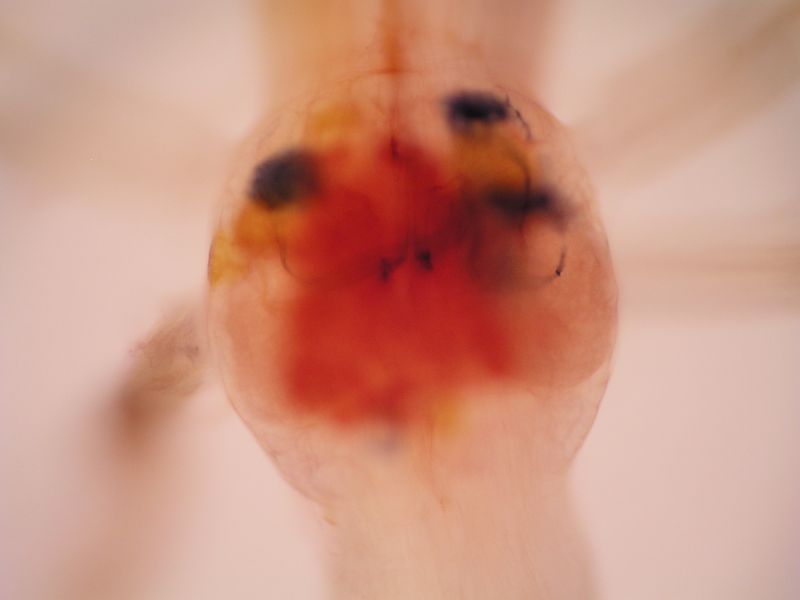

Supplement: Supplemental Information 2 — Micrographs of N3 backfills. Images have been reduced in size. [file peerj-03-1112-s003.zip › A1N3 2009 07 13 (13).jpg]

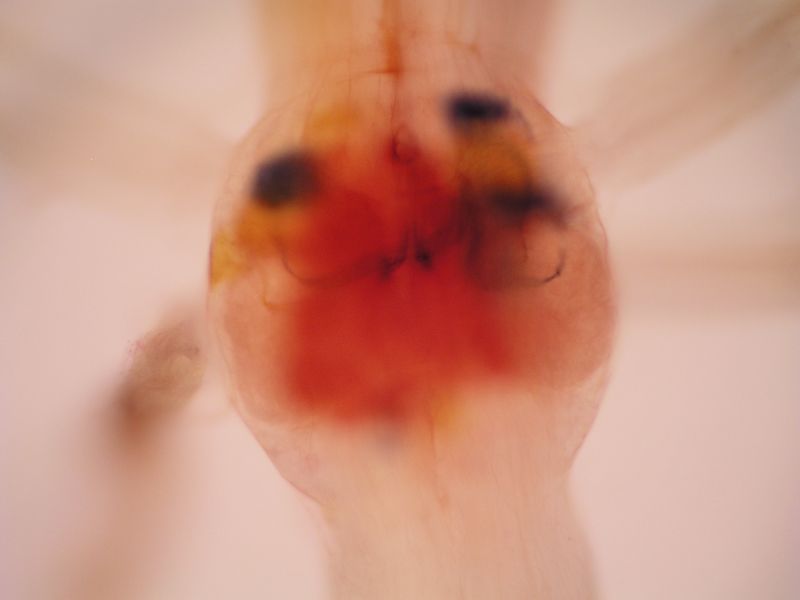

Supplement: Supplemental Information 2 — Micrographs of N3 backfills. Images have been reduced in size. [file peerj-03-1112-s003.zip › A1N3 2009 07 13 (14).jpg]

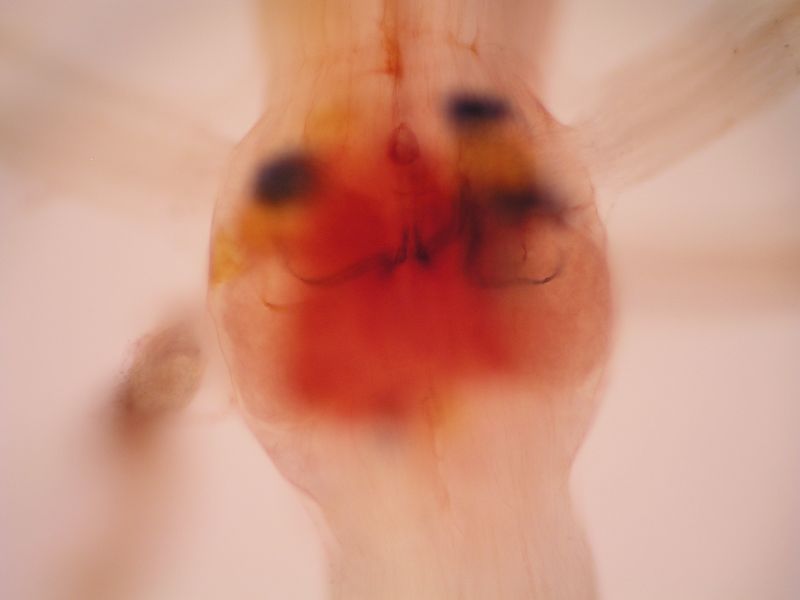

Supplement: Supplemental Information 2 — Micrographs of N3 backfills. Images have been reduced in size. [file peerj-03-1112-s003.zip › A1N3 2009 07 13 (15).jpg]

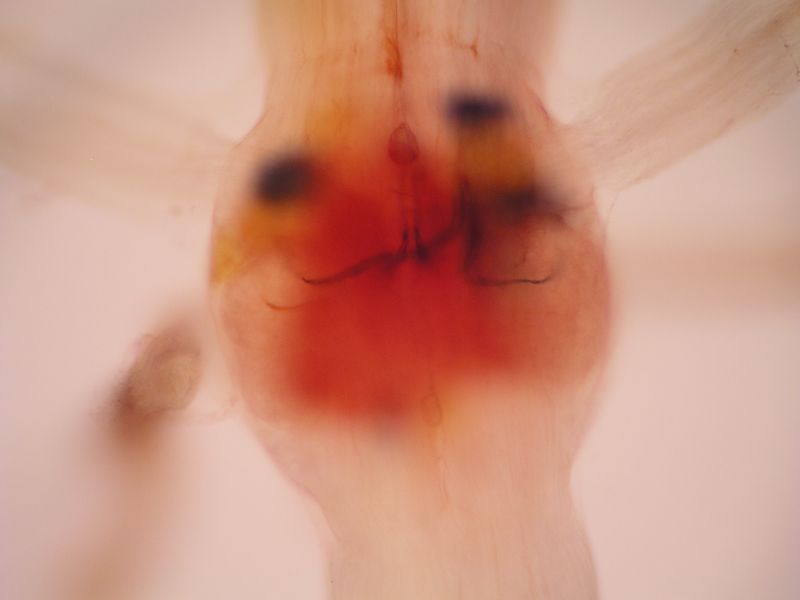

Supplement: Supplemental Information 2 — Micrographs of N3 backfills. Images have been reduced in size. [file peerj-03-1112-s003.zip › A1N3 2009 07 13 (16).jpg]

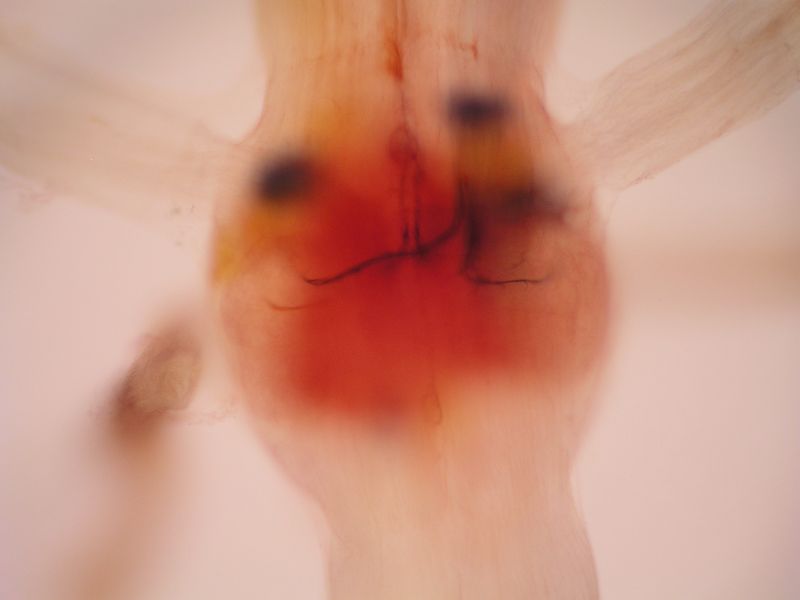

Supplement: Supplemental Information 2 — Micrographs of N3 backfills. Images have been reduced in size. [file peerj-03-1112-s003.zip › A1N3 2009 07 13.jpg]

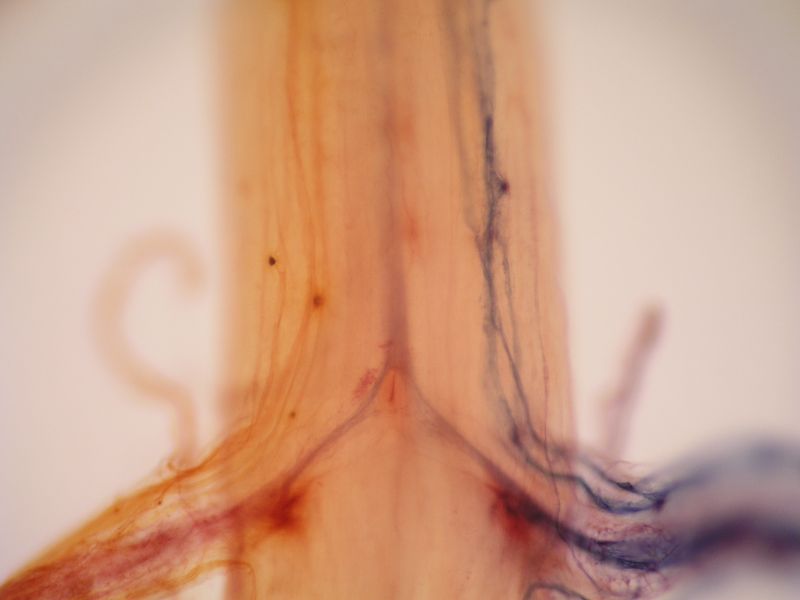

Supplement: Supplemental Information 2 — Micrographs of N3 backfills. Images have been reduced in size. [file peerj-03-1112-s003.zip › A1N3 axons 2009 07 15 (1).jpg]

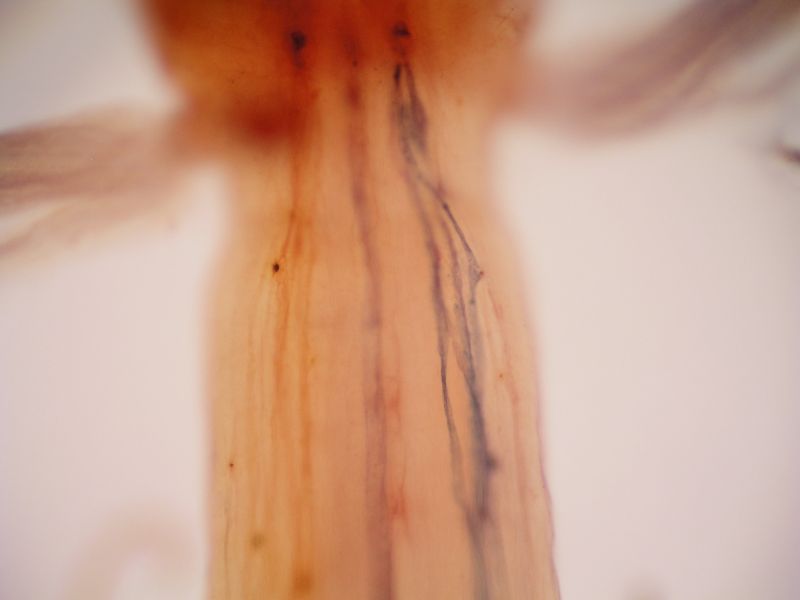

Supplement: Supplemental Information 2 — Micrographs of N3 backfills. Images have been reduced in size. [file peerj-03-1112-s003.zip › A1N3 axons 2009 07 15 (2).jpg]

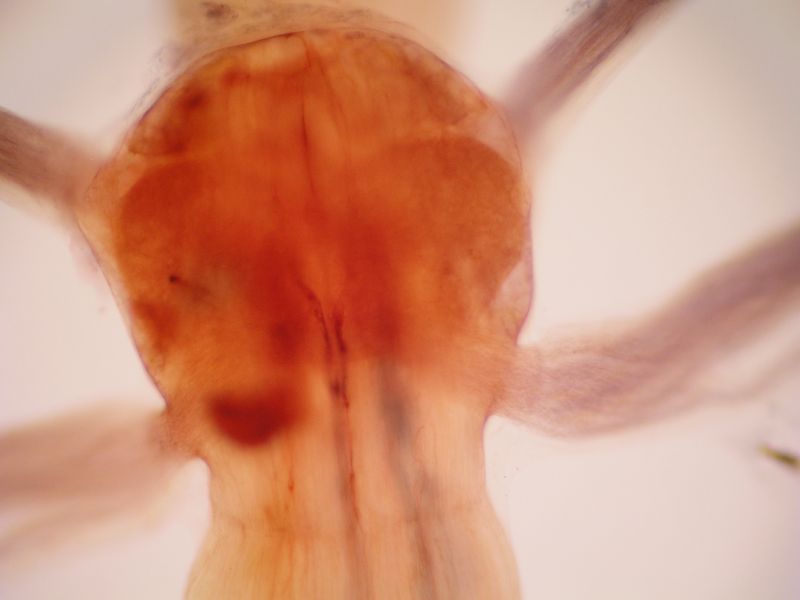

Supplement: Supplemental Information 2 — Micrographs of N3 backfills. Images have been reduced in size. [file peerj-03-1112-s003.zip › A1N3 axons 2009 07 15.jpg]

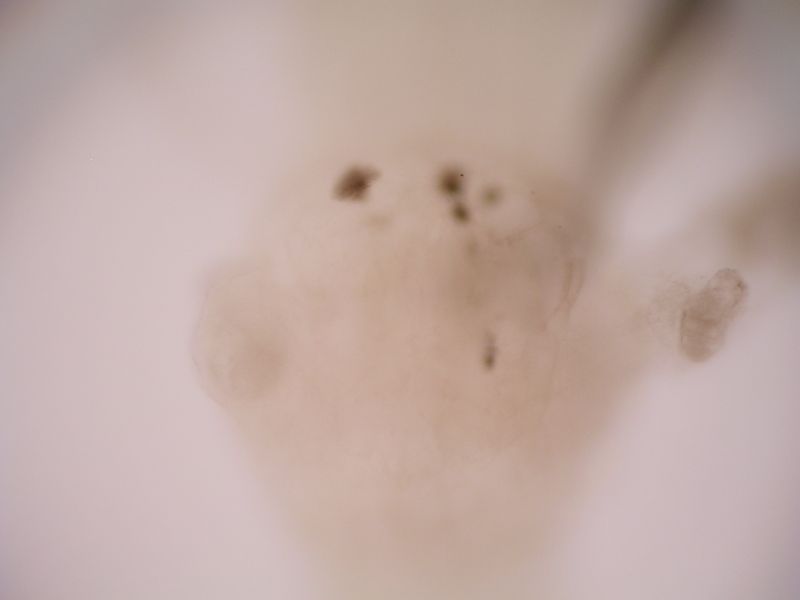

Supplement: Supplemental Information 2 — Micrographs of N3 backfills. Images have been reduced in size. [file peerj-03-1112-s003.zip › A1N3 FAC 2009 07 10 (1).jpg]

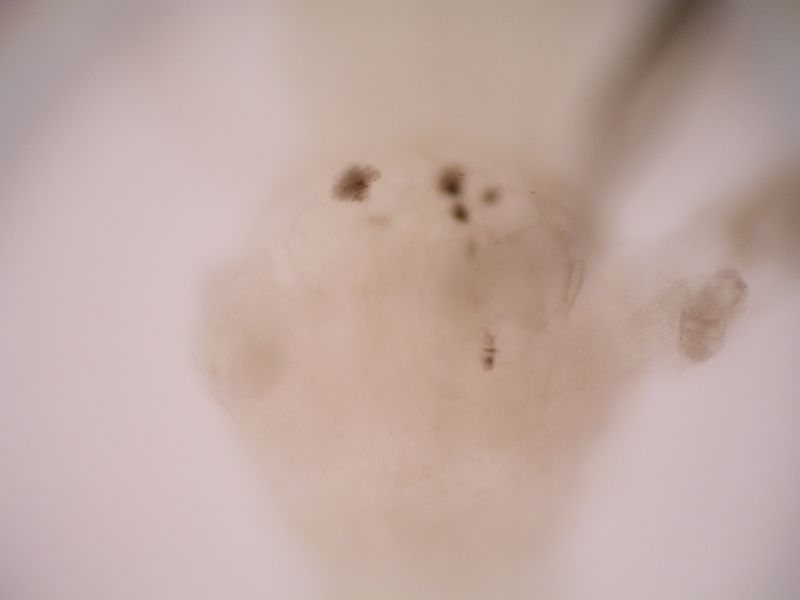

Supplement: Supplemental Information 2 — Micrographs of N3 backfills. Images have been reduced in size. [file peerj-03-1112-s003.zip › A1N3 FAC 2009 07 10 (2).jpg]

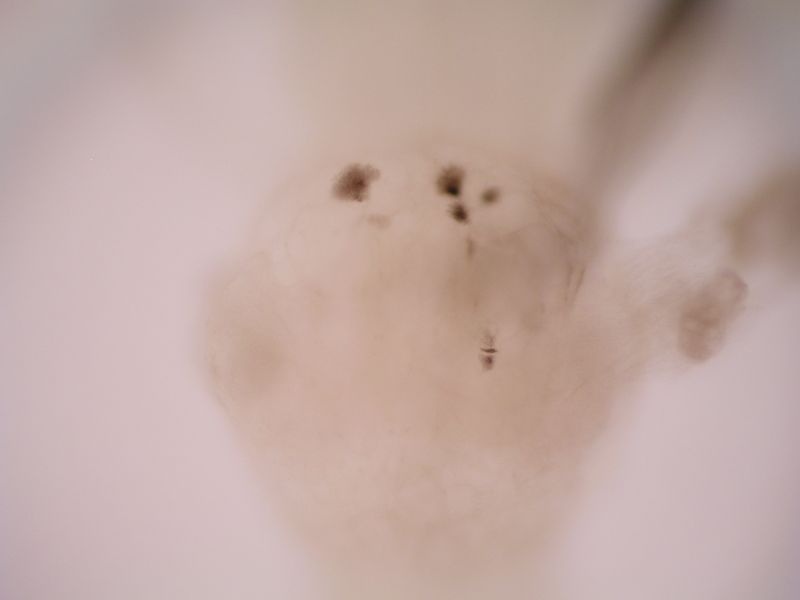

Supplement: Supplemental Information 2 — Micrographs of N3 backfills. Images have been reduced in size. [file peerj-03-1112-s003.zip › A1N3 FAC 2009 07 10 (3).jpg]

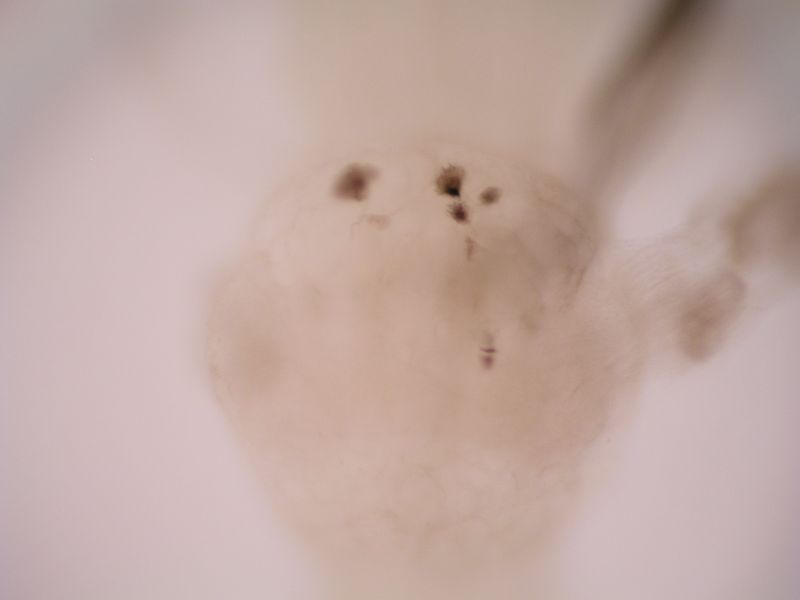

Supplement: Supplemental Information 2 — Micrographs of N3 backfills. Images have been reduced in size. [file peerj-03-1112-s003.zip › A1N3 FAC 2009 07 10 (4).jpg]

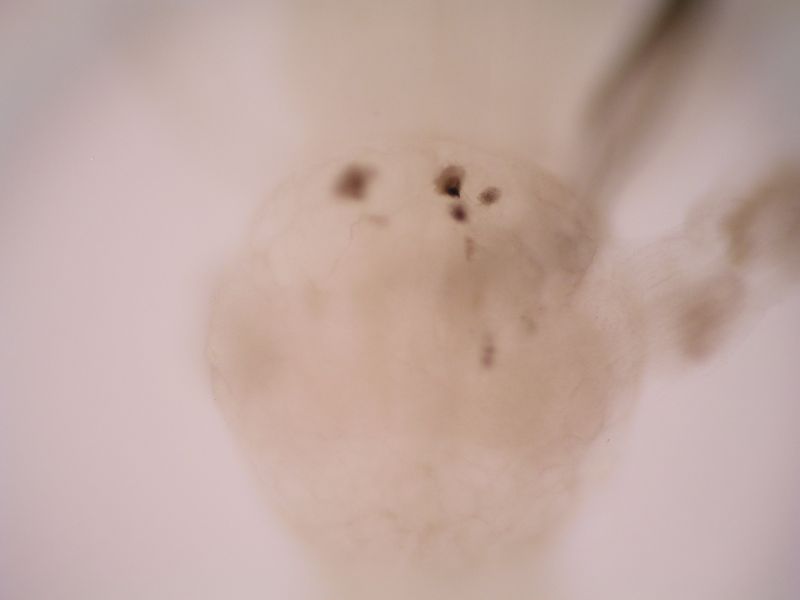

Supplement: Supplemental Information 2 — Micrographs of N3 backfills. Images have been reduced in size. [file peerj-03-1112-s003.zip › A1N3 FAC 2009 07 10 (5).jpg]

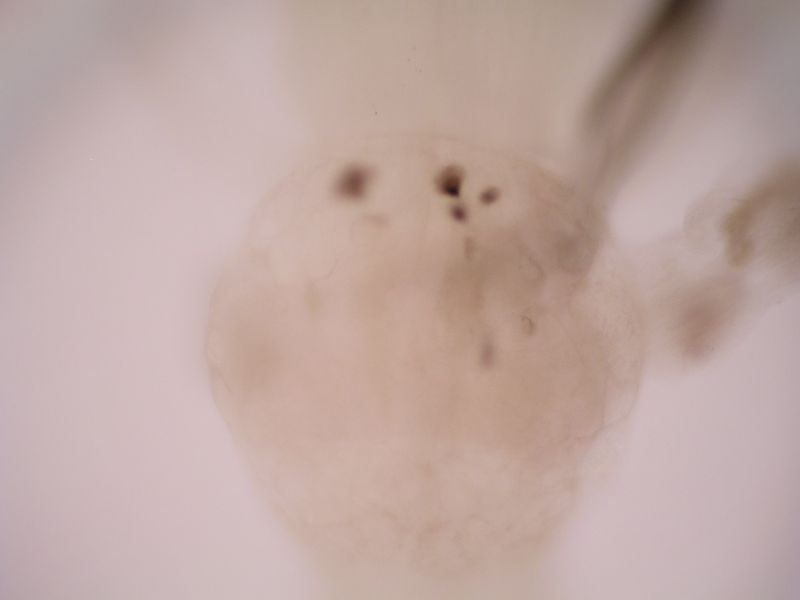

Supplement: Supplemental Information 2 — Micrographs of N3 backfills. Images have been reduced in size. [file peerj-03-1112-s003.zip › A1N3 FAC 2009 07 10 (6).jpg]

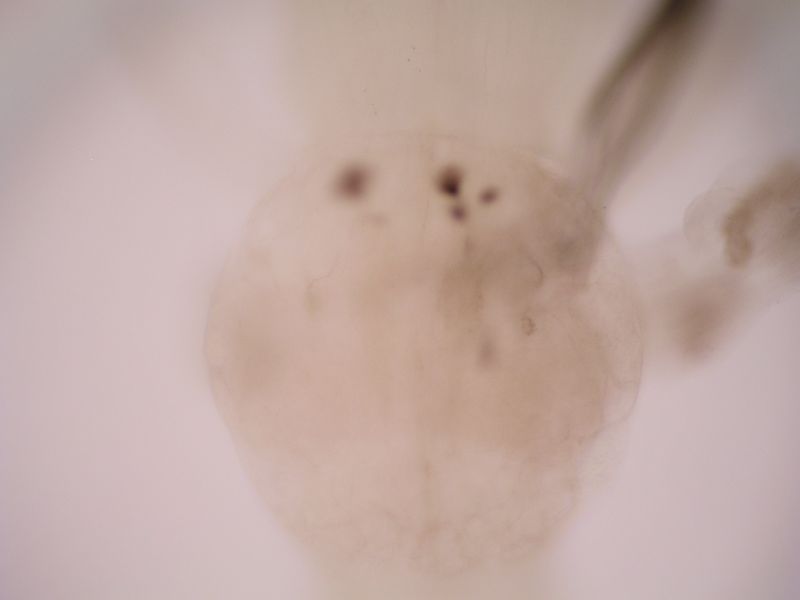

Supplement: Supplemental Information 2 — Micrographs of N3 backfills. Images have been reduced in size. [file peerj-03-1112-s003.zip › A1N3 FAC 2009 07 10 (7).jpg]

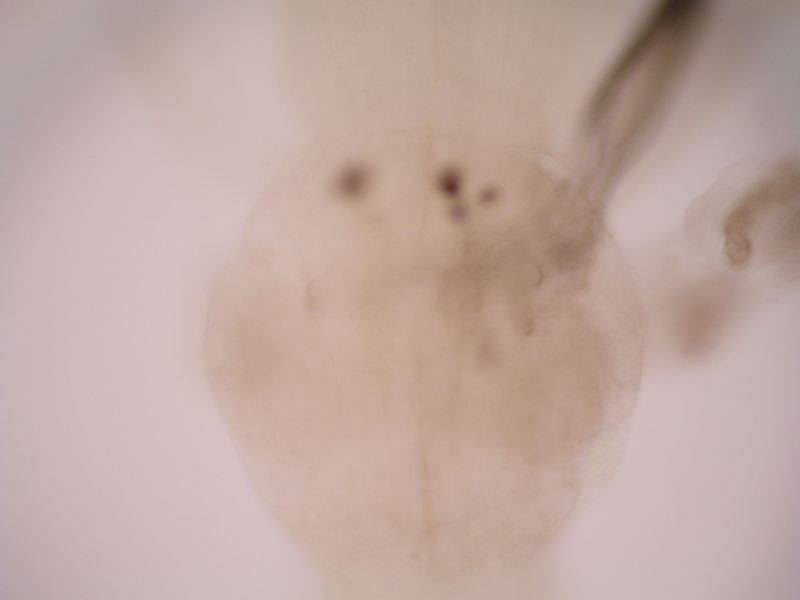

Supplement: Supplemental Information 2 — Micrographs of N3 backfills. Images have been reduced in size. [file peerj-03-1112-s003.zip › A1N3 FAC 2009 07 10 (8).jpg]

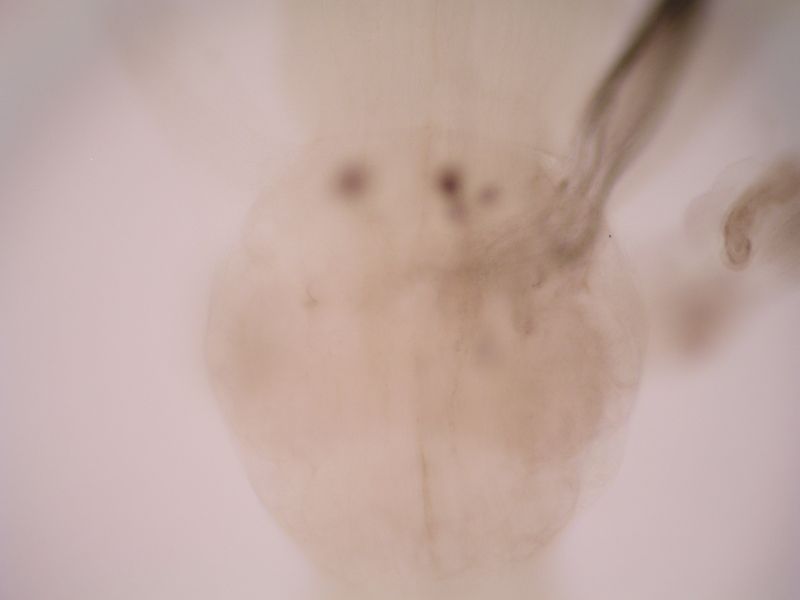

Supplement: Supplemental Information 2 — Micrographs of N3 backfills. Images have been reduced in size. [file peerj-03-1112-s003.zip › A1N3 FAC 2009 07 10 (9).jpg]

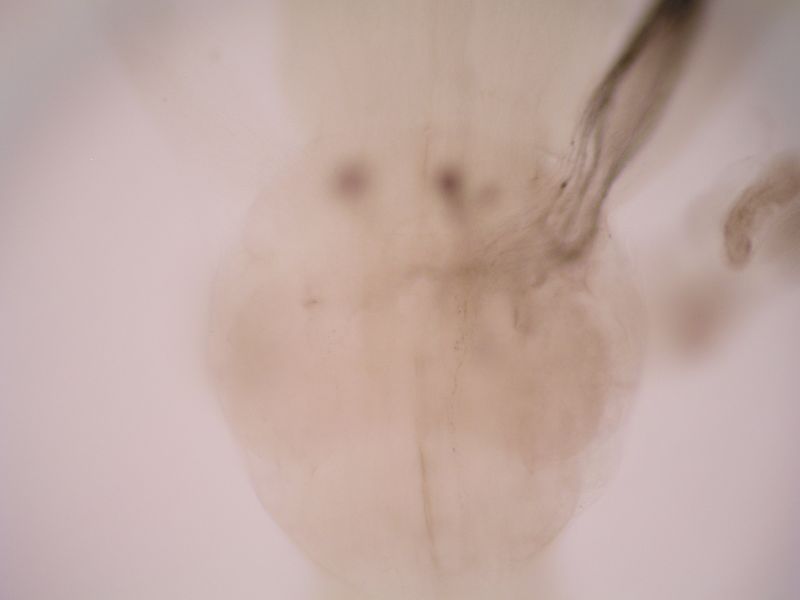

Supplement: Supplemental Information 2 — Micrographs of N3 backfills. Images have been reduced in size. [file peerj-03-1112-s003.zip › A1N3 FAC 2009 07 10 (10).jpg]

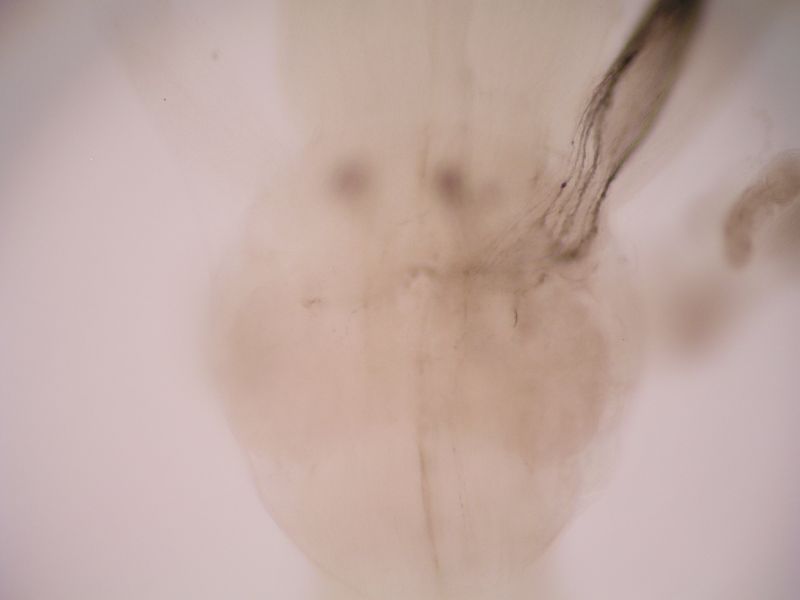

Supplement: Supplemental Information 2 — Micrographs of N3 backfills. Images have been reduced in size. [file peerj-03-1112-s003.zip › A1N3 FAC 2009 07 10 (11).jpg]

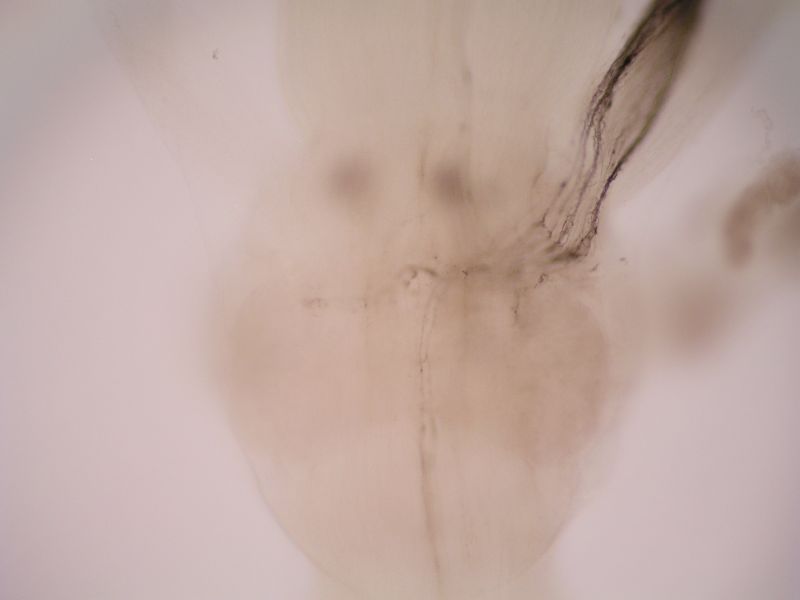

Supplement: Supplemental Information 2 — Micrographs of N3 backfills. Images have been reduced in size. [file peerj-03-1112-s003.zip › A1N3 FAC 2009 07 10 (12).jpg]

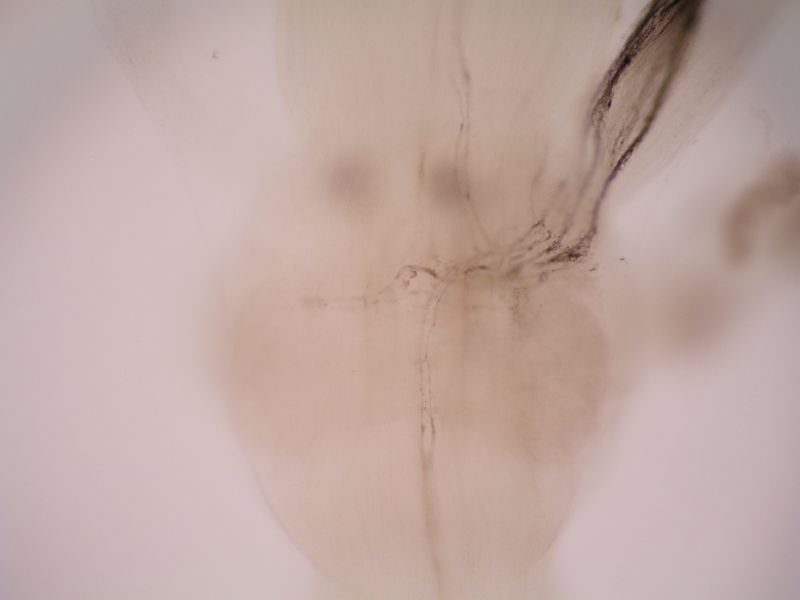

Supplement: Supplemental Information 2 — Micrographs of N3 backfills. Images have been reduced in size. [file peerj-03-1112-s003.zip › A1N3 FAC 2009 07 10 (13).jpg]

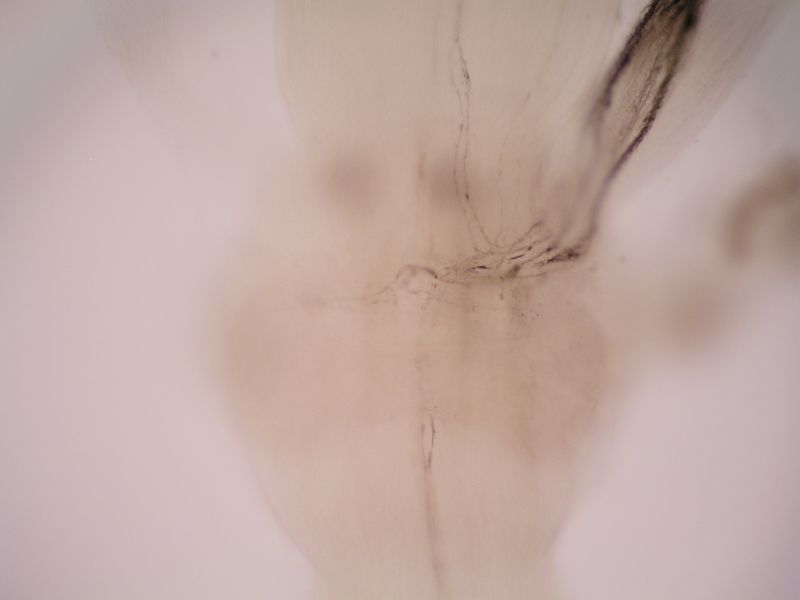

Supplement: Supplemental Information 2 — Micrographs of N3 backfills. Images have been reduced in size. [file peerj-03-1112-s003.zip › A1N3 FAC 2009 07 10 (14).jpg]

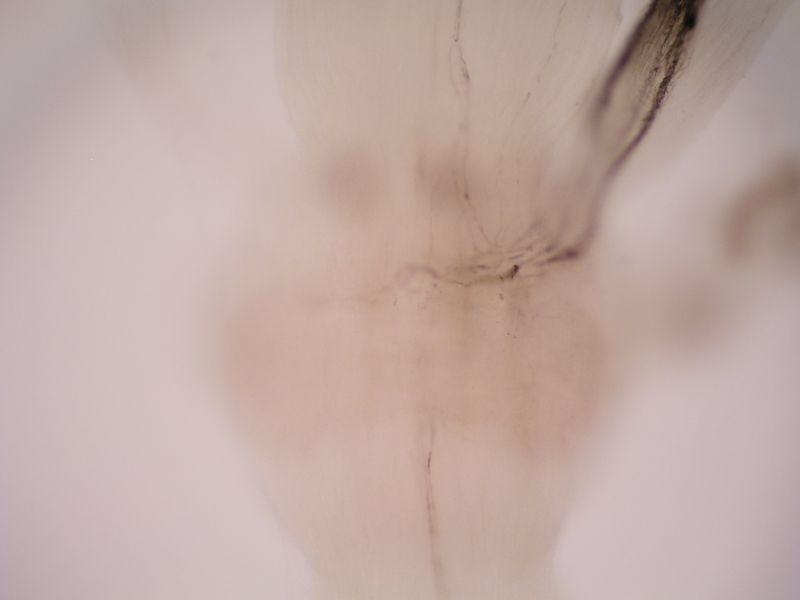

Supplement: Supplemental Information 2 — Micrographs of N3 backfills. Images have been reduced in size. [file peerj-03-1112-s003.zip › A1N3 FAC 2009 07 10 (15).jpg]

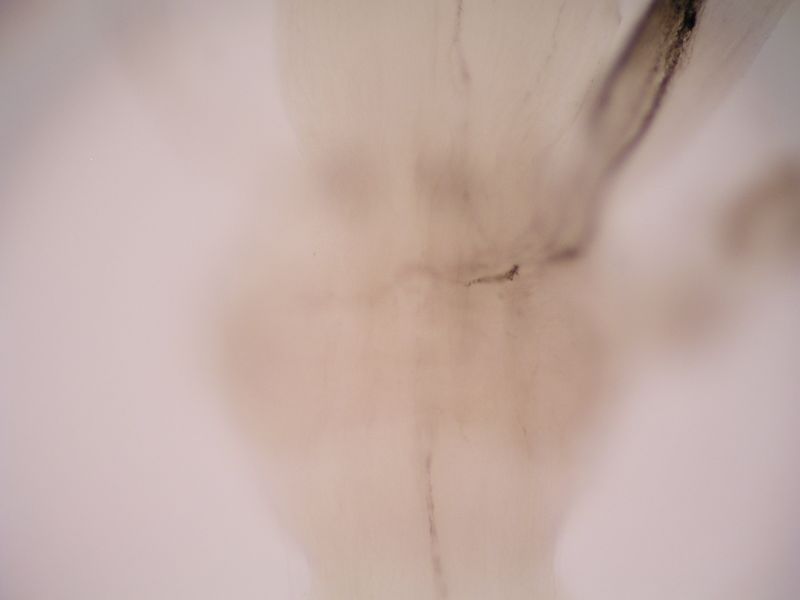

Supplement: Supplemental Information 2 — Micrographs of N3 backfills. Images have been reduced in size. [file peerj-03-1112-s003.zip › A1N3 FAC 2009 07 10 (16).jpg]

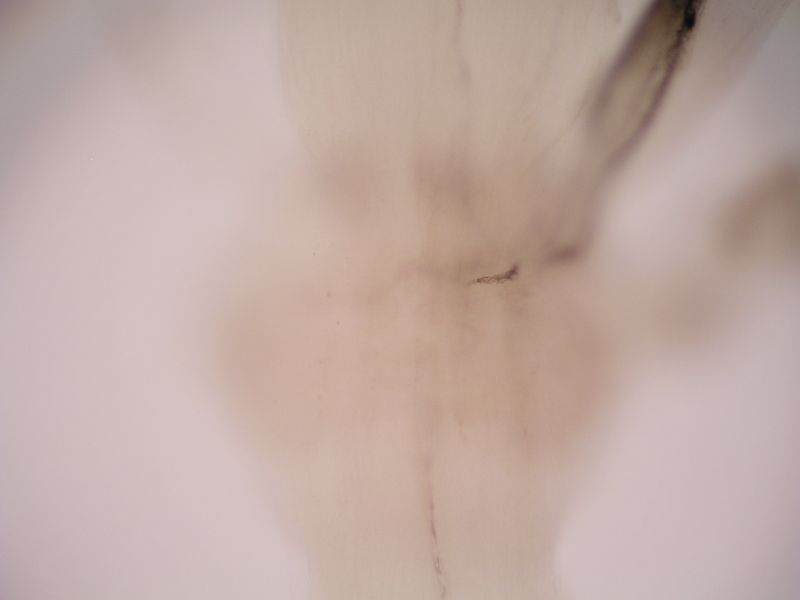

Supplement: Supplemental Information 2 — Micrographs of N3 backfills. Images have been reduced in size. [file peerj-03-1112-s003.zip › A1N3 FAC 2009 07 10 (17).jpg]

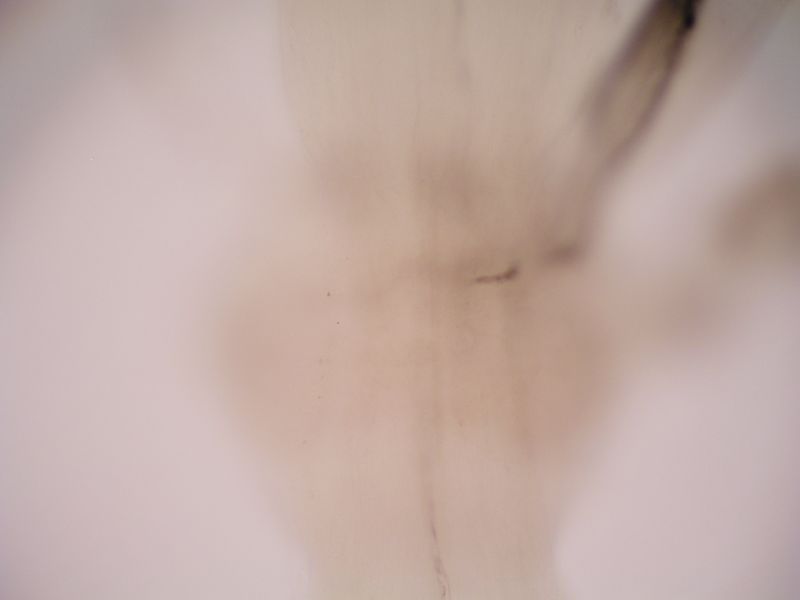

Supplement: Supplemental Information 2 — Micrographs of N3 backfills. Images have been reduced in size. [file peerj-03-1112-s003.zip › A1N3 FAC 2009 07 10 (18).jpg]

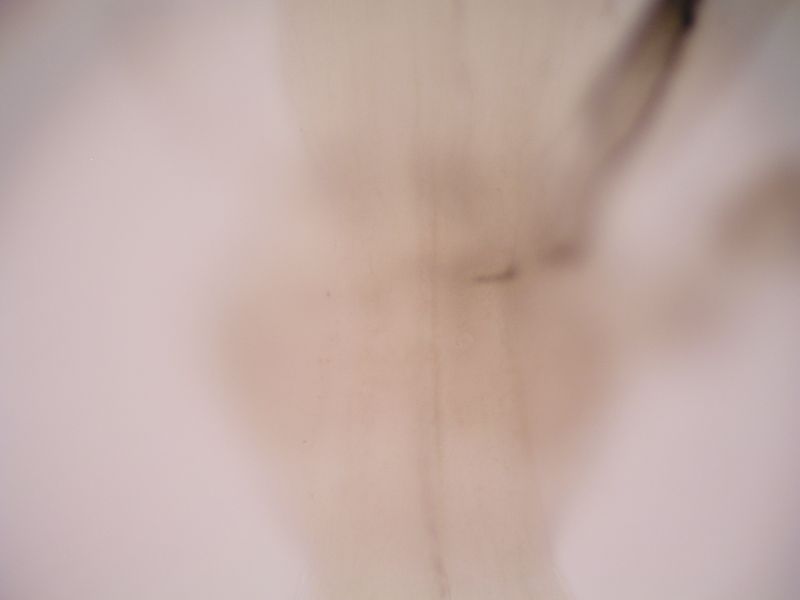

Supplement: Supplemental Information 2 — Micrographs of N3 backfills. Images have been reduced in size. [file peerj-03-1112-s003.zip › A1N3 FAC 2009 07 10 (19).jpg]

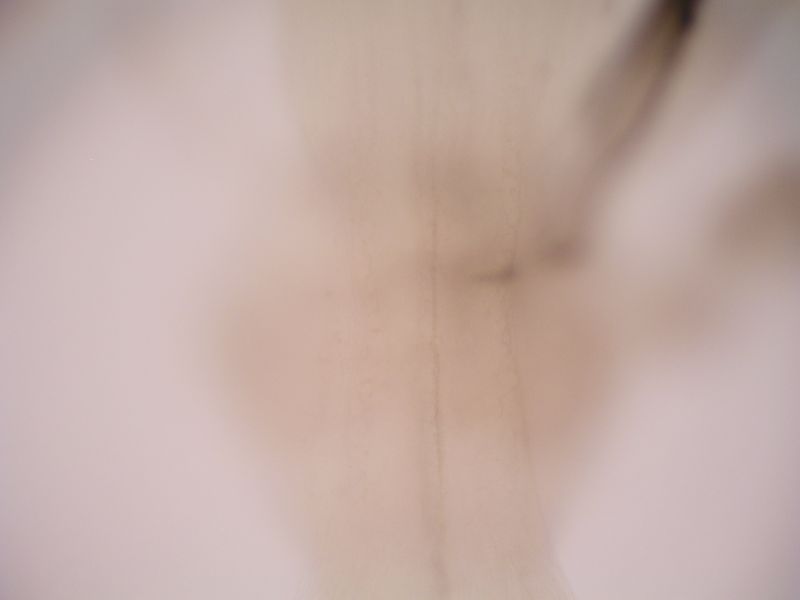

Supplement: Supplemental Information 2 — Micrographs of N3 backfills. Images have been reduced in size. [file peerj-03-1112-s003.zip › A1N3 FAC 2009 07 10.jpg]

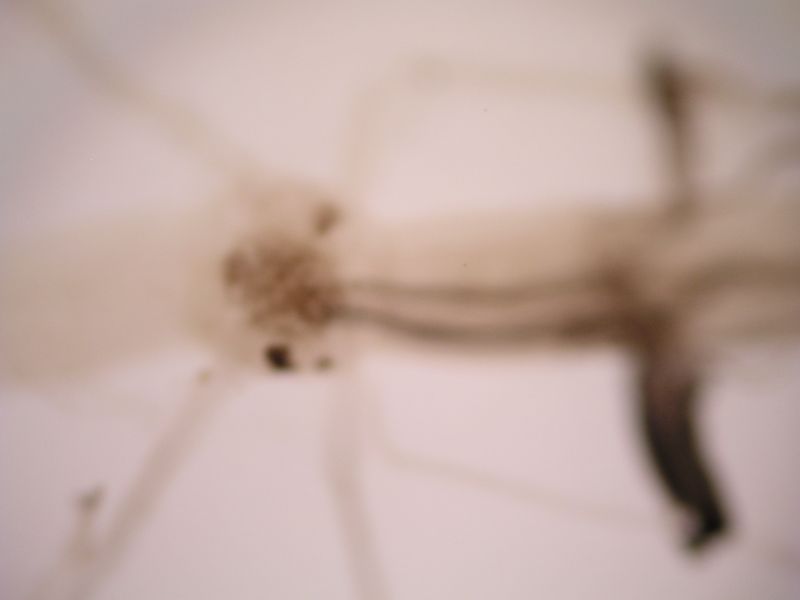

Supplement: Supplemental Information 2 — Micrographs of N3 backfills. Images have been reduced in size. [file peerj-03-1112-s003.zip › A1N3 low 2007 09 10 (1).jpg]

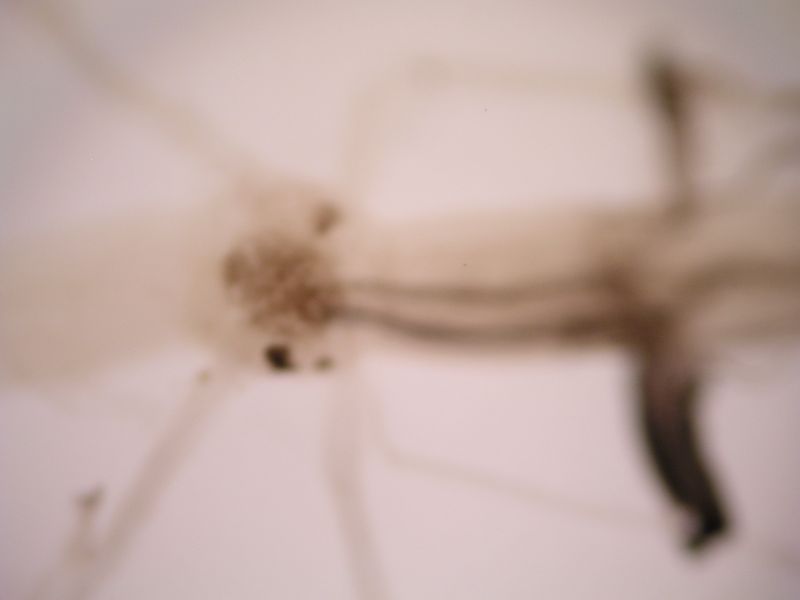

Supplement: Supplemental Information 2 — Micrographs of N3 backfills. Images have been reduced in size. [file peerj-03-1112-s003.zip › A1N3 low 2007 09 10 (2).jpg]

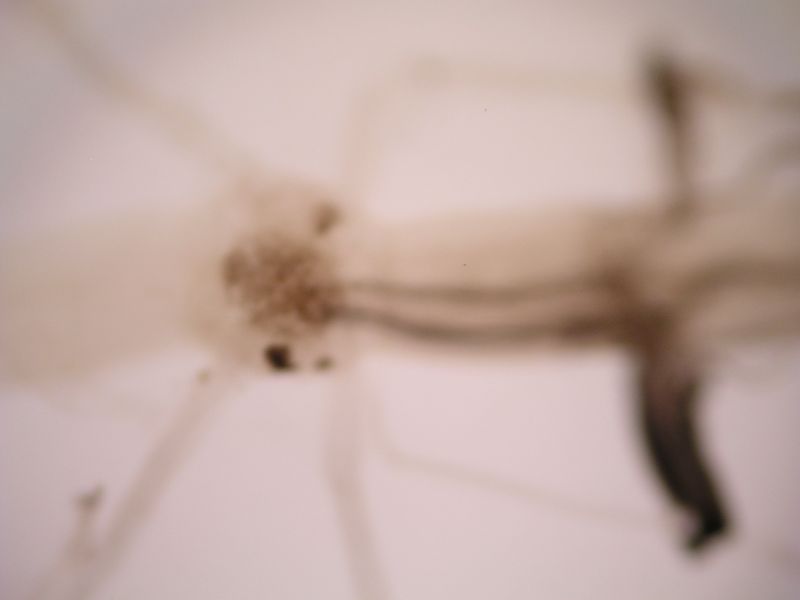

Supplement: Supplemental Information 2 — Micrographs of N3 backfills. Images have been reduced in size. [file peerj-03-1112-s003.zip › A1N3 low 2007 09 10 (3).jpg]

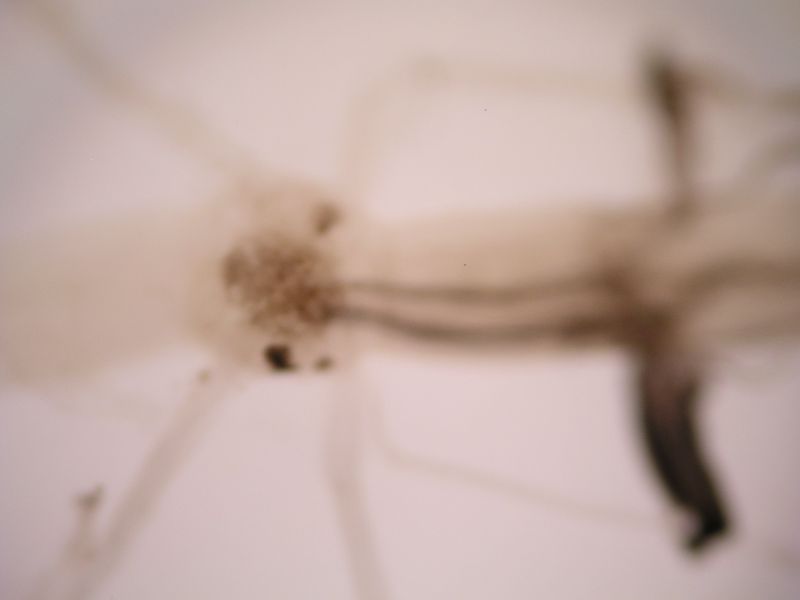

Supplement: Supplemental Information 2 — Micrographs of N3 backfills. Images have been reduced in size. [file peerj-03-1112-s003.zip › A1N3 low 2007 09 10 (4).jpg]

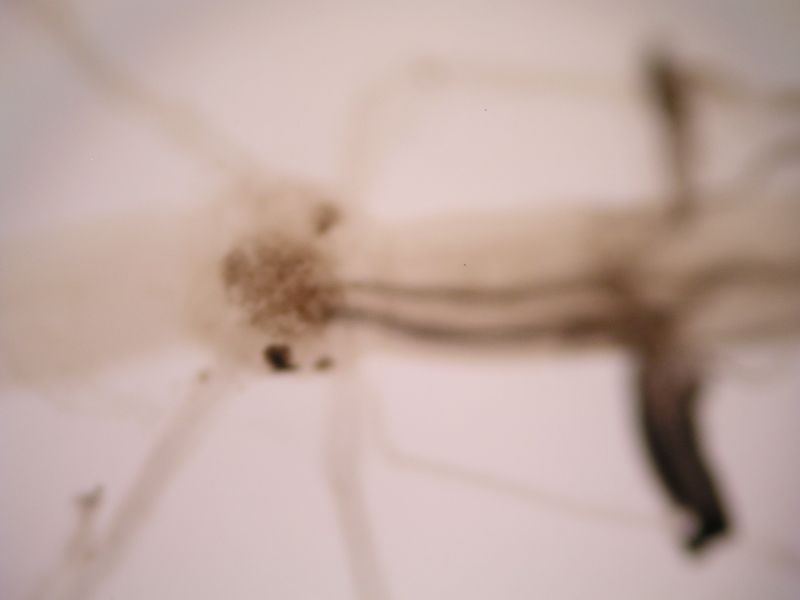

Supplement: Supplemental Information 2 — Micrographs of N3 backfills. Images have been reduced in size. [file peerj-03-1112-s003.zip › A1N3 low 2007 09 10 (5).jpg]

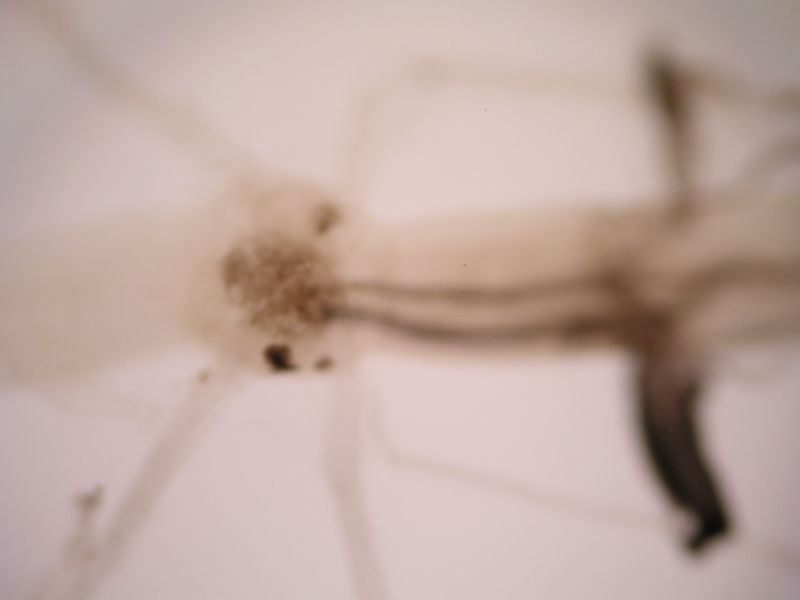

Supplement: Supplemental Information 2 — Micrographs of N3 backfills. Images have been reduced in size. [file peerj-03-1112-s003.zip › A1N3 low 2007 09 10 (6).jpg]

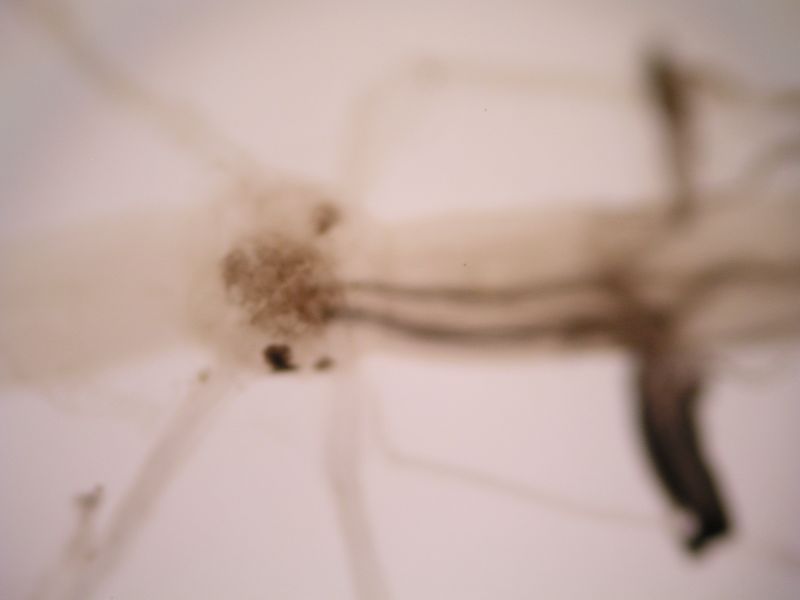

Supplement: Supplemental Information 2 — Micrographs of N3 backfills. Images have been reduced in size. [file peerj-03-1112-s003.zip › A1N3 low 2007 09 10 (7).jpg]

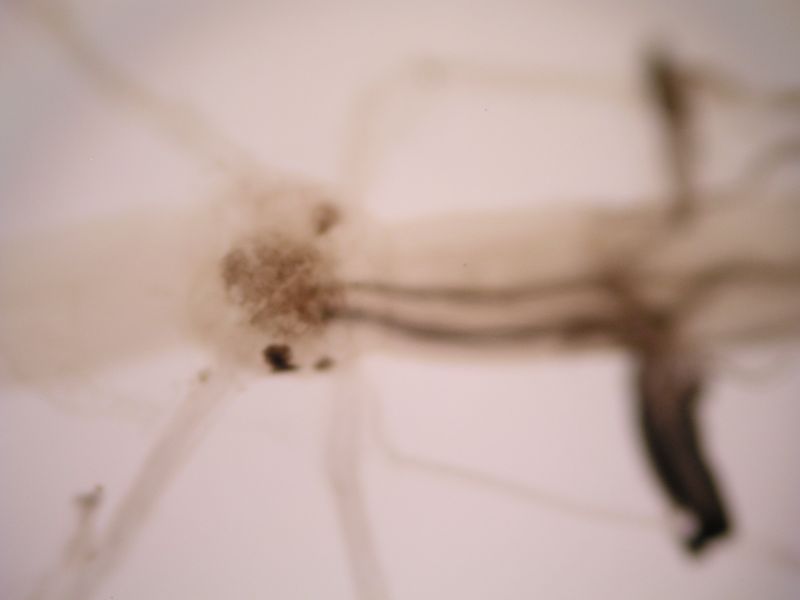

Supplement: Supplemental Information 2 — Micrographs of N3 backfills. Images have been reduced in size. [file peerj-03-1112-s003.zip › A1N3 low 2007 09 10 (8).jpg]

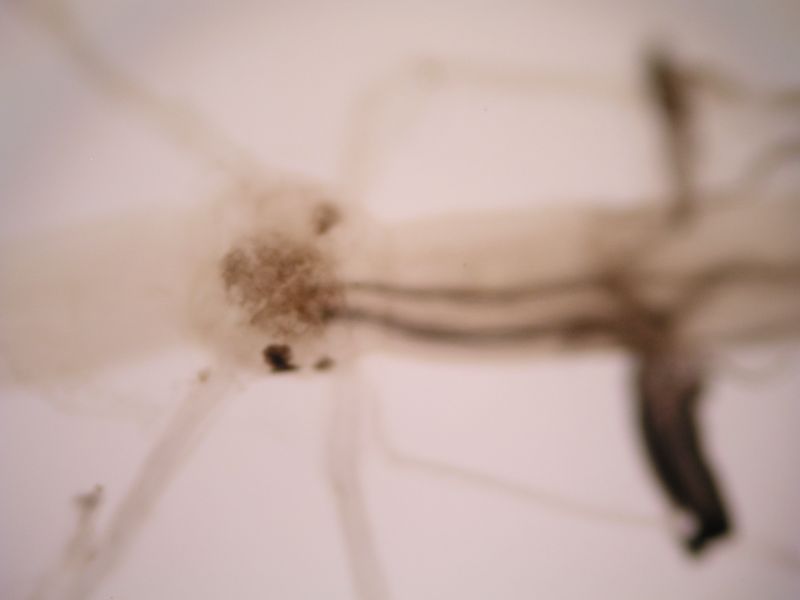

Supplement: Supplemental Information 2 — Micrographs of N3 backfills. Images have been reduced in size. [file peerj-03-1112-s003.zip › A1N3 low 2007 09 10 (9).jpg]

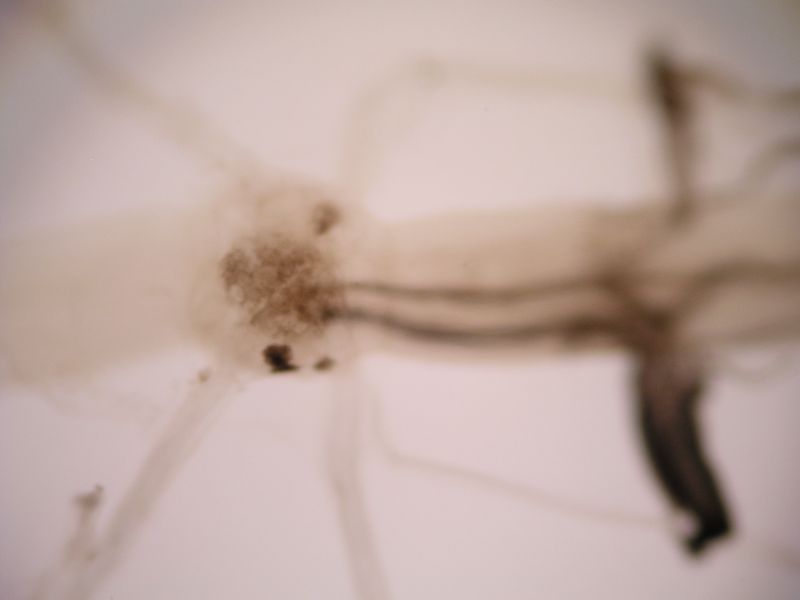

Supplement: Supplemental Information 2 — Micrographs of N3 backfills. Images have been reduced in size. [file peerj-03-1112-s003.zip › A1N3 low 2007 09 10 (10).jpg]

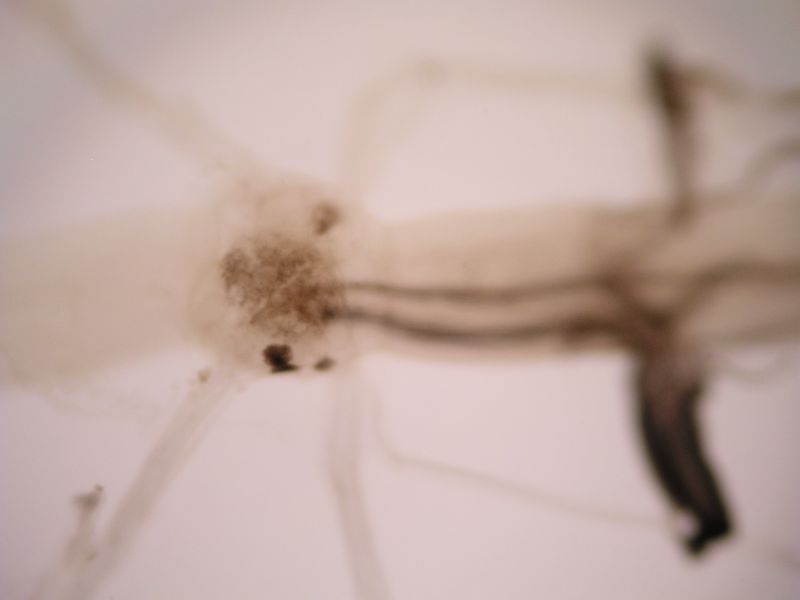

Supplement: Supplemental Information 2 — Micrographs of N3 backfills. Images have been reduced in size. [file peerj-03-1112-s003.zip › A1N3 low 2007 09 10 (11).jpg]

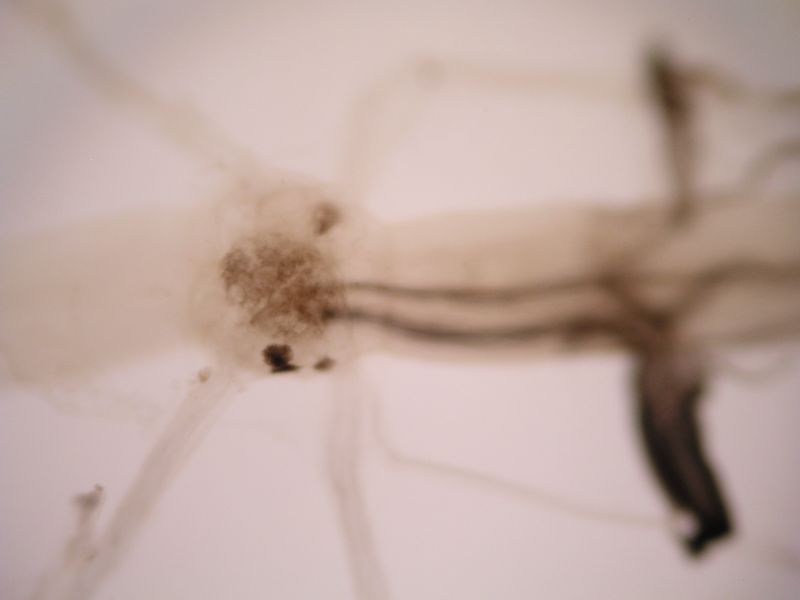

Supplement: Supplemental Information 2 — Micrographs of N3 backfills. Images have been reduced in size. [file peerj-03-1112-s003.zip › A1N3 low 2007 09 10 (12).jpg]

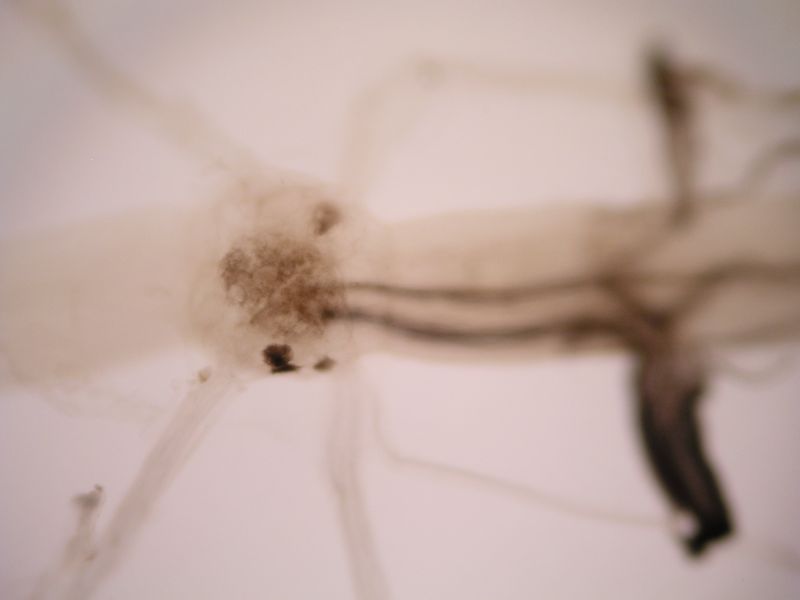

Supplement: Supplemental Information 2 — Micrographs of N3 backfills. Images have been reduced in size. [file peerj-03-1112-s003.zip › A1N3 low 2007 09 10 (13).jpg]

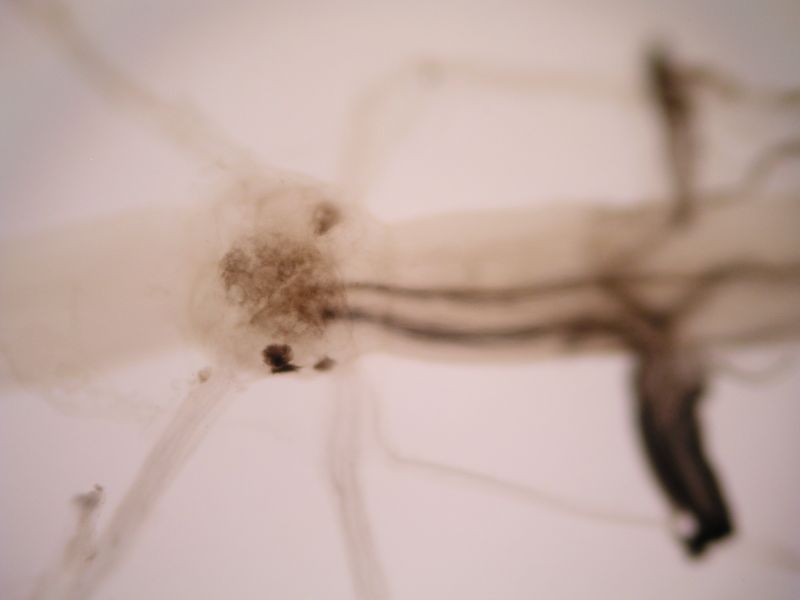

Supplement: Supplemental Information 2 — Micrographs of N3 backfills. Images have been reduced in size. [file peerj-03-1112-s003.zip › A1N3 low 2007 09 10 (14).jpg]

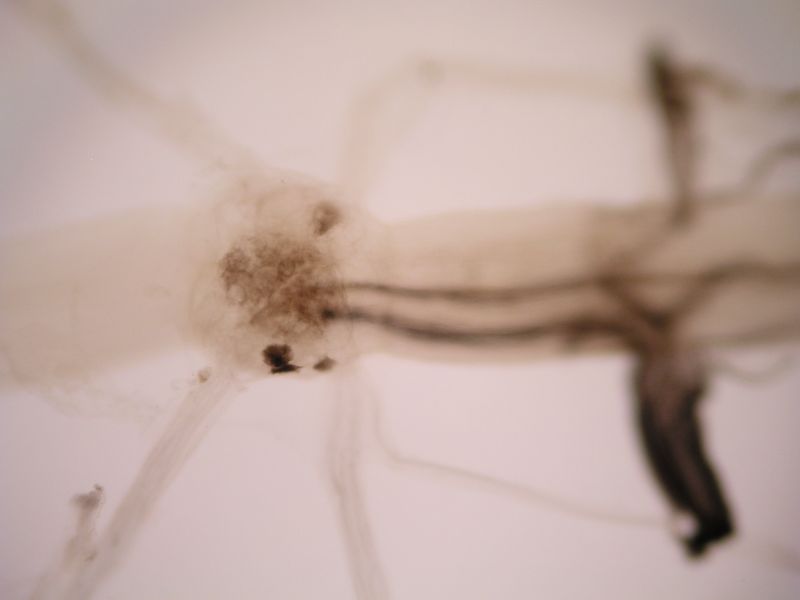

Supplement: Supplemental Information 2 — Micrographs of N3 backfills. Images have been reduced in size. [file peerj-03-1112-s003.zip › A1N3 low 2007 09 10 (15).jpg]

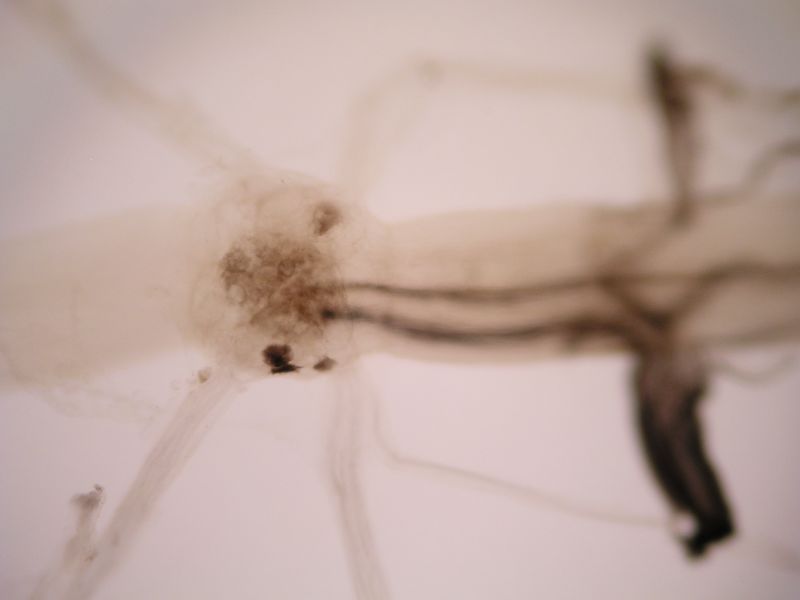

Supplement: Supplemental Information 2 — Micrographs of N3 backfills. Images have been reduced in size. [file peerj-03-1112-s003.zip › A1N3 low 2007 09 10 (16).jpg]

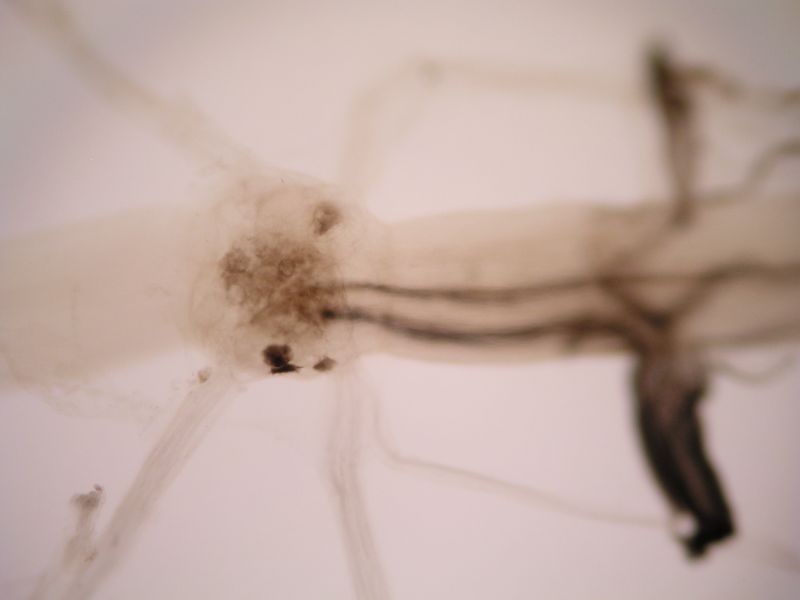

Supplement: Supplemental Information 2 — Micrographs of N3 backfills. Images have been reduced in size. [file peerj-03-1112-s003.zip › A1N3 low 2007 09 10 (17).jpg]

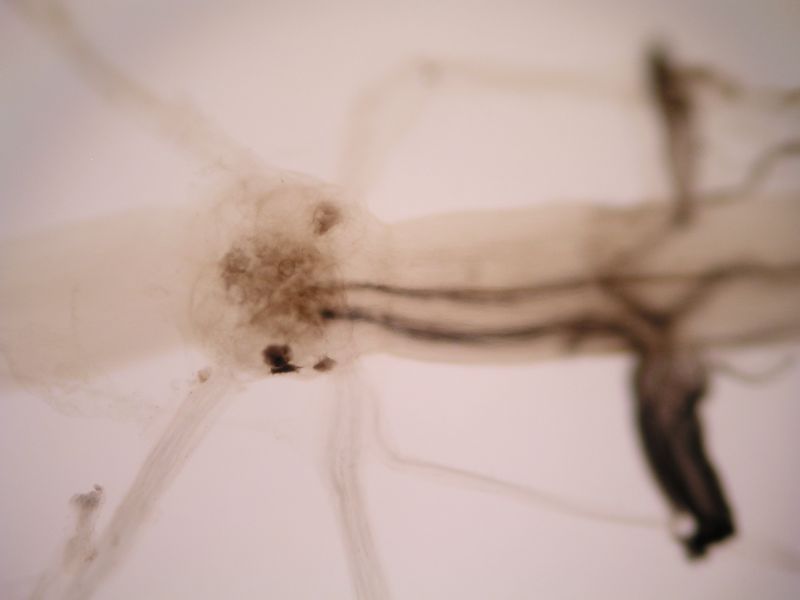

Supplement: Supplemental Information 2 — Micrographs of N3 backfills. Images have been reduced in size. [file peerj-03-1112-s003.zip › A1N3 low 2007 09 10 (18).jpg]

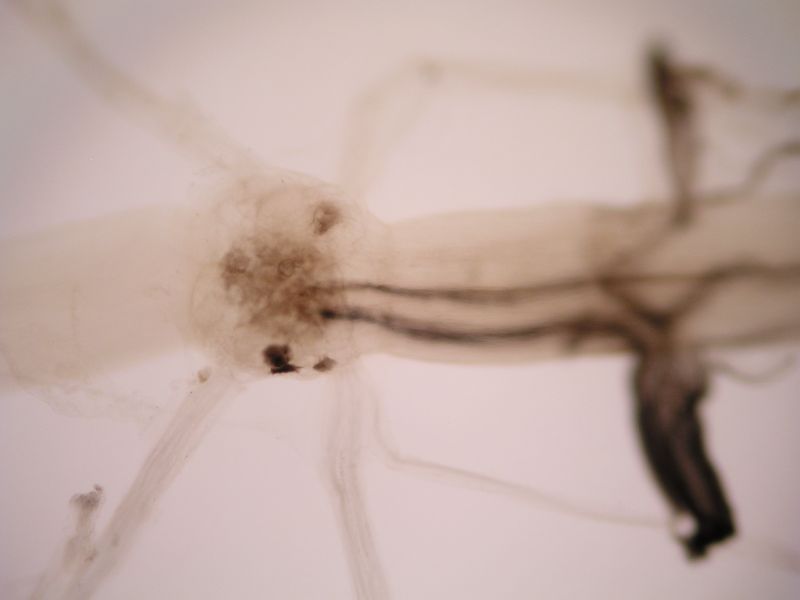

Supplement: Supplemental Information 2 — Micrographs of N3 backfills. Images have been reduced in size. [file peerj-03-1112-s003.zip › A1N3 low 2007 09 10 (19).jpg]

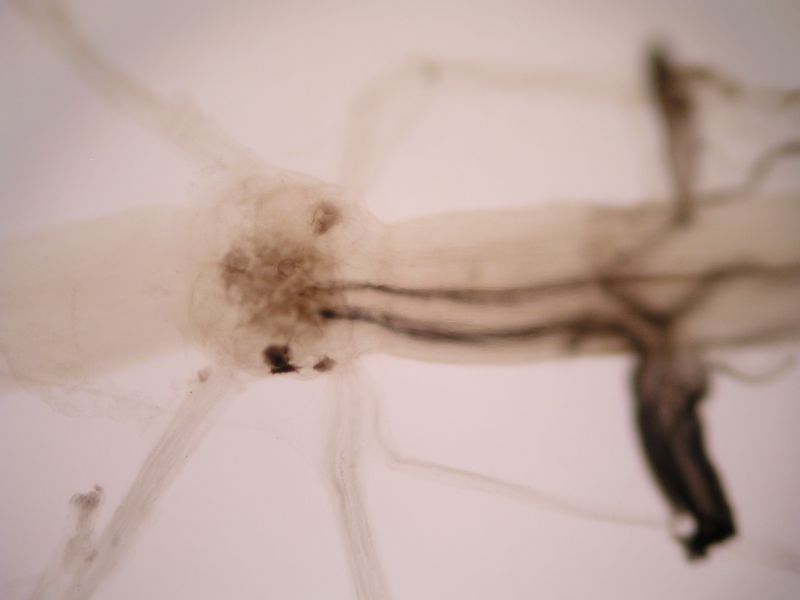

Supplement: Supplemental Information 2 — Micrographs of N3 backfills. Images have been reduced in size. [file peerj-03-1112-s003.zip › A1N3 low 2007 09 10 (20).jpg]

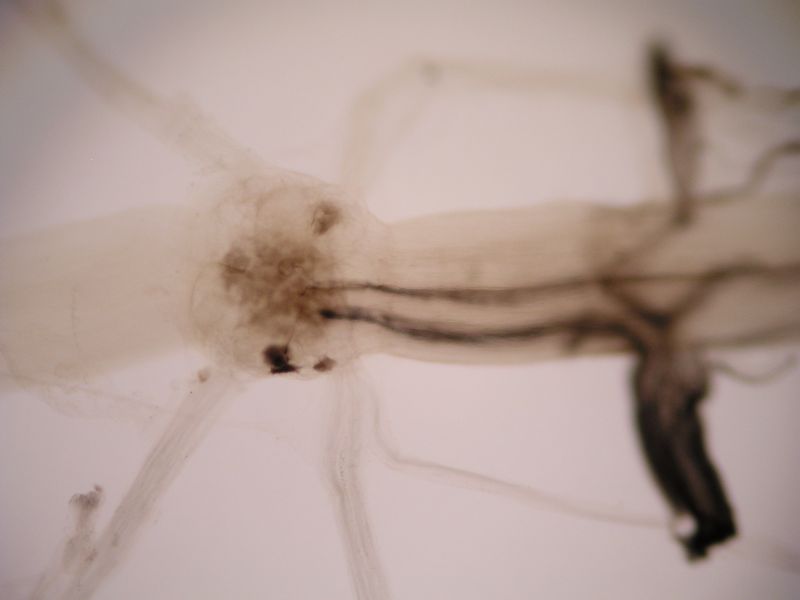

Supplement: Supplemental Information 2 — Micrographs of N3 backfills. Images have been reduced in size. [file peerj-03-1112-s003.zip › A1N3 low 2007 09 10 (21).jpg]

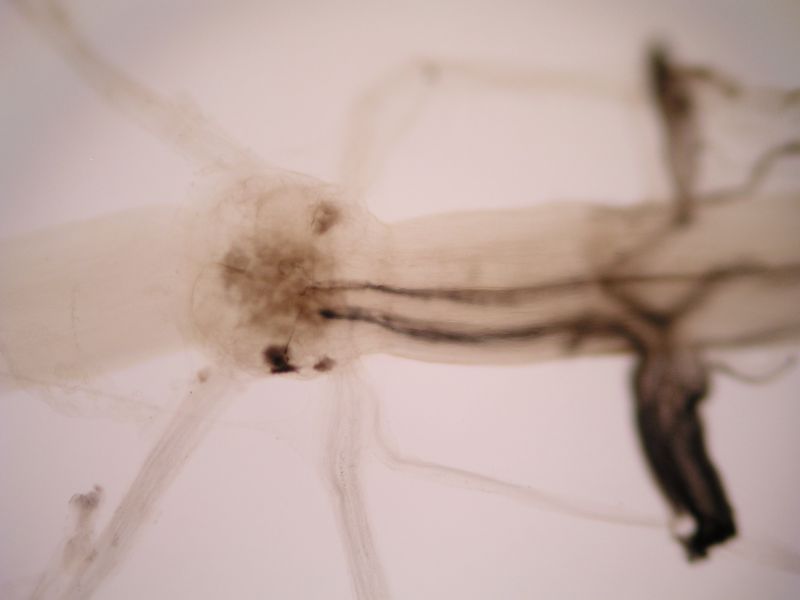

Supplement: Supplemental Information 2 — Micrographs of N3 backfills. Images have been reduced in size. [file peerj-03-1112-s003.zip › A1N3 low 2007 09 10 (22).jpg]

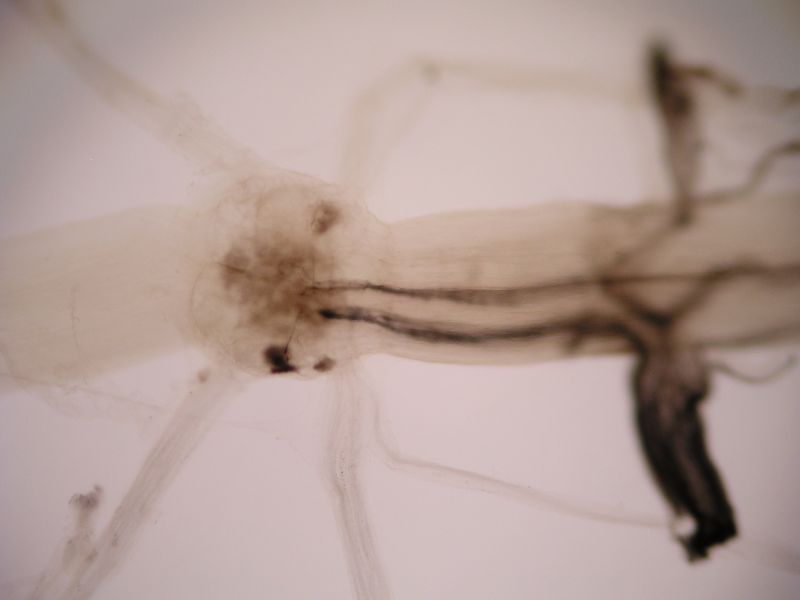

Supplement: Supplemental Information 2 — Micrographs of N3 backfills. Images have been reduced in size. [file peerj-03-1112-s003.zip › A1N3 low 2007 09 10 (23).jpg]

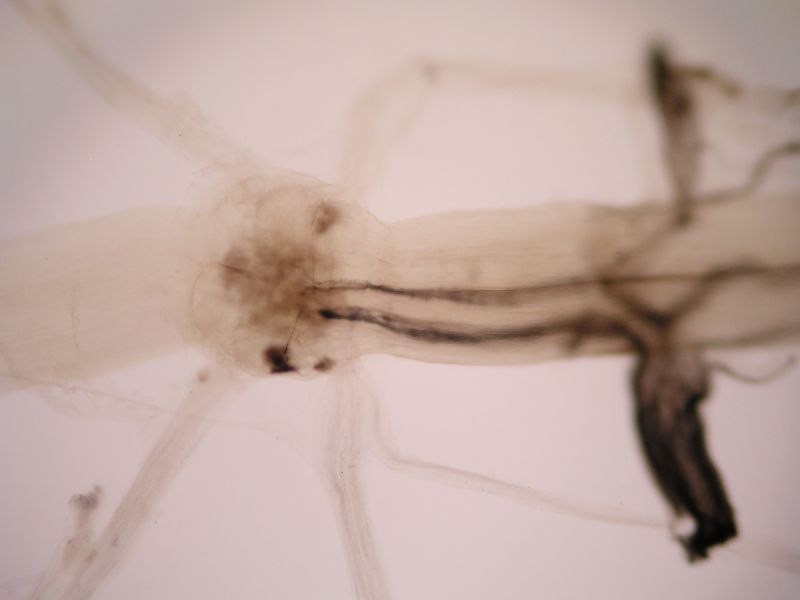

Supplement: Supplemental Information 2 — Micrographs of N3 backfills. Images have been reduced in size. [file peerj-03-1112-s003.zip › A1N3 low 2007 09 10 (24).jpg]

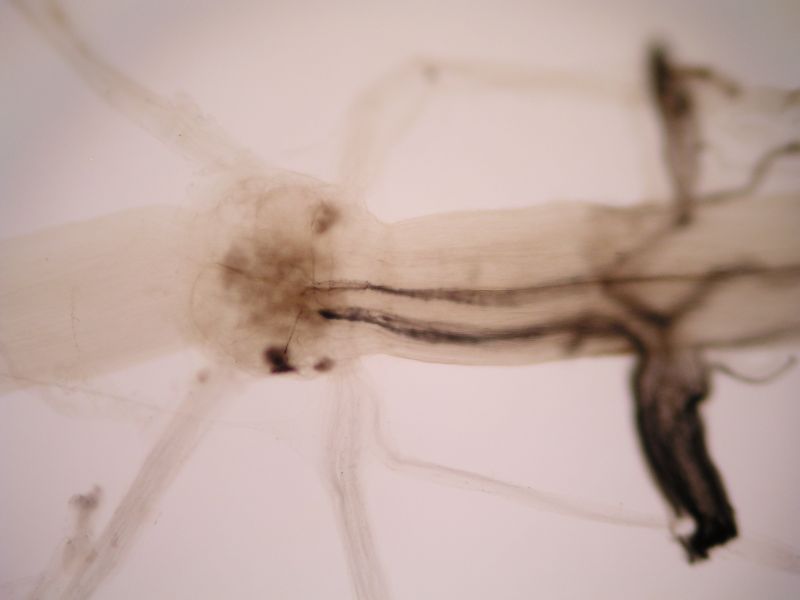

Supplement: Supplemental Information 2 — Micrographs of N3 backfills. Images have been reduced in size. [file peerj-03-1112-s003.zip › A1N3 low 2007 09 10 (25).jpg]

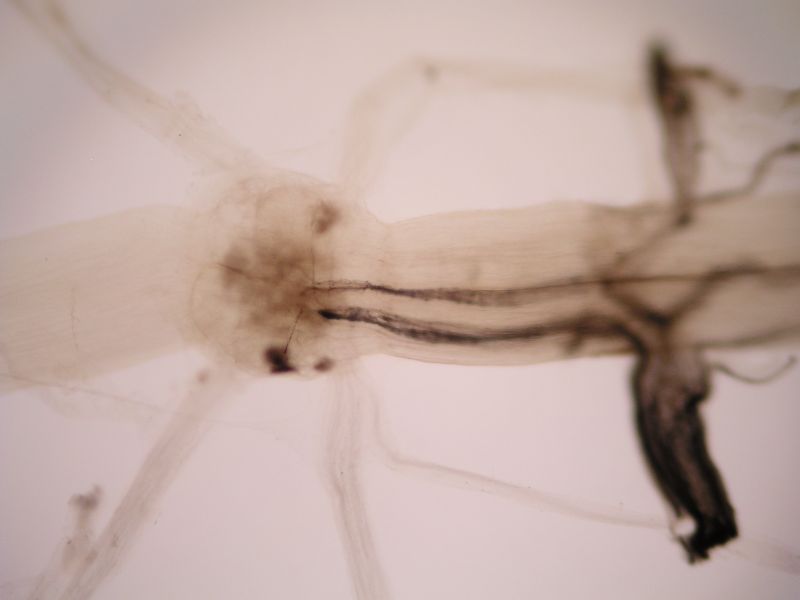

Supplement: Supplemental Information 2 — Micrographs of N3 backfills. Images have been reduced in size. [file peerj-03-1112-s003.zip › A1N3 low 2007 09 10 (26).jpg]

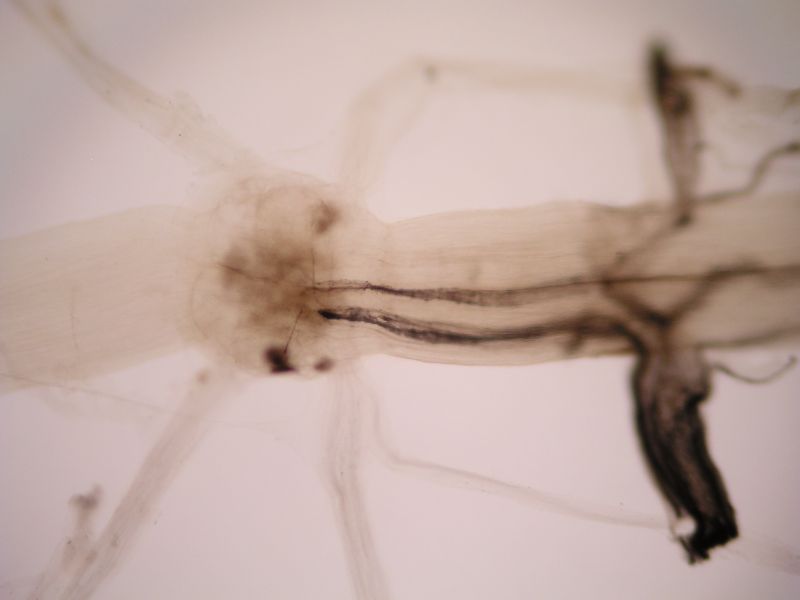

Supplement: Supplemental Information 2 — Micrographs of N3 backfills. Images have been reduced in size. [file peerj-03-1112-s003.zip › A1N3 low 2007 09 10 (27).jpg]

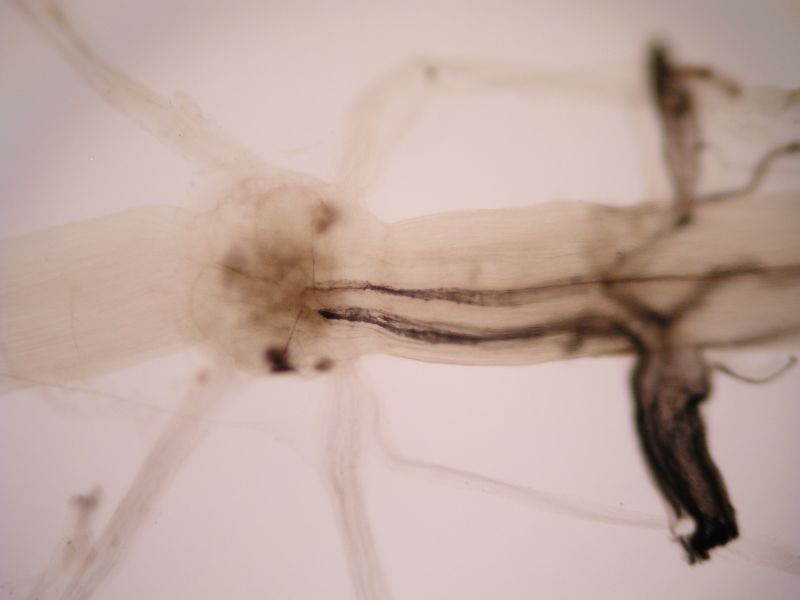

Supplement: Supplemental Information 2 — Micrographs of N3 backfills. Images have been reduced in size. [file peerj-03-1112-s003.zip › A1N3 low 2007 09 10 (28).jpg]

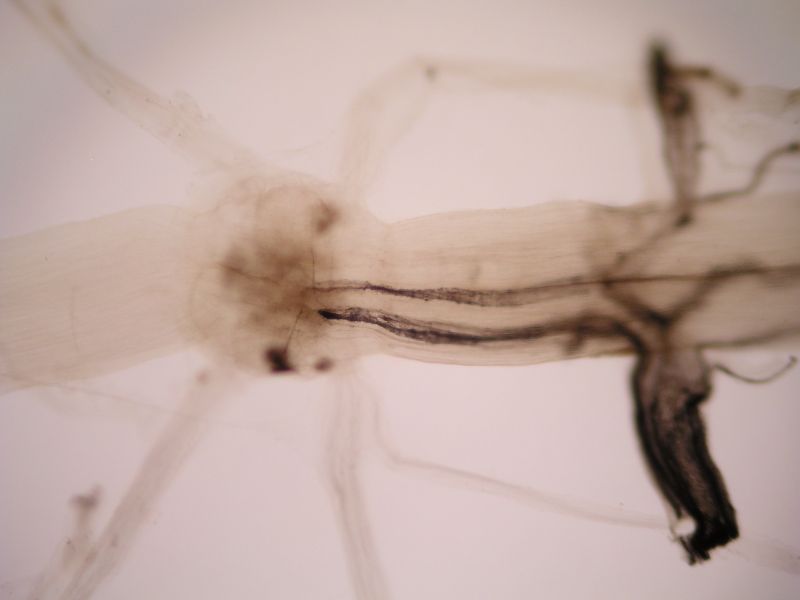

Supplement: Supplemental Information 2 — Micrographs of N3 backfills. Images have been reduced in size. [file peerj-03-1112-s003.zip › A1N3 low 2007 09 10 (29).jpg]

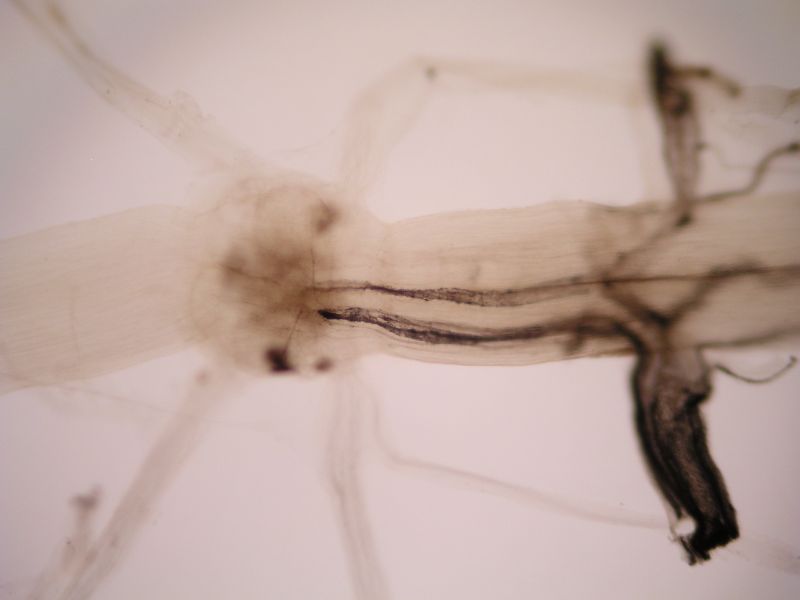

Supplement: Supplemental Information 2 — Micrographs of N3 backfills. Images have been reduced in size. [file peerj-03-1112-s003.zip › A1N3 low 2007 09 10 (30).jpg]

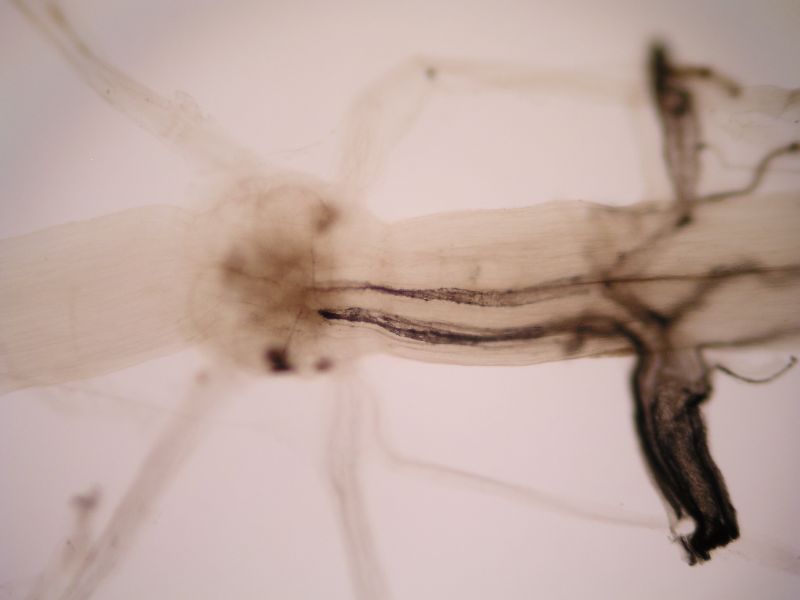

Supplement: Supplemental Information 2 — Micrographs of N3 backfills. Images have been reduced in size. [file peerj-03-1112-s003.zip › A1N3 low 2007 09 10 (31).jpg]

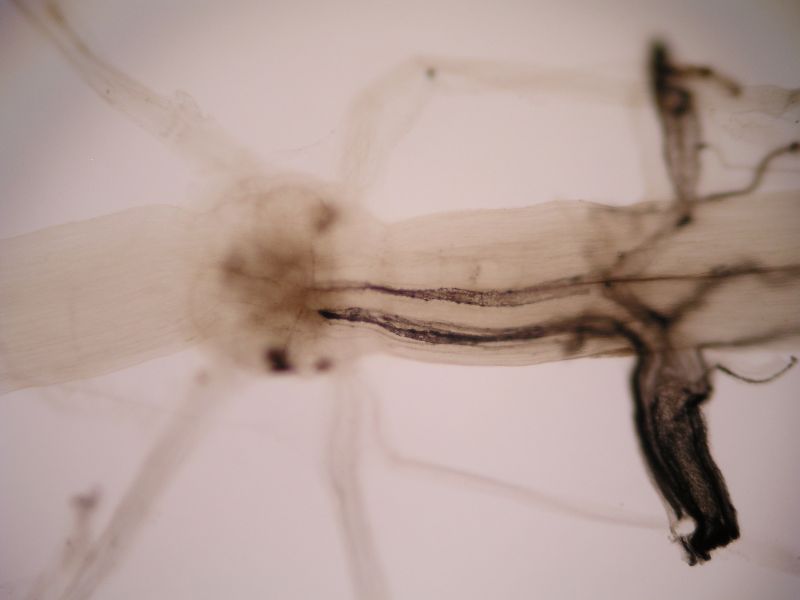

Supplement: Supplemental Information 2 — Micrographs of N3 backfills. Images have been reduced in size. [file peerj-03-1112-s003.zip › A1N3 low 2007 09 10 (32).jpg]

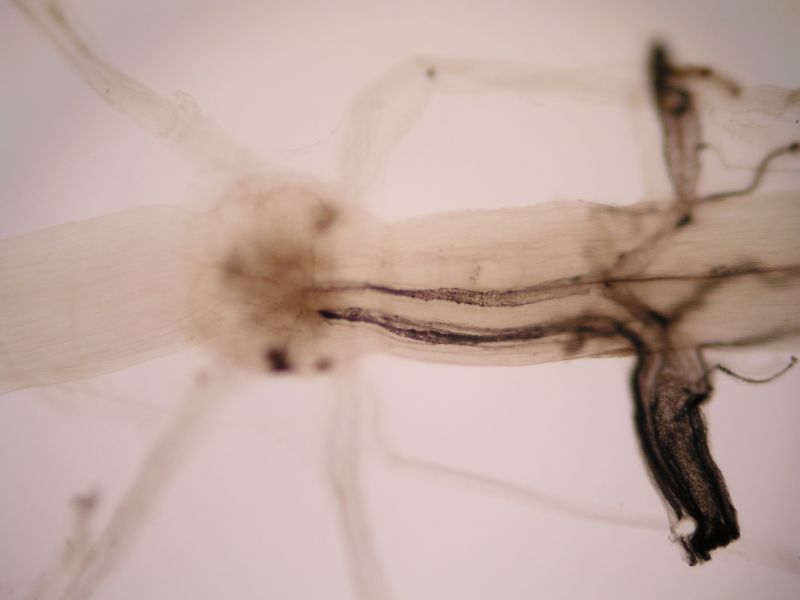

Supplement: Supplemental Information 2 — Micrographs of N3 backfills. Images have been reduced in size. [file peerj-03-1112-s003.zip › A1N3 low 2007 09 10 (33).jpg]

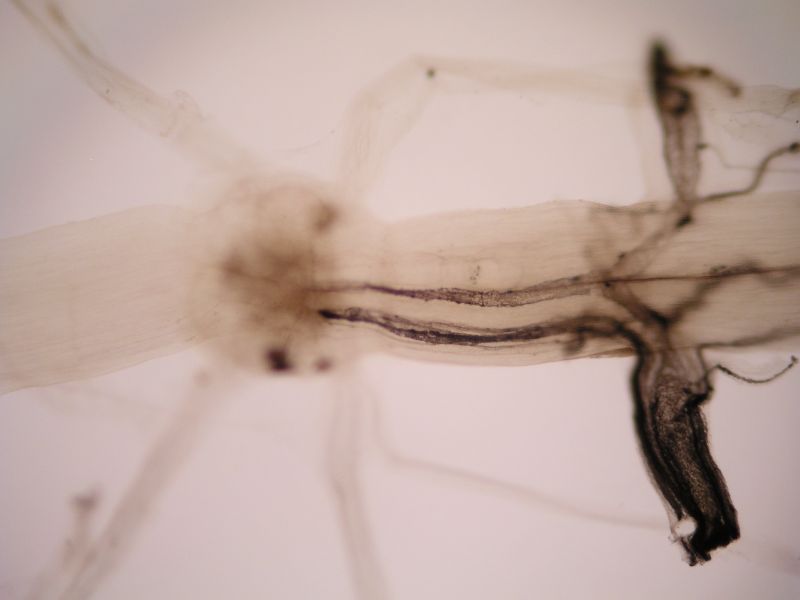

Supplement: Supplemental Information 2 — Micrographs of N3 backfills. Images have been reduced in size. [file peerj-03-1112-s003.zip › A1N3 low 2007 09 10 (34).jpg]

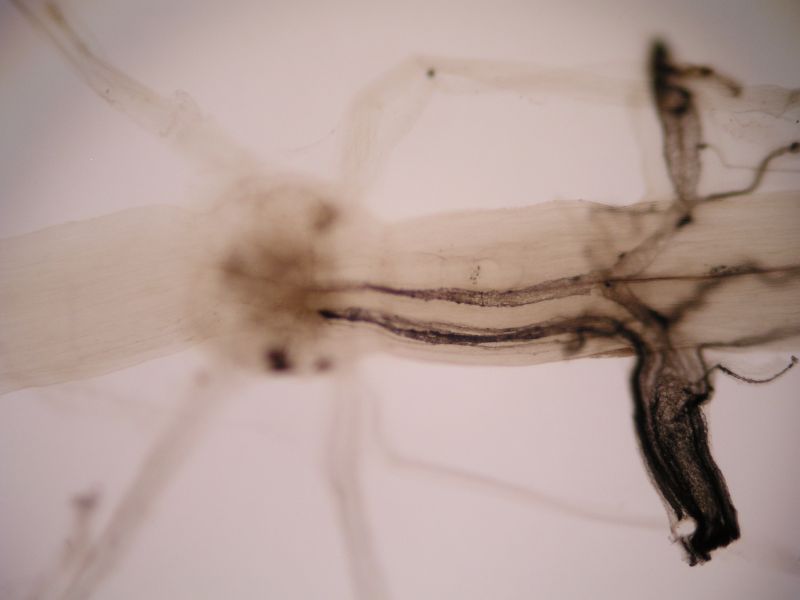

Supplement: Supplemental Information 2 — Micrographs of N3 backfills. Images have been reduced in size. [file peerj-03-1112-s003.zip › A1N3 low 2007 09 10 (35).jpg]

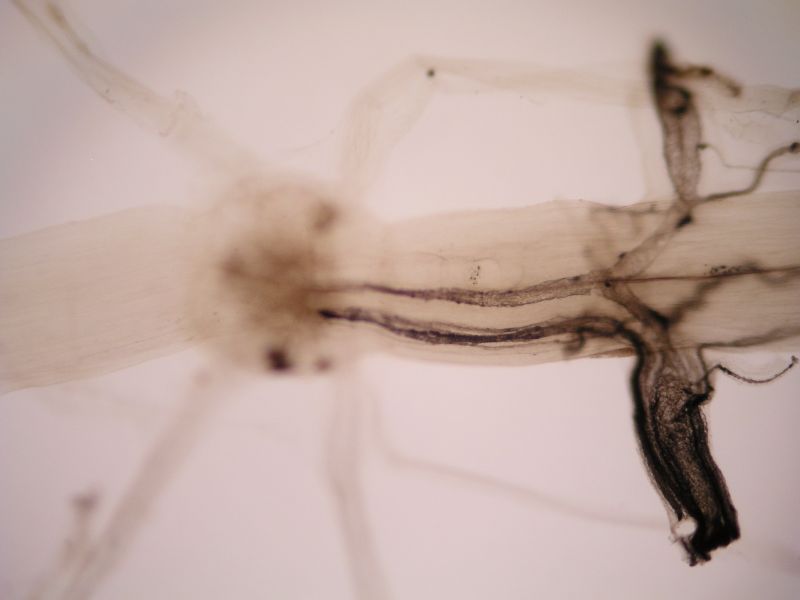

Supplement: Supplemental Information 2 — Micrographs of N3 backfills. Images have been reduced in size. [file peerj-03-1112-s003.zip › A1N3 low 2007 09 10 (36).jpg]

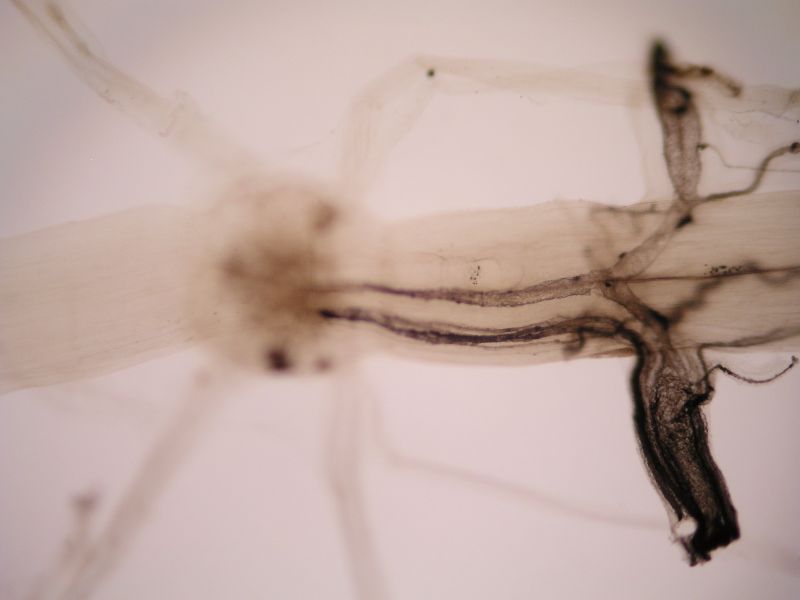

Supplement: Supplemental Information 2 — Micrographs of N3 backfills. Images have been reduced in size. [file peerj-03-1112-s003.zip › A1N3 low 2007 09 10 (37).jpg]

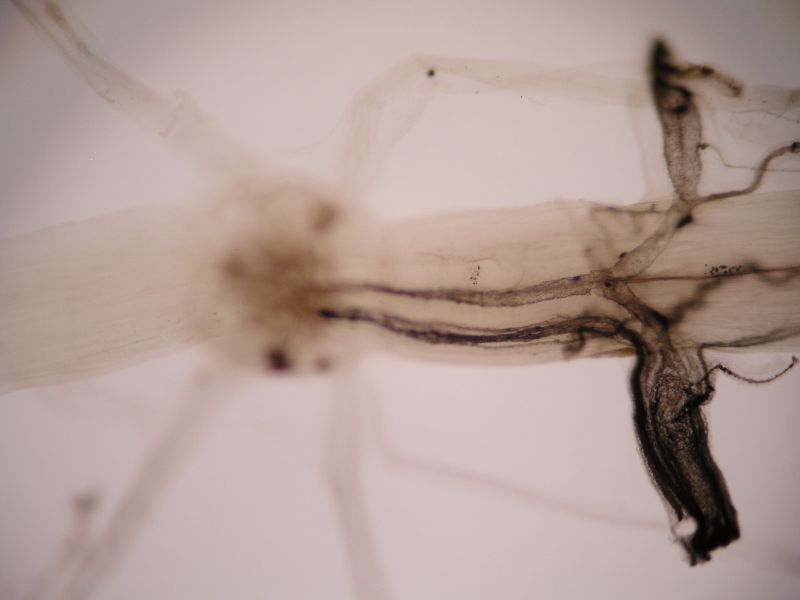

Supplement: Supplemental Information 2 — Micrographs of N3 backfills. Images have been reduced in size. [file peerj-03-1112-s003.zip › A1N3 low 2007 09 10 (38).jpg]

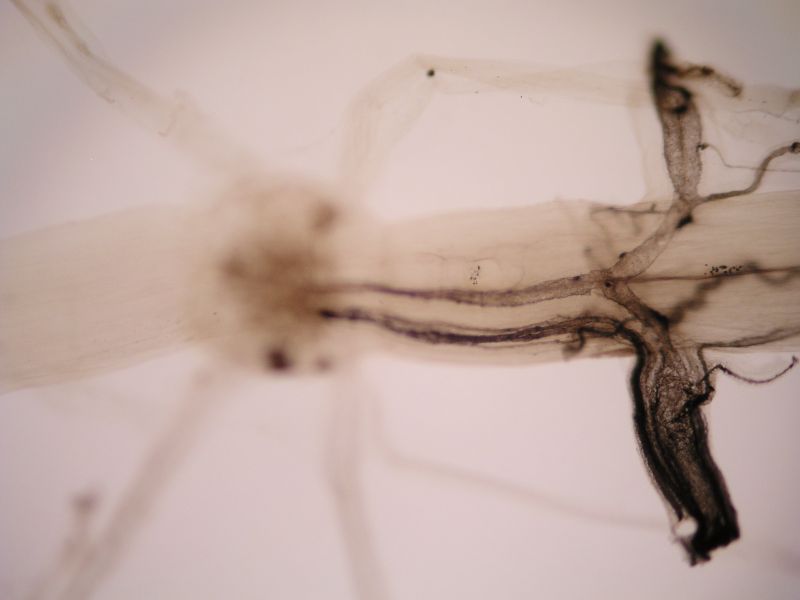

Supplement: Supplemental Information 2 — Micrographs of N3 backfills. Images have been reduced in size. [file peerj-03-1112-s003.zip › A1N3 low 2007 09 10 (39).jpg]

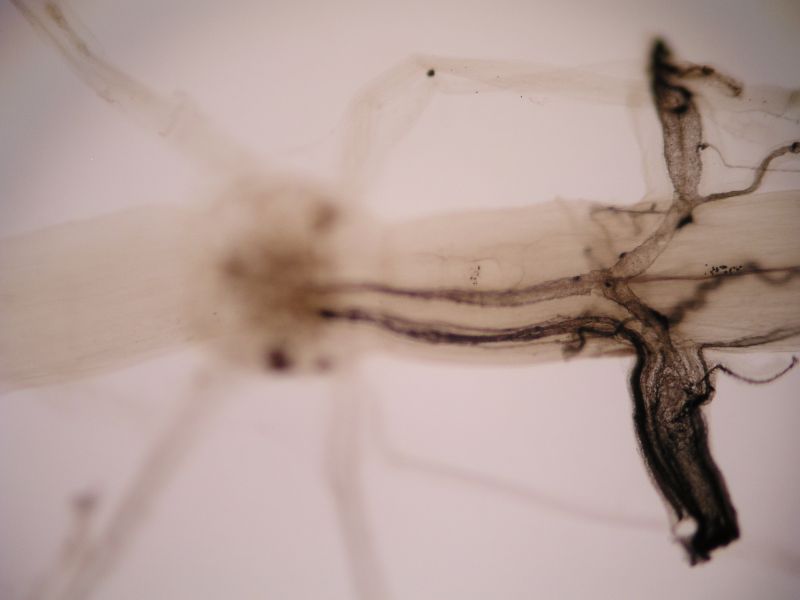

Supplement: Supplemental Information 2 — Micrographs of N3 backfills. Images have been reduced in size. [file peerj-03-1112-s003.zip › A1N3 low 2007 09 10 (40).jpg]

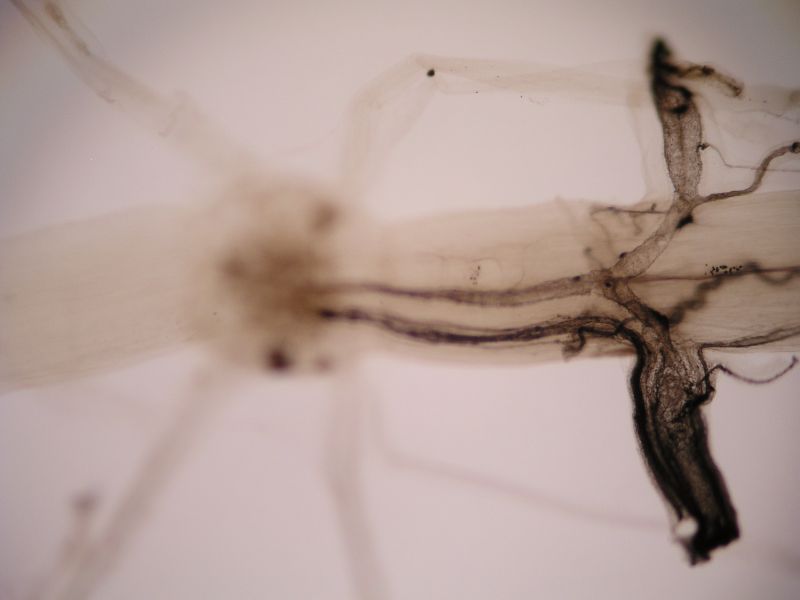

Supplement: Supplemental Information 2 — Micrographs of N3 backfills. Images have been reduced in size. [file peerj-03-1112-s003.zip › A1N3 low 2007 09 10 (41).jpg]

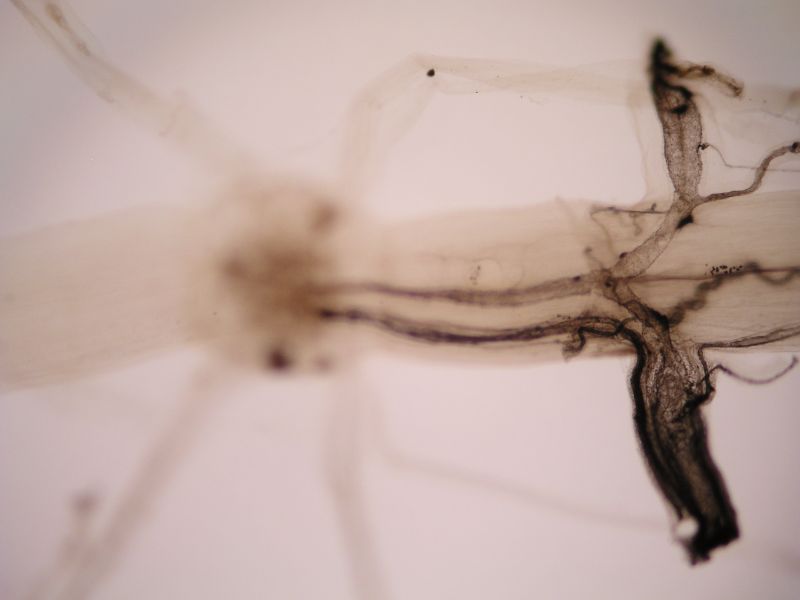

Supplement: Supplemental Information 2 — Micrographs of N3 backfills. Images have been reduced in size. [file peerj-03-1112-s003.zip › A1N3 low 2007 09 10 (42).jpg]

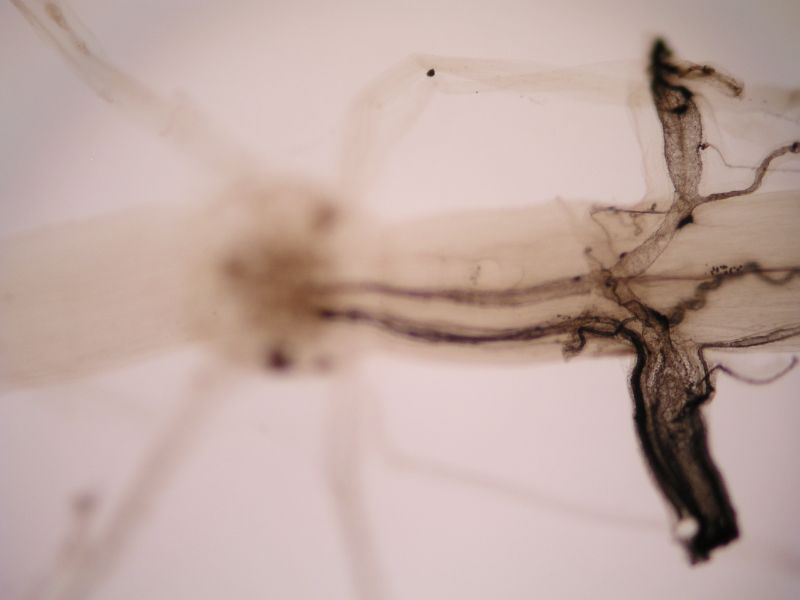

Supplement: Supplemental Information 2 — Micrographs of N3 backfills. Images have been reduced in size. [file peerj-03-1112-s003.zip › A1N3 low 2007 09 10 (43).jpg]

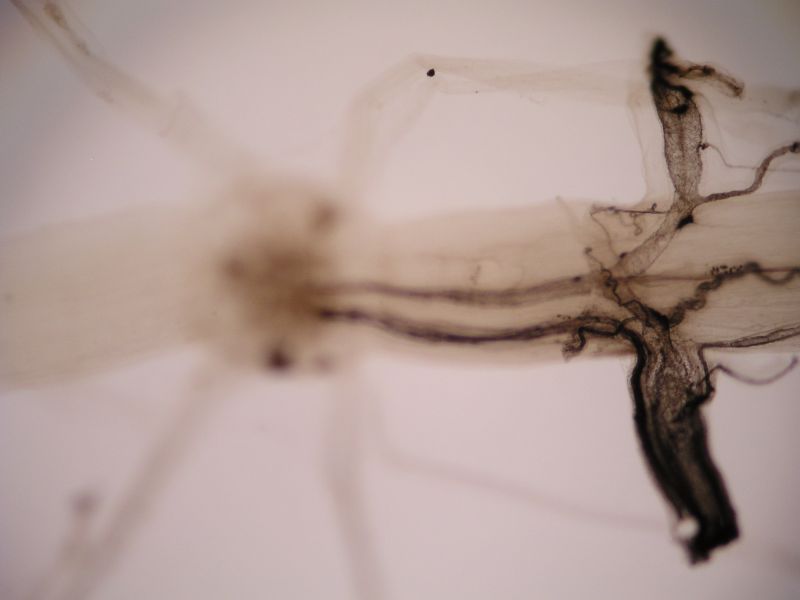

Supplement: Supplemental Information 2 — Micrographs of N3 backfills. Images have been reduced in size. [file peerj-03-1112-s003.zip › A1N3 low 2007 09 10.jpg]

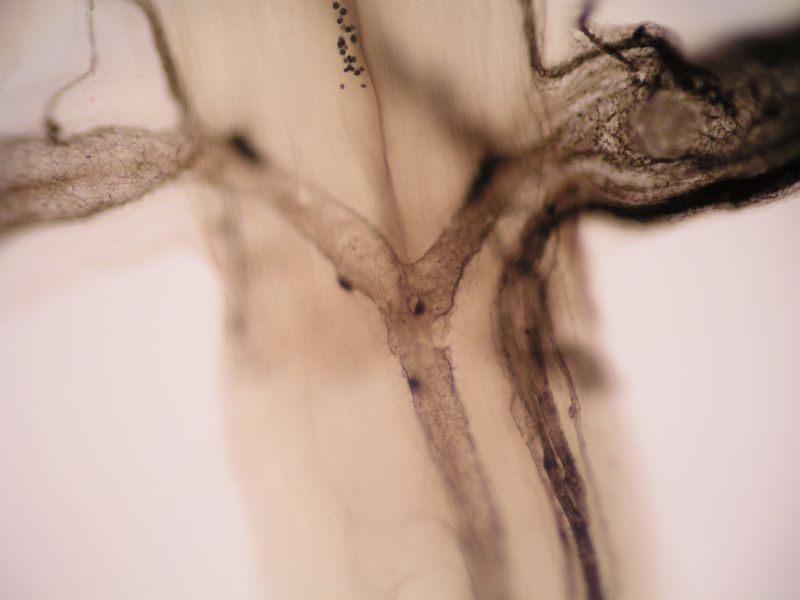

Supplement: Supplemental Information 2 — Micrographs of N3 backfills. Images have been reduced in size. [file peerj-03-1112-s003.zip › A1N3 MoG axon 2009 07 10.jpg]

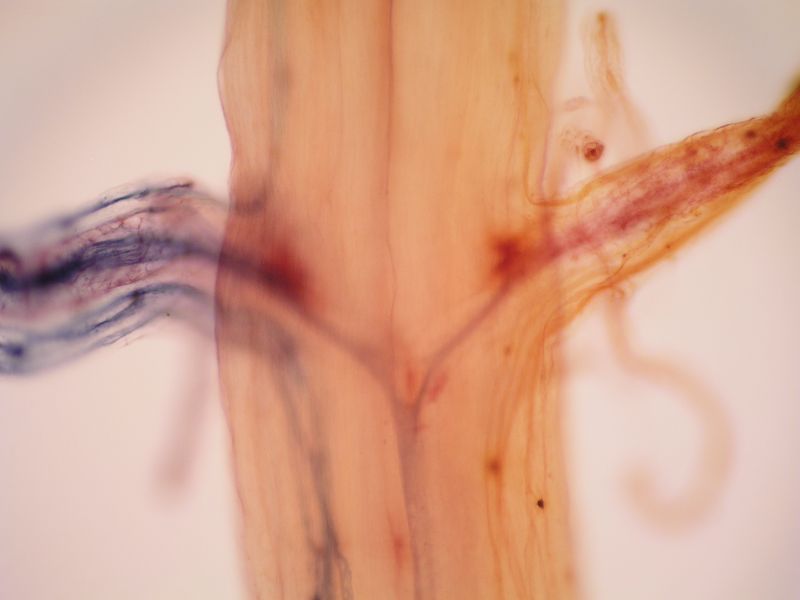

Supplement: Supplemental Information 2 — Micrographs of N3 backfills. Images have been reduced in size. [file peerj-03-1112-s003.zip › A1N3 Mog synapse 2009 07 15 (1).jpg]

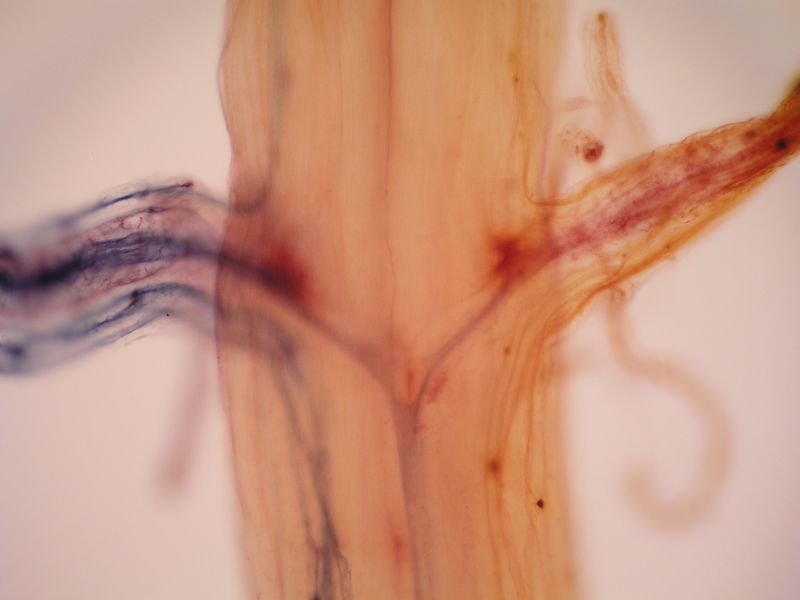

Supplement: Supplemental Information 2 — Micrographs of N3 backfills. Images have been reduced in size. [file peerj-03-1112-s003.zip › A1N3 Mog synapse 2009 07 15 (2).jpg]

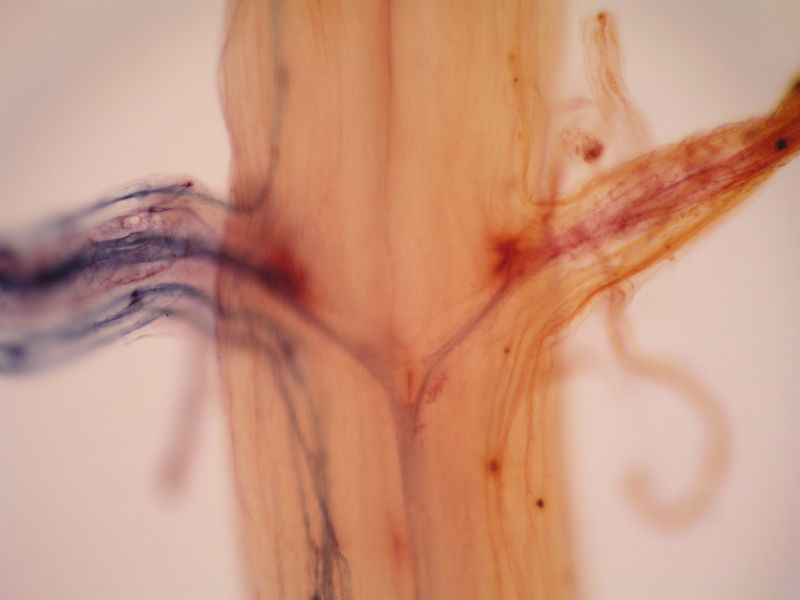

Supplement: Supplemental Information 2 — Micrographs of N3 backfills. Images have been reduced in size. [file peerj-03-1112-s003.zip › A1N3 Mog synapse 2009 07 15 (3).jpg]

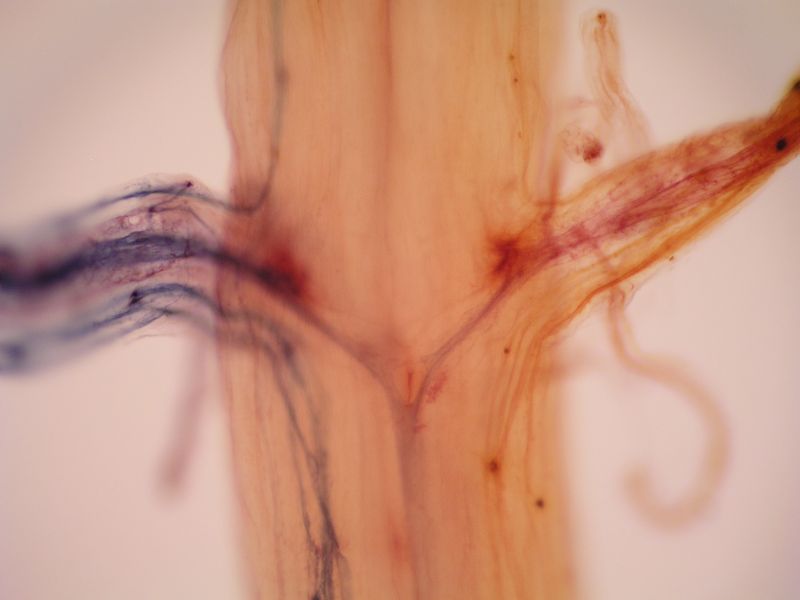

Supplement: Supplemental Information 2 — Micrographs of N3 backfills. Images have been reduced in size. [file peerj-03-1112-s003.zip › A1N3 Mog synapse 2009 07 15 (4).jpg]

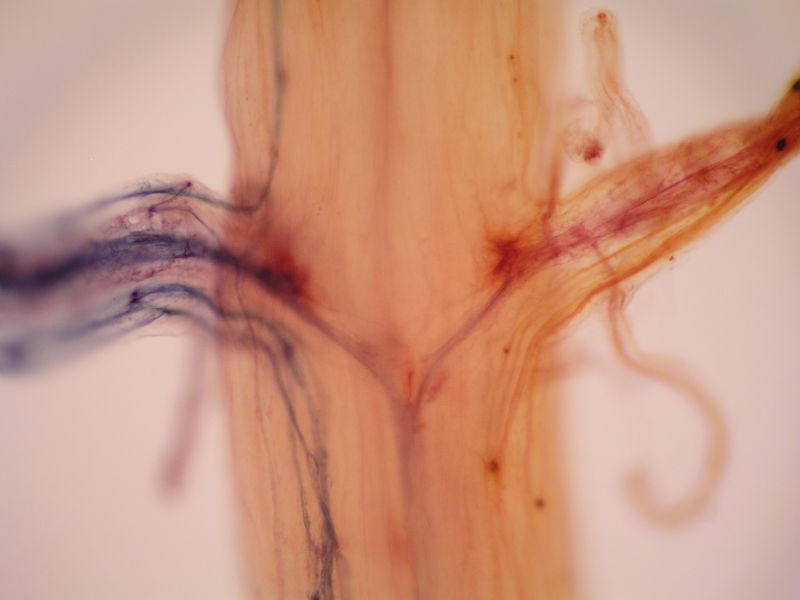

Supplement: Supplemental Information 2 — Micrographs of N3 backfills. Images have been reduced in size. [file peerj-03-1112-s003.zip › A1N3 Mog synapse 2009 07 15 (5).jpg]

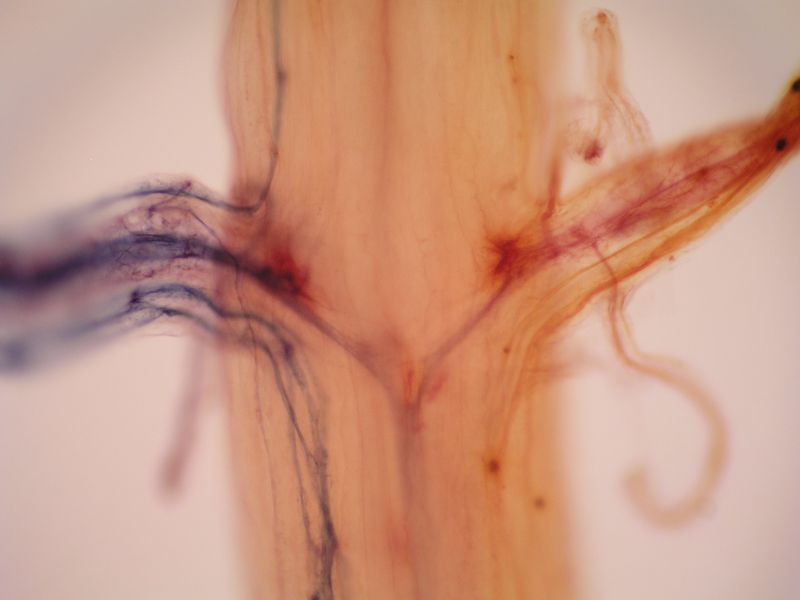

Supplement: Supplemental Information 2 — Micrographs of N3 backfills. Images have been reduced in size. [file peerj-03-1112-s003.zip › A1N3 Mog synapse 2009 07 15 (6).jpg]

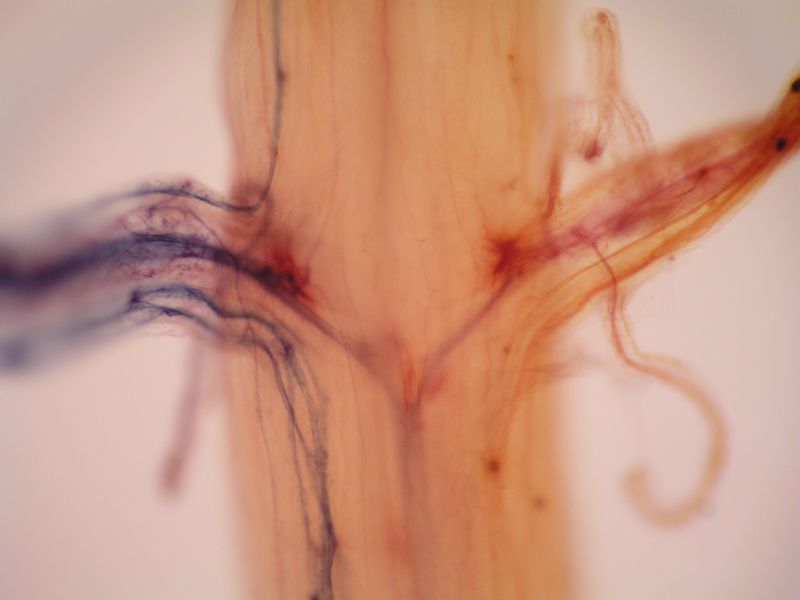

Supplement: Supplemental Information 2 — Micrographs of N3 backfills. Images have been reduced in size. [file peerj-03-1112-s003.zip › A1N3 Mog synapse 2009 07 15 (7).jpg]

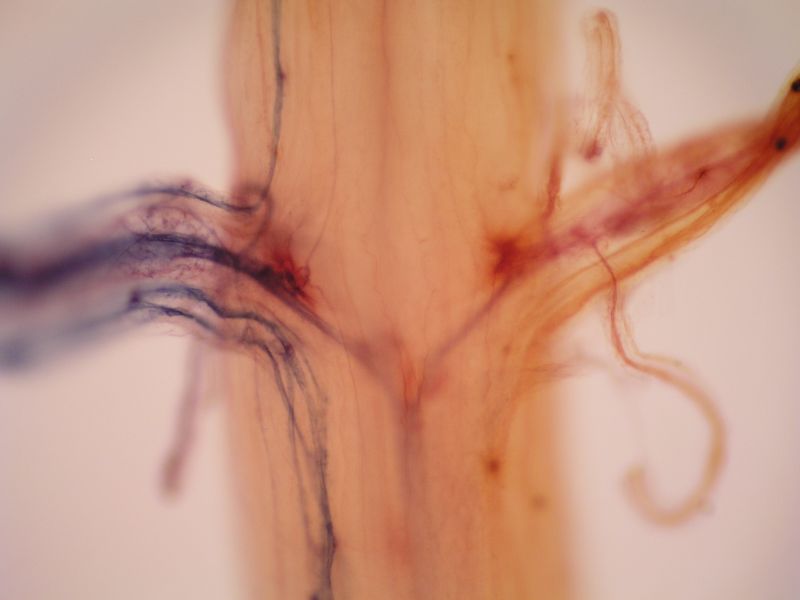

Supplement: Supplemental Information 2 — Micrographs of N3 backfills. Images have been reduced in size. [file peerj-03-1112-s003.zip › A1N3 Mog synapse 2009 07 15 (8).jpg]

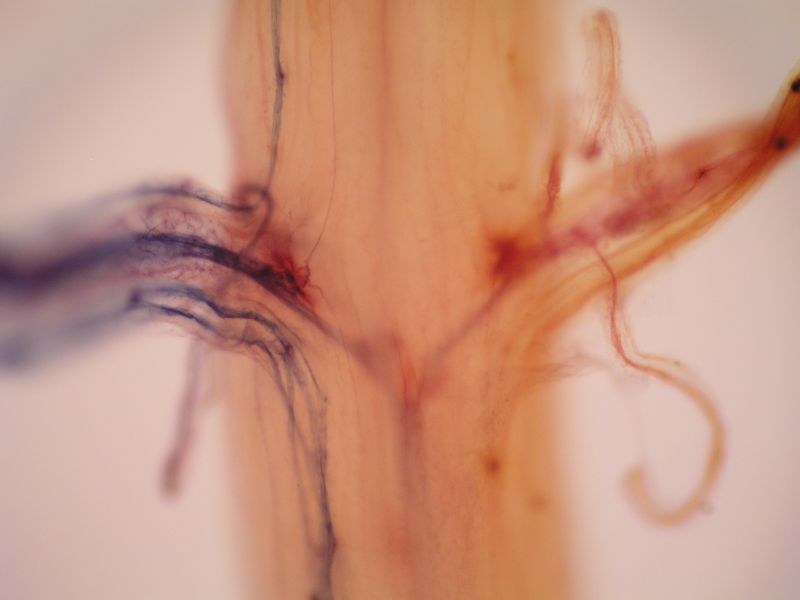

Supplement: Supplemental Information 2 — Micrographs of N3 backfills. Images have been reduced in size. [file peerj-03-1112-s003.zip › A1N3 Mog synapse 2009 07 15 (9).jpg]

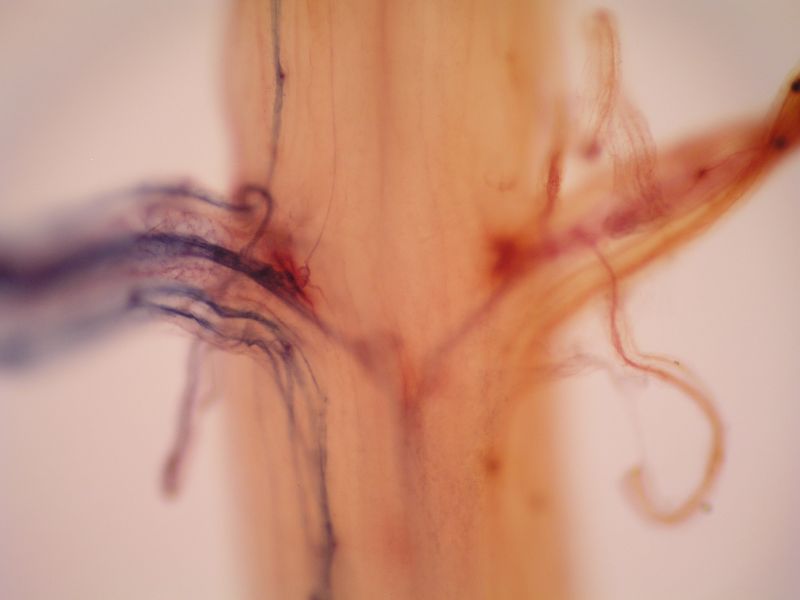

Supplement: Supplemental Information 2 — Micrographs of N3 backfills. Images have been reduced in size. [file peerj-03-1112-s003.zip › A1N3 Mog synapse 2009 07 15 (10).jpg]

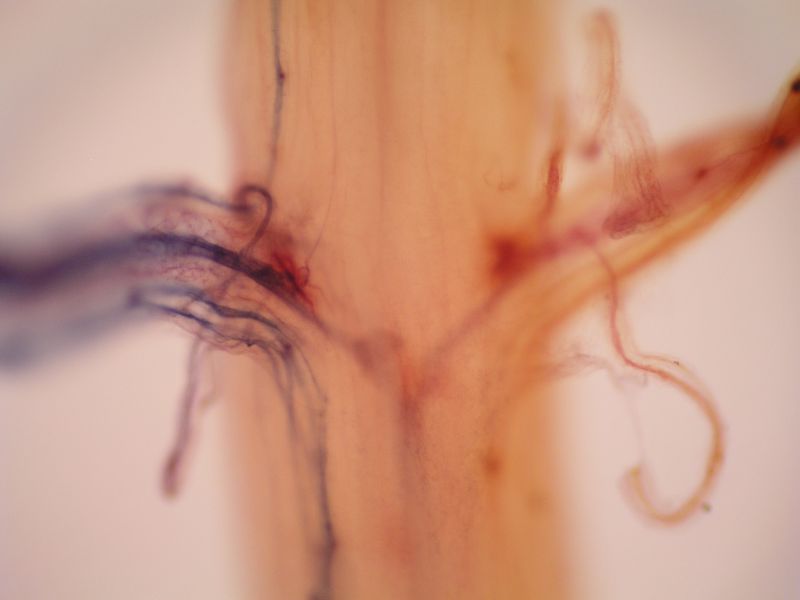

Supplement: Supplemental Information 2 — Micrographs of N3 backfills. Images have been reduced in size. [file peerj-03-1112-s003.zip › A1N3 Mog synapse 2009 07 15.jpg]
